# Cytochrome c oxidase barcodes for aquatic oligochaete identification: development of a Swiss reference database

**DOI:** 10.7717/peerj.4122

**Published:** 2017-12-06

**Authors:** Régis Vivien, Maria Holzmann, Inge Werner, Jan Pawlowski, Michel Lafont, Benoit J.D. Ferrari

**Affiliations:** 1 Swiss Centre for Applied Ecotoxicology (Ecotox Centre) Eawag-EPFL, Lausanne/Dübendorf, Switzerland; 2 Department of Genetics and Evolution, University of Geneva, Geneva, Switzerland; 3 Laboratoire d’Ecologie des Hydrosystèmes Naturels et Anthropisés, Université Lyon I, Villeurbanne, France

**Keywords:** DNA barcoding, Aquatic oligochaetes, Genetic diversity, Biomonitoring

## Abstract

**Introduction:**

Aquatic oligochaetes represent valuable indicators of the quality of sediments of watercourses and lakes, but their difficult identification based on morphological criteria compromises their more common use for eco-diagnostic analyses. This issue could be overcome by using DNA barcodes for species identification. A 10% threshold of cytochrome c oxidase (COI) divergence was proposed for differentiating between oligochaete species based on molecular and morphological data. A Swiss database of COI sequences of aquatic oligochaetes was initiated in 2012. The aim of this study is to complement the Swiss oligochaete database of COI sequences and to confirm the relevance of this threshold for species delimitation.

**Methods:**

We sequenced the COI sequence of 216 specimens collected in different regions of Switzerland and ITS2 region of some lineages whose delimitation with COI data was doubtful.

**Results:**

We distinguished 53 lineages, among which 34 were new for Switzerland and 17 sequenced for the first time. All the lineages were separated by more than 10% of COI variation, with the exception of some species within Nais and Uncinais. In these two genera, the threshold was lowered to 8% to be congruent with the morphological analysis. The total number of lineages reported so far for Switzerland is 75, including 59 morphospecies or unidentified species and 16 cryptic species.

**Discussion:**

Our study shows that the threshold of 10% of COI divergence is generally appropriate to distinguish aquatic oligochaete lineages, but that it must be adjusted for some species. The database reported here will be complemented in the future in parallel to the development of genetic oligochaete indices.

## Introduction

Freshwater oligochaetes include a large number of species showing a wide range of tolerance to chemical pollution (Rodriguez & Reynoldson, 2011). For some decades they have been used in many countries for assessing the biological quality of river and lake sediments (e.g., Särkä, 1994; Milbrink, Timm & Lundberg, 2002; Lang, 1997). Different methods based on the analysis of oligochaete communities have been proposed to characterize the ecological status of fine sediments in rivers and lakes (e.g., Lafont et al., 2010; Lafont et al., 2012), and of the compartments of coarse sediments and hyporheic zone in rivers (Lafont & Vivier, 2006).

Difficulties related to oligochaete species identification based on morphological features constitute a major obstacle for a more common use of this taxonomic group for eco-diagnostic analyses. The morphological approach does not allow to identify the totality of specimens present in a sample for three main reasons. First, an important number of species (in Tubificinae, Lumbriculidae and Enchytraeidae) can be identified only when the specimens are in a mature state. Secondly, the identification of most species in Lumbriculidae and Enchytraeidae requires dissection, which is too time-consuming to be performed in routine analyses. Thirdly, many aquatic oligochaetes include cryptic species undetectable morphologically (e.g., Beauchamp et al., 2001; Gustafsson, Price & Erséus, 2009; Bely & Wray, 2004).

The identification of oligochaete species using DNA barcodes can overcome the issues associated with the morphological identification, facilitate their use in biomonitoring and lead to the improvement of the ecological diagnostics. The mitochondrial COI gene is an effective barcode for oligochaetes (Rodriguez & Reynoldson, 2011; Rougerie et al., 2009; Kvist, Sarkar & Erséus, 2010; Martinsson et al., 2013) and ITS2 region was used in some studies as a complementary marker to COI (Achurra & Erséus, 2013; Envall, Gustavsson & Erséus, 2012). A 10% threshold of COI divergence has been suggested for segregating between aquatic oligochaetes species (Erséus & Gustafsson, 2009; Zhou et al., 2010). Vivien et al. (2015) sequenced the COI and ITS2 markers of a high number of aquatic oligochaete specimens and showed that the distinction of the vast majority of 41 lineages with the 10% threshold of COI divergence was in agreement with the ITS2 data. In 2012, a database of COI sequences of aquatic oligochaetes collected in Switzerland was initiated (Vivien et al., 2015). COI sequences were assigned to 26 morphospecies and cryptic species were detected in the common species *Tubifex tubifex*, *Limnodrilus hoffmeisteri* and *Eiseniella tetraedra*. The results showed that the morphological identification largely underestimated oligochaete diversity. A high throughput sequencing (HTS) approach that allows to sequence the specimens of a large number of samples at the same time has been proposed as a cost effective way to assess biodiversity in routine biomonitoring (Bohmann et al., 2014). The application of HTS on samples composed of genetically tagged specimens could constitute a promising way to both identify the species present in a sample and estimate their abundances. In comparison to Sanger sequencing, this approach would allow to reduce the duration and the cost of the analyses (Shokralla et al., 2014).

In the perspective of developing a HTS based oligochaete index, we intended to complement the Swiss database of COI sequences of aquatic oligochaetes by analysing specimens collected in different parts of Switzerland. We also tested the relevance of the 10% threshold of COI divergence to discriminate between aquatic oligochaete lineages. The ITS2 region of two lineages whose delimitation with COI data was doubtful was also sequenced.

## Material and Methods

### Sampling and morphological analysis

The sampling was performed between 2013 and 2017 in eleven streams/rivers of four Swiss cantons (Geneva, Vaud, Bern and Lucerne) and at ten sites in Lake Geneva (Table S1). Sediments from the upper 10 cm were sampled using a shovel, a Surber type net (0.2 mm mesh size) or an Ekman type grab sampler (deep zones of lake). Before sieving, the biological material was either fixed with absolute ethanol or 5% formalin. The fixation of oligochaetes with formalin does not prevent genetic analyses if the specimens are preserved in this medium for a short time (<1 week) (Vivien, Ferrari & Pawlowski, 2016). At the laboratory, sediment samples were sieved (using a sieve of 0.5 mm mesh size). The material retained in the sieve was transferred into a Tupperware box, then examined under a stereomicroscope and oligochaete specimens were extracted. Each specimen was cut in two. The anterior parts were fixed and preserved in 5% formalin for morphological analysis and the posterior parts were preserved in 100% ethanol for DNA analysis. Anterior parts were cleared in an acid lactic/glycerol solution and mounted between slide and coverslip in a coating solution composed of lactic acid, glycerol and polyvinylic alcohol (Mowiol 4–88). Oligochaete specimens were identified to the lowest level (species if possible). The identification keys of Sperber (1950) and Timm (2009) were mainly used. The anterior parts served as reference vouchers and will be deposited at the Museum of Natural History of the city of Geneva.

### Genetic analyses

Total genomic DNA was extracted using guanidine thiocyanate as described by Tkach & Pawlowski (1999). A fragment of 658 base pairs of the COI gene was amplified from each DNA extract using LCO 1490 and HCO 2198 primers (Folmer et al., 1994). The ITS2 rRNA region was amplified from some DNA extracts using the primers described in Navajas et al. (1998). The PCRs were performed in a total volume of 20 μl containing 0.6 Unit of Taq polymerase (Roche, Basel, Switzerland), 2 μl of the 10× buffer (Roche) containing 20 mM of MgCl2, 0.5 μl of each primer (10 mM each), 0.4 μl of a mix containing 10 mM of each dNTP (Roche, Basel, Switzerland) and 0.8 μl of template DNA of undetermined concentration. The PCR comprised an initial denaturation step at 95 °C for 5 min, followed by 35 cycles of denaturation at 95 °C for 40 s, annealing at 44 °C for 45 s and elongation at 72 °C for 1 min, with a final elongation step at 72 °C for 8 min.

COI and ITS2 PCR products were then bi-directionally Sanger sequenced on an ABI 3031 automated sequencer (Applied Biosystems, Foster City, CA, USA) using the same primers and following the manufacturer’s protocol. The raw sequence editing and the generation of contiguous sequences were accomplished using CodonCode Aligner (CodonCode Corporation, Centerville, MA, USA). Multiple sequence alignments were automatically generated using Muscle (Edgar, 2004) as implemented in Seaview program (Gouy, Guindon & Gascuel, 2010).

Phylogenetic trees comprising the COI sequences obtained in the present work and in our previous work (Vivien et al., 2015) were constructed using maximum likelihood phylogeny (PhyML 3.0) as implemented in ATGC: PhyML (Guindon et al., 2010). An automatic model selection based on Akaike Information Criterion (AIC) was used for PhyML 3.0 yielding in a GTR substitution model being selected for the analysis. Additional trees were constructed using FastMe 2.0, a distance based phylogeny inference program as implemented in ATGC: FastMe (Lefort, Desper & Gascuel, 2015). Two substitution models (F84 and T93) were tested including tree refinement with Subtree Pruning and Regrafting (SPR). Bootstrap values are based on 100 replicates for all analyses.

Two trees combining sequences with a divergence <5% were constructed: one tree comprising all Tubificinae lineages and some lineages of the other families/subfamilies; a second tree comprising all Naidinae, Pristininae, Rhyacodrilinae, Lumbriculidae, Lumbricidae, Enchytraeidae, Haplotaxidae and two lineages of Tubificinae. Additionally, two trees combining sequences with a divergence <1% were constructed (provided as Figs. S1 and S2): one tree for Tubificinae and another one for the remaining families/subfamilies mentioned above. On the four illustrated trees, the bootstrap values (BV) higher than 70% are shown.

A 10% threshold of COI divergence was applied to distinguish between species (Vivien et al., 2015). The intra and inter-lineage distances were calculated using the K2P model in MEGA 5.1 (Tamura et al., 2011). For the genetic distance calculations, we took into account all the sequences obtained in the present work and one or two sequence(s) per lineage of our database (Vivien et al., 2015). The sequences of our database used for distance calculations are represented in the trees. The discrimination of lineages diverging by a distance situated between 10 and 13.5% were considered as doubtful if the specimens corresponding to these sequences showed no morphological difference. ITS2 of these specimens were sequenced to confirm or not the segregation of the lineages.

The new COI sequences for Switzerland were compared to Genbank (NCBI) sequences using BLAST (http://www.ncbi.nlm.nih.gov/BLAST/Blast.cgi) and to BOLD sequences (http://www.boldsystems.org/index.php/IDS_OpenIdEngine), until July 2017. Our sequences and Genbank’s or Bold’s sequences were considered as belonging to the same species if their genetic divergence were ≤10%.

The COI and ITS2 sequences (raw data) are provided as  Files S1 and S2. The COI sequences are accessible in the European Nucleotide Archive (ENA) at LT598611–LT598612; LT598614, LT598615, LT598616, LT598617; LT598619, LT598620, LT598621; LT598625; LT598628, LT598629, LT598630, LT598631, LT598632, LT598633; LT899859, LT899860, LT899861, LT899862, LT899863, LT899864, LT899865, LT899866, LT899867; LT899869–LT899898; LT903797–LT903836; LT903777–LT903796; LT903837, LT903838, LT903839, LT903840, LT903841, LT903842, LT903843, LT903844, LT903845, LT903846; LT904767, LT904768, LT904769, LT904770, LT904771, LT904772, LT904773, LT904774, LT904775, LT904776; LT905357–LT905411; LT906396, LT906397, LT906398, LT906399, LT906400, LT906401; LT906407–LT906426. The ITS2 sequences are available at LT906402, LT906403, LT906404, LT906405, LT906406; LT906427, LT906428, LT906429, LT906430, LT906431, LT906432, LT906433.

## Results

### New COI data

A total of 180 specimens originating from stream/river sediments and 36 from lake sediments were sequenced (Table S1). These specimens belonged to five families: 140 specimens to Naididae (22 Naidinae, 117 Tubificinae, one Pristininae), 31 to Lumbriculidae, two to Lumbricidae, 41 to Enchytraeidae and two to Haplotaxidae (Table 1).

All the lineages obtained were separated by more than 10% of COI variation, with the exception of four lineages within the genera *Nais* and *Uncinais*. The minimal interlineage variation of the species *Nais christinae* and *Nais stolci/pardalis* was slightly >10%, while it was between 8.1 and 9 for the species *Nais alpina*, *Nais communis* (lineage N10), *Nais pseudobtusa* and *Uncinais uncinata*. These lineages were clearly differentiated by the morphological analysis and so the threshold of genetic variation of COI to discriminate the species in these two genera was fixed at 8%. The sequences within the lineage of *Limnodrilus udekemianus* and the lineage of *Globulidrilus riparius* E11 presented a genetic variation situated between 10 and 13.5%. The variation within *L. udekemianus* was between 6.7 and 10.7%. The three specimens of this group, all in an immature state, presented no morphological differences. The ITS2 sequences of the specimens diverging in COI by 10.7% were identical. So these COI sequences could be considered as belonging to the same lineage as neither the morphological analysis nor ITS2 data allowed to differentiate them. In *G. riparius* E11, the sequences diverged by 10.6–13.3%. All the specimens of this group were in an immature form and presented no morphological differences. ITS2 sequences of these specimens were identical. These COI sequences were so grouped in one lineage.

**Table 1:** Lineages obtained in the present work. For each lineage are indicated the lineage number, the number of specimens and morphologically identified specimens, the maximum COI intralineage variability and the minimum COI interlineage variability. The ITS2 intralineage variability of two lineages is also mentioned. The new lineages for Switzerland are indicated with an asterisk following the lineage numbers. Taxonomic authors of species are cited in Table S2.

	Lineage No	Specimens	Morpho identified specimens	Maximum COI intralineage variability (%)	Minimum COI interlineage variability (%)	ITS2 intralineage variability (%)
**Tubificinae**						
Tubificinae sp (with hair setae)	T2	2	2	0.2	18.6	
*Aulodrilus pluriseta*	T4	8	6	7.2	20.9	
*Lophochaeta ignota*	T6	12	12	0.9	19.6	
*Potamothrix bavaricus*	T7	4	2	0.5	18.6	
*Psammoryctides barbatus*	T8	4	4	2	21.8	
*Tubifex tubifex*	T11	12	4	6.4	21.9	
*Tubifex tubifex*	T12	7	6	3.4	20.9	
*Limnodrilus hoffmeisteri*	T16	1	1	0.765	18.2	
*Limnodrilus hoffmeisteri*	T17	11	10	8.7	18.2	
*Limnodrilus hoffmeisteri*	T18	13	8	3.6	19.6	
*Limnodrilus hoffmeisteri*	T20	2	1	0	18.6	
*Limnodrilus claparedianus*	T22	6	6	5.9	18.4	
*Limnodrilus udekemianus*	T23	2	2	10.7	19.6	0
*Spirosperma ferox*	T24*	2	2	1.23	22.9	
*Embolocephalus velutinus*	T25*	10	10	6.453	24.7	
*Tubifex* sp	T26*	1	1	NC	21.5	
*Tubifex tubifex*	T27*	6	6	1.985	16.5	
*Potamothrix hammoniensis*	T28*	2	2	4.407	17.1	
*Potamothrix vejdovskyi*	T29*	1	1	NC	17.1	
*Potamothrix moldaviensis*	T30*	4	2	4.07	21.4	
*Potamothrix heuscheri*	T31*	4	4	0.613	13.5	
Tubificinae sp (with hair setae)	T32*	1	1	NC	23.1	
*Tasserkidrilus kessleri*	T33*	2	2	0	21	
**Naidinae**						
*Nais elinguis*	N4	4	4	1.5	15.7	
*Ophidonais serpentina*	N5	5	5	2.1	14.9	
*Vejdovskyella intermedia*	N7*	1	1	NC	17.9	
*Nais alpina*	N8*	2	2	1.8	9.0	
*Nais communis*	N9*	3	3	6.4	16.1	
*Nais communis*	N10*	1	1	NC	8.3	
*Nais christinae*	N11*	1	1	NC	10.5	
*Nais stolci* or *Nais pardalis*	N12*	2	2	0.9	10.3	
*Nais pseudobtusa*	N13*	1	1	NC	8.3	
*Uncinais uncinata*	N14*	1	1	NC	8.1	
*Chaetogaster diastrophus*	N15*	1	1	NC	15.5	
**Pristininae**						
*Pristina jenkinae*	P1*	1	1	NC	21.8	
**Enchytraeidae**						
*Marionina argentea*	E5*	1	1	NC	23.2	
*Achaeta* sp	E6*	1	1	NC	17.8	
*Achaeta* sp	E7*	3	3	0.3	21.2	
*Cernosvitoviella minor*	E8*	2	2	5.1	19.5	
*Globulidrilus riparius*	E9*	4	4	5.932	17.8	
*Globulidrilus riparius*	E10*	1	1	NC	15	
*Globulidrilus riparius*	E11*	19	16	13.3	15	0
*Fridericia* sp	E12*	1	1	NC	19.7	
*Lumbricillus* sp	E13*	1	1	NC	21.2	
*Fridericia* sp	E14*	2	2	0	18.8	
*Fridericia* sp	E15*	1	1	NC	19.7	
*Henlea perpusilla*	E16*	4	0	1.6	20.3	
*Enchytraeus buchholzi*	E17*	1	1	NC	17.8	
**Lumbriculidae**						
*Lumbriculus variegatus*	LL2	1	1	1.2	22.9	
*Stylodrilus heringianus*	LL3	30	12	0.8	22.9	
**Lumbricidae**						
*Eiseniella tetraedra*	LC3	1	1	8.7	10.6	
*Helodrilus oculatus*	LC4	1	0	2.4	21.4	
**Haplotaxidae**						
*Haplotaxis gordioides*	H1*	2	2	2.3	22.2	

**Notes.**

NC not calculated as the lineage contains only one sequence

A total of 53 lineages could be distinguished based on COI and ITS2 divergence and morphological analysis: 35 Naididae (23 Tubificinae, 11 Naidinae and 1 Pristininae), 13 Enchytraeidae, two Lumbriculidae, two Lumbricidae and one Haplotaxidae.

One hundred and twenty specimens were assigned to 19 already described lineages in Switzerland, while 96 corresponded to newly identified lineages for Switzerland. Thirty-four new lineages were added to the Swiss COI database. They included 23 species (*Potamothrix hammoniensis*, *Potamothrix vejdovskyi*, *Potamothrix moldaviensis*, *Potamothrix heuscheri*, *Spirosperma ferox, Embolocephalus velutinus, Tasserkidrilus kessleri*, *Vejdovskyella intermedia, Henlea perpusilla*, *Nais alpina*, *Haplotaxis gordioides*, *Nais christinae*, *Nais stolci/pardalis*, *Nais pseudobtusa*, *Uncinais uncinata*, *Chaetogaster diastrophus*, *Pristina jenkinae*, *Tubifex tubifex*, *Marionina argentea*, *Enchytraeus buchholzi, Nais communis* (two lineages), *Cernosvitoviella minor*, *Globulidrilus riparius* (three lineages), six lineages of Enchytraeidae (three *Fridericia* sp, two *Achaeta* sp and one *Lumbricillus* sp) and two lineages of Tubificinae (one *Tubifex* sp and one Tubificinae sp). COI sequences of the species *T. tubifex*, *M. argentea*, *E. buchholzi* and *N. communis* had already been reported (Vivien et al., 2015), so the new lineages of these species corresponded to cryptic species. A mature and identifiable specimen of the lineage T16, previously identified as “Tubificinae without hair setae” (Vivien et al., 2015), was found. This lineage corresponds to an additional cryptic species of *Limnodrilus hoffmeisteri*.

Out of the 34 newly found lineages in Switzerland, the sequences of *Potamothrix hammoniensis*, *Potamothrix vejdovskyi*, *Potamothrix moldaviensis*, *Spirosperma ferox*, *Henlea perpusilla*, *Cernosvitoviella* minor, *Nais alpina*, *Nais christinae*, *Nais communis* (two lineages), *Nais stolci/pardalis*, *Tubifex tubifex*, *Tasserkidrilus kessleri*, *Uncinais uncinata*, *Enchytraeus buchholzi*, *Vejdovskyella intermedia* and one of the three lineages of *Gobulidrilus riparius* (E9) were present in the Genbank database. However, in Genbank, the sequences of *Tasserkidrilus kessleri* and *Enchytraeus buchholzi* were identified at the family level, the sequences of *Nais pseudobtusa* and *Vejdovskyella intermedia* were identified at the genus level and the sequence of *Uncinais uncinata* was falsely identified (*Nais* sp). Sequences corresponding to *Potamothrix heuscheri* and *Chaetogaster diastrophus* were present in Genbank but differed from our sequences of these species (COI divergence >13%). Our specimens of *Henlea perpusilla* and *Cernosvitoviella minor* were immature and so were identified only based on Genbank data. The lineage N12 could correspond to *Nais stolci* or *Nais pardalis*. According to the Genbank database, they belonged to *N. stolci*, but our two specimens of this lineage did not present clearly the specific features of this species, namely strongly enlarged ventral crotchets from segment VI. So they seemed closer to *N. pardalis* than to *N. stolci*. The differentiation of these two species is difficult on the basis of chaetal morphology as *N. pardalis* can also possess strongly enlarged ventral chaetae (Timm, 2009). *N. pardalis* also differs from *N. stolci* in revealing abrupt stomach dilatation and presenting equally long teeth of posterior ventral crotchets. The stomach dilatation was not visible in our preparations and the character of the length of teeth of posterior crotchets could not be considered as the posterior parts of the specimens had been used for genetic analysis.

### Inventory of COI lineages in Switzerland

In total, 75 lineages have been found in Switzerland (Table S2): 33 Tubificinae, 15 Naidinae, one Pristininae, one Rhyacodrilinae, three Lumbricidae, 17 Enchytraeidae, four Lumbricidae and one Haplotaxidae. They corresponded to 44 morphospecies and 15 unidentified species and to cryptic species. The total number of cryptic species was 23: five in *Tubifex tubifex*, six in *Limnodrilus hoffmeisteri*, two in *Enchytraeus buchholzi*, two in *Eiseniella tetraedra*, two in *Marionina argentea*, three in *Globulidrilus riparius* and three in *Nais communis*. The sequence E3 (Enchytraeidae), identified in our previous work (Vivien et al., 2015) as *Lumbricillus rivalis* Levinsen 1884, was attributed by Klinth, Rota & Erséus (2017) to *Lumbricillus rutilus* as part of a systematics study of the genus *Lumbricillus*. Therefore, we assigned the species *L. rutilus* to the lineage E3.

### Phylogenetic analysis of all COI lineages found in Switzerland

**Figure 1:**
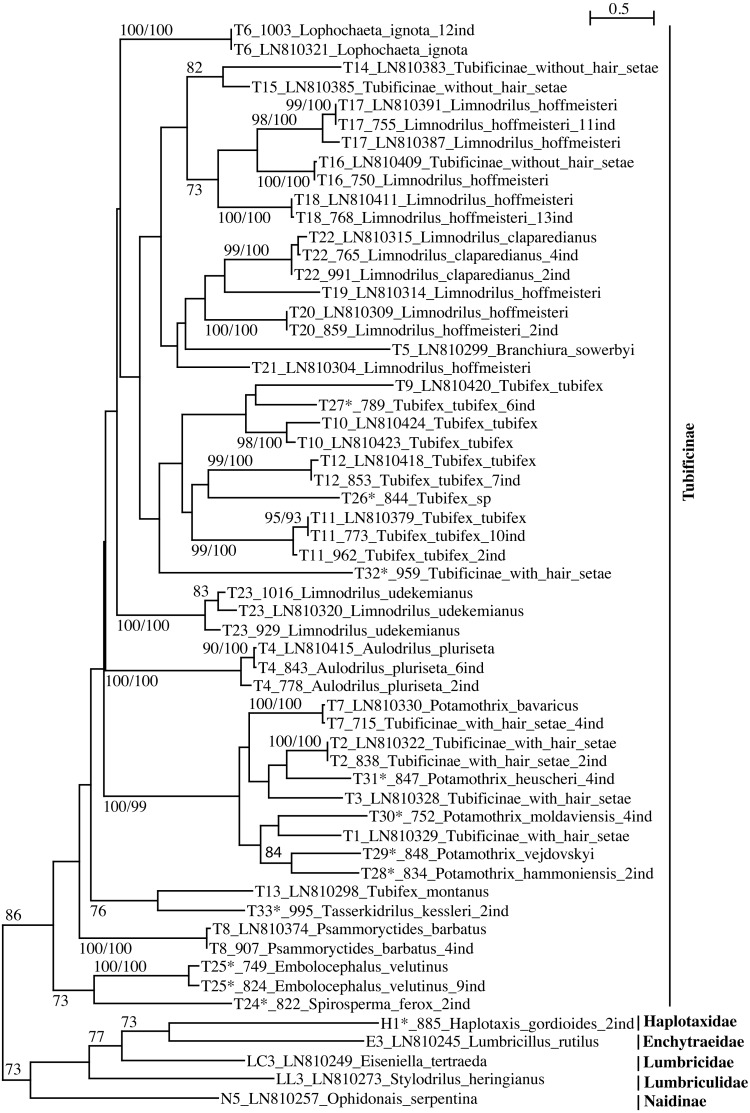
PhyML tree of Tubificinae subfamily (with some lineages of other families/subfamilies) based on COI sequences obtained in Switzerland. The tree shows the sequences separated by ≥5% of genetic divergence. The numbers above the internal nodes correspond to bootstrap values of ML and FastMe distance analyses; only those higher than 70% are indicated. For each lineage are indicated the number of lineage, followed by the number of isolate (for sequences obtained in the present work) or the accession number of Genbank (for sequences obtained anteriorly) and the name of the taxon. The number followed by “ind” corresponds to the number of sequences diverging by less than 5%. The lineages for which the lineage number is followed by an asterisk correspond to new lineages for Switzerland.

**Figure 2:**
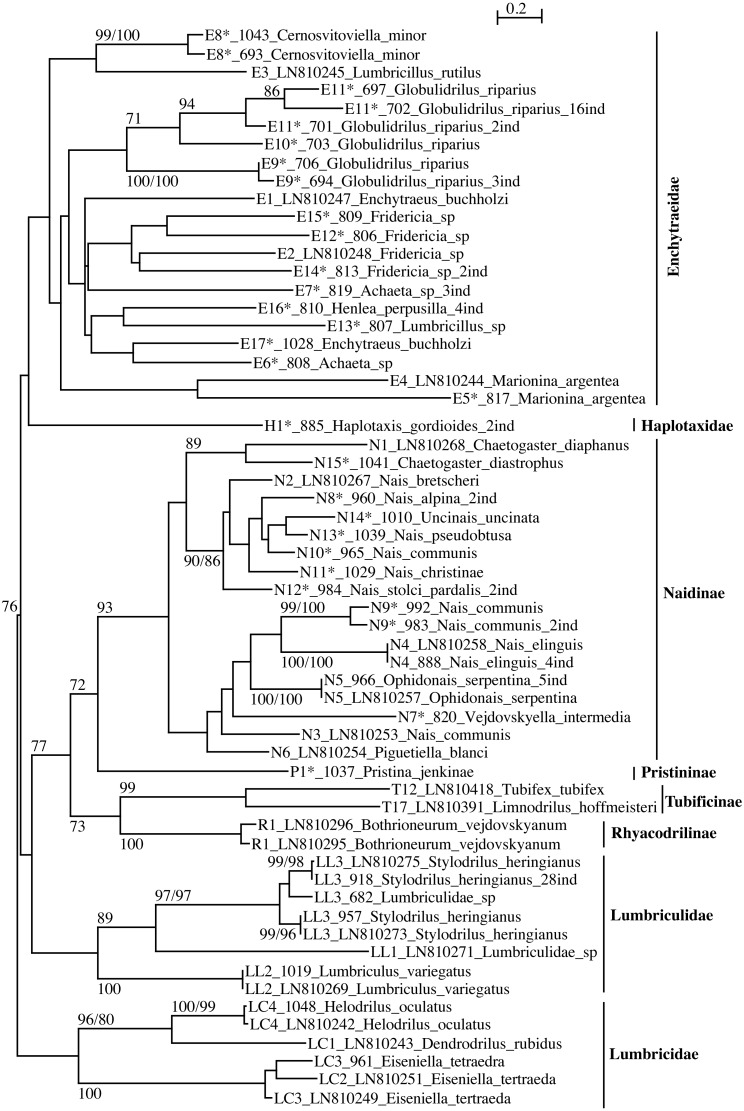
PhyML tree of all aquatic oligochaete families/subfamilies (only two lineages of Tubificinae represented) based on COI sequences obtained in Switzerland. The tree shows the sequences separated by ≥5% of genetic divergence. The numbers above the internal nodes correspond to bootstrap values of ML and FastMe distance analyses; only those higher than 70% are indicated. For each lineage are indicated the number of lineage, followed by the number of isolate (for sequences obtained in the present work) or the accession number of Genbank (for sequences obtained anteriorly) and the name of the taxon. The number followed by “ind” corresponds to the number of sequences diverging by less than 5%. The lineages for which the lineage number is followed by an asterisk correspond to new lineages for Switzerland.

The phylogenetic trees (Figs. 1 and 2, Figs. S1 and S2) represent all the lineages found so far in Switzerland. The two different substitution models (F84, T93) tested for FastMe 2.0 yielded congruent results.

The subfamily Tubificinae was monophyletic, sustained by a BV of 86% (Fig. 1). The Tubificinae with and without hair setae were globally well separated. The Tubificinae with hair setae correspond to the lineages T1–T13, T24–T29 and T31–T33 and the Tubificinae without hair setae to the lineages T14–T23 and T30. These two groups did not branch together, with the exception of the species *Branchiura sowerbyi* (Tubificinae with hair setae) and *Potamothrix moldaviensis* (Tubificinae without hair setae). This last species logically branched with the other *Potamothrix* species that all possess hair setae. The genera *Tubifex* and *Limnodrilus* appeared as polyphyletic. The tree combining sequences with a divergence <1% (Fig. S1) was almost identical to the one represented in Fig. 1. Differences occurred in the branching position of lineages T24 and T25 that clustered at the base of Tubificinae in Fig. 1 but branched with lineage T4 in Fig. S1. Lineages T13 and T33 branched at the base of Tubificinae next to lineage T8 in Fig. 1, while they branched with lineages T4, T24 and T25 in Fig. S1.

The families Enchytraeidae, Lumbriculidae and Lumbricidae were monophyletic but none of them showed any support (Fig. 2). Within the family Naididae, the subfamilies Naidinae, Tubificinae and Rhyacodrilinae were monophyletic and well supported (BV respectively 93%, 99% and 100%). The subfamily Pristininae branched at the base of the subfamily Naidinae but the support was moderate (BV 72%). The family Haplotaxidae branched at the base of the family Enchytraeidae but the branching was not supported. The tree combining sequences with a divergence <1% (Fig. S2) differed from the one represented in Fig. 2 in the branching position of Haplotaxidae, clustering within Enchytraeidae, but the branching was not supported.

## Discussion

This study confirms the advantages of molecular identification when compared to morphological identification of oligochaetes. The genetic analyses allowed to identify a number of species that remained undetected when identification was performed based on morphological traits. They corresponded either to cryptic species (two in *Nais communis*, three in *Globulidrilus riparius*, one in *Marionina argentea*, one in *Enchytraeus buchholzi* and one in *Tubifex tubifex*) or to morphologically distinct or cryptic species (one Tubificinae sp, three *Fridericia* sp and two *Achaeta* sp). The identification of most species within the genera *Fridericia* and *Achaeta* requires that the specimens are mature and can be dissected (Schmelz & Collado, 2010).

We found cryptic species among others in *Marionina argentea*, *Enchytraeus buchholzi*, *Globulidrilus riparius* and *Nais communis*. Two COI sequences of *E. buchholzi*, one COI sequence of *Globulidrilus riparius* and one COI sequence of *M. argentea*, different from our sequences, were present in Genbank. *M. argentea*, *E. buchholzi* and *G. riparius* had already been suspected to be species complexes on the basis of morphological observations (Schmelz & Collado, 2010; Rota, 2013). In *N. communis*, the lineages corresponding to our cryptic species had been mentioned by Envall, Gustavsson & Erséus (2012). These authors analysed several specimens of each of these lineages and could not find clear morphological criteria allowing to differentiate them. Our work reveals the possible existence of a cryptic diversity within the morphospecies *Potamothrix heuscheri* and *Chaetogaster diastrophus*, as our sequences and Genbank’s sequences corresponding to these species were different.

The distinction of cryptic species is important as they can show differences in their ecology and ecotoxicology. For example, Sturmbauer et al. (1999) showed that the cryptic species of *Tubifex tubifex* differed in their resistance to cadmium. Gustafsson, Price & Erséus (2009) demonstrated the existence of three cryptic species within *Lumbriculus variegatus* and strongly recommended to genetically identify this test organism before its use in ecotoxicological studies.

We observed that the 10% threshold of COI divergence was appropriate for distinguishing the majority of lineages. In the genera *Nais* and *Uncinais*, a threshold of 8% instead of 10% should be applied for species delimitation. Five lineages (two in *Limnodrilus udekemianus* and three in *Globulidrilus riparius* E11) presented a COI variation slightly superior to 10% (between 10.6 and 13.3%). As neither the morphological analysis nor the ITS2 data allowed to distinguish them, we grouped the sequences of *G. riparius* E11 in one lineage and the sequences of *L. udekemianus* in one lineage. A complementary morphological analysis and the sequencing of other markers would be necessary to determine with certainty if these sequences should be grouped in two or more lineages. It has been demonstrated that in aquatic oligochaetes COI evolved much faster than ITS region (Achurra & Erséus, 2013; Vivien et al., 2015). For example, Envall, Gustavsson & Erséus (2012) observed that within a clade of *Nais communis* (corresponding to our lineage N9), two phylotypes could be differentiated with COI and 16S data and not with ITS data.

Half of lineages reported in the present work had so far never been mentioned in the international databases. This is explained by the fact that most studies on molecular systematics of freshwater oligochaetes deal with the phylogenetic relationships within some subfamilies, genus or morphospecies (e.g., Bely & Wray, 2004; Timm et al., 2013; Liu et al., 2017). To our knowledge, none focuses on the genetic diversity of all commonly found species in rivers and lakes. All oligochaete lineages reported in Switzerland can probably be found in other countries, in particular in Europe, and so our database is of relevance not only nationally but also internationally.

The genetic diversity observed so far in Switzerland (75 lineages) is very high given the low number (about 400) of analysed specimens. In comparison, out of a total of 11,650 specimens analysed morphologically in the Geneva area between 2008 and 2013, 81 taxa were identified (Vivien & Lafont, 2015). This high genetic diversity could be explained by several factors. First, all sorted specimens have been assigned to molecular lineages, considerably increasing the identification ratio compared to the morphological studies. Secondly, during the sorting step, we selected specimens that were suspected to belong to new lineages. Thirdly, we investigated two different types of water bodies (rivers and lake) and sediments presenting different degrees of pollution, in order to maximize the species diversity. Nevertheless, this number of oligochaetes lineages does not seem to be artificially inflated given the important cryptic diversity revealed by this and other studies.

The perspectives are to continue enriching the COI database of Swiss aquatic oligochaetes, while determining the adequate threshold of COI divergence for the newly obtained oligochaete lineages. The development of a reliable and comprehensive database will be a determinant for a successful development of an oligochaete index based on the molecular identification of species.

##  Supplemental Information

10.7717/peerj.4122/supp-1 Table S1 Sampling and number of sequenced specimens per site

10.7717/peerj.4122/supp-2 Table S2 List of sequenced lineages (COI) so far in Switzerland and number of specimens per lineage sequenced in the present study and in our former study (Vivien et al., 2015) The new lineages for Switzerland are indicated with an asterisk following the lineage numbers.

10.7717/peerj.4122/supp-3 Figure S1 PhyML tree of Tubificinae subfamily (with some lineages of other families/subfamilies) based on COI sequences obtained in Switzerland The tree shows the sequences separated by ≥1% of genetic divergence. The numbers above the internal nodes correspond to bootstrap values of ML and FastMe distance analyses; only those higher than 70% are indicated. For each lineage are indicated the number of lineage, followed by the number of isolate (for sequences obtained in the present work) or the accession number of Genbank (for sequences obtained anteriorly) and the name of the taxon. The number followed by “ind” corresponds to the number of sequences diverging by less than 1%. The lineages for which the lineage number is followed by an asterisk correspond to new lineages for Switzerland.

10.7717/peerj.4122/supp-4 Figure S2 PhyML tree of all aquatic oligochaete families/subfamilies except Tubificinae based on COI sequences obtained in Switzerland The tree shows the sequences separated by ≥1% of genetic divergence. The numbers above the internal nodes correspond to bootstrap values of ML and FastMe distance analyses; only those higher than 70% are indicated. For each lineage are indicated the number of lineage, followed by the number of isolate (for sequences obtained in the present work) or the accession number of Genbank (for sequences obtained anteriorly) and the name of the taxon. The number followed by “ind” corresponds to the number of sequences diverging by less than 1%. The lineages for which the lineage number is followed by an asterisk correspond to new lineages for Switzerland.

10.7717/peerj.4122/supp-5 File S1 Raw data 1 Sequences presented in the phylogenetic trees (Figs. 1 and 2, Figs. S1 and S2).

10.7717/peerj.4122/supp-6 File S2 Raw data 2 All the sequences (COI and ITS2) obtained in the present work.

## Additional Information and Declarations

### DNA Deposition

The following information was supplied regarding the deposition of DNA sequences: The information about the deposition of COI and ITS2 sequences is indicated in the ‘Material and Methods’ section.

### Data Availability

The following information was supplied regarding data availability: All the DNA sequences have been deposited in the European Nucleotide Archive. In addition, they are provided the sequences as Files S1 and S2.
