# Supplementary material for: Cytochrome c oxidase barcodes for aquatic oligochaete identification: development of a Swiss reference database
Source: PeerJ. 2017 Dec 6;5:e4122. doi: 10.7717/peerj.4122 (PMC5723135; doi:10.7717/peerj.4122)
Supplement: Table S1 [file peerj-05-4122-s001.docx]

**Supplemental Table S1: Sampling and number of sequenced specimens per site**

| Site (canton) | Watercourse / lake | Sampling year | Coordinates X Y | Number of sequenced specimens |
| --- | --- | --- | --- | --- |
| La Plaine (GE) | Allondon | 2014 | 46.17930^o^N 6.00890^o^E | 8 |
| Claparède (GE) | Seymaz | 2013 | 46.18850^o^N 6.18506^o^E | 27 |
| site 2 (VD) | Venoge | 2015 | 46.54349^o^N 6.55083^o^E | 14 |
| Abbaye (VD) | Talent | 2015 | 46.59058^o^N 6.66702^o^E | 15 |
| site 1 (BE) | Cul des Prés | 2015 | 47.16085^o^N 6.87548^o^E | 7 |
| L'isle (VD) | Venoge | 2015 | 46.61821^o^N 6.40739^o^E | 14 |
| Source (VD) | Tinière | 2015 | 46.41076^o^N 6.96782^o^E | 3 |
| CIPEL_58 | Lake Geneva | 2015 | 46.45355^o^N 6.64467^o^E | 3 |
| CIPEL_49 | Lake Geneva | 2015 | 46.44409^o^N 6.57974^o^E | 4 |
| CIPEL_35 | Lake Geneva | 2015 | 46.44942 ^o^N 6.43948^o^E | 2 |
| CIPEL_30 | Lake Geneva | 2015 | 46.40329 ^o^N 6.38916^o^E | 1 |
| CIPEL_32 | Lake Geneva | 2015 | 46.46618 ^o^N 6.42316^o^E | 2 |
| CIPEL_38 | Lake Geneva | 2015 | 46.48822 ^o^N 6.47484^o^E | 6 |
| CIPEL_11 | Lake Geneva | 2015 | 46.32424^o^N 6.23064 ^o^E | 3 |
| CIPEL_15 | Lake Geneva | 2015 | 46.35990^o^N 6.25591 ^o^E | 6 |
| St Sulpice (VD) | Lake Geneva | 2015 | 46.51666^o^N 6.57772^o^E | 4 |
| site1 (GE) | Hermance | 2016 | 46.29618^o^N 6.24996^o^E | 27 |
| EPFL (VD) | Sorge | 2016 | 46.52266^o^N 6.57357^o^E | 7 |
| Versoix (GE) | Versoix | 2016 | 46.27482^o^N 6.17032^o^E | 11 |
| Hochdorf (LU) | Ron | 2016 | 47.17462^o^N 8.27986^o^E | 46 |
| Genève-Plage (GE) | Lake Geneva | 2017 | 46.20774^o^N 6.16126^o^E | 5 |
