# Supplementary material for: Cytochrome c oxidase barcodes for aquatic oligochaete identification: development of a Swiss reference database
Source: PeerJ. 2017 Dec 6;5:e4122. doi: 10.7717/peerj.4122 (PMC5723135; doi:10.7717/peerj.4122)
Supplement: Table S2 — The new lineages for Switzerland are indicated with an asterisk following the lineage numbers. [file peerj-05-4122-s002.docx]

**Supplemental Table S2 : List of sequenced lineages (COI) so far in Switzerland and number of specimens per lineage sequenced in the present study and in our former study (Vivien et al., 2015).** The new lineages for Switzerland are indicated with an asterisk following the lineage numbers.

|  |  | Lineage No | Sequences (present study) | Sequences (Vivien et al., 2015) |
| --- | --- | --- | --- | --- |
| **Naididae / Tubificinae** | Tubificinae with hair setae | T1 |  | 1 |
|  | Tubificinae with hair setae | T2 | 2 | 6 |
|  | Tubificinae with hair setae | T3 |  | 1 |
|  | *Aulodrilus pluriseta* (Piguet, 1906) | T4 | 8 | 1 |
|  | *Branchiura sowerbyi* Beddard, 1892 | T5 |  | 5 |
|  | *Lophochaeta ignota* Stolc, 1886 | T6 | 12 | 1 |
|  | *Potamothrix bavaricus* (Oschmann, 1913) | T7 | 4 | 44 |
|  | *Psammoryctides barbatus* (Grube, 1861) | T8 | 4 | 4 |
|  | *Tubifex tubifex* (Müller, 1774) | T9 |  | 4 |
|  | *Tubifex tubifex* (Müller, 1774) | T10 |  | 4 |
|  | *Tubifex tubifex* (Müller, 1774) | T11 | 12 | 5 |
|  | *Tubifex tubifex* (Müller, 1774) | T12 | 7 | 3 |
|  | *Tubifex montanus* Kowalewski, 1919 | T13 |  | 1 |
|  | *Tubificinae without hair setae* | T14 |  | 1 |
|  | *Tubificinae without hair setae* | T15 |  | 3 |
|  | *Limnodrilus hoffmeisteri* Claparede, 1862 | T16 | 1 | 1 |
|  | *Limnodrilus hoffmeisteri* Claparede, 1862 | T17 | 11 | 22 |
|  | *Limnodrilus hoffmeisteri* Claparede, 1862 | T18 | 13 | 5 |
|  | *Limnodrilus hoffmeisteri* Claparede, 1862 | T19 |  | 1 |
|  | *Limnodrilus hoffmeisteri* Claparede, 1862 | T20 | 2 | 5 |
|  | *Limnodrilus hoffmeisteri* Claparede, 1862 | T21 |  | 5 |
|  | *Limnodrilus claparedianus* Ratzel, 1868 | T22 | 6 | 5 |
|  | *Limnodrilus udekemianus* Claparede, 1862 | T23 | 2 | 1 |
|  | *Spirosperma ferox* Eisen, 1879 | T24 * | 2 |  |
|  | *Embolocephalus velutinus* (Grube, 1879) | T25 * | 10 |  |
|  | *Tubifex* sp | T26 * | 1 |  |
|  | *Tubifex tubifex* (Müller, 1774) | T27 * | 6 |  |
|  | *Potamothrix hammoniensis* (Michaelsen, 1901) | T28 * | 2 |  |
|  | *Potamothrix vejdovskyi* (Hrabe, 1941) | T29 * | 1 |  |
|  | *Potamothrix moldaviensis* Vejdovsky & Mrazek, 1903 | T30 * | 4 |  |
|  | *Potamothrix heuscheri* (Bretscher, 1900) | T31 * | 4 |  |
|  | Tubificinae with hair setae | T32 * | 1 |  |
|  | *Tasserkidrilus kessleri* (Hrabe, 1962) | T33 * | 2 |  |
| **Naididae / Naidinae** | *Chaetogaster diaphanus* (Gruithuisen, 1828) | N1 |  | 1 |
|  | *Nais bretscheri* Michaelsen, 1899 | N2 |  | 1 |
|  | *Nais communis* Piguet, 1906 | N3 |  | 1 |
|  | *Nais elinguis* Müller, 1774 | N4 | 4 | 9 |
|  | *Ophidonais serpentina* (Müller, 1774) | N5 | 5 | 1 |
|  | *Piguetiella blanci* (Piguet, 1906) | N6 |  | 3 |
|  | *Vejdovskyella intermedia* (Bretscher, 1896) | N7 * | 1 |  |
|  | *Nais alpina* Sperber, 1948 | N8 * | 2 |  |
|  | *Nais communis* Piguet, 1906 | N9 * | 3 |  |
|  | *Nais communis* Piguet, 1906 | N10 * | 1 |  |
|  | *Nais christinae* Kasprzak, 1973 | N11 * | 1 |  |
|  | *Nais stolci* Hrabe, 1981 or *Nais pardalis* Piguet, 1906 | N12 * | 2 |  |
|  | *Nais pseudobtusa* Piguet, 1906 | N13 * | 1 |  |
|  | *Uncinais uncinata* (Orsted, 1842) | N14 * | 1 |  |
|  | *Chaetogaster diastrophus* (Gruithuisen, 1828) | N15 * | 1 |  |
| **Naididae / Pristininae** | *Pristina jenkinae* | P1 * | 1 |  |
| **Naididae / Rhyacodrilinae** | *Bothrioneurum vejdovskyanum* Stolc, 1886 | R1 |  | 3 |
| **Enchytraeidae** | *Enchytraeus buchholzi* Vejdovsky, 1878 | E1 |  | 1 |
|  | *Fridericia* sp | E2 |  | 1 |
|  | *Lumbricillus rutilus* Welch, 1914 | E3 |  | 2 |
|  | *Marionina argentea* (Michaelsen, 1889) | E4 |  | 1 |
|  | *Marionina argentea* (Michaelsen, 1889) | E5 * | 1 |  |
|  | *Achaeta* sp | E6 * | 1 |  |
|  | *Achaeta* sp | E7 * | 3 |  |
|  | *Cernosvitoviella minor* Dozsa-Farkas, 1990 | E8 * | 2 |  |
|  | *Globulidrilus riparius* Bretscher, 1899 | E9 * | 4 |  |
|  | *Globulidrilus riparius* Bretscher, 1899 | E10 * | 1 |  |
|  | *Globulidrilus riparius* Bretscher, 1899 | E11 * | 19 |  |
|  | *Fridericia* sp | E12 * | 1 |  |
|  | *Lumbricillus* sp | E13 * | 1 |  |
|  | *Fridericia* sp | E14 * | 2 |  |
|  | *Fridericia* sp | E15 * | 1 |  |
|  | *Henlea perpusilla* Friend, 1911 | E16 * | 4 |  |
|  | *Enchytraeus bucholzi* Vejdovsky, 1878 | E17 * | 1 |  |
| **Lumbriculidae** | *Lumbriculidae* sp | LL1 |  | 2 |
|  | *Lumbriculus variegatus* (Muller, 1774) | LL2 | 1 | 2 |
|  | *Stylodrilus heringianus* Claparede, 1862 | LL3 | 30 | 22 |
| **Lumbricidae** | *Dendrodrilus rubidus* (Savigny, 1826) | LC1 |  | 1 |
|  | *Eiseniella tetraedra* (Savigny, 1826) | LC2 |  | 2 |
|  | *Eiseniella tetraedra* (Savigny, 1826) | LC3 | 1 | 2 |
|  | *Helodrilus oculatus* Hoffmeister, 1845 | LC4 | 1 | 1 |
| **Haplotaxidae** | *Haplotaxis gordioides* (Hartmann, 1821) | H1 * | 2 |  |
