# Supplementary figures and images for: Cytochrome c oxidase barcodes for aquatic oligochaete identification: development of a Swiss reference database
Source: PeerJ. 2017 Dec 6;5:e4122. doi: 10.7717/peerj.4122 (PMC5723135; doi:10.7717/peerj.4122)

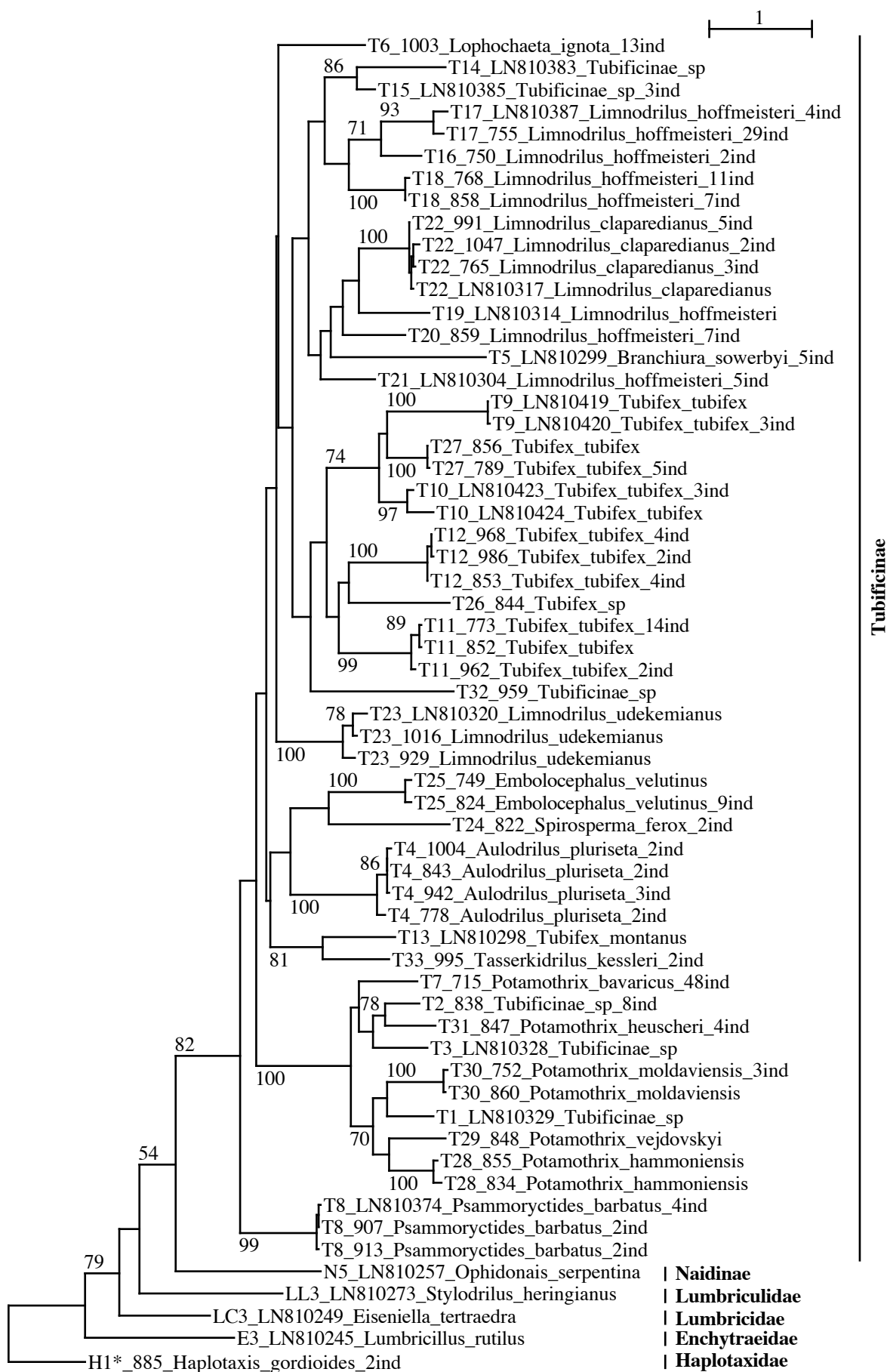

Supplement: Figure S1 — The tree shows the sequences separated by ≥1% of genetic divergence. The numbers above the internal nodes correspond to bootstrap values of ML and FastMe distance analyses; only those higher than 70% are indicated. For each lineage are indicated the number of lineage, followed by the number of isolate (for sequences obtained in the present work) or the accession number of Genbank (for sequences obtained anteriorly) and the name of the taxon. The number followed by “ind” corresponds to the number of sequences diverging by less than 1%. The lineages for which the lineage number is followed by an asterisk correspond to new lineages for Switzerland. [file peerj-05-4122-s003.pdf]

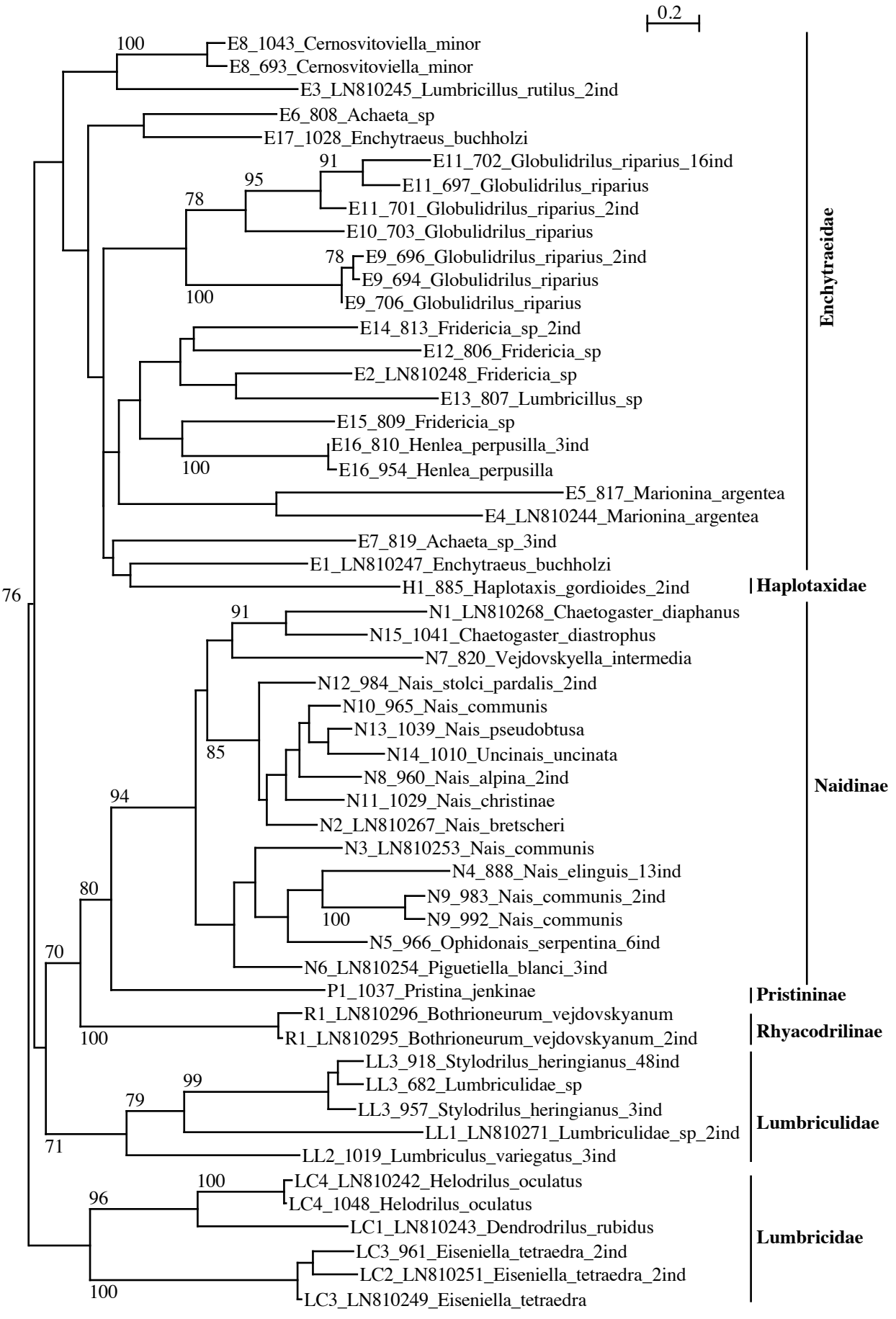

Supplement: Figure S2 — The tree shows the sequences separated by ≥1% of genetic divergence. The numbers above the internal nodes correspond to bootstrap values of ML and FastMe distance analyses; only those higher than 70% are indicated. For each lineage are indicated the number of lineage, followed by the number of isolate (for sequences obtained in the present work) or the accession number of Genbank (for sequences obtained anteriorly) and the name of the taxon. The number followed by “ind” corresponds to the number of sequences diverging by less than 1%. The lineages for which the lineage number is followed by an asterisk correspond to new lineages for Switzerland. [file peerj-05-4122-s004.pdf]
