# Supplementary material for: Cytochrome c oxidase barcodes for aquatic oligochaete identification: development of a Swiss reference database
Source: PeerJ. 2017 Dec 6;5:e4122. doi: 10.7717/peerj.4122 (PMC5723135; doi:10.7717/peerj.4122)
Supplement: File S1 — Sequences presented in the phylogenetic trees (Figs. 1 and 2, Figs. S1 and S2). [file peerj-05-4122-s005.docx]

**Supplemental File S1 : Raw data 1.** Sequences presented in the phylogenetic trees (Figure 1, Figure 2, Supplemental Fig. 1, Supplemental Fig. 2)

**Figure 1**

>T27*_789_Tubifex_tubifex_6ind

AACCCTTTACATACTATTTGGTATTTGAGCAGGTATAGTTGGCACCGGAACAAGTCTACT

AATTCGTCTAGAACTAGCTCAACCTGGCTCTTTCCTCGGCAGCGACCAATTATATAATAC

ATTAGTTACAGCACATGCATTCCTTATAATCTTCTTTATGGTAATACCTATTTACATTGG

GGGATTCGGAAATTGACTAGTCCCATTAATACTGGGGGCACCAGACATAGCATTCCCACG

ACTAAATAATCTTAGATTTTGACTTCTACCCCCCTCCCTAATTCTACTTGTATCATCTGC

TGCAGTGGAAAAAGGAGCTGGAACAGGGTGAACTGTCTACCCACCACTAGCCAGTAACTT

AGCACACTCTGGACCCTCAGTAGACTTAGCAATCTTCTCATTACACTTAGCTGGTGTAGC

CTCAATTCTAGGTGCCATTAACTTCATCACTACAATAATTAATATACGTTGAAAAGGTAT

ACGCCTAGAACGAATCCCATTATTTGTTTGATCAGTAATTATTACTGTAATTCTTTTACT

ACTTACACTTCCAGTACTAGCCGGTGCAATTACCATACTACTAACAGACCGAAATCTAAA

TACTTCATTCTTCGACCCTGCCGGTGGGGGAGACCCTGTTCTTTATCAACATCTATTC

>T24*_822_Spirosperma_ferox_2ind

--CCTTATATATAATTTTTGGTGTATGAGCCGGTATAGTAGGCACCGGAACTAGTCTATT

AATTCGCTTCGAGCTTGCTCAACCAGGGTCATTCCTAGGTAGAGACCAATTATACAATAC

ATTAGTAACCGCACATGCCTTCCTAATAATTTTCTTCTTAGTAATACCAGTATTTATTGG

GGGCTTCGGCAACTGACTAATCCCACTAATATTAGGTGCCCCTGATATAGCATTCCCACG

ACTGAATAATTTAAGATTCTGACTACTTCCTCCGTCCCTTATTCTCCTTGTATCCTCTGC

CGCAGTAGAAAAAGGTGCAGGTACCGGATGAACTGTTTATCCCCCTTTAGCTGGCAACCT

CGCCCACTCCGGCCCTTCAGTAGACTTAGCAATTTTTTCTCTCCATCTAGCCGGTGCATC

TTCTATTTTAGGTGCAATCAACTTTATTACAACTATGGTTAACATACGAAGAAAAGGTAT

ACGCCTGGAACGAGTTCCTCTATTTGTTTGAGCTGTTATTTTAACAGTAATCCTCCTATT

ACTTACACTTCCTGTATTAGCCGGTGCTATCACCATACTTCTAACAGATCGTAATCTCAA

CACGTCATTCTTTGACCCTGCTGGAGGGGGCGACCCTGTACTATACCAACACCTATTC

>T25*_824_Embolocephalus_velutinus_9ind

--CCCTATATATAGTATTTGGTGTATGAGCCGGTATAGTTGGAACCGGCACTAGATTACT

AATCCGCTTTGAGCTCGCGCAACCTGGATCATTCCTAGGTAGTGACCAACTCTACAATAC

GTTAGTTACTGCTCACGCCTTCCTTATAATCTTTTTCCTAGTAATACCTGTATTCATCGG

GGGATTCGGAAATTGACTTCTCCCATTAATATTAGGGGCACCAGATATAGCATTCCCTCG

CTTAAATAATCTAAGATTTTGGCTGCTGCCACCATCTTTAATTCTTCTAGTATCTTCGGC

GGCTGTAGAAAAGGGGGCCGGAACAGGGTGAACAGTGTACCCCCCACTATCAGGTAACTT

AGCCCATTCGGGACCATCAGTAGACTTAGCAATCTTTTCTCTTCATTTAGCTGGTGCATC

GTCCATTTTAGGGGCAATCAACTTCATTACTACAGTAATTAACATACGAAGTAAAGGTAT

GCGGCTGGAACGGGTACCACTATTTGTATGGGCTGTCCTTCTTACAGTAATTCTTCTCCT

GTTAACCCTTCCAGTACTCGCCGGAGCTATTACTATATTACTGACAGACCGCAATCTAAA

TACGTCGTTCTTTGATCCAGCTGGAGGTGGTGACCCAGTACTATACCAACACCTATTC

>T16_750_Limnodrilus_hoffmeisteri

CACTCTATACATGGTCTTCGGCCTCTGAGCAGGAATAGTGGGCACAGGAACCAGACTACT

AATTCGATTTGAATTAGCACAACCTGGCTCATTCCTTGGCAGAGATCAATTATATAACAC

CCTAGTTACAGCTCATGGATTTTTAATAATTTTCTTTATAGTTATACCTATTTTCATTGG

GGGCTTCGGTAACTGACTAGTCCCCCTAATACTTGGAGCTCCAGATATAGCCTTCCCACG

ACTCAATAATTTGAGATTTTGATTAATACCCCCATCTCTAATTCTATTAGTCTCCTCGGC

GGCTGTTGAGAAGGGTGCCGGAACAGGATGAACTGTATATCCACCTTTAGCTAGAAACCT

GGCTCATTCTGGGCCCTCTGTAGACCTGGCCATTTTTTCACTTCACTTAGCAGGGGCTGC

ATCAATCTTAGGTGCAATTAACTTCATTACCACAATAATTAATATACGATGAAAAGGAAT

ACGTTTAGAACGTATTCCCCTATTCGTGTGATCAGTTATTATTACGGTTATTTTACTCCT

TCTAACCTTACCAGTTCTAGCAGGGGCAATCACTATACTTCTAACAGACCGAAATCTAAA

CACGTCATTCTTCGACCCTGCGGGTGGTGGCGACCCGGTCCTATATCAACACTTATTC

>T30*_752_Potamothrix_moldaviensis_4ind

ACTTCTTTATATATTATATGGACTATGAACAGGTATGGTGGGAACAGGAACTAGAATTTT

AATTCGTCTGGAACTTGCTCAGCCTGGGTCATTCCTAGGTAGTGATCAGTTATATAACAC

CCTCGTAACTGCTCACGCCTTTCTGATAATCTTTTTTCTAGTAATACCTGTATATATTGG

AGCTTTCGGGAACTTCCTAATCCCACTAATACTAGGGGCTCCTGATATAGCTTTCCCACG

GTTAAACAATTTAAGATTTTGACTTATACCTTCATCTGTAATCCTCCTTGTGGCATCTGC

AGCTGTTGAAAAGGGAGCAGGAACAGGTTGAACAGTGTACCCTCCCCTAGCAAGAAACCT

TGCCCATTCCGGGCCTTCGGTAGACCTGGCCATTTTCTCTCTCCATCTAGCAGGAATTGC

TTCCATTTTAGGGGCAATTAACTTCATTACAACAATAATTAATATACGTTGAAAAGGGAT

ACGATTAGAGCGAATCCCCTTATTCGTTTGAGCGACAATCATCACAGTTATTTTACTACT

ATTAACTCTACCCGTATTAGCGGGCGCTATTACCATACTATTAACAGACCGAAATCTAAA

TACCTCATTCTTCGATCCTGCTGGGGGAGGAGACCCTGTCTTATATCAACACCTATTT

>T17_755_Limnodrilus_hoffmeisteri_11ind

CACCCTATACATAATCTTTGGCCTTTGAGCAGGAATAGTAGGCACAGGAACTAGACTTTT

AATTCGATTTGAGCTAGCACAACCCGGCTCATTTCTCGGTAGAGACCAATTATATAACAC

TCTAGTCACGGCTCACGGATTTTTAATAATTTTCTTTATAGTAATACCTATCTTTATTGG

GGGATTTGGAAATTGATTAGTGCCTTTAATACTTGGAGCACCTGATATGGCATTCCCACG

GCTTAATAACCTAAGATTCTGACTAATGCCCCCATCACTAATTCTACTAGTCTCATCAGC

TGCAGTTGAAAAAGGCGCAGGGACAGGGTGAACTGTATACCCCCCCTTAGCAAGAAATCT

AGCTCATTCCGGGCCTTCTGTAGATCTGGCAATTTTTTCACTACACTTAGCAGGAGCCGC

ATCAATCCTGGGAGCAATTAACTTCATCACAACAATAATCAATATACGATGAAAGGGAAT

ACGCTTAGAGCGTATTCCTTTATTTGTGTGATCCGTAATCATCACAGTTATTTTACTTCT

TCTTACCCTTCCAGTTCTTGCCGGAGCTATCACCATACTTTTAACAGATCGAAACCTAAA

CACCTCATTCTTTGATCCGGCAGGTGGAGGCGATCCAGTTTTATACCAACATCTATTT

>T22_765_Limnodrilus_claparedianus_4ind

CACTCTCTACATAGTTTTCGGCCTTTGAGCCGGAATAGTCGGTACTGGAACAAGCCTACT

AATTCGATTTGAGTTAGCTCAACCCGGATCATTCTTAGGCAGAGACCAGTTATATAACAC

TTTAGTGACAGCCCACGGATTCTTAATAATTTTCTTTATAGTAATGCCAATCTTTATTGG

TGGATTTGGAAATTGATTAGTTCCTTTAATACTTGGGGCACCAGATATAGCATTCCCACG

ATTAAATAATCTCAGCTTTTGACTAATGCCGCCATCATTAATTCTATTAGTGTCATCTGC

TGCTGTAGAAAAAGGTGCCGGTACAGGTTGAACTGTATATCCACCCCTGGCAAGAAATTT

AGCACATTCAGGACCATCTGTAGATTTAGCAATTTTCTCCCTTCATCTTGCTGGTGCAGC

TTCAATTCTAGGGGCAATTAACTTTATTACAACAATAATTAACATGCGATGAAAAGGAAT

ACGCCTCGAACGAATTCCATTATTCGTATGATCTGTAATTATTACTGTTATTCTACTACT

TCTAACCCTTCCAGTACTTGCGGGAGCTATCACGATGCTGTTAACAGACCGAAATCTAAA

TACATCGTTCTTCGACCCTGCGGGGGGGGGAGACCCTGTACTTTATCAACACTTATTC

>T18_768_Limnodrilus_hoffmeisteri_13ind

CACCTTATATATAATCTTCGGCCTATGAGCCGGAATAGTGGGCACAGGAACAAGCCTGCT

AATTCGATTCGAATTAGCACAACCTGGTTCATTCCTCGGAAGAGATCAACTCTATAATAC

CTTAGTGACAGCCCACGGTTTCCTTATAATCTTCTTCATGGTGATACCAATTTTTATCGG

TGGCTTCGGAAATTGACTAGTCCCCCTAATACTAGGAGCTCCTGACATGGCCTTTCCACG

ACTAAATAACCTAAGATTTTGACTAATACCTCCATCACTCATTCTATTAGTTTCATCAGC

CGCAGTCGAAAAGGGAGCGGGGACAGGGTGAACTGTATACCCCCCTCTAGCCAGAAACCT

AGCGCATTCTGGGCCATCTGTGGATCTAGCAATCTTCTCTCTTCACTTAGCCGGGGCTGC

ATCAATTCTAGGTGCTATTAACTTCATTACCACAATAATTAATATACGATGAAAAGGAAT

GCGCCTAGAGCGCATTCCTCTATTTGTATGATCAGTAATCATTACAGTTGTCCTCCTTCT

TCTTACATTACCGGTTTTAGCGGGGGCAATCACCATACTTTTAACAGACCGAAACTTAAA

CACCTCATTCTTCGATCCTGCGGGAGGGGGGGACCCGGTACTATATCAACACTTATTT

>T11_773_Tubifex_tubifex_10ind

AACTCTATATATCATTTTCGGGATTTGAGCTGGGATGGTCGGGACAGGAACTAGACTCTT

AATTCGTCTAGAACTGGCTCAACCTGGCTCATTCCTTGGAAGGGATCAGCTATACAACAC

CCTAGTTACAGCACATGCATTTCTGATAATTTTCTTCATGGTAATACCTATCTATATTGG

GGGTTTCGGAAATTGGTTAGTACCCTTAATGCTGGGAGCTCCTGACATAGCATTCCCCCG

ATTAAATAATTTAAGATTTTGGTTATTACCCCCTTCCTTAATCCTCCTTGTATCCTCCGC

GGCCGTAGAAAAGGGGGCTGGAACAGGGTGAACAGTATATCCGCCCCTAGCTAGAAATCT

GGCTCATTCTGGGCCTTCTGTAGACTTAGCTATTTTCTCTCTACATTTAGCTGGGGTGGC

ATCGATTCTAGGAGCTATTAACTTTATTACTACAATAATTAACATGCGATGAAAAGGGAT

ACGCCTTGAACGAATTCCCCTATTTGTATGAGCTGTAATTCTAACCGTAATCTTACTTTT

ACTAACATTACCAGTTTTAGCTGGTGCCATTACCATGCTACTAACAGATCGAAATCTAAA

CACATCCTTCTTTGACCCTGCTGGGGGTGGAGATCCTGTGCTCTATCAACACTTATTC

>T4_778_Aulodrilus_pluriseta_2ind

AACTCTATACTTCATTTTCGGAATCTGAGCCGGTATAGTTGGTACTGGTACCAGACTATT

AATTCGCTTAGAACTAGCACAACCTGGATCCTTCCTAGGAAGAGATCAACTATATAATAC

ATTAGTAACAGCCCACGCATTTCTAATAATCTTCTTCCTAGTTATACCTGTATTTATCGG

GGGATTCGGAAATTGATTAATCCCATTAATACTAGGTGCTCCCGATATAGCATTTCCACG

ACTCAATAACCTAAGATTTTGACTAATACCACCTTCACTAATTTTACTAGTCTCATCTGC

AGCTGTAGAAAAAGGTGCCGGAACAGGTTGAACAGTATACCCACCACTTGCTGGCAATCT

AGCCCACTCAGGACCTTCTGTAGACCTCGCAATCTTCTCTCTACACTTAGCAGGAGTAGC

ATCAATTTTAGGTGCTATTAACTTCATTACCACCATAATTAACATGCGATGAAAAGGGAT

ACGCCTAGAACGAATTCCTCTATTTGTATGAGCCGTAATTCTTACCGTTGTACTCCTTCT

ATTAACCCTACCAGTTCTTGCAGGAGCAATCACTATACTTTTAACTGACCGCAATCTAAA

CACCTCCTTCTTCGATCCGGCTGGAGGTGGTGACCCTGTTCTATACCAACATCTATTC

>T25*_749_Embolocephalus_velutinus

AACTCTATATATAGTATTTGGTGTGTGAGCCGGTATAGTTGGAACCGGCACTAGATTACT

AATCCGCTTTGAGCTTGCGCAACCTGGATCATTCCTAGGCAGTGACCAACTCTACAATAC

ATTAGTTACTGCTCACGCCTTCCTAATAATCTTTTTCCTAGTAATACCTGTATTCATCGG

AGGATTCGGAAATTGGCTTCTCCCGCTAATATTAGGGGCACCAGATATAGCATTCCCCCG

CTTAAATAACCTAAGATTTTGGCTGCTGCCACCATCTTTAATTCTTCTAGTGTCTTCGGC

GGCCGTAGAAAAAGGAGCCGGAACAGGATGAACAGTATATCCCCCGCTTTCCGGTAACTT

AGCCCATTCGGGGCCATCAGTAGACTTAGCAATCTTTTCCCTTCACTTAGCCGGTGCGTC

GTCCATTTTAGGGGCAATCAACTTCATTACTACAGTAATTAACATACGAAGTAAAGGCAT

ACGACTAGAGCGGGTACCACTATTTGTATGAGCTGTCCTTCTTACAGTAATTCTTCTCCT

GTTAACCCTCCCAGTACTCGCCGGAGCTATTACTATATTACTGACAGACCGCAATCTAAA

TACGTCATTCTTTGACCCGGCTGGTGGTGGCGACCCAGTACTATACCAACACCTATTC

>T28*_834_Potamothrix_hammoniensis_2ind

ACTCCTTTATATAATATATGGACTATGAACAGGTATGGTGGGAACTGGAACTAGAATACT

AATCCGAATAGAATTAGCTCAACCTGGATCCTTCCTTGGAAGGGACCAACTATATAATAC

TCTTGTAACTGCTCATGCCTTCTTAATAATTTTCTTCTTGGTTATACCGGTATATATTGG

TGCCTTCGGAAACTTTCTTGTACCTTTAATACTAGGTGCACCTGATATAGCTTTCCCCCG

AATAAATAATTTAAGATTTTGACTTATACCTTCATCTGTAATTCTATGCGTAGCATCTGC

AGCAGTTGAAAAAGGAGCAGGAACAGGTTGAACAGTATACCCGCCCTTAGCAAGAAATCT

TGCTCACTCGGGACCATCTGTAGATTTAGCAATCTTCTCTTTACATTTAGCCGGAATTTC

CTCTATTTTAGGGGCAATCAATTTTATTACTACAATAATTAACATACGTTGAAAAGGAAT

GCGATTAGAACGAATTCCTTTATTTGTGTGAGCGACAATCATTACTGTTCTTCTTCTATT

ATTAACTCTACCAGTTCTTGCTGGTGCTATTACCATACTATTAACAGATCGAAATCTAAA

TACCTCATTCTTTGATCCTGCTGGTGGTGGGGACCCTGTTCTATATCAACACCTATTC

>T2_838_Tubificinae_with_hair_setae_2ind

ACTTCTCTATATAATGTATGGCCTATGAACAGGTATGGTAGGAACAGGAACTAGAATTCT

AATTCGAATAGAACTTGCCCAACCAGGATCATTCCTTGGGAGAGACCAGCTATATAATAC

ACTCGTAACTGCCCATGCTTTCTTAATAATTTTCTTCCTAGTAATGCCCGTATACATTGG

TGCTTTTGGAAACTTCCTGGTACCATTAATACTTGGCGCACCAGATATAGCATTTCCACG

AATAAATAATCTCAGATTCTGATTAATACCTTCCTCTGTAATTTTATGTGTAGCATCTGC

TGCCGTTGAAAAAGGAGCCGGTACAGGTTGAACCGTTTACCCTCCATTAGCAAGAAATCT

TGCTCACTCTGGCCCATCTGTAGACTTAGCCATCTTCTCCCTTCACTTAGCAGGTATTTC

CTCTATTTTAAGAGCAATTAACTTCATTACTACAATAATCAATATACGATGAAAAGGAAT

ACGACTTGAACGAATTCCTTTATTTGTATGAGCTACAATCATTACAGTAATTCTACTACT

ATTAACACTTCCAGTTCTAGCTGGGGCTATCACCATATTACTAACAGACCGAAATCTAAA

TACCTCATTCTTTGACCCTGCAGGAGGGGGAGACCCTGTGCTTTATCAACATTTATTC

>T4_843_Aulodrilus_pluriseta_6ind

AACTCTATACTTCATTTTCGGAATCTGAGCCGGTATAGTTGGTACCGGTACCAGACTATT

AATTCGCTTAGAACTGGCACAACCTGGATCCTTCTTAGGAAGTGATCAACTATATAATAC

ATTAGTAACAGCTCACGCATTTCTAATAATCTTCTTCCTAGTTATACCTGTATTCATCGG

GGGATTCGGAAATTGATTAATCCCATTAATACTAGGTGCCCCTGATATAGCATTTCCACG

ACTAAATAACCTAAGATTTTGACTAATACCGCCCTCACTAATTCTACTAGTTTCATCCGC

AGCTGTAGAAAAAGGTGCAGGGACAGGTTGAACAGTATATCCACCACTTGCTGGCAATCT

AGCCCATTCAGGGCCTTCAGTAGATCTGGCAATCTTCTCTCTACACTTAGCAGGGGTAGC

ATCAATTCTAGGTGCTATTAACTTCATTACCACTATAATCAACATACGATGAAAAGGAAT

GCGCCTAGAACGAATTCCTCTATTTGTCTGAGCCGTAATTCTTACCGTTGTACTCCTTCT

ATTAACCTTACCAGTTCTGGCAGGGGCAATTACTATACTTCTAACTGACCGCAATCTAAA

CACCTCCTTCTTCGATCCAGCTGGGGGCGGTGACCCTGTATTATACCAACATCTATTC

>T26*_844_Tubifex_sp

AACACTATATATCATTTTTGGGGTATGAGCAGGAATGGTCGGAACAGGTACTAGATTATT

AATTCGACTAGAACTTGCCCAACCTGGCTCATTTCTTGGTAGCGATCAATTATTTAATAC

TCTGGTTACAGCCCATGCATTCCTGATAATCTTCTTCATAGTAATACCAATCTACATCGG

GGGATTCGGAAACTGATTAATTCCCTTAATACTGGGCGCCCCTGACATGGCCTTCCCTCG

TCTAAATAACTTAAGATTCTGACTTCTTCCACCATCTCTAATTCTATGTATTTCCTCGGC

AGCCGTTGAAAAAGGTGCTGGAACTGGGTGAACTGTATACCCCCCACTAGCAAGAAACCT

TGCCCACTCGGGACCATCTGTAGATCTTGCTATTTTCTCCTTACACTTAGCCGGGGTTGC

ATCAATTCTTGGTGCCATTAACTTTATTACAACTATAATCAACATACGATGAAAAGGAAT

ACGACTTGAACGAATCCCTCTATTTGTATGATCTGTAATTATTACAGTAGTTCTCCTACT

TCTTACATTACCAGTATTAGCGGGTGCTATCACTATACTACTAACAGACCGAAACCTAAA

TACCTCATTCTTCGATCCTGCCGGAGGAGGTGACCCAGTTCTTTACCAACATCTATTC

>T31*_847_Potamothrix_heuscheri_4ind

GCTTCTATATATAATATATGGCCTATGAACAGGTATAGTGGGAACCGGGACTAGAATTCT

AATTCGTATAGAACTAGCTCAACCTGGATCATTTCTAGGCAGGGACCAACTATATAATAC

ACTTGTAACTGCTCATGCCTTTTTAATAATTTTCTTCTTAGTAATACCAGTATATATTGG

GGCTTTTGGAAACTTTCTTGTTCCTTTAATACTAGGTGCACCAGATATAGCATTCCCTCG

AATAAACAATCTTAGATTCTGATTAATACCATCTTCTGTAATCTTATGTGTAGCTTCTGC

TGCCGTAGAAAAAGGGGCCGGTACAGGATGAACAGTATATCCTCCATTAGCAAGAAATCT

TGCTCACTCTGGACCATCTGTAGATCTTGCCATTTTCTCTCTTCACTTAGCGGGGATCTC

CTCTATTCTAGGCGCAATTAATTTCATTACTACGATAATTAATATACGATGAAAAGGAAT

GCGCCTAGAACGAATTCCTTTATTTGTATGAGCTACAATTATTACAGTAGTCTTACTACT

ACTAACACTTCCAGTTCTAGCTGGGGCTATTACTATGCTTCTAACAGACCGAAACCTGAA

TACCTCATTCTTTGATCCTGCAGGTGGAGGGGATCCTGTCCTGTACCAACATTTATTC

>T29*_848_Potamothrix_vejdovskyi

ATTCCTTTACATAATATATGGTTTATGAACAGGTATAGTAGGAACGGGAACTAGAATTTT

AATCCGGATAGAGCTAGCTCAACCGGGATCATTCCTTGGGAGTGACCAGCTATATAATAC

TCTTGTAACTGCTCACGCTTTTCTGATAATCTTCTTCTTAGTGATACCTGTTTATATCGG

CGCATTTGGGAACTTTCTTATACCTTTAATACTCGGGGCACCTGACATAGCATTTCCACG

ACTTAATAATTTAAGATTTTGATTGATACCTTCATCCGTAATTCTATGCGTAGCCTCTGC

AGCTGTAGAAAAGGGAGCAGGTACAGGCTGAACAGTATACCCTCCTCTAGCTAGGAACCT

TGCTCATTCTGGACCGTCTGTGGATTTAGCCATTTTCTCTTTACATTTAGCAGGTATCGC

CTCTATTTTAGGTGCAATCAATTTTATTACAACCATAATTAATATGCGGTGAATAGGAAT

ACGGTTAGAACGAATTCCTTTATTTGTATGAGCTACAATTATTACAGTTCTCCTTCTATT

ACTTACCCTACCTGTTCTGGCGGGGGCTATTACTATACTCTTAACAGATCGAAATCTAAA

TACCTCATTCTTTGACCCTGCGGGAGGGGGAGACCCTGTACTATATCAGCATCTATTC

>T12_853_Tubifex_tubifex_7ind

--------------TTTTCGGAATCTGAGCTGGTATGGTTGGAACTGGGACTAGGCTATT

AATTCGCCTTGAACTTGCCCAACCTGGGTCCTTTTTAGGAAGAGATCAATTATATAATAC

CCTGGTAACGGCTCACGCATTTTTAATAATCTTCTTCATAGTAATACCAATCTATATTGG

GGGCTTCGGCAACTGACTAGTACCATTAATACTAGGGGCTCCGGACATGGCCTTCCCACG

ATTAAACAACTTAAGATTCTGACTCCTACCCCCATCACTAATCCTTTTAGTATCCTCTGC

CGCCGTTGAAAAAGGCGCCGGAACTGGCTGAACTGTTTACCCCCCTCTAGCTAGAAATCT

AGCTCACTCTGGGCCCTCAGTAGATTTAGCAATCTTTTCTCTACACTTAGCTGGTGTTGC

ATCAATCCTAGGTGCAATCAACTTCATTACTACAATAATCAACATACGATGAAAAGGAAT

GCGACTAGAGCGTATTCCTTTATTTGTATGATCAGTAATTATCACTGTAATTCTACTCCT

ACTCACACTTCCAGTTTTAGCTGGAGCTATTACAATACTTCTAACAGACCGGAATCTAAA

TACATCATTTTTCGATCCTGCTGGAGGGGGTGACCCTGTACTATATCAACACCTATTC

>T20_859_Limnodrilus_hoffmeisteri_2ind

CACCCTTTACATAGTTTTTGGCTTATGAGCCGGAATAGTAGGTACCGGGACAAGATTACT

AATTCGCTTCGAACTGGCACAACCGGGATCATTCCTAGGCAGAGATCAGTTATATAATAC

ATTAGTAACTGCTCATGGTTTCTTAATAATTTTCTTCATGGTGATACCAATCTTTATCGG

TGGTTTTGGAAATTGATTAATTCCTTTAATACTTGGAGCCCCAGATATAGCGTTTCCTCG

ACTAAATAATCTTAGATTTTGATTAATGCCACCCTCTCTGATTCTACTTGTATCGTCAGC

AGCCGTGGAAAAAGGTGCAGGTACCGGATGAACTGTATACCCGCCGCTAGCTAGAAATCT

GGCACATTCAGGTCCATCGGTAGATCTAGCAATTTTTTCTTTACATTTAGCAGGTGCAGC

TTCAATTCTCGGGGCAATTAACTTCATTACTACAATAATTAACATACGGTGAAAAGGAAT

ACGTTTAGAACGTATTCCACTATTTGTGTGATCCGTAATTATTACTGTAGTCCTTCTTCT

ACTAACCCTACCTGTATTAGCAGGAGCCATTACAATATTATTAACAGATCGAAATCTAAA

TACATCATTCTTTGACCCTGCAGGAGGTGGAGACCCTGTACTCTACCAACATCTATTT

>T8_907_Psammoryctides_barbatus_4ind

TACTTTATATTTAATTTTTGGCTTATGAGCTGGGATGGTCGGAACTGGAACTAGTTTATT

AATTCGTATAGAACTCGCTCAACCGGGATCATTCCTTGGAAGAGACCAGTTATATAACAC

ACTAGTAACAGCACATGCTTTCCTAATAATCTTCTTCCTAGTAATACCCGTATTTATTGG

TGGGTTTGGTAATTGACTTCTACCTTTAATACTTGGGGCACCAGATATAGCTTTTCCACG

CCTAAACAATTTAAGATTTTGATTATTACCTCCATCCCTTATTCTTCTAGTATCATCTGC

TGCTGTTGAAAAAGGTGCTGGAACAGGATGAACAGTATATCCACCACTAGCTGGTAACCT

GGCACACTCTGGCCCTTCTGTAGACCTGGCTATTTTCTCTCTTCATTTAGCTGGTGCCGC

ATCTATTTTAGGAGCCATTAATTTTATTACTACTATAATTAATATACGGTGAAAAGGTAT

ACGATTAGAGCGAATTCCTCTATTTGTATGAGCTGTAATCATTACAGTAATTCTTCTTTT

ATTAACCCTTCCAGTATTAGCAGGTGCAATTACTATACTACTAACGGATCGAAATCTAAA

TACATCATTCTTTGATCCTGCTGGTGGTGGGGATCCTGTTCTTTATCAACACTTATTC

>T7_715_Tubificinae_with_hair_setae_4ind

ACTCCTTTATATAATGTATGGCCTATGAACAGGCATGGTAGGAACTGGAACTAGACTACT

AATTCGAATGGAACTTGCTCAACCAGGATCATTCCTTGGCAGAGATCAACTCTATAACAC

TCTCGTAACCGCACACGCATTTCTTATAATCTTTTTCCTTGTTATACCTGTATATATTGG

TGCCTTCGGAAACTTCCTCGTCCCATTAATACTTGGCGCCCCTGATATAGCATTTCCACG

GCTAAATAACTTAAGATTTTGACTAATACCCTCATCTGTAATTCTATGCGTGTCATCAGC

TGCTGTTGAAAAAGGAGCCGGTACTGGTTGAACAGTATACCCCCCATTAGCAAGAAATCT

TGCTCATTCAGGCCCATCTGTCGACTTAGCTATTTTCTCTCTTCACTTAGCAGGTATCTC

CTCTATTCTAGGGGCGATTAACTTTATTACCACTATAATTAATATGCGTTGAAAAGGAAT

GCGCCTAGAACGAATTCCATTATTTGTATGAGCTACTATTATTACAGTAGTATTACTCCT

ACTAACTCTTCCTGTCCTGGCCGGAGCTATTACTATACTTCTAACCG-------------

----------------------------------------------------------

>T13_LN810298_Tubifex_montanus

GACACTATATATAATCTTCGGATTCTGAGCCGGAATAGTAGGAACCGGAACAAGTTTACT

AATTCGATTTGAACTAGCTCAACCAGGATCTTTCCTGGGCAGGGATCAACTATATAATAC

TTTAGTTACGGCTCATGCCTTCTTAATAATTTTCTTCCTTGTAATGCCCGTCTTTATTGG

GGGGTTTGGCAATTGATTAGTTCCATTAATGCTAGGGGCACCTGACATAGCCTTCCCTCG

TCTAAATAATCTAAGATTTTGATTACTTCCACCATCTTTAATTCTTCTAGTGTCATCAGC

CGCTGTAGAAAAAGGAGCAGGAACTGGATGAACTGTCTACCCACCATTAGCCGGGAACTT

AGCTCATTCAGGCCCTTCAGTAGATTTAGCCATTTTTTCACTTCATTTAGCAGGAATTGC

ATCAATTCTAGGAGCTATTAACTTCATTACTACAATAATCAACATACGATGAAAAGGAAT

GCGTTTAGAACGAATCCCATTATTTGTATGAGCTGTTATTTTAACAGTAATTCTCCTCCT

ACTAACTCTTCCTGTTCTAGCAGGAGCAATTACTATATTACTAACAGATCGAAATCTAAA

TACATCATTTTTTGATCCTGCAGGTGGGGGGGACCCCGTTCTCTACCAACATTTATTC

>T5_LN810299_Branchiura_sowerbyi

AACACTATACATAGTATTCGGCCTATGAGCCGGAATAGTGGGAACAGGAACCAGAATTCT

AATTCGAACTGAACTAACTCAACCAGGATCCTTTCTTGGAAGAGATCAACTATATAACAC

CCTAGTGACAGCTCACGGATTCTTAATAATTTTCTTTATAGTAATACCAATCTTTATTGG

GGGTTTTGGGAACTGATTATTACCTTTAATACTCGGGGCACCAGATATAGCATTTCCCCG

AATAAATAATCTTAGATTCTGATTAATACCACCATCGTTAATTCTTCTTGTATCATCAGC

CGCAGTAGAAAAAGGTGCCGGTACAGGCTGAACAGTATATCCACCACTTTCCAGAAATCT

AGCCCATTCTGGACCATCAGTAGATTTAGCCATTTTTTCACTCCACTTAGCTGGTGCCTC

ATCAATTTTAGGGTCAATTAATTTTATCACCACAATAATTAACATACGATCAAAAGGAAT

ACGACTAGAACGAATCCCCTTATTTGTATGAGCTGTAATTATTACAACCATTCTTCTAGT

ATTAACCCTTCCAGTTCTAGCTGGTGCTATTACAATACTACTTACAGACCGAAATCTTAA

TACGTCATTTTTTGATCCTGCTGGAGGGGGGGATCCTGTTTTATATCAACACCTATTT

>T21_LN810304_Limnodrilus_hoffmeisteri

TACTCTATATATAATCTTTGGCTTATGAGCAGGAATAGTTGGAACCGGAACAAGCCTATT

AATTCGATTTGAGTTAGCTCAACCAGGCTCTTTCCTAGGCAGAGATCAATTATATAACAC

CTTAGTAACAGCACATGGGTTCTTAATAATTTTCTTCATAGTAATACCAATCTTCATTGG

GGGATTCGGAAACTGATTAATCCCATTAATATTAGGAGCTCCAGATATAGCATTCCCTCG

ACTAAATAATCTTAGATTCTGACTAATGCCCCCATCCTTAATTCTACTTGTGTCATCAGC

AGCTGTAGAAAAAGGAGCGGGTACAGGGTGAACCGTATACCCTCCACTAGCCAGTAATCT

GGCCCACTCCGGGCCATCTGTAGATTTAGCCATTTTCTCCTTACATTTAGCAGGTGCAGC

CTCAATTCTCGGGGCCATCAACTTCATTACCACAATAATTAATATGCGGTGAAAAGGAAT

ACGATTAGAGCGTATTCCCTTATTTGTTTGATCAGTAATTATTACTGTTATCCTTCTACT

TCTAACTCTTCCAGTACTAGCAGGAGCCATTACTATGCTCCTAACGGACCGAAATCTAAA

TACATCATTCTTTGATCCTGCTGGAGGGGGAGATCCTGTTCTATATCAACACTTATTC

>T20_LN810309_Limnodrilus_hoffmeisteri

CACCCTTTACATAGTTTTTGGCTTATGAGCCGGAATAGTAGGTACCGGGACAAGATTACT

AATTCGCTTCGAACTGGCACAACCGGGATCATTCCTAGGCAGAGATCAGTTATATAATAC

ATTAGTAACTGCTCATGGTTTCTTAATAATTTTCTTCATGGTGATACCAATCTTTATCGG

TGGTTTTGGAAATTGATTAATTCCTTTAATACTTGGAGCCCCAGATATAGCGTTTCCTCG

ACTAAATAATCTTAGATTTTGATTAATGCCACCCTCTCTGATTCTACTTGTATCGTCAGC

AGCCGTGGAAAAAGGTGCAGGTACCGGATGAACTGTATACCCGCCGCTAGCTAGAAATCT

GGCACATTCAGGTCCATCGGTAGATCTAGCAATTTTTTCTTTACATTTAGCAGGTGCAGC

TTCAATTCTCGGGGCAATTAACTTCATTACTACAATAATTAACATACGGTGAAAAGGAAT

ACGTTTAGAACGTATTCCACTATTTGTGTGATCCGTAATTATTACTGTAGTCCTTCTTCT

ACTAACCCTACCTGTATTAGCAGGAGCCATTACAATATTATTAACAGATCGAAATCTAAA

TACATCATTCTTTGACCCTGCAGGAGGTGGAGACCCTGTACTCTACCAACATCTATTT

>T19_LN810314_Limnodrilus_hoffmeisteri

CACTCTATATATAATTTTTGGTCTCTGAGCTGGAATAGTGGGAACTGGAACAAGACTACT

AATTCGATTTGAATTAGCTCAACCCGGATCATTCTTAGGTAGTGATCAATTATACAATAC

ACTAGTAACAGCCCACGGCTTCTTAATAATTTTTTTTATGGTGATACCAATCTTTATTGG

GGGATTCGGAAATTGACTAATTCCATTAATGCTGGGAGCCCCAGATATGGCCTTTCCTCG

TCTCAATAATCTTAGATTTTGGCTGATACCACCATCATTAATTTTACTAGTATCCTCTGC

TGCAGTAGAAAAAGGCGCAGGTACCGGATGAACTGTATATCCTCCATTAGCTAGAAATCT

AGCACATTCAGGGCCATCTGTAGATCTAGCAATTTTCTCACTACATTTAGCCGGAGTAGC

TTCAATTCTAGGGGCTATCAACTTTATCACAACAATAATTAATATACGATGAAAAGGAAT

GCGATTAGAACGAATCCCATTATTTGTATGATCTGTAATTATTACTGTCATTTTACTCCT

TTTAACCCTACCAGTATTAGCCGGAGCTATTACAATATTACTGACTGACCGAAATCTAAA

TACATCATTCTTCGACCCAGCAGGGGGGGGTGATCCTGTACTTTATCAACACTTATTT

>T22_LN810315_Limnodrilus_claparedianus

CACTCTCTACATAGTTTTCGGCCTTTGAGCCGGAATAGTTGGTACTGGGACAAGCCTACT

AATTCGATTTGAACTAGCTCAACCCGGATCATTTTTAGGCAGGGACCAGTTATATAACAC

TTTAGTGACAGCCCACGGATTCTTAATAATTTTCTTTATAGTGATGCCCATCTTTATTGG

TGGGTTCGGAAATTGATTAATTCCCTTAATACTTGGGGCACCAGATATAGCATTCCCACG

ATTAAATAATCTCAGCTTTTGACTAATGCCACCATCATTAATTCTATTAGTATCATCTGC

TGCTGTAGAAAAAGGTGCCGGCACAGGTTGAACTGTATATCCACCCCTGGCAAGAAATTT

AGCACATTCAGGGCCATCTGTAGATTTAGCAATTTTCTCCCTTCATCTTGCTGGTGCAGC

TTCAATTCTAGGGGCAATTAACTTTATTACAACAATAATTAACATACGATGAAAAGGAAT

GCGCCTCGAACGAATTCCATTATTTGTATGATCTGTAATTATTACTGTTATTCTACTACT

TCTAACCCTTCCAGTACTTGCGGGAGCTATCACGATGCTATTAACAGACCGAAATCTAAA

TACATCATTCTTCGACCCTGCGGGGGGAGGAGACCCTGTACTTTATCAGCACTTATTC

>T23_LN810320_Limnodrilus_udekemianus

----------------------CTGTGAGCCGGTATAGTTGGCACAGGTACCAGACTATT

AATTCGATTTGAACTAGCACAACCCGGATCATTCCTAGGCAGAGACCAACTCTATAATAC

ACTAGTAACAGCACATGGATTTCTAATAATTTTCTTCCTTGTAATACCAGTATTTATTGG

GGGATTCGGTAATTGACTAGTACCATTAATACTTGGGGCCCCTGATATGGCATTTCCACG

ACTAAACAACCTAAGATTTTGATTAATACCTCCATCACTTATTCTACTTGTATCCTCAGC

AGCAGTAGAAAAAGGGGCCGGAACAGGATGAACTGTATATCCGCCATTAGCAGGAAATCT

TGCCCATTCCGGGCCTTCCGTGGACCTCGCTATTTTCTCTCTCCATTTAGCTGGAATTGC

CTCAATTCTAGGAGCTATTAATTTTATCACCACTATAATTAACATACGATGAAAAGGAAT

GCGACTCGAACGAATTCCTCTATTTGTGTGATCCGTAATTATCACTGTAGTCTTACTTCT

ATTAACTCTTCCTGTATTAGCAGGGGCGATTACAATACTCCTAACAGATCGAAATCTAAA

TACATCATTTTTTGATCCTGCCGGAGGAGGGGATCCTGTCCTATATCAACACTTATTT

>T6_LN810321_Lophochaeta_ignota

AACTCTCTATATAATCTTCGGCCTATGAGCGGGAATAGTCGGCACCGGTACTAGACTACT

AATTCGACTAGAATTAGCTCAACCCGGATCATTCCTTGGTAGTGATCAACTATATAATAC

ACTTGTTACTGCCCATGGATTCCTTATAATTTTCTTCATGGTAATACCAGTATTTATTGG

GGGATTTGGTAACTGACTAGTCCCATTAATACTTGGTGCCCCAGATATAGCCTTCCCACG

AATAAATAATTTAAGATTTTGATTAATACCCCCCTCTTTAATCCTTCTCGTATCTTCAGC

AGCCGTTGAAAAAGGTGCTGGAACAGGCTGAACAGTATATCCTCCATTAGCCGGGAATTT

AGCTCACTCAGGACCATCCGTAGACCTAGCTATTTTCTCACTGCATTTAGCCGGAGCTGC

CTCAATTTTAGGTGCAATTAACTTTATTACGACTATAATTAACATGCGATGAAAAGGAAT

ACGCCTAGAACGCATTCCTCTATTTGTTTGATCCGTAATTATCACAGTAGTCCTACTTTT

ATTAACACTACCAGTTCTAGCAGGGGCCATTACAATATTACTTACAGACCGAAACTTAAA

TACCTCATTCTTTGACCCTGCTGGAGGTGGAGACCCTGTTTTATATCAACATCTATTC

>T2_LN810322_Tubificinae_with_hair_setae

ACTTCTCTATATAATGTATGGCCTATGAACAGGTATGGTAGGAACAGGAACTAGAATTCT

AATTCGAATAGAACTTGCCCAACCAGGATCATTCCTTGGGAGAGACCAGCTATATAATAC

ACTCGTAACTGCCCATGCTTTCTTAATAATTTTCTTCCTAGTAATGCCCGTATACATTGG

TGCTTTTGGAAACTTCCTGGTACCATTAATACTTGGCGCACCAGATATAGCATTTCCACG

AATAAATAATCTCAGATTCTGATTAATACCTTCCTCTGTAATTTTATGTGTAGCATCTGC

TGCCGTTGAAAAAGGAGCCGGTACAGGTTGAACCGTTTACCCTCCATTAGCAAGAAATCT

TGCTCACTCTGGCCCATCTGTAGACTTAGCCATCTTCTCCCTTCACTTAGCAGGTATTTC

CTCTATTTTAGGAGCAATTAACTTCATTACTACAATAATCAATATACGATGAAAAGGAAT

ACGACTTGAACGAATTCCTTTATTTGTATGAGCTACAATCATTACAGTAATTCTACTACT

ATTAACACTTCCAGTTCTAGCTGGGGCTATCACCATATTACTAACAGACCGAAATCTAAA

TACCTCATTCTTTGACCCTGCAGGAGGGGGAGACCCTGTGCTTTATCAACATTTATTC

>T3_LN810328_Tubificinae_with_hair_setae

ACTCCTTTACATAATATATGGCCTATGAACCGGAATGGTAGGAACTGGGACTAGATTACT

AATTCGAATAGAACTAGCTCAACCGGGATCATTCCTGGGTAGAGATCAATTATATAATAC

TCTCGTAACTGCCCATGCATTCCTAATAATTTTCTTCCTTGTAATACCAGTATATATCGG

TGCTTTTGGAAACTTCCTAGTTCCATTAATGCTCGGCGCCCCAGATATAGCTTTCCCCCG

AATAAATAATCTAAGATTCTGATTAATACCTTCTTCTGTGATCTTATGCGTAGCATCTGC

TGCTGTGGAAAAAGGGGCTGGAACAGGCTGAACAGTATATCCCCCATTAGCAAGAAATCT

TGCTCATTCTGGGCCCTCTGTGGACCTAGCTATTTTTTCCCTTCATTTAGCGGGAATTTC

ATCTATTTTAGGCGCAATTAACTTCATTACTACTATAATCAATATACGGTGAAAAGGGAT

ACGATTAGAACGAATTCCCCTGTTTGTGTGAAGTGTGATCATCACAGTAGTACTACTACT

ACTAACTCTTCCAGTCTTAGCTGGGGCTATTACTATACTTTTAACTGACCGTAATCTAAA

TACATCATTCTTCGACCCTGCCGGGGGAGGGGACCCTGTATTATATCAACATTTATTC

>T1_LN810329_Tubificinae_with_hair_setae

ACTCCTTTATATAATATATGGGCTATGAACAGGTATAGTTGGCACAGGGACTAGAATTTT

AATTCGAATAGAACTTGCTCAACCGGGATCATTTTTAGGGAGAGATCAACTATATAACAC

CCTTGTAACTGCCCACGCCTTTCTAATAATTTTCTTTCTCGTAATACCAGTATATATTGG

AGCCTTCGGAAACTTCCTTGTCCCATTAATACTAGGAGCTCCTGATATAGCATTTCCGCG

ATTAAATAATTTAAGATTTTGACTAATACCTTCATCAGTAATCTTATGTGTAGCTTCCGC

CGCAGTTGAAAAGGGGGCGGGCACAGGCTGAACAGTATATCCTCCATTAGCAAGAAACCT

TGCTCATTCCGGACCCTCAGTCGATTTAGCTATTTTCTCGCTTCACTTAGCAGGAATCGC

CTCCATTCTAGGTGCAATCAACTTTATCACAACTATGATCAATATACGATGAAAAGGTAT

ACGACTAGAACGAATTCCTTTATTTGTATGAGCTACAATTATTACAGTTCTTCTCCTTTT

ATTAACTCTTCCTGTCTTAGCCGGTGCTATTACCATACTACTAACAGACCGAAATCTAAA

TACCTCATTCTTTGATCCTGCTGGTGGTGGAGACCCAGTCTTATACCAACATCTATTC

>T7_LN810330_Potamothrix_bavaricus

ACTCCTTTATATAATGTATGGCCTATGAACAGGCATGGTAGGAACTGGAACTAGACTACT

AATTCGAATGGAACTTGCTCAACCAGGATCATTCCTTGGCAGAGATCAGCTCTATAACAC

TCTCGTAACCGCACACGCATTTCTTATAATCTTTTTCCTTGTTATACCTGTATATATTGG

TGCCTTCGGAAACTTCCTCGTCCCATTAATACTCGGCGCCCCTGATATAGCATTTCCACG

GCTAAATAACTTAAGATTTTGACTAATACCCTCATCTGTAATTCTATGCGTGTCATCAGC

TGCTGTTGAAAAAGGAGCCGGTACTGGTTGAACAGTATACCCCCCATTAGCAAGAAATCT

TGCTCATTCAGGCCCATCTGTCGACTTAGCTATTTTCTCTCTTCACTTAGCAGGTATCTC

CTCTATTCTAGGGGCGATTAACTTTATTACCACTATAATTAATATGCGTTGAAAAGGGAT

GCGCCTAGAACGAATTCCATTATTTGTATGAGCTACTATTATTACAGTAGTATTACTCCT

ACTAACTCTTCCTGTCCTGGCCGGAGCTATTACTATACTTCTAACCGACCGTAATCTAAA

TACCTCATTCTTCGACCCTGCTGGAGGGGGTGACCCTGTCCTATACCAACACCTGTTC

>T8_LN810374_Psammoryctides_barbatus

TACTTTATATTTAATTTTTGGCTTATGAGCTGGGATGATCGGAACTGGAACTAGTTTATT

AATTCGTATAGAACTCGCTCAACCGGGATCATTCCTTGGAAGAGACCAGTTATATAACAC

ACTAGTAACAGCACATGCTTTCCTAATAATCTTCTTCCTAGTAATACCCGTATTTATTGG

TGGGTTTGGTAATTGACTTCTACCCTTAATACTTGGGGCACCAGATATAGCTTTCCCACG

CCTAAACAATTTAAGATTTTGATTATTACCTCCATCCCTTATTCTTCTAGTATCATCTGC

TGCTGTTGAAAAAGGTGCTGGAACAGGATGAACAGTATATCCACCACTAGCTGGTAACCT

CGCACACTCTGGCCCTTCTGTAGACCTGGCTATTTTCTCTCTTCACTTAGCTGGTGCCGC

ATCTATTTTAGGAGCCATTAATTTTATTACTACTATAATTAATATACGGTGAAAAGGTAT

ACGATTAGAGCGAATTCCTCTATTTGTGTGGGCTGTAATCATTACAGTAATTCTTCTTTT

ATTAACCCTTCCAGTATTAGCCGGTGCAATTACTATACTACTAACGGATCGAAATCTAAA

TACATCATTCTTTGATCCTGCTGGTGGTGGGGATCCTGTTCTTTATCAACACTTATTC

>T11_LN810379_Tubifex_tubifex

-----------------------------------------------GAACTAGACTCTT

AATTCGTCTAGAACTGGCTCAACCTGGCTCATTCCTTGGAAGGGATCAGCTATACAACAC

CCTAGTTACAGCACATGCATTTCTGATAATTTTCTTCATGGTAATACCTATCTATATTGG

GGGTTTCGGAAATTGGTTAGTACCCTTAATGCTGGGAGCTCCTGACATAGCATTCCCCCG

ATTAAATAATTTAAGATTTTGGTTATTACCCCCTTCCTTAATCCTCCTTGTATCCTCCGC

GGCCGTAGAAAAGGGGGCTGGAACAGGGTGAACAGTATATCCGCCCCTAGCTAGAAATCT

GGCTCATTCTGGGCCTTCTGTAGACTTAGCTATTTTCTCTCTACATTTAGCTGGGGTGGC

ATCGATTCTAGGAGCTATTAACTTTATTACTACAATAATTAACATGCGATGAAAAGGGAT

ACGCCTTGAACGAATTCCCCTATTTGTATGAGCTGTAATTCTAACCGTAATCTTACTTTT

ACTAACATTACCAGTTTTAGCTGGTGCCATTACCATGCTACTAACAGATCGAAATCTAAA

CACATCCTTCTTTGACCCTGCTGGGGGTGGAGATCCTGTGCTCTATCAACACTTATTC

>T14_LN810383_Tubificinae_without_hair_setae

TATACTGTACATAATCTTCGGCCTTTACGCCGGAGCAGTTGGAACAGGAACAAGAATACT

AATTCGATTTGAACTATCCCAACCTGGGTCATTTCTAGGAAGAGATCAATTATATAACAC

CCTAGTTACAGCTCATGGATTCCTAATAATCTTCTTTATAGTAATACCAATTTTTGTAGG

TGCATTCGGAAATTCGCTAGTTCCCCTAATACTAGGGGCCCCCGATATAGCATTTCCTCG

CCTAAACAACCTAAGATTCTGATTAATACCACCATCATTAATTCTTCTCATTTCCTCTGC

AGCTGTTGAAAAAGGTGCGGGAACTGGGTGAACTGTATATCCACCTTTAGCTAGAAATCT

AGCACATTCAGGACCATCCGTAGATCTTTGCATTTTCTCCTTACACTTAGCCGGAATTTC

ATCAATTATTGGGGCTATTAACTTTATTACTACAATAATCAATATACGATGAAAAGGTAT

GCGCCTAGAACGAATTCCTCTATTCGTGTGATCAGTAATTATTACAGTTGTTCTTCTTCT

ACTTACACTTCCAGTACTAGCCGGCGCAATTACTATACTTCTTACAGATCGAAATCTAAA

TACCTCATTCTTCGACCCGGCGGGCGGGGGTGACCCAGTCCTATACCAACATCTATTC

>T15_LN810385_Tubificinae_without_hair_setae

CACACTATATGTAATCTTTGGCCTTTGAGCCGGGGCAGTTGGAACTGGAACAAGACTATT

AATTCGATCTGAGCTAGCCCAACCTGGATCATTCCTCGGCAGAGATCAGTTATACAATAC

CTTAGTAACAGCCCATGGATTCCTGATAATCTTCTTTATGGTAATACCAATCTTTATCGG

GGGCTTCGGAAATTGATTAGTCCCTTTAATACTAGGAGCTCCAGATATGGCATTCCCGCG

TCTAAATAATCTGAGATTTTGATTAATACCTCCATCTCTAATTCTTCTTGTTTCCTCTGC

AGCAGTTGAAAAGGGCGCAGGGACAGGATGAACTGTATATCCTCCTCTAGCTAGTAACCT

AGCACATTCAGGACCATCTGTAGATCTTGCCATTTTCTCCTTACATTTAGCAGGAATTGC

ATCAATTCTAGGATCCATCAACTTTATTACCACAATAATTAATATACGATGAAAAGGAAT

GCGCTTAGAGCGCATTCCTTTATTCGTGTGATCAGTAATTATTACTGTTGTCCTTCTCCT

GCTAACTCTACCAGTGTTAGCAGGTGCTATTACTATATTACTCACAGACCGAAATCTAAA

TACTTCGTTCTTTGACCCTGCGGGTGGAGGAGACCCAGTTCTATACCAACACCTATTC

>T17_LN810387_Limnodrilus_hoffmeisteri

CACCCTATACATAATCTTTGGTCTTTGAGCAGGAATAGTAGGCACAGGAACTAGACTTTT

AATTCGATTTGAACTAGCTCAACCTGGCTCATTTCTTGGTAGAGACCAGTTATATAACAC

CCTGGTTACAGCTCACGGGTTTTTAATAATTTTCTTTATAGTAATACCAATCTTCATTGG

TGGATTTGGAAATTGATTAGTACCTTTAATACTTGGGGCACCTGATATGGCATTCCCACG

ACTTAATAACCTAAGATTCTGACTAATACCCCCATCACTAATTCTACTAGTCTCCTCGGC

TGCAGTTGAAAAAGGTGCGGGAACAGGGTGAACTGTATACCCCCCCCTAGCAAGAAATCT

AGCTCATTCCGGACCTTCTGTGGATCTGGCAATTTTTTCATTACACTTGGCGGGGGCCGC

ATCAATCCTTGGGGCAATCAACTTCATTACAACAATAATTAATATACGATGAAAAGGAAT

ACGTTTAGAGCGTATTCCCTTATTTGTGTGATCTGTAATTATCACGGTTGTTTTACTTCT

TCTCACTCTTCCAGTACTTGCCGGAGCTATTACCATACTTTTAACAGATCGAAACCTAAA

CACCTCATTCTTTGATCCGGCGGGGGGAGGTGATCCAGTTTTATATCAACACCTATTT

>T17_LN810391_Limnodrilus_hoffmeisteri

CACCCTATACATAATCTTTGGCCTTTGAGCAGGAATAGTAGGCACAGGAACTAGACTTTT

AATTCGATTTGAGCTAGCACAACCCGGCTCATTTCTCGGTAGAGACCAATTATATAACAC

TCTAGTCACGGCTCACGGATTTTTAATAATTTTCTTTATAGTAATACCTATCTTTATTGG

GGGATTTGGAAATTGATTAGTGCCTTTAATACTTGGAGCACCTGATATGGCATTCCCACG

GCTTAATAACCTAAGATTCTGACTAATGCCCCCATCACTAATTCTACTAGTCTCATCAGC

TGCAGTTGAAAAAGGCGCAGGGACAGGGTGAACTGTATACCCCCCCTTAGCAAGAAATCT

AGCTCATTCCGGGCCTTCTGTAGATCTGGCAATTTTTTCACTACACTTAGCAGGAGCCGC

ATCAATCCTGGGAGCAATTAACTTCATCACAACAATAATCAATATACGATGAAAGGGAAT

ACGCTTAGAGCGTATTCCTTTATTTGTGTGATCCGTAATCATCACAGTTATTTTACTTCT

TCTTACCCTTCCAGTTCTTGCCGGAGCTATCACCATACTTTTAACAGATCGAAACCTAAA

CACCTCATTCTTTGATCCGGCAGGTGGAGGCGATCCAGTTTTATACCAACATCTATTT

>T16_LN810409_Tubificinae_without_hair_setae

-CTCTTATACATGGTCTTCGGACTCTGAGCAGGAATAGTGGGCACAGGAACCAGACTACT

AATTCGATTTGAATTAGCACAACCTGGCTCATTCCTTGGCAGAGATCAATTATATAACAC

CCTAGTTACAGCTCATGGATTTTTAATAATTTTCTTTATAGTTATACCTATTTTCATTGG

GGGCTTCGGTAACTGACTAGTCCCCCTAATACTTGGAGCTCCAGATATAGCCTTCCCACG

ACTCAATAATTTGAGATTTTGATTAATACCCCCATCTCTAATTCTATTAGTCTCCTCGGC

GGCTGTTGAGAAGGGTGCCGGAACAGGATGAACTGTATATCCACCTTTAGCTAGAAACCT

GGCTCATTCTGGGCCCTCTGTAGACCTGGCCATTTTTTCACTTCACTTAGCAGGGGCTGC

ATCAATCTTAGGTGCAATTAACTTCATTACCACAATAATTAATATACGATGAAAAGGAAT

ACGTTTAGAACGTATTCCCCTATTCGTGTGATCAGTTATTATTACGGTTATTTTACTCCT

TCTAACCTTACCAGTTCTAGCAGGGGCAATCACTATACTTCTAACAGACCGAAATCTAAA

CACGTCATTCTTCGACCCTGCGGGTGGTGGCGACCCGGTCCTATATCAACACTTATTC

>T18_LN810411_Limnodrilus_hoffmeisteri

-ACCTTATATATAATCTTCGGCCTATGAGCCGGAATAGTGGGCACAGGAACAAGCCTGCT

AATTCGATTCGAATTAGCACAACCTGGTTCATTCCTCGGAAGAGATCAACTCTATAATAC

CTTAGTGACAGCCCACGGTTTCCTTATAATCTTCTTCATGGTGATACCAATTTTTATTGG

TGGCTTCGGAAATTGACTAGTCCCCCTAATACTAGGAGCTCCTGACATGGCCTTTCCACG

ACTAAATAACCTAAGATTTTGACTAATACCTCCATCACTCATTCTATTAGTTTCATCAGC

CGCAGTCGAAAAGGGAGCGGGGACAGGGTGAACTGTATACCCCCCTCTAGCCAGAAACCT

AGCGCATTCTGGGCCATCTGTGGATCTAGCAATCTTCTCTCTTCACTTAGCCGGGGCTGC

ATCAATTCTAGGTGCTATTAACTTCATTACCACAATAATTAATATACGATGAAAAGGAAT

GCGCCTAGAGCGCATTCCTCTATTTGTATGATCAGTAATCATTACAGTTGTCCTCCTTCT

TCTTACATTACCGGTTTTAGCGGGGGCAATCACCATACTTTTAACAGACCGAAACTTAAA

CACCTCATTCTTCGATCCTGCGGGAGGGGGGGACCCGGTACTATATCAACACTTATTT

>T4_LN810415_Aulodrilus_pluriseta

AACTCTATACTTCATTTTCGGAATCTGGGCCGGTATAGTTGGTACTGGTACCAGACTATT

AATTCGCTTAGAACTGGCACAACCTGGATCCTTCTTAGGAAGTGATCAACTATATAATAC

ATTAGTAACAGCTCACGCATTTCTAATAATCTTCTTCCTAGTTATACCTGTATTCATCGG

GGGATTCGGAAATTGATTAATCCCATTAATACTAGGTGCCCCTGATATAGCATTCCCACG

ACTAAATAACCTAAGATTTTGACTAATACCACCCTCACTAATTCTACTAGTTTCATCCGC

AGCTGTAGAAAAAGGTGCGGGGACAGGTTGAACAGTATATCCACCACTTGCTGGCAATCT

AGCCCATTCAGGACCTTCCGTAGATCTGGCAATCTTCTCTCTACACTTAGCAGGGGTAGC

ATCAATTCTAGGTGCTATTAACTTCATTACCACTATAATCAACATACGATGAAAAGGAAT

GCGCCTAGAACGAATTCCTCTATTTGTCTGAGCCGTAATTCTTACCGTTGTACTCCTTCT

ATTAACCTTACCAGTTCTGGCGGGGGCAATTACTATACTTCTAACTGACCGCAATCTAAA

CACCTCCTTCTTCGATCCAGCTGGGGGCGGTGACCCTGTATTATACCAACATCTATTC

>T12_LN810418_Tubifex_tubifex

AACCCTATACATAATTTTCGGAATCTGAGCTGGTATAGTTGGAACTGGAACTAGGCTATT

AATTCGCCTTGAACTTGCCCAACCTGGGTCCTTTTTAGGAAGAGACCAATTATATAATAC

CCTGGTAACGGCTCACGCATTTTTAATAATCTTCTTCATAGTGATACCAATCTATATTGG

GGGCTTCGGCAACTGACTAGTACCATTAATACTGGGGGCTCCAGACATGGCCTTCCCACG

ATTAAACAACTTAAGATTCTGACTCCTACCCCCATCATTAATCCTTTTAGTGTCCTCTGC

CGCCGTTGAAAAAGGCGCCGGAACTGGCTGAACTGTTTACCCTCCTCTAGCTAGAAATCT

AGCTCACTCTGGACCCTCAGTAGATTTAGCAATCTTTTCTCTACACTTAGCTGGTGTTGC

ATCAATCCTGGGTGCAATCAACTTCATTACTACAATAATCAACATACGATGAAAAGGTAT

GCGACTAGAACGTATTCCTTTATTCGTATGATCAGTAATTATCACTGTAATTCTACTCCT

ACTCACACTTCCAGTTTTAGCTGGGGCTATCACAATACTTCTAACAGACCGGAATCTAAA

TACATCATTTTTCGATCCTGCTGGAGGGGGTGACCCTGTACTATATCAACACCTATTC

>T9_LN810420_Tubifex_tubifex

AACCCTTTATATCGTATTTGGAATTTGAGCTGGAATAGTGGGAACAGGTACAAGCCTCTT

AATCCGCTTAGAATTAGCTCAACCTGGCTCTTTCCTGGGCAGAGACCAACTATATAACAC

TCTAGTTACAGCCCATGCATTCCTGATAATCTTCTTTATAGTAATACCTATCTACATTGG

TGGTTTTGGCAATTGACTGGTCCCACTTATATTAGGGGCGCCCGATATAGCATTTCCACG

ATTAAATAACTTAAGATTTTGACTACTGCCCCCTTCCTTAATTCTTCTAGTATCATCTGC

AGCGGTTGAGAAAGGGGCTGGAACTGGGTGAACCGTTTATCCTCCACTATCAAGAAATCT

TGCACACTCGGGCCCATCCGTAGACCTTGCAATCTTCTCACTCCACTTAGCCGGAGTAGC

CTCAATTTTAGGCGCTATCAATTTCATCACCACAATAATTAACATACGATGAAAAGGTAT

ACGGTTAGAACGAATTCCATTATTCGTGTGATCAGTAATTCTGACAGTAATTCTATTACT

GCTTACCTTACCTGTACTAGCAGGCGCTATTACTATACTCCTAACAGATCGAAACCTAAA

TACATCATTCTTTGATCCTGCGGGTGGTGGTGATCCAGTTCTTTACCAACATCTATTC

>T10_LN810423_Tubifex_tubifex

AACCCTTTATATAGTATTTGGTATTTGAGCTGGTATAGTAGGCACTGGAACAAGTTTATT

AATTCGTTTAGAATTAGCTCAACCTGGCTCCTTCTTAGGCAGAGATCAATTATATAACAC

CTTAGTTACAGCCCACGCCTTCCTGATAATCTTCTTTATGGTAATGCCAATCTACATTGG

TGGCTTCGGAAACTGACTAGTACCACTAATGCTAGGGGCACCAGACATAGCATTCCCCCG

ACTAAATAACCTAAGATTTTGACTATTACCTCCATCCCTAATCTTACTTGTATCATCTGC

TGCAGTAGAAAAAGGGGCAGGAACAGGTTGAACTGTATACCCTCCCCTAGCTAGAAATCT

AGCACATTCCGGACCCTCCGTAGACCTGGCTATCTTCTCACTACATTTAGCTGGTGTAGC

ATCAATTCTAGGAGCCATTAATTTCATTACCACAATAATCAATATACGCTGAAAAGGTAT

ACGCCTAGAACGTATTCCTTTATTCGTATGATCAGTTATTATTACTGTAATCCTTCTATT

ACTCACACTCCCAGTACTAGCCGGTGCTATTACTATACTTCTTACAGACCGAAATCTAAA

CACCTCATTCTTCGACCCTGCTGGTGGTGGAGACCCTGTCCTTTACCAACATCTATTC

>T10_LN810424_Tubifex_tubifex

AACCCTTTATATTGTATTTGGTATTTGAGCCGGTATAGTAGGCACCGGAACAAGTTTACT

GATTCGTCTAGAATTAGCTCAACCTGGCTCCTTCTTAGGCAGAGATCAATTATATAATAC

CCTAGTTACAGCCCACGCCTTCCTAATGATTTTCTTCATAGTAATGCCAATCTACATTGG

TGGCTTCGGAAACTGATTAGTACCTTTAATGCTGGGGGCACCAGACATGGCATTCCCTCG

ACTAAATAACTTAAGATTTTGATTACTACCCCCATCCTTAATCTTACTCGTATCGTCTGC

TGCAGTAGAAAAGGGGGCCGGAACAGGCTGAACTGTGTACCCTCCCTTAGCCAGAAACTT

AGCTCACTCTGGACCTTCCGTAGACCTGGCGATCTTCTCGCTACATTTAGCAGGTGTAGC

ATCAATCTTAGGGGCCATTAATTTCATTACCACAATAATTAATATACGTTGAAAAGGTAT

GCGCCTAGAACGAATTCCTTTATTTGTATGATCAGTTATTATCACTGTAATCCTTCTATT

ACTCACGCTCCCAGTACTAGCCGGTGCTATTACTATACTTCTTACAGATCGAAACCTAAA

TACCTCATTCTTCGACCCAGCCGGTGGTGGGGACCCTGTCCTTTACCAACACCTATTC

>T11_962_Tubifex_tubifex_2ind

AACTCTATATATTATTTTCGGGATTTGAGCTGGAATGGTCGGGACAGGAACTAGCCTCTT

AATTCGTCTAGAACTGGCTCAACCTGGCTCATTCCTTGGAAGGGATCAACTATACAACAC

CCTAGTTACAGCACACGCATTTCTGATAATTTTCTTCATGGTAATACCTATCTATATCGG

GGGCTTCGGCAATTGACTAGTGCCTTTAATACTGGGGGCCCCTGACATAGCATTCCCACG

ATTAAATAATTTAAGATTTTGACTATTACCTCCTTCCTTAATCCTCCTTGTATCCTCCGC

AGCCGTAGAAAAGGGGGCTGGAACAGGGTGAACAGTATATCCACCCCTAGCTAGAAATCT

GGCTCATTCTGGACCTTCTGTAGACTTAGCTATTTTCTCCCTACATTTAGCGGGAGTAGC

ATCGATTCTAGGGGCTATTAACTTTATTACTACAATAATTAACATGCGGTGAAAAGGGAT

ACGTCTTGAACGAATTCCTCTATTTGTATGAGCCGTAATTCTAACTGTAATCTTACTTCT

ACTAACATTACCAGTTTTAGCGGGTGCTATTACCATGCTACTGACAGATCGAAACCTAAA

CACATCCTTCTTTGACCCTGCTGGGGGTGGTGATCCTGTACTCTATCAACACTTATTC

>T23_929_Limnodrilus_udekemianus

CACCCTTTATTTTATCTTCGGCCTATGAGCCGGTATAGTCGGTACCGGAACCAGATTATT

AATTCGATTTGAACTAGCACAACCCGGATCATTTCTAGGTAGAGACCAACTCTATAACAC

ACTAGTAACAGCACATGGATTTCTAATAATTTTCTTCCTTGTAATACCAGTATTTATTGG

GGGATTCGGCAATTGACTAGTACCATTAATACTTGGGGCTCCCGACATAGCATTCCCACG

ACTAAATAATCTTAGATTTTGACTAATACCTCCATCACTTATTCTACTTGTATCCTCAGC

AGCCGTAGAAAAAGGGGCTGGAACAGGGTGAACTGTATATCCTCCATTAGCAGGGAATCT

TGCTCATTCAGGCCCTTCTGTAGACCTTGCCATTTTTTCCCTTCATCTGGCCGGAATTTC

TTCAATTTTAGGGGCTATTAACTTCATCACCACTATAATCAATATACGATGAAAAGGAAT

ACGACTTGAACGAATTCCTTTATTTGTGTGATCTGTAATTATCACTGTAGTCTTACTTCT

ACTAACTCTCCCTGTATTAGCAGGTGCAATTACAATACTTCTAACAGATCGAAATCTGAA

TACATCATTCTTTGATCCTGCCGGAGGAGGTGACCCTGTCCTATATCAACATCTATTC

>T32*_959_Tubificinae_with_hair_setae

ATTTTTATATATATTATTTGGGCTATGAGCTGGAATAGTAGGAACTGGTACCAGTCTTCT

AATTCGGTTAGAACTTGCTCAACCAGGCTCATTCCTGGGAAGAGATCAACTTTATAATAC

TATTGTAACAGCACATGCATTCCTGATAATCTTCTACATAGTAATACCAATTTATGTAGG

AGGATTTGGAAACTATTTAATACCTTTAATACTGGGGGCACCAGATATAGCATTCCCACG

ACTAAATAACCTGAGATTCTGATTAATACCGCCATCTTTAATTCTTCTAGTAGCATCAGC

TGCTGTTGAAAAAGGGGCCGGAACTGGATGAACTGTTTACCCTCCTCTAGCTAGAAATCT

GGCACATTCTGGTCCATCAGTAGATCTAGCTATTTTTGCCTTACATCTTGCTGGTGCAGC

CTCTATTATCGGAGCCATTAATTTCATTACAACAATAATTAATATACGCTGAAAAGGGAT

GCGACTAGAGCGTATTCCCCTGTTCGTGTGATCTGTAATTATTACTGTAGTTCTATTATT

ACTTACACTTCCTGTTCTTGCAGGTGCCATTACTATACTCCTAACAGATCGAAATCTAAA

TACATCATTCTTCGATCCTGCTGGTGGCGGTGACCCTGTACTATACCAACATCTATTC

>T6_1003_Lophochaeta_ignota_12ind

AACTCTCTATATAATCTTCGGCCTATGAGCGGGAATAGTCGGCACCGGTACTAGACTACT

AATTCGACTAGAATTAGCTCAACCCGGATCATTCCTTGGTAGTGATCAACTATATAATAC

ACTTGTTACTGCCCATGGATTCCTTATAATTTTCTTCATGGTAATACCAGTATTTATTGG

GGGATTTGGTAACTGACTAGTCCCATTAATACTTGGTGCCCCAGATATAGCCTTCCCACG

AATAAATAATTTAAGATTTTGATTAATACCCCCCTCTTTAATCCTTCTCGTATCTTCAGC

AGCCGTTGAAAAAGGTGCTGGAACAGGCTGAACAGTATATCCTCCATTAGCCGGGAATTT

AGCTCACTCAGGACCATCCGTAGACCTAGCTATTTTCTCACTGCATTTAGCCGGAGCTGC

CTCAATTTTAGGTGCAATTAACTTTATTACGACTATAATTAACATGCGATGAAAAGGAAT

ACGCCTAGAACGCATTCCTCTATTTGTTTGATCCGTAATTATCACAGTAGTCCTACTTTT

ATTAACACTACCAGTTCTAGCAGGGGCCATTACAATATTACTTACAGACCGAAACTTAAA

TACCTCATTCTTTGACCCTGCTGGAGGTGGAGACCCTGTTTTATATCAACATCTATTC

>T22_991_Limnodrilus_claparedianus_2ind

CACTCTCTACATAATTTTCGGCCTTTGAGCCGGAATAGTCGGTACTGGAACAAGCCTACT

AATTCGATTTGAGTTAGCTCAACCCGGGTCATTCTTAGGCAGAGACCAGTTATATAACAC

TTTAGTGACAGCCCACGGATTCTTAATAATTTTCTTTATGGTAATGCCAATCTTTATCGG

TGGATTTGGAAATTGACTAATTCCCTTAATACTTGGGGCACCAGATATAGCATTCCCACG

ATTAAATAATCTCAGCTTTTGACTAATGCCACCATCGTTAATTCTATTAGTGTCATCTGC

TGCTGTAGAAAAAGGTGCTGGCACAGGTTGAACTGTATATCCGCCACTGGCAAGAAATTT

AGCACATTCGGGGCCATCTGTAGATTTAGCAATTTTCTCCCTTCATCTTGCTGGTGCAGC

TTCAATTCTAGGGGCAATTAACTTTATTACAACAATAATTAACATGCGATGAAAAGGAAT

ACGCCTCGAACGAATTCCATTATTCGTATGATCTGTAATTATTACTGTTATTCTACTACT

TCTGACCCTTCCGGTACTTGCGGGGGCTATCACGATACTATTAACAGACCGAAATCTAAA

TACATCGTTCTTCGACCCTGCGGGGGGGGGAGACCCTGTACTTTATCAACACTTATTC

>T33*_995_Tasserkidrilus_kessleri_2ind

AACACTATATATAATCTTTGGATTCTGAGCCGGAATAGTTGGTACTGGAACAAGACTTTT

AATTCGATTTGAATTAGCCCAACCTGGGTCCTTCCTCGGCAGAGATCAACTCTACAATAC

ACTAGTTACAGCCCATGCATTCTTAATAATTTTCTTCCTTGTTATGCCAGTCTTCATTGG

TGGATTTGGCAATTGATTAGTTCCCTTAATACTAGGAGCTCCTGATATAGCATTCCCACG

ATTAAATAATTTAAGATTCTGACTACTACCCCCCTCCCTGATCCTACTAGTATCCTCGGC

CGCCGTAGAAAAGGGGGCAGGAACAGGATGAACAGTTTATCCACCATTAGCTGGCAATTT

AGCCCATTCCGGCCCATCAGTAGACCTGGCTATTTTCTCTCTTCACCTGGCAGGAATCGC

TTCTATTTTAGGGGCAATTAATTTTATTACTACAATAATTAATATACGATGAAAAGGTAT

ACGTCTAGAACGTATCCCTCTATTTGTATGAGCAGTAATCCTGACAGTAATTCTTCTTCT

CCTAACTCTCCCTGTCCTAGCAGGTGCTATTACTATACTCCTAACTGACCGAAATCTAAA

TACATCATTCTTTGACCCTGCTGGGGGTGGTGATCCTGTGCTATATCAACATCTATTT

>T23_1016_Limnodrilus_udekemianus

CACCCTTTACTTTATCTTCGGCCTATGAGCCGGTATAGTTGGTACAGGTACCAGACTATT

AATTCGATTTGAGCTAGCACAACCCGGATCATTCCTAGGCAGAGACCAACTCTATAACAC

GCTAGTAACAGCACATGGATTTCTAATAATCTTCTTCCTTGTAATACCAGTATTTATTGG

GGGATTCGGTAATTGATTAGTACCATTAATACTTGGGGCTCCTGACATAGCATTCCCACG

ATTAAATAATCTAAGATTTTGATTAATACCTCCATCACTTATTCTACTTGTATCCTCAGC

AGCAGTGGAAAAAGGAGCTGGAACAGGATGAACTGTATACCCTCCATTAGCAGGAAATCT

TGCCCATTCCGGACCTTCTGTAGATCTTGCTATTTTCTCTCTTCACCTGGCTGGAATTGC

CTCAATTCTAGGAGCTATCAACTTTATCACCACTATAATTAATATACGATGAAAAGGAAT

ACGACTCGAACGAATTCCTTTATTTGTGTGATCTGTAATTATCACTGTAGTCTTACTTCT

ACTAACTCTCCCTGTATTAGCAGGGGCAATTACAATGCTCCTAACAGATCGAAATCTAAA

TACATCATTTTTTGATCCTGCCGGAGGAGGAGATCCTGTCCTATATCAACACTTATTC

>LC3_LN810249_Eiseniella_tertraedra

------------CATTTTAGGTATTTGAGCTGGGATAGTGGGAGCTGGTATAAGCTTACT

AATTCGAATCGAATTAAGCCAACCAGGAGCCTTCCTAGGTAGTGACCAATTATATAACAC

TATTGTTACAGCACATGCATTCGTAATAATCTTCTTCCTAGTAATACCCGTATTCATTGG

TGGATTCGGAAACTGATTGCTACCTCTAATACTAGGTGCACCAGATATAGCATTTCCACG

TTTAAACAACATAAGATTTTGACTTTTACCTCCTTCTTTAATTCTACTAGTATCTTCCGC

AGCCGTAGAAAAGGGGGCCGGGACAGGGTGAACTGTTTATCCGCCCTTAGCAAGAAACTT

GGCCCATGCAGGACCATCAGTAGATCTAGCTATTTTCTCCTTACACTTAGCAGGAGCTTC

ATCTATTTTAGGTGCCATCAACTTTATTACTACAGTCATTAATATGCGATGAAGGGGCTT

ACGATTAGAACGAATTCCCCTTTTCGTGTGAGCTGTACTGATTACAGTAATTCTTCTATT

ATTATCGTTGCCCGTGCTAGCAGGAGCAATTACCATACTATTAACCGACCGAAATCTCAA

TACATCATTCTTTGACCCGGCTGGTGGTGGTGACCCAATTCTGTACCAACACCTCTTC

>E3_LN810245_Lumbricillus_rutilus

-ACACTATATTTTATTTTAGGAGTATGAGCTGGAATACTAGGAGCAGCCATAAGACTTTT

AATTCGAATTGAATTAAGACAACCTGGCGCTTTTTTAGGAAGAGATCAGCTTTATAATAC

TATCGTAACAGCTCATGCATTCTTAATAATTTTTTTCTTAGTTATACCAGTATTTATTGG

TGGATTTGGAAATTGATTAATTCCGCTAATATTGGGAGCTCCTGACATAGCATTCCCTCG

TCTTAACAATATAAGATTTTGACTTCTACCTCCAGCTCTTTTACTTCTAGTTTCTTCAGC

AGCAGTAGAAAAAGGTGCTGGGACTGGCTGAACAGTTTACCCACCTCTAGCAAGAAATCT

AGCTCATGCAGGTCCATCCGTAGATTTAGCAATTTTCTCTCTTCATTTAGCCGGTGCCTC

ATCTATTCTTGGAGCAGTAAACTTTATTACTACAGTAGTAAATATACGTTGACAAGGTCT

TCGACTTGAACGAATTCCTCTTTTTGTATGAGCAGTAGTAATTACAACAGTTCTTCTTCT

TCTATCTCTTCCAGTTCTTGCAGGGGCAATTACAATACTACTAACTGATCGAAATCTAAA

CACTTCATTTTTTGACCCAGCCGGAGGTGGAGATCCTGTTCTTTATCAACATTTATTT

>H1*_885_Haplotaxis_gordioides_2ind

AACCCTTTACTTTATCCTAGGCATCTGAGGGGGACTTCTAGGAACAAGAATAAGAATAGT

AATCCGAATTGAACTAAGACAACCAGGGTCATTCCTTGGTAGAGATCAATTATACAATAC

CATTGTTACTGCCCATGCCTTCCTAATAATTTTCTTTCTTGTCATACCAGTATTTATTGG

AGGGTTCGGAAACTGACTCTTACCTCTAATGTTAGGAGCCCCCGACATAGCATTCCCACG

ACTCAACAATATAAGATTTTGACTTCTACCTCCTGCAACAATCCTTCTTGTGTCCTCAGC

AGCAGTAGAAAAAGGAGCAGGAACTGGCTGAACTGTTTACCCGCCCTTAGCCAGAAATCT

TGCACATGCTGGCCCTTCTGTTGATCTCGCCATTTTCTCTCTTCATTTAGCAGGAGTTTC

CTCCATTTTAGGAGCAGTAAATTTCATCACCACTGTTGTCAATATACGATGAAATGGCCT

GCGGTTAGAACGAATTCCTTTATTCGTATGATCCGTAACTATTACAGTGGTTCTTCTCCT

TTTATCTCTACCAGTGCTAGCCGGCGCTATTACCATACTACTAACTGACCGAAATCTTAA

TACATCATTCTTCGACCCAGCTGGAGGAGGAGATCCAGTTCTGTATCAACACCTATTC

>LL3_LN810273_Stylodrilus_heringianus

AACCTTATATTTCATCTTAGGAGTTTGAGCTGGGATAGTAGGAGCAGGGATGAGACTATT

AATTCGAGTTGAATTAACACAACCCGGGTCATTTTTAGGAAGTGACCAACTATATAATAC

TATTGTTACGGCTCACGCATTTATTATAATTTTCTTTATAGTTATACCTATATTTATTGG

TGGTTTTGGTAACTGAATACTACCGTTAATATTAGGAGCACCAGACATAGCATTTCCACG

ACTTAACAACCTAAGATTTTGATTACTACCACCTTCTTTAACTTTATTAGTAGCATCGGC

TGCAGTAGAAAAAGGAGCCGGCACAGGATGAACAGTTTATCCACCTCTATCAAGAAATTT

AGCTCATGCTGGACCCTCAGTAGACCTGGCAATCTTCTCACTCCATCTAGCAGGAGCCTC

CTCCATTTTAGGAGCCATTAACTTTATTACGACTGTAATTAATATACGATGAAATGGACT

ACGACTTGAACGAGTTCCATTATTTGTATGAGCAGTAACAATTACAGTAGTTCTATTACT

ATTATCATTACCAGTACTAGCTGGAGCAATCACTATACTTCTTACAGATCGAAACTTAAA

TACAACATTTTTTGATCCAGCTGGAGGTGGAGACCCCGTACTATATCAACATTTATTT

>N5_LN810257_Ophidonais_serpentina

TACATTATATTTAATCTTAGGAGTATGAGCAGGAATAGTTGGTACAGGAACAAGAATACT

GATTCGAATTGAACTAGCTCAACCAGGAGCTTTTCTAGGAAGAGATCAATTATATAACAC

TCTAGTAACAGCACATGCGTTTTTAATAATTTTCTTTTTAGTTATACCTGTATTTATTGG

CGGATTCGGAAACTGACTTCTTCCATTAATATTAGGTGCTCCAGATATGGCATTCCCACG

ACTAAATAATCTTAGATTCTGACTTCTACCACCATCATTAATTCTATTAATTTCATCTGC

AGCCGTTGAAAAAGGTGCTGGAACAGGATGAACTGTATATCCTCCATTATCAAGAAATCT

AGCTCACGCTGGACCTTCAGTTGACATGGCTATTTTTTCACTACATCTAGCAGGTGCATC

TTCTATTTTAGGTGCAGTTAACTTCATTACTACAGTAATAAACATACGATGAAATGGAAT

ACGACTTGAACGAGTACCATTATTTGTATGAGCTGTAACACTTACTGTAATTCTTCTTCT

TTTATCATTACCTGTATTAGCTGGTGCAATTACCATACTATTAACAGATCGAAATCTAAA

TACCTCATTCTTCGATCCTGCAGGAGGGGGAGACCCAATTTTATACCAACATTTATTC

**Figure 2**

>E7*_819_Achaeta_sp_3ind

--CTCTATNCTTTATCCTCGGAATATGAGCTGGCATAATAGGTGCAGCTATAAGACTCCT

CATCCGATTTGAACTAAGTCAACCCGGATCATTTTTAGGGAGAGACCAATTATATAATAC

CATTGTAACAGCACATGCATTCTTAATAATTTTCTTTCTTGTAATACCAGTATTTATTGG

AGGATTTGGAAATTGACTCATTCCATTAATACTTGGAGCTCCAGATATAGCTTTTCCACG

ATTAAATAATATAAGATTCTGATTACTTCCACCATCCCTCATATTACTTCTTTCTTCTAC

AGCTGTAGAAAAAGGTATAGGAACAGGATGAACAGTATATCCTCCTTTAGCTAGAAACAT

TGCTCATGCAGGACCATCTGTAGATTTAGCCATTTTCTCTCTTCACTTAGCAGGAGCTTC

ATCAATTCTAGGAGCAGTAAATTTCATTACTACTGTAATTAATATACGATGACAAGGTAT

ACGACTAGAACGAATTCCACTATTTGTATGAGCTATAATAATCACAGTAGCCCTCCTACT

TTTAGCACTACCAGTCCTAGCTGGCGCAATTACTATGCTTCTTACTGATCGAAACCTTAA

CACATCATTCTTTGATCCAGCTGGTGGAGGTGACCCTATTCTATATCAACATTTATTT

>E12*_806_Fridericia_sp

--CACTCTTCTTCATTTTAGGAGTTTGAGCAGGAATAATAGGAGCCGCTATAAGACTTCT

AATTCGAATTGAACTTAGACAACCAGGATCATTTCTTGGCAGAGACCAACTATATAATAC

AATTGTTACAGCTCATGCATTTCTTATAATCTTTTTTTTAGTGATACCAGTATTTATTGG

GGGATTTGGAAACTGACTCTTACCATTAATATTAGGTGCACCAGATATAGCATTCCCACG

TCTAAATAATATAAGATTTTGATTACTTCCTCCGTCTCTAATACTTCTTCTATCATCTGC

CGCAGTAGAAAAAGGTGCAGGTACAGGATGAACAGTATACCCACCATTAGCAAGAAATAT

AGCCCACGCAGGTCCATCAGTAGATTTAGCTATTTTTTCTCTACATTTAGCAGGAGCTTC

ATCTATTCTAGGAGCCGTTAATTTTATTTCAACAGTAATCAATATACGCTGACAAGGTCT

TCAACTTGAACGAATTCCATTATTTGTATGAGCTGTCACTATTACTGTTGTACTCTTATT

ATTATCCTTACCAGTATTAGCTGGTGCTATTACCATACTTCTCACAGATCGTAACCTAAA

TACATCTTTCTTCGACCCCGCTGGCGGAGGAGACCCTATTTTGTATCAACACTTATTT

>E13*_807_Lumbricillus_sp

--CACTTTATTTTATCCTCGGAGTATGAGCAGGTATAATAGGCGCAGCTATAAGCCTAAT

TATTCGAACAGAACTTAGCCAACCAGGATCATTCTTAGGTAGGGACCAGTTATACAATAC

TGTTGTAACAGCCCACGCATTTTTAATAATTTTCTTTTTAGTTATACCAGTATTCATTGG

GGGCTTTGGTAATTGGCTTTTACCCCTAATACTCGGAGCACCTGATATAGCATTTCCGCG

ACTCAACAATATAAGATTTTGACTTCTTCCCCCTTCACTACTTCTACTTCTCTCATCAGC

CGCAGTAGAAAAAGGTGCTGGAACTGGTTGAACAGTTTACCCACCCCTAGCAAGAAATAT

GGCACATGCAGGACCATCTGTAGATTTAGCAATTTTTTCCCTTCATTTAGCAGGGGCTTC

CTCTATTCTTGGTGCCGTAAATTTTATTTCCACTGTAATCAATATACGATGACAAGGCCT

CCAGTTAGAGCGAATTCCACTATTTGTATGAGCAGTTACAATCACAGTAGTACTTCTTCT

CTTATCTTTACCAGTATTAGCTGGTGCTATTACCATATTACTAACAGATCGAAACCTAAA

TACTTCATTCTTTGACCCTGCAGGTG--------------------------------

>E6*_808_Achaeta_sp

--CATTATATTTTATCCTAGGGATCTGAGCAGGTATAATAGGAGCAGCTATAAGCCTACT

AATTCGATTTGAACTAAGACAACCAGGCTCATTTTTAGGAAGAGATCAACTGTACAACAC

AATTGTAACAGCTCATGCATTTTTAATAATCTTCTTCCTAGTAATACCAGTATTTATTGG

GGGATTTGGTAACTGATTACTACCTCTAATGTTAGGTGCACCAGATATAGCATTCCCACG

ACTTAACAACATAAGATTTTGATTATTACCCCCCTCACTTATACTTCTTCTATCCTCAGC

AGCAGTTGAAAAAGGAGCAGGTACCGGATGAACAGTATACCCACCACTAGCCAGAAACAT

TGCACACGCTGGCCCATCAGTAGACCTAGCAATCTTTTCTCTACATCTTGCAGGGGCATC

CTCAATTCTTGGAGCAGTAAACTTCATTACTACAGTAATCAATATACGATGACAAGGAAT

ACGATTAGAACGAATCCCCCTTTTTGTATGAGCAGTAGTAATTACAGTAGTTCTACTACT

ACTATCTCTACCAGTCCTTGCTGGGGCTATTACTATATTACTTACAGATCGCAATCTAAA

CACATCATTTTTTGACCCGGCAGGTGGTGGAGACCCCATTCTATACCAACATTTATTT

>E15*_809_Fridericia_sp

--CACTATATTTCATTCTAGGAGTATGAGCAGGCATAATAGGAGCAGCCATAAGACTATT

AATTCGAATTGAATTAAGACAACCAGGCTCCTTTCTAGGCAGAGACCAACTATATAACAC

AATCGTAACTGCCCATGCCTTTCTCATAATTTTTTTTCTAGTAATACCAGTATTTATTGG

GGGATTCGGAAATTGATTACTTCCATTAATACTAGGGGCACCAGACATAGCCTTCCCGCG

ACTCAATAACATAAGATTCTGACTCTTGCCCCCCTCCCTTCTCCTTCTTTTATCTTCTGC

AGCAGTAGAAAAAGGTGCAGGTACAGGTTGAACAGTCTATCCTCCGCTAGCTAGAAATAT

GGCTCACGCCGGACCATCAGTAGATCTAGCTATTTTTTCTCTACATTTAGCCGGAGCCTC

ATCCATTCTAGGGGCAGTAAATTTTATTTCAACCGTCATTAACATACGATGACAAGGTCT

ACAATTAGAACGAATTCCATTATTTGTATGAGCTGTAACCATTACAGTAGTTCTTCTATT

ATTATCTCTACCAGTTCTAGCCGGAGCAATTACTATACTTTTAACAGATCGAAACCTTAA

TACATCATTTTTTGATCCAGCCGGAGGTGGAGATCCAATTTTATACCAACACCTATTT

>E16*_810_Henlea_perpusilla_4ind

------------------------------------GATAGGAGCAGCCATAAGCCTTCT

AATTCGAATTGAACTAAGTCAACCAGGTTCATTCCTCGGAAGAGATCAACTCTACAACAC

TATTGTTACTGCACATGCATTTCTTATAATTTTTTTCCTAGTTATACCTGTATTTATTGG

GGGGTTCGGAAACTGACTTCTCCCATTAATACTGGGAGCCCCAGATATAGCTTTTCCCCG

ACTAAATAACATAAGATTTTGACTTCTTCCCCCATCACTTCTTCTTCTACTTTCCTCTGC

AGCTGTGGAAAAAGGAGCCGGAACCGGCTGAACTGTATACCCCCCCCTATCAAGAAATAT

CGCTCATGCGGGCCCATCTGTAGACCTAGCCATTTTCTCCCTCCACTTAGCCGGAGCATC

CTCCATCTTAGGTGCAGTAAACTTTATCACTACTGTTATTAACATACGTTGACAAGGCCT

TCACCTAGAACGAATTCCCCTATTTGTATGAGCTGTAACAATTACAGTAGTCCTTCTCCT

CTTATCTTTACCAGTGTTAGCCGGAGCAATTACTATACTACTAACTGACCGAAACCTAAA

CACATCCTTTTTCGACCCAGCTGGAGGAGGTGACCCAATTCTATATCAACACCTATTT

>E14*_813_Fridericia_sp_2ind

------------------------------------AATAGGAGCCGCAATAAGATTATT

AATTCGAATTGAACTCAGACAACCAGGCTCCTTCCTTGGAAGAGATCAGCTTTATAACAC

TATCGTAACAGCACATGCATTTCTAATAATCTTTTTCTTAGTAATACCAGTATTTATTGG

AGGCTTTGGTAATTGACTTTTACCATTAATATTAGGAGCCCCAGATATAGCATTCCCCCG

ACTAAATAATATAAGATTCTGATTACTACCCCCATCTTTAATACTTCTTCTATCTTCAGC

TGCAGTAGAAAAAGGTGCTGGCACTGGGTGAACTGTATACCCCCCACTAGCAAGAAATAT

AGCTCATTCTGGGCCATCCGTTGACTTAGCAATTTTTTCTCTACATCTTGCAGGAGCATC

TTCAATTCTAGGCGCAGTAAATTTTATTTCCACAGTTATTAATATGCGATGACAAGGCCT

ACAACTAGAACGAATTCCACTATTTGTATGAGCTGTTACAATCACAGTAGTATTACTACT

ACTATCTCTCCCAGTTCTAGCTGGCGCAATTACTATATTATTAACTGATCGAAATCTAAA

TACATCGTTCTTCGACCCTGCTGGTGGAGGAGATCCAATCCTATATCAACACCTCTTT

>E5*_817_Marionina_argentea

--CTCTATATTTTATTTTAGGAACTTGAGCTGGGATATTAGGAGCAGCTATAAGATTATT

AATTCGTTTTGAACTAGGTCAACCTGGCTCATTTTTAGGTAGAGATCAATTATATAATAC

TATTGTAACTGCTCATGCCTTTTTAATAATTTTTTTTTTAGTGATACCTGTCTTTATTGG

AGGATTTGGAAATTGATTATTACCACTAATATTAGGTGCGCCAGACATGGCTTTCCCTCG

ACTTAATAATTTGAGATTTTGGCTATTACCCCCATCTTTGCTACTTTTAGTGTCTTCAGC

TGCGGTAGAAAAGGGGGCGGGAACAGGTTGGACAGTGTACCCACCACTTTCGTCAAATAT

TGCCCATTCAGGTCCATCTGTAGACTTAGCTATCTTTTCTCTTCATCTAGCAGGAGCATC

ATCTATTTTAGGGGCCATTAATTTTATTACAACTGTAATTAATATACGATGACGGGGATT

GCAATTAGAGCGTATTCCTTTATTTGTTTGAGCAGTTACTATTACAGTTGTGCTATTATT

ACTATCTTTACCTGTATTAGCTGGAGCTATTACTATATTGTTAACTGATCGTAATTTAAA

TACATCATTTTTTGATCCTGCTGGAGGAGGGGACCCAATTTTATATCAACACTTATTT

>N7*_820_Vejdovskyella_intermedia

--CATTATATTTAATTTTAGGGGTATGAGCAGGAATAGTTGGAACCGGAACTAGAATATT

AATTCGAGTTGAATTATCACAACCAGGAGCTTTTCTTGGGAGAGACCAATTATATAATAC

ATTAGTAACTGCACACGCATTCTTAATAATCTTTTTCTTAGTAATACCCGTATTTATTGG

TGGATTCGGAAATTGACTATTACCATTAATACTAGGAGCACCGGATATAGCATTCCCACG

ACTTAATAATCTAAGATTTTGACTATTACCACCATCATTAATTCTATTAATTTCTTCAGC

TGCAGTAGAAAAAGGAGCAGGAACAGGCTGAACCGTATATCCACCACTTTCAAGAAATCT

AGCACATGCGGGACCATCAGTAGATATAGCAATTTTTTCACTACATTTAGCTGGTGCATC

ATCTATTCTAGGAGCCGTAAATTTTATTACAACTGTAATAAATATACGATGAAATGGAAT

ACGATTAGAACGAGTACCATTATTCGTTTGATCGGTAATATTAACAGTTATTCTTCTTAT

TCTATCATTACCTGTACTTGCGGGAGCA-ATACAATATTATTAACAGATCGAAACTTAAA

TACCTCATTCTTCGATCCTGCTGGGGGTGGAGACCCAATTCTTTATCAACATCTATTT

>H1*_885_Haplotaxis_gordioides_2ind

AACCCTTTACTTTATCCTAGGCATCTGAGGGGGACTTCTAGGAACAAGAATAAGAATAGT

AATCCGAATTGAACTAAGACAACCAGGGTCATTCCTTGGTAGAGATCAATTATACAATAC

CATTGTTACTGCCCATGCCTTCCTAATAATTTTCTTTCTTGTCATACCAGTATTTATTGG

AGGGTTCGGAAACTGACTCTTACCTCTAATGTTAGGAGCCCCCGACATAGCATTCCCACG

ACTCAACAATATAAGATTTTGACTTCTACCTCCTGCAACAATCCTTCTTGTGTCCTCAGC

AGCAGTAGAAAAAGGAGCAGGAACTGGCTGAACTGTTTACCCGCCCTTAGCCAGAAATCT

TGCACATGCTGGCCCTTCTGTTGATCTCGCCATTTTCTCTCTTCATTTAGCAGGAGTTTC

CTCCATTTTAGGAGCAGTAAATTTCATCACCACTGTTGTCAATATACGATGAAATGGCCT

GCGGTTAGAACGAATTCCTTTATTCGTATGATCCGTAACTATTACAGTGGTTCTTCTCCT

TTTATCTCTACCAGTGCTAGCCGGCGCTATTACCATACTACTAACTGACCGAAATCTTAA

TACATCATTCTTCGACCCAGCTGGAGGAGGAGATCCAGTTCTGTATCAACACCTATTC

>N4_888_Nais_elinguis_4ind

TACATTATACTTAATTCTAGGAGTATGAGCGGGAATAGTTGGAACTGGAACAAGAATACT

AATTCGAATTGAACTTGCTCAACCAGGAGCCTTCCTCGGAAGAGACCAACTCTATAACAC

CCTAGTTACAGCTCATGCATTTTTAATAATTTTCTTTCTTGTAATGCCAGTATTTATTGG

TGGATTCGGAAATTGACTTCTACCACTAATACTTGGAGCCCCAGATATAGCATTTCCACG

ATTAAATAATCTAAGATTTTGATTACTACCACCATCATTAATTATACTAATTTCATCAGC

CGCAGTTGAAAAAGGTGCAGGAACAGGATGAACTGTTTACCCTCCATTATCAAGAAATTT

AGCTCATGCTGGACCATCAGTTGACATGGCTATTTTTTCTCTTCATTTAGCAGGTGCATC

ATCTATTTTAGGTGCAGTAAATTTTATTACTACAGTAATAAATATACGATGAAACGGTAT

ACGATTAGAACGTTTACCTTTATTTGTATGAGCTGTATTCCTTACTGTAATTCTCCTTCT

ACTATCGCTACCAGTATTAGCTGGCGCTATTACAATACTTCTTACAGACCGTAATTTAAA

TACCTCATTCTTTGATCCAGCAGGAGGAGGAGATCCGATTCTTTATCAACACTTATTT

>LL3_918_Stylodrilus_heringianus_28ind

AACCTTATATTTCATCTTAGGAGTTTGAGCTGGAATAGTAGGAGCAGGAATAAGACTATT

AATTCGAGTTGAATTAACACAACCAGGATCATTTTTAGGAAGTGATCAATTATATAATAC

TATTGTTACGGCCCACGCCTTTATTATAATTTTCTTTATAGTTATACCTATATTTATTGG

TGGCTTTGGTAACTGAATATTACCATTAATATTAGGAGCACCAGATATAGCATTCCCACG

ACTTAACAACCTAAGATTTTGGTTACTCCCGCCTTCTTTAACTTTATTAGTAGCATCGGC

TGCAGTTGAAAAGGGAGCCGGCACAGGATGAACAGTTTATCCACCTCTATCAAGAAATTT

AGCTCATGCAGGTCCATCAGTAGACCTAGCAATCTTTTCACTACATCTAGCTGGGGCATC

CTCCATTCTAGGAGCTATTAACTTTATTACTACGGTAATTAATATACGATGAAATGGACT

CCGACTTGAACGAGTTCCATTATTTGTATGAGCAGTAACAATTACAGTTGTTCTATTACT

ATTATCGTTACCAGTACTAGCTGGGGCAATCACTATACTTCTTACAGATCGAAACTTAAA

TACAACATTTTTCGATCCGGCTGGTGGTGGAGACCCAGTATTATATCAACATTTATTT

>E8*_693_Cernosvitoviella_minor

AACACTTTACTTTATTTTAGGTGTATGAGCAGGAATACTAGGAGCTGCTATAAGACTTCT

AATTCGAATTGAACTGAGACAACCTGGCTCATTCCTAGGGAGAGATCAATTATATAATAC

TATCGTTACAGCACATGCATTTTTAATAATTTTCTTCTTAGTAATACCAGTATTTATTGG

AGGATTTGGGAACTGACTTTTACCTTTAATATTAGGAGCTCCAGACATGGCTTTTCCTCG

ATTAAACAACATAAGATTTTGACTCCTACCACCATCATTAATATTATTAGTATCCTCAGC

AGCCGTAGAAAAAGGAGCCGGCACTGGATGAACAGTATATCCACCTCTTTCTAGAAATTT

AGCACATGCTGGACCATCAGTAGATTTAGCAATTTTCTCTCTTCATCTTGCAGGAGCTTC

CTCAATTCTTGGGGCCGTAAACTTTATTACTACAGTAGTAAATATACGTTGACAAGGCCT

TCGACTAGAACGAATCCCCCTATTTGTATGAGCAGTAGTAATTACAGTAGTTCTACTACT

CTTATCTCTACCAGTACTAGCAGGAGCAATTACTATACTATTAACAGACCGAAATTTAAA

CACATCATTCTTTGACCCGGCAGGAGGGGGAGACCCTATTTTATACCAACACTTATTC

>E9*_694_Globulidrilus_riparius_3ind

CACTATATATTTTATCCTAGGTGTTTGAGCCGGAATACTAGGAGCAGCAATAAGACTATT

AATTCGAATTGAATTAAGTCAACCTGGTGCATTTCTTGGAAGAGACCAACTCTATAATAC

TATCGTAACAGCTCATGCATTTCTAATAATTTTCTTCTTAGTTATGCCTGTGTTCATTGG

TGGATTTGGAAACTGATTACTCCCTTTAATGCTAGGAGCACCAGATATAGCCTTTCCACG

TCTAAATAATATAAGATTTTGACTTCTTCCACCTTCACTACTTCTTCTTGTTTCATCTGC

AGCAGTAGAAAAAGGAGCTGGAACTGGTTGAACTGTATATCCCCCTCTATCAAGAAACAT

AGCTCACGCAGGACCATCCGTAGACCTAGCAATTTTTTCCCTTCATTTAGCAGGTGCATC

ATCAATTCTTGGAGCAGTAAATTTTATCACAACAGTAATTAACATACGATGACAAGGATT

GAGATTAGAGCGAATTCCTTTATTTGTATGAGCTGTAACAATCACTGTAGTTCTTCTTCT

TCTATCTCTCCCTGTTCTGGCTGGTGCAATCACCATATTATTAACTGATCGAAATCTAAA

CACTTCATTTTTTGACCCTGCTGGAGGAGGAGATNCAGTACTATATCAACACTTATTT

>E11*_697_Globulidrilus_riparius

CACTATATATTTTATTCTAGGTATTTGAGCAGGTATACTTGGAGCTGCTATAAGGCTTTT

AATTCGAATCGAATTAAGACAACCTGGATCCTTTCTTGGTAGAGATCAGCTTTATAATAC

TATTGTAACAGCTCATGCCTTTCTAATAATCTTCTTCTTAGTTATACCTGTATTTATTGG

GGGTTTCGGAAATTGATTATTACCATTAATACTAGGGGCTCCAGATATAGCATTTCCACG

TTTAAATAATATAAGATTTTGACTTCTACCTCCATCATTATTATTACTTGTATCATCCGC

TGCAGTTGAAAAAGGTGCAGGTACAGGATGAACAGTATATCCACCTCTTTCTAGAAATAT

GGCACATGCAGGACCTTCTGTAGATTTAGCTATTTTTTCTCTTCATCTAGCAGGTGCTTC

ATCAATTTTAGGAGCAGTAAACTTTATTACAACAGTAATTAATATACGATGACAAGGTCT

TAGATTAGAACGAATCCCTCTATTTGTATGGGCAGTAACCATTACTGTAGTACTTCTTCT

ATTATCCCTTCCAGTTTTAGCCGGCGCTATTACTATACTTTTAACTGATCGAAATTTAAA

TACATCATTTTTCGATCCTGCTGGAGGCGGGGACCCTGTTTTATATCAACACTTATTT

>E11*_701_Globulidrilus_riparius_2ind

CACTATATATTTTATTCTAGGCATTTGAGCAGGTATACTTGGTGCTGCTATAAGACTTTT

AATTCGAATTGAATTAAGTCAACCAGGATCTTTTCTTGGTAGAGACCAACTATATAATAC

TATTGTAACAGCCCATGCCTTCCTAATAATCTTTTTCCTAGTTATACCTGTATTTATTGG

GGGTTTTGGAAATTGACTACTCCCATTAATACTTGGGGCCCCAGATATAGCATTTCCACG

ACTAAATAATATAAGATTTTGACTTCTACCTCCTTCATTATTACTTCTTGTATCATCTGC

TGCTGTTGAAAAAGGCGCAGGAACAGGATGAACAGTATATCCTCCCCTTTCTAGAAATAT

AGCACATGCTGGACCCTCTGTAGACTTAGCCATTTTCTCCCTTCACTTAGCAGGTGCTTC

TTCAATTCTAGGAGCAGTAAATTTCATTACGACAGTAATTAATATACGATGACAAGGTCT

TAGATTAGAACGAATCCCTTTATTTGTATGAGCAGTAACAATCACTGTAGTACTTCTTCT

TCTATCTCTTCCAGTATTAGCTGGTGCCATTACTATACTTTTAACTGATCGAAATTTAAA

TACATCATTTTTTGATCCAGCTGGAGGAGGTGATCCCGTTTTATACCAACACTTATT-

>E11*_702_Globulidrilus_riparius_16ind

CACTATATATTTTATTTTAGGTATTTGAGCAGGTATACTTGGAGCAGCCATAAGACTCTT

AATTCGAATTGAATTAAGACAACCTGGATCTTTCCTTGGTAGAGATCAGTTATATAATAC

AATCGTAACAGCTCACGCCTTTCTAATAATTTTCTTCTTAGTTATACCTGTATTTATTGG

TGGTTTCGGAAATTGATTATTACCTTTAATACTTGGAGCCCCAGATATAGCATTTCCACG

ACTAAATAATATAAGATTTTGACTTCTACCCCCATCATTACTACTTCTTGTATCATCTGC

TGCAGTTGAAAAAGGTGCAGGAACAGGGTGAACAGTATACCCCCCTCTTTCCAGTAATAT

AGCTCATGCAGGGCCTTCTGTTGATTTAGCTATTTTTTCTCTACATTTAGCAGGTGCTTC

TTCAATCTTAGGGGCAGTAAATTTTATTACTACCGTAATTAATATACGATGACAAGGTCT

CAGACTAGAACGAATTCCACTATTTGTATGAGCAGTAACTATTACTGTAGTACTTCTCCT

ATTATCTCTCCCAGTTTTAGCAGGTGCCATTACTATACTTTTAACTGACCGAAATTTAAA

CACATCATTTTTCGATCCTGCTGGAGGGGGTGATCCTGTATTATATCAACATTTATTT

>E10*_703_Globulidrilus_riparius

CACAATATATTTTATTTTAGGTGTTTGAGCAGGTATACTAGGTGCAGCTATAAGATTACT

AATTCGTATTGAATTAAGCCAACCAGGATCATTTCTTGGTAGAGATCAATTATACAATAC

CATTGTAACCGCTCATGCCTTCTTAATAATCTTCTTCTTAGTAATACCTGTATTTATTGG

AGGATTTGGAAACTGACTACTTCCATTAATACTAGGTGCCCCTGACATAGCATTCCCGCG

ACTAAATAATATAAGATTCTGACTCCTTCCTCCATCACTATTACTACTAGTATCTTCAGC

CGCTGTTGAAAAAGGTGCAGGAACAGGATGAACTGTATACCCACCCCTTTCAAGAAATAT

AGCTCATGCAGGCCCATCTGTAGATCTAGCTATTTTCTCTCTTCATTTAGCTGGTGCGTC

TTCAATTCTAGGTGCTGTAAATTTCATCACTACAGTAATTAATATACGATGACAAGGTCT

TAGATTAGAACGAATCCCTTTATTTGTTTGAGCAGTAACTATTACTGTAGTTCTTCTTCT

ACTATCTCTACCAGTTTTAGCAGGAGCCATTACTATACTTCTAACTGACCGTAATTTAAA

CACCTCATTCTTTGATCCTGCTGGAGGTGGAG--------------------------

>E9*_706_Globulidrilus_riparius

CACTATATATTTTATCCTAGGTGTTTGAGCCGGAATACTAGGAGCAGCAATAAGACTATT

AATTCGAATTGAATTAAGTCAACCTGGTGCATTCCTTGGAAGAGATCAACTCTATAATAC

TATCGTAACAGCTCATGCATTTCTAATAATTTTCTTCTTAGTTATGCCTGTATTCATTGG

TGGATTTGGAAACTGATTACTGCCTTTAATACTAGGGGCACCAGATATAGCCTTTCCACG

TCTAAATAACATAAGATTTTGACTTCTTCCACCTTCACTACTTCTTCTTGTTTCATCTGC

AGCAGTAGAAAAAGGAGCTGGAACTGGTTGAACCGTATATCCTCCTCTATCAAGAAACAT

AGCTCATGCAGGACCATCTGTAGATCTAGCAATTTTTTCTCTTCATTTAGCAGGTGCATC

ATCAATTCTTGGAGCAGTAAATTTTATCACAACAGTAATTAACATACGATGACAAGGATT

AAGATTAGAACGAATTCCTTTATTTGTATGAGCTGTAACAATCACTGTAGTTCTTCTTCT

TCTATCTCTCCCTGTTCTGGCTGGTGCAATCACCATATTATTAACTGATCGAAATCTAAA

CACTTCATTTTTTGATCCTGCTGGTGGAGGAGA-------------------------

>LL3_682_Lumbriculidae_sp

GACCTTATATTTCATCTTAGGAGTTTGAGCTGGAATAGTGGGAGCAGGCATAAGACTATT

AATTCGAGTTGAATTAACACAACCTGGGTCATTTTTAGGAAGTGACCAACTATACAATAC

TATTGTTACGGCTCACGCCTTTATTATAATTTTCTTTATAGTCATACCTATATTTATTGG

TGGTTTTGGTAACTGAATACTACCATTAATATTAGGAGCACCAGATATAGCATTCCCACG

GCTTAACAACCTAAGATTTTGGTTACTTCCACCATCTTTAACTTTATTAGTAGCATCCGC

TGCAGTTGAAAAAGGAGCAGGTACAGGATGAACAGTTTACCCGCCTCTATCAAGAAATTT

AGCCCATGCAGGTCCATCAGTAGATCTAGCAATCTTTTCACTTCATCTAGCCGGAGCATC

CTCCATTCTAGGAGCTATTAACTTTATTACAACAGTAATTAATATACGATGAAATGGACT

ACGACTTGAACGAGTTCCATTATTTGTATGAGCAGTAACAATTACAGTAGTACTCTTACT

ATTATCTTTACCAGTACTAGCTGGGGCAATCACTATACTTCTTACAGATCGAAACTTAAA

TACAACATTTTTTGATCCAGCGGGAGGTGGAGACCCAGTACTA---------------

>LC4_LN810242_Helodrilus_oculatus

AACCTTATATTTTATCCTTGGCGTTTGAGCCGGCATAGTAGGAGCTGGCATAAGCCTTCT

CATTCGAATTGAACTAAGACAACCGGGAGCCTTCCTGGGAAGAGATCAACTTTACAATAC

AATTGTTACAGCTCATGCATTCGTAATAATCTTCTTCCTTGTTATGCCCGTATTTATTGG

AGGATTTGGAAATTGACTTCTTCCATTAATACTGGGTGCCCCTGATATGGCGTTCCCCCG

ACTAAATAACATAAGATTTTGACTACTTCCCCCCTCATTAATCCTCTTAGTTTCCTCTGC

AGCAGTAGAGAAAGGAGCAGGAACCGGCTGAACAGTGTATCCACCTTTAGCCAGAAATCT

TGCCCACGCAGGTCCCTCAGTAGATTTAGCCATTTTTTCTCTACACTTAGCAGGGGCATC

TTCAATTCTCGGGGCCATCAATTTTATTACAACAGTTATCAACATACGATGAAGAGGTCT

ACGTCTAGAACGTATTCCTTTATTCATCTGAGCCGTTGTAATTACGGTAGTACTCCTACT

CCTATCTCTTCCAGTGCTGGCAGGGGCAATTACTATGCTCCTCACAGACCGAAACCTTAA

TACCTCTTTCTTTGACCCTGCTGGAGGGGG-----------------------------

>LC1_LN810243_Dendrodrilus_rubidus

AACACTATATTTCATTTTAGGCGTCTGAGCTGGCATAGTCGGCGCTGGAATAAGACTTCT

AATTCGAATTGAACTAAGACAGCCGGGAGCCTTTCTAGGAAGAGATCAACTTTATAATAC

AATTGTAACAGCCCATGCATTTGTAATAATCTTCTTTCTAGTTATGCCAGTATTTATTGG

GGGGTTTGGAAATTGACTTCTCCCTTTAATACTAGGTGCTCCTGATATAGCATTCCCCCG

TCTAAATAACATGAGATTCTGACTACTACCCCCTGCACTTATTCTCTTAGTATCTTCTGC

TGCAGTAGAAAAGGGGGCTGGAACTGGATGAACTGTTTACCCCCCCTTATCTAGAAACAT

TGCGCATGCTGGGCCCTCTGTGGATCTAGCAATTTTCTCTCTTCATTTAGCTGGGGCATC

CTCAATTCTCGGTGCCATTAACTTTATTACAACAGTTATTAATATACGCTGAAGGGGTCT

ACGTCTAGAGCGTATTCCATTATTTGTATGAGCAGTCTTAATTACTGTAGTATTACTTCT

TCTCTCTTTACCAGTTCTTGCTGGCGCAATTACTATACTTCTCACAGACCGAAACCTAAA

CACCTCATTCTT----------------------------------------------

>E4_LN810244_Marionina_argentea

TCATTATACTTTTATTCTAGGAACTTGAGCAGGGATACTAGGAGCAGCTATAAGCCTCTT

AATTCGATTTGAACTAAGACAACCTGGATCGTTTCTAGGTAGAGATCAACTCTACAACAC

TATCGTTACAGCTCATGCATTCTTAATAATCTTTTTCTTAGTTATACCAGTATTTATTGG

TGGATTTGGAAACTGACTCTTACCCCTTATATTAGGAGCCCCTGACATAGCCTTCCCCCG

CCTTAATAATTTAAGATTTTGATTACTTCCTCCATCTCTCCTACTTTTAGTCTCATCTGC

CGCAGTTGAAAAAGGCGCAGGCACTGGATGAACAGTATACCCGCCCTG-GCCTCCAATAT

TGCCCATTCAGGACCTTCTGTTGATTTAGCCATCTTCTCCCTTCATCTAGCAGGTGCGTC

CTCCATCTTAGGTGCAATTAATTTCATCACAACTGTTATCAATATACGCTGACAAGGCCT

CCAACTAGAACGAATTCCTCTATTTGTCTGAGCCGTTACAATTACAGTAGTTCTTCTTCT

TTTATCACTTCCAGTACTAGCAGGGGCTATTACCATACTACTAACAGATCGAAACCTAAA

CACTTCCTTCTTTGACCCTGCAGGAGGGGGGGACCCTATTTTATATCAACACTTATTT

>E3_LN810245_Lumbricillus_rutilus

-ACACTATATTTTATTTTAGGAGTATGAGCTGGAATACTAGGAGCAGCCATAAGACTTTT

AATTCGAATTGAATTAAGACAACCTGGCGCTTTTTTAGGAAGAGATCAGCTTTATAATAC

TATCGTAACAGCTCATGCATTCTTAATAATTTTTTTCTTAGTTATACCAGTATTTATTGG

TGGATTTGGAAATTGATTAATTCCGCTAATATTGGGAGCTCCTGACATAGCATTCCCTCG

TCTTAACAATATAAGATTTTGACTTCTACCTCCAGCTCTTTTACTTCTAGTTTCTTCAGC

AGCAGTAGAAAAAGGTGCTGGGACTGGCTGAACAGTTTACCCACCTCTAGCAAGAAATCT

AGCTCATGCAGGTCCATCCGTAGATTTAGCAATTTTCTCTCTTCATTTAGCCGGTGCCTC

ATCTATTCTTGGAGCAGTAAACTTTATTACTACAGTAGTAAATATACGTTGACAAGGTCT

TCGACTTGAACGAATTCCTCTTTTTGTATGAGCAGTAGTAATTACAACAGTTCTTCTTCT

TCTATCTCTTCCAGTTCTTGCAGGGGCAATTACAATACTACTAACTGATCGAAATCTAAA

CACTTCATTTTTTGACCCAGCCGGAGGTGGAGATCCTGTTCTTTATCAACATTTATTT

>E1_LN810247_Enchytraeus_buchholzi

------------------------------------------------------------

-----------------------------CATTTCTAGGAAGAGATCAGTTATATAATAC

AATTGTAACTGCCCATGCATTCCTAATAATTTTCTTTCTAGTAATACCTGTATTTATCGG

GGGATTTGGAAATTGACTATTACCACTAATACTAGGTGCCCCAGATATAGCTTTTCCGCG

ATTAAATAACATAAGATTCTGCATTCTACCTCCAGCACTAATACTACTTCTATCCTCAGC

AGCAGTAGAAAAGGGGGCTGGAACAGGGGGAACAGTTTATCCTCCTTTAGCTAGAAATAT

TGCTCACGCTGGACCATCTGTAGACTTAGCAATTTTCTCTCTACATTTAGCTGGAGCATC

ATCAATTCTAGGAGCTGTTAATTTCATCACTACAGTAATTAACATACGATGACAAGGACT

AACCCTAGAACGAATTCCTTTATTTGTATGAGCCGTAACTATCACAGTAGTTCTTCTTCT

TTTATCTCTTCCAGTATTAGCAGGGGCTATCACTATATTATTAACCGATCGTAATTTAAA

TACCTCATTCTTCGACCCTGCAGGCGGAGGAGATCCTATTCTATATCAACACTTATTC

>E2_LN810248_Fridericia_sp

-ACACTATCCTTCATCCTTGGTGTATGAGCTGGCATAATGGGAGCAGCAATAAGCCTCCT

AATTCGAATTGAACTAAGTCAACCAGGATCATTCTTAGGAAGAGATCAACTATATAACAC

TATCGTAACAGCTCACGCATTCCTAATAATTTTCTTCCTAGTGATACCAGTATTTATTGG

TGGCTTCGGCAACTGACTTCTTCCATTAATACTTGGTGCACCAGATATAGCATTTCCTCG

ACTTAACAATATAAGATTTTGACTTCTACCACCTTCCCTAATACTTCTTCTTTCATCAGC

AGCTGTAGAAAAGGGTGCCGGCACAGGTTGAACAGTTTACCCACCACTAGCTAGAAACAT

AGCACACGCTGGCCCATCCGTAGACTTAGCTATTTTCTCACTACACTTAGCAGGAGCATC

ATCTATTTTAGGAGCAGTAAACTTTATCTCAACTGTAATTAACATACGATGACAAGGCCT

TCAATTAGAACGAATTCCTCTATTTGTGTGAGCTGTCACCATTACTGTAGTACTACTACT

TCTCTCTCTTCCAGTTTTAGCAGGAGCCATTACTATATTATTAACAGATCGAAACTTAAA

CACCTCATTCTTCGATCCTGCCGGTGGAGGAGATCCTATTCTATATCAACACTTATTC

>LC3_LN810249_Eiseniella_tertraedra

------------CATTTTAGGTATTTGAGCTGGGATAGTGGGAGCTGGTATAAGCTTACT

AATTCGAATCGAATTAAGCCAACCAGGAGCCTTCCTAGGTAGTGACCAATTATATAACAC

TATTGTTACAGCACATGCATTCGTAATAATCTTCTTCCTAGTAATACCCGTATTCATTGG

TGGATTCGGAAACTGATTGCTACCTCTAATACTAGGTGCACCAGATATAGCATTTCCACG

TTTAAACAACATAAGATTTTGACTTTTACCTCCTTCTTTAATTCTACTAGTATCTTCCGC

AGCCGTAGAAAAGGGGGCCGGGACAGGGTGAACTGTTTATCCGCCCTTAGCAAGAAACTT

GGCCCATGCAGGACCATCAGTAGATCTAGCTATTTTCTCCTTACACTTAGCAGGAGCTTC

ATCTATTTTAGGTGCCATCAACTTTATTACTACAGTCATTAATATGCGATGAAGGGGCTT

ACGATTAGAACGAATTCCCCTTTTCGTGTGAGCTGTACTGATTACAGTAATTCTTCTATT

ATTATCGTTGCCCGTGCTAGCAGGAGCAATTACCATACTATTAACCGACCGAAATCTCAA

TACATCATTCTTTGACCCGGCTGGTGGTGGTGACCCAATTCTGTACCAACACCTCTTC

>LC2_LN810251_Eiseniella_tertraedra

-ACCCTTTATTTCACTTTAGGTATCTGAGCTGGCATGGTGGGAGCTGGAATAAGCTTACT

AATTCGAATCGAATTAAGACAACCTGGAGCCTTCTTAGGTAGCGACCAATTATACAATAC

TATTGTTACAGCACATGCATTTGTAATAATCTTCTTCCTGGTAATGCCCGTATTCATTGG

TGGATTCGGAAACTGACTGCTACCTCTAATACTAGGTGCACCAGATATAGCATTTCCACG

TTTAAATAACATGAGATTTTGACTTCTGCCTCCTTCCTTAATTCTACTAGTATCCTCTGC

AGCCGTAGAAAAGGGGGCCGGGACAGGATGAACTGTTTATCCTCCCTTAGCAAGAAATTT

AGCTCATGCAGGTCCATCAGTAGACTTAGCTATTTTCTCATTACATCTAGCAGGAGCTTC

ATCTATTTTAGGGGCCATCAACTTTATTACTACAGTCATCAATATGCGATGAAGAGGTTT

ACGATTAGAGCGAATCCCCCTTTTCGTATGAGCTGTATTAATTACAGTGATTCTTCTTCT

ACTATCATTGCCCGTGCTAGCAGGAGCAATCACCATATTACTAACCGACCGAAATCTAAA

TACATCATTCTTCGATCCGGCTGGTGGCGGTGATCCAATTCTATATCAACACCTCTTC

>N3_LN810253_Nais_communis

TACATTATATTTAATTTTAGGAGTATGAGCAGGAATGGTTGGAACAGGAACAAGACTATT

AATTCGAATTGAACTTGCCCAACCAGGATCTTTCCTAGGAAGAGATCAATTATATAACAC

TCTTGTGACTGCACATGCATTTTTAATAATTTTCTTCTTAGTTATGCCAGTATTTATTGG

TGGTTTTGGAAACTGACTACTACCTCTAATATTAGGAGCACCTGACATAGCATTTCCACG

ATTAAATAACCTTAGATTTTGATTACTACCACCTTCACTAATTCTATTAGTATCATCTGC

CGCTGTAGAAAAAGGAGCCGGAACAGGATGAACTGTATATCCGCCACTATCAAGAAACTT

AGCACATGCAGGACCATCAGTTGACATGGCTATTTTCTCATTACACTTAGCAGGTGCATC

ATCTATTTTAGGTGCAGTAAACTTTATTACAACAGTAATAAATATACGATGAAATGGAAT

ACGACTAGAACGAGTCCCATTATTTGTATGAGCAGTTCTACTTACCGTAATTCTACTTCT

ACTATCATTACCAGTACTTGCAGGAGCAATTACAATACTACTAACAGATCGAAATCTAAA

TACTTCATTCTTCGATCCAGCAGGAGGGGGAGATCCAATTCTATACCAACATTTATTT

>N6_LN810254_Piguetiella_blanci

TACATTATATTTAATTTTAGGAGTATGAGCAGGAATAGTTGGAACAGGAACAAGAATATT

AATTCGAATTGAACTAGCTCAACCAGGATCATTCCTAGGAAGAGATCAGCTATACAATAC

TCTTGTAACAGCACATGCATTCTTAATAATTTTCTTTTTAGTTATACCTGTATTCATTGG

TGGGTTTGGAAACTGACTACTTCCATTAATACTTGGTGCCCCAGATATGGCATTTCCACG

ACTAAACAACTTAAGATTCTGATTATTACCACCTTCACTAATTCTTCTAGTCTCATCAGC

TGCAGTAGAAAAAGGAGCAGGAACAGGCTGAACTGTATATCCACCACTATCTAGAAATCT

AGCACACGCTGGCCCATCAGTTGACATGGCTATTTTCTCTCTTCATTTAGCAGGTGCATC

CTCTATTTTAGGTGCAGTTAATTTTATTACAACAGTAATAAACATACGATGAAATGGAAT

ACGATTAGAACGAGTACCGTTATTTGTATGAGCTGTAACGCTTACCGTTATTCTTCTTCT

ACTATCACTGCCGGTACTTGCAGGTGCAATTACAATACTACTAACAGATCGAAATCTAAA

TACTTCATTCTTCGATCCTGCTGGTGGTGGAGATCCAATTCTATATCAACATTTATTC

>N5_LN810257_Ophidonais_serpentina

TACATTATATTTAATCTTAGGAGTATGAGCAGGAATAGTTGGTACAGGAACAAGAATACT

GATTCGAATTGAACTAGCTCAACCAGGAGCTTTTCTAGGAAGAGATCAATTATATAACAC

TCTAGTAACAGCACATGCGTTTTTAATAATTTTCTTTTTAGTTATACCTGTATTTATTGG

CGGATTCGGAAACTGACTTCTTCCATTAATATTAGGTGCTCCAGATATGGCATTCCCACG

ACTAAATAATCTTAGATTCTGACTTCTACCACCATCATTAATTCTATTAATTTCATCTGC

AGCCGTTGAAAAAGGTGCTGGAACAGGATGAACTGTATATCCTCCATTATCAAGAAATCT

AGCTCACGCTGGACCTTCAGTTGACATGGCTATTTTTTCACTACATCTAGCAGGTGCATC

TTCTATTTTAGGTGCAGTTAACTTCATTACTACAGTAATAAACATACGATGAAATGGAAT

ACGACTTGAACGAGTACCATTATTTGTATGAGCTGTAACACTTACTGTAATTCTTCTTCT

TTTATCATTACCTGTATTAGCTGGTGCAATTACCATACTATTAACAGATCGAAATCTAAA

TACCTCATTCTTCGATCCTGCAGGAGGGGGAGACCCAATTTTATACCAACATTTATTC

>N4_LN810258_Nais_elinguis

-ACATTATACTTAATTCTAGGAGTATGAGCGGGAATAGTTGGAACTGGAACAAGAATACT

AATTCGAATTGAACTTGCTCAACCAGGAGCCTTCCTCGGAAGAGACCAACTCTATAACAC

CCTAGTTACAGCTCATGCATTTTTAATAATTTTCTTTCTTGTAATGCCAGTATTTATTGG

TGGATTCGGAAATTGACTTCTACCACTAATACTTGGAGCCCCAGATATAGCATTTCCACG

ATTAAATAATCTAAGATTTTGATTACTACCACCATCATTAATTCTACTAATTTCATCAGC

CGCAGTTGAAAAAGGTGCAGGAACAGGATGAACTGTTTACCCTCCATTATCAAGAAATTT

AGCTCATGCTGGACCATCAGTTGACATGGCTATTTTTTCTCTTCATTTAGCAGGTGCATC

ATCTATTTTAGGTGCAGTAAATTTTATTACTACAGTAATAAATATACGATGAAACGGTAT

ACGATTAGAACGTTTACCTTTATTTGTATGAGCTGTATTCCTTACTGTAATTCTCCTTCT

ACTATCGCTACCAGTATTAGCTGGCGCTATTACAATACTTCTTACAGACCGTAATTTAAA

TACCTCATTCTTTGATCCAGCAGGAGGAGGAGATCCGATTCTTTATCAACACTTATTT

>N2_LN810267_Nais_bretscheri

TACACTATATTTAATTTTAGGAGTATGAGCAGGAATAGTAGGAACTGGAACAAGACTACT

TATTCGAATTGAACTATCACAACCAGGATCATTTCTTGGAAGAGACCAATTATATAATAC

TCTTGTAACAGCACATGCATTCTTAATAATTTTCTTCTTAGTAATACCTGTATTTATTGG

GGGATTTGGAAATTGACTTCTCCCACTAATACTAGGTGCTGCTGATATAGCATTTCCACG

ACTAAATAATCTTAGATTTTGATTACTACCACCATCATTAATTCTATTAGTTTCTTCTGC

AGCTGTAGAAAAAGGAGCTGGAACAGGATGAACAGTATACCCACCACTATCAAGGAATCT

AGCTCATGCCGGACCATCTGTAGACATGGCTATCTTTTCACTTCACTTGGCAGGTGCCTC

TTCTATTTTAGGGGCAGTAAATTTTATTACAACTGTAATAAATATACGTTGAAATGGTAT

ACGATTAGAACGACTACCATTATTTGTATGAGCTGTATTTCTTACAGTAATCCTTCTGCT

ACTTTCTCTTCCAGTTCTTGCTGGAGCTATTACCATACTACTAACAGACCGAAACTTAAA

TACTTCATTCTTTGATCCTGCTGGAGGAGGAGACCCAATTCTATATCAACATCTATTC

>N1_LN810268_Chaetogaster_diaphanus

CACTCTATATTTAATTTTAGGGGTATGAGCAGGAATAATTGGTACAGGAACTAGAATACT

AATTCGAATTGAACTATCTCAGCCTGGGTCATTTCTAGGAAGAGATCAACTATATAATAC

TTTAGTTACTGCACATGCATTCTTGATAATTTTCTTTTTAGTAATACCTGTATTTATTGG

GGGATTTGGAAATTGACTTCTACCATTAATACTAGGTGCACCAGATATAGCTTTTCCACG

TTTAAATAATTTAAGATTTTGATTATTACCTCCTTCATTAATTTTATTAATTTCATCTGC

TGCTGTAGAAAAAGGAGCTGGAACAGGATGAACAGTATACCCTCCACTATCAAGAAATCT

TGCCCATGCGGGACCATCAGTAGATATAGCAATTTTCTCTCTTCACTTAGCAGGGGCTTC

ATCTATTTTAGGAGCAGTTAACTTTATTGCAACAACAATTAATATGCGATGAAACGGAAT

ACGGCTAGAACGACTACCTTTATTTGTATGGGCAGTTCTACTAACCGTTATTCTTCTTCT

ATTATCACTTCCAGTGCTTGCTGGGGCTATTACAATGTTACTTACAGATCGAAACCTTAA

TACTTCTTTCTTTGATCCGGCTGGTGGTGGAGATCCTATTTTATATCAACATCTATTC

>LL2_LN810269_Lumbriculus_variegatus

-ACTCTATATTTTATTCTTGGCGTCTGAGCCGGCATAGTAGGAGCAGGAATAAGACTACT

AATCCGAATTGAGCTCACACAACCAGGATCATTTCTAGGCAGAGATCAACTATACAATAC

CATAGTTACCGCACATGCCTTTATTATAATTTTCTTTATAGTAATACCTATATTTATTGG

CGGATTCGGAAATTGATTACTACCATTAATACTAGGTGCTCCAGACATAGCATTTCCACG

ACTAAATAATCTTAGTTTTTGACTACTACCCCCTTCCCTAATTTTATTAGTAAGATCAGC

TGCAGTAGAAAAAGGAGCAGGAACTGGATGAACTGTATACCCACCTCTAGCAAGTAATCT

AGCACACGCTGGACCTTCTGTAGATCTAGCTATCTTCTCCCTTCATTTAGCCGGAGCATC

CTCAATTCTAGGAGCCCTAAACTTTATTACAACTGTTATCAATATACGATGAAATGGTAT

ACGTCTAGAACGAATTCCTTTATTTGTATGAGCCGTAATAATTACAGTAATTTTACTCCT

ATTATCCTTACCGGTATTAGCAGGAGCCATTACTATATTACTAACAGATCGAAACCTAAA

CACCTCCTTCTTTGATCCAGCTGGGGGTGGAGACCCTGTCCTATATCAACATCTATTT

>LL1_LN810271_Lumbriculidae_sp

GACTCTATACTTCATTTTAGGAGTTTGAGCAGGAATAGTAGGAGCTGGTATAAGCCTTTT

AATTCGAATTGAATTGACCCAGCCAGGGTCATTCCTAGGAAGAGATCAACTATATAATAC

TATAGTAACAGCTCATGCTTTTATCATAATTTTTTTTATAGTAATACCCATATTTATTGG

GGGGTTTGGAAATTGAATACTCCCATTAATACTAGGAGCACCAGATATAGCTTTCCCTCG

ATTAAATAATCTAAGTTTTTGGTTACTTCCTCCATCACTTATTCTACTAGTTTCTTCTGC

TGCAGTAGAAAAAGGTGCTGGTACTGGATGAACAGTATACCCCCCATTAGCAAGAAATCT

TGCACATAGAGGGCCTTCAGTTGATTTAGCAATCTTTTCATTACATCTTGCTGGTGCCTC

GTCTATTTTAGGAGCTTTAAATTTTATTACTACCGTAGTAAATATGCGATGAAACGGGCT

ACGATTAGAGCGAATTCCTTTATTCGTTTGAGCTGTGACAATTACAGTGGTTCTATTACT

TTTGTCACTTCCTGTACTTGCAGGCGCAATTACAATACTTCTTACTGATCGAAACTTAAA

TACTTCTTTTTTTGACCCAGCAGGGGGTGGAGACCCAGTTCTATACCAGCATTTATTC

>LL3_LN810273_Stylodrilus_heringianus

AACCTTATATTTCATCTTAGGAGTTTGAGCTGGGATAGTAGGAGCAGGGATGAGACTATT

AATTCGAGTTGAATTAACACAACCCGGGTCATTTTTAGGAAGTGACCAACTATATAATAC

TATTGTTACGGCTCACGCATTTATTATAATTTTCTTTATAGTTATACCTATATTTATTGG

TGGTTTTGGTAACTGAATACTACCGTTAATATTAGGAGCACCAGACATAGCATTTCCACG

ACTTAACAACCTAAGATTTTGATTACTACCACCTTCTTTAACTTTATTAGTAGCATCGGC

TGCAGTAGAAAAAGGAGCCGGCACAGGATGAACAGTTTATCCACCTCTATCAAGAAATTT

AGCTCATGCTGGACCCTCAGTAGACCTGGCAATCTTCTCACTCCATCTAGCAGGAGCCTC

CTCCATTTTAGGAGCCATTAACTTTATTACGACTGTAATTAATATACGATGAAATGGACT

ACGACTTGAACGAGTTCCATTATTTGTATGAGCAGTAACAATTACAGTAGTTCTATTACT

ATTATCATTACCAGTACTAGCTGGAGCAATCACTATACTTCTTACAGATCGAAACTTAAA

TACAACATTTTTTGATCCAGCTGGAGGTGGAGACCCCGTACTATATCAACATTTATTT

>LL3_LN810275_Stylodrilus_heringianus

---CTTATATTTCATCTTAGGAGTTTGAGCTGGAATAGTAGGAGCAGGAATAAGACTATT

AATTCGAGTTGAATTAACACAACCAGGATCATTTTTAGGAAGTGATCAATTATATAATAC

TATTGTTACGGCTCACGCCTTTATTATAATTTTCTTTATAGTTATACCTATATTTATTGG

TGGCTTTGGTAACTGAATATTACCATTAATATTAGGAGCACCAGATATGGCATTCCCACG

ACTTAACAACCTAAGATTTTGGTTACTCCCGCCTTCTTTAACTTTATTAGTAGCATCGGC

TGCAGTTGAAAAGGGAGCCGGCACAGGATGAACAGTTTATCCACCTCTATCAAGAAATTT

AGCTCATGCAGGTCCATCAGTAGACCTAGCAATCTTTTCACTACATCTAGCTGGGGCATC

CTCCATTCTAGGAGCTATTAACTTTATTACTACGGTAATTAATATACGATGAAATGGACT

CCGACTTGAACGAGTTCCATTATTTGTATGAGCAGTAACAATTACAGTTGTTCTATTACT

ATTATCATTACCAGTACTAGCTGGGGCAATCACTATACTTCTTACAGATCGAAACTTAAA

TACAACATTTTTCGATCCGGCTGGTGGTGGAGACCCAGTATTATATCAACATTTATTT

>R1_LN810295_Bothrioneurum_vejdovskyanum

CACTCTATACCTAATCTTCGGCGTATGAGCTGGAATGGTAGGAACTGGAACAAGACTCCT

AATTCGAATCGAACTAGCTCAACCAGGATCATTCCTAGGCAGAGACCAGCTATTCAACAC

TCTGGTCACAGCCCATGCATTCCTGATAATCTTCTTCTTTGTAATACCAGTATTCATTGG

CGGCTTTGGCAACTACTTAATCCCACTAATACTAGGCGCACCAGACATGGCGTTCCCACG

ACTCAACAACATAAGATTCTGACTCCTACCCCCATCCCTAATTCTCCTAGTATCATCCGC

AGCAGTAGAAAAAGGAGCCGGTACAGGATGAACAGTGTATCCACCCCTAGCAAGAAACCT

TGCACACTCCGGCCCATCAGTAGATCTAGCAATTTTCTCCCTCCACTTAGCCGGGGCATC

CTCAATCCTAGGCGCTATTAACTTCATCACCACAATAATCAACATACGCTGAAACGGACT

TCGACTTGAACGAATCCCCCTATTCGTCTGAGCAGCAGTAATTACAGTAATCCTACTCCT

ACTATCACTTCCAGTACTAGCTGGGGCTATTACCATACTATTAACAGACCGCAACCTAAA

TACATCCTTCTTTGACCCAGCTGGAGGGGGCGACCCTATTCTATACCAACATCTATTC

>R1_LN810296_Bothrioneurum_vejdovskyanum

CACCCTGTACCTAATCTTCGGCGTATGAGCCGGAATAGTGGGAACTGGAACAAGACTCCT

AATTCGAATCGAATTAGCTCAACCAGGATCATTCCTAGGCAGAGACCAACTATTCAACAC

CCTAGTCACAGCCCATGCATTCCTAATAATCTTCTTCTTTGTAATACCAGTATTCATCGG

CGGCTTTGGCAACTACTTAATCCCGCTAATACTAGGCGCACCAGACATAGCATTCCCACG

ACTAAACAACATAAGATTCTGACTCCTACCCCCATCCCTAATTCTCCTAGTATCATCCGC

AGCAGTAGAAAAAGGAGCCGGTACAGGATGAACAGTATATCCACCCCTAGCAAGAAATCT

CGCACACTCCGGTCCATCAGTAGACCTGGCAATTTTCTCCCTTCATTTAGCCGGAGCATC

CTCAATCCTAGGTGCTATCAACTTCATCACCACAATAATCAACATACGCTGAAACGGACT

TCGACTTGAACGAATCCCTCTATTCGTCTGAGCAGCAGTAATCACAGTAATCCTACTCCT

ACTATCACTTCCAGTACTAGCTGGGGCCATTACCATACTATTAACAGACCGCAACCTAAA

TACATCCTTCTTCGACCCAGCTGGTGGGGGCGACCCTATTCTATACCAACATCTATTC

>N8*_960_Nais_alpina_2ind

TACACTATATTTAATTTTAGGAGTATGAGCAGGAATAGTGGGAACTGGAACAAGATTACT

TATTCGAATTGAACTATCACAACCAGGATCATTTCTTGGAAGAGATCAACTATACAACAC

TCTTGTAACAGCACATGCATTCTTAATAATTTTCTTCTTGGTAATACCAGTATTCATTGG

GGGGTTTGGAAACTGACTTCTTCCATTAATACTAGGTGCTGCCGATATAGCATTTCCACG

ACTTAATAATCTTAGATTTTGATTGCTACCACCATCATTAATTTTATTAATTTCTTCTGC

AGCTGTAGAAAAAGGTGCTGGAACAGGATGAACTGTTTATCCGCCTCTATCAAGAAATCT

AGCACATGCCGGACCTTCTGTAGATATGGCTATTTTTTCACTTCATTTAGCAGGTGCTTC

ATCTATTTTAGGAGCAGTAAATTTTATTACTACAGTAATAAATATACGATGAAATGGAAT

ACGACTAGAACGGCTACCATTATTTGTTTGAGCAGTATTTCTTACAGTAATTCTTCTTTT

ATTATCTCTTCCAGTACTTGCTGGGGCAATTACAATACTATTAACAGATCGAAATCTTAA

TACTTCATTTTTTGATCCTGCTGGAGGTGGGGATCCAATCTTATATCAACATCTATTT

>LC3_961_Eiseniella_tetraedra

AACCCTTTACTTCATTTTAGGTATTTGAGCTGGGATGGTAGGAGCTGGAATAAGCTTACT

AATTCGAATCGAATTAAGCCAACCTGGAGCCTTTTTAGGTAGTGACCAACTATACAACAC

TATTGTCACAGCACACGCATTCGTAATAATCTTTTTCCTAGTAATACCAGTATTCATTGG

TGGATTCGGAAACTGGTTATTACCTTTAATACTTGGTGCGCCAGATATAGCATTTCCACG

TTTAAATAACATAAGATTTTGACTTTTACCTCCTTCTCTAATTCTATTAGTATCTTCCGC

CGCCGTAGAAAAGGGTGCCGGGACAGGTTGAACTGTTTACCCACCCTTAGCAAGAAACTT

AGCTCATGCAGGACCATCCGTAGATCTAGCTATTTTCTCCTTACATTTAGCGGGAGCTTC

ATCTATTTTAGGGGCCATCAACTTTATTACCACAGTCATTAATATACGATGAAGGGGCTT

ACGATTAGAACGAATCCCCCTTTTCGTCTGAGCTGTGCTAATTACAGTGATTCTTCTACT

ACTATCATTGCCCGTGCTTGCAGGAGCAATTACCATGTTATTAACTGACCGAAATCTCAA

CACATCTTTTTTTGATCCGGCTGGTGGTGGTGACCCAATTCTATATCAACACCTCTTC

>LL3_957_Stylodrilus_heringianus

AACCTTATATTTCATCTTAGGAGTTTGAGCTGGGATAGTAGGAGCAGGGATGAGACTATT

AATTCGAGTTGAATTAACACAACCCGGGTCATTTTTAGGAAGTGACCAACTATATAATAC

TATTGTTACGGCTCACGCATTTATTATAATTTTCTTTATAGTTATACCTATATTTATTGG

TGGTTTTGGTAACTGAATACTACCGTTAATATTAGGAGCACCAGACATAGCATTTCCACG

ACTTAACAACCTAAGATTTTGATTACTACCACCTTCTTTAACTTTATTAGTAGCATCGGC

TGCAGTAGAAAAAGGAGCCGGCACAGGATGAACAGTTTATCCACCTCTATCAAGAAATTT

AGCTCATGCTGGACCCTCAGTAGACCTGGCAATCTTCTCACTCCATCTAGCAGGAGCCTC

CTCCATTTTAGGAGCCATTAACTTTATTACGACTGTAATTAATATACGATGAAATGGACT

ACGACTTGAACGAGTTCCATTATTTGTATGAGCAGTAACAATTACAGTAGTTCTATTACT

ATTATCATTACCAGTACTAGCTGGAGCAATCACTATACTTCTTACAGATCGAAACTTAAA

TACAACATTTTTTGATCCAGCTGGAGGTGGAGACCCCGTACTATATCAACATTTATTT

>N10*_965_Nais_communis

---------------TTTAGGAGTATGAGCAGGAATAGTAGGAACTGGAACTAGATTACT

TATTCGAATTGAATTATCACAACCAGGATCATTTCTTGGAAGAGATCAATTATATAATAC

TCTTGTAACAGCACACGCGTTCTTAATAATTTTCTTCTTAGTAATACCAGTATTTATTGG

GGGGTTCGGAAACTGACTTCTCCCACTAATACTAGGTGCTGCTGATATAGCATTCCCACG

ACTAAACAATCTTAGATTTTGACTACTACCACCATCATTAATTCTATTAATTTCTTCTGC

TGCTGTAGAAAAAGGTGCAGGAACAGGATGAACTGTTTATCCGCCTCTATCAAGAAATCT

AGCACACGCTGGACCTTCAGTAGACATGGCCATTTTCTCACTTCACTTAGCAGGTGCTTC

TTCTATTTTAGGGGCAGTAAATTTCATTACAACAGTAATAAATATACGATGAAACGGAAT

ACGATTAGAACGACTTCCACTATTCGTATGAGCAGTATTTCTTACAGTAATTCTCCTTCT

TCTATCACTTCCCGTTCTTGCTGGTGCAATTACAATACTATTAACAGATCGAAATCTTAA

TACCTCATTCTTCGATCCTGCTGGTGGTGGAGATCCGATCTTATATCAACACTTATTC

>N5_966_Ophidonais_serpentina_5ind

------------------AGGAGTATGAGCAGGAATAGTTGGTACAGGAACAAGAATACT

GATTCGAATTGAACTAGCTCAACCAGGAGCTTTTCTAGGAAGAGATCAATTATATAACAC

TCTAGTAACAGCACATGCGTTTTTAATAATTTTCTTTTTAGTTATACCTGTATTTATTGG

CGGATTCGGAAACTGACTTCTTCCATTAATATTAGGTGCTCCAGATATGGCATTCCCACG

ACTAAATAATCTTAGATTCTGACTTCTACCACCATCATTAATTCTATTAATTTCATCTGC

AGCCGTTGAAAAAGGTGCTGGAACAGGATGAACTGTATATCCTCCATTATCAAGAAATCT

AGCTCACGCTGGACCTTCAGTTGACATGGCTATTTTTTCACTACATCTAGCAGGTGCATC

TTCTATTTTAGGTGCAGTTAACTTCATTACTACAGTAATAAACATACGATGAAATGGAAT

ACGACTTGAACGAGTACCATTATTTGTATGAGCTGTAACACTTACTGTAATTCTTCTTCT

TTTATCATTACCTGTATTAGCTGGTGCAATTACCATACTATTAACAGATCGAAATCTAAA

TACCTCATTCTTCGATCCTGCAGGAGGGGGAGACCCAATTTTATACCAACATTTATTC

>N9*_983_Nais_communis_2ind

TACATTATATTTAATTCTAGGAGTATGAGCAGGAATAATCGGAACAGGGACAAGAATACT

AATTCGAATTGAATTAGCTCAACCCGGAGCCTTCTTAGGAAGAGACCAACTATACAACAC

ATTAGTTACTGCACACGCGTTTTTAATAATTTTCTTTCTTGTTATACCTGTATTCATTGG

TGGATTCGGAAACTGACTTCTACCTCTAATACTTGGTGCACCAGATATAGCATTCCCACG

ACTTAATAATCTTAGGTTCTGATTACTCCCCCCATCATTAATTCTTTTAGTTTCATCCGC

GGCCGTTGAAAAAGGAGCCGGAACAGGTTGAACTGTTTACCCACCACTATCAAGAAACCT

GGCCCATGCAGGACCATCTGTAGACATGGCTATTTTCTCACTACATTTAGCCGGGGCATC

ATCTATTTTAGGAGCAGTAAATTTTATTACAACAGTAATAAATATACGATGAAACGGTAT

ACGATTAGAACGACTACCACTATTTGTATGAGCTGTAATACTTACTGTAATCCTTCTACT

ACTATCACTACCAGTTCTAGCAGGAGCAATTACTATACTATTAACAGACCGTAATTTAAA

TACATCATTCTTCGACCCCGCTGGAGGGGGAGACCCAATCTTATATCAACATTTATTC

>N12*_984_Nais_stolci_pardalis_2ind

TACACTATATCTAATTTTAGGAGTATGAGCAGGAATAGTAGGAACTGGTACAAGATTATT

AATTCGAATTGAACTATCACAACCAGGATCATTTCTTGGAAGAGATCAACTATATAATAC

TCTCGTAACAGCCCACGCATTCTTAATAATTTTCTTCTTAGTAATACCTGTATTTATTGG

GGGGTTTGGAAACTGACTTCTTCCATTAATACTAGGTGCTGCTGATATGGCATTCCCACG

ACTAAACAACCTTAGATTTTGACTACTACCACCATCACTAATTCTATTAGTTTCTTCTGC

TGCTGTAGAAAAAGGAGCCGGCACAGGATGAACAGTATATCCACCACTATCAAGAAATCT

AGCCCATGCTGGACCTTCAGTAGATATGGCTATTTTTTCACTTCATTTAGCAGGTGCTTC

TTCTATTTTAGGAGCTGTAAACTTTATTACGACTGTAATAAACATGCGTTGAAATGGAAT

ACGATTAGAACGACTACCACTATTTGTATGAGCTGTATTCCTTACAGTAATTCTTCTACT

ATTATCTCTTCCAGTTCTTGCCGGAGCTATTACAATGCTTCTAACAGACCGAAACCTTAA

TACTTCATTCTTCGACCCTGCTGGTGGTGGAGACCCGATCCTTTACCAACACTTATTC

>N9*_992_Nais_communis

TACATTATATTTAATTCTAGGAGTATGAGCAGGAATAATCGGAACAGGGACAAGAATACT

AATTCGAATCGAATTAGCTCAACCCGGAGCCTTCTTAGGAAGAGATCAATTATACAACAC

CTTAGTTACTGCACACGCGTTTTTAATAATTTTCTTTCTTGTTATACCTGTATTTATTGG

TGGATTCGGAAATTGACTTCTACCTCTAATACTAGGTGCACCAGATATAGCATTCCCACG

ACTTAATAATCTTAGATTCTGATTACTTCCGCCATCATTAATTCTTTTAGTTTCATCAGC

GGCCGTTGAAAAAGGAGCCGGAACTGGTTGAACTGTTTACCCACCATTATCAAGAAATCT

AGCCCATGCAGGACCATCAGTAGACATGGCTATTTTCTCATTACATTTAGCCGGGGCATC

ATCTATTTTAGGAGCAGTTAATTTTATTACAACAGTAATAAATATGCGATGAAATGGTAT

ACGATTAGAACGACTACCACTATTTGTATGAGCTGTAACACTTACTGTAATCCTTCTACT

ATTATCATTACCAGTTCTAGCAGGAGCAATTACTATGCTATTAACAGACCGTAATTTAAA

TACATCATTCTTCGACCCAGCGGGAGGAGGAGACCCAATTCTATATCAACATTTATTT

>N14*_1010_Uncinais_uncinata

TACACTATACTTAATTTTAGGAGTATGAGCGGGAATAGTAGGAACTGGAACTAGATTACT

TATTCGAATTGAACTATCACAACCAGGATCATTTCTTGGAAGAGATCAATTATATAACAC

TCTTGTAACAGCACATGCATTCTTAATAATTTTCTTCTTAGTAATACCAGTATTTATTGG

GGGATTTGGCAACTGACTTCTCCCATTAATACTAGGTGCTGCCGATATAGCATTTCCTCG

ATTAAATAATCTTAGATTTTGATTACTTCCACCATCATTAATTCTATTAGTTTCTTCTGC

TGCAGTAGAAAAAGGTGCAGGAACAGGATGAACTGTATACCCACCACTATCAAGAAATCT

AGCTCACGCTGGCCCTTCTGTAGATATGGCTATTTTTTCACTACACTTAGCTGGTGCTTC

TTCTATTCTAGGAGCAGTTAATTTTATCACCACTGTAATAAATATACGTTGAAACGGAAT

ACGACTAGAACGACTTCCACTATTTGTTTGATCAGTATTCCTTACAGTAATTCTTCTCTT

ATTATCTCTTCCTGTACTTGCTGGTGCAATTACAATACTATTAACAGATCGAAACCTAAA

TACCTCATTCTTCGATCCTGCTGGTGGTGGAGACCCTATTCTATATCAACATTTATTC

>LL2_1019_Lumbriculus_variegatus

CACTCTATATTTTATTCTTGGCGTCTGAGCCGGCATAGTAGGAGCAGGAATAAGACTACT

AATCCGAATTGAGCTCACACAACCAGGATCATTTCTAGGCAGAGATCAACTATACAATAC

CATAGTTACCGCACATGCCTTTATTATAATTTTCTTTATAGTAATACCTATATTTATTGG

CGGATTCGGAAATTGATTACTACCATTAATACTAGGTGCTCCAGACATAGCATTTCCACG

ACTAAATAATCTTAGTTTTTGACTACTACCCCCTTCCCTAATTTTATTAGTAAGATCAGC

TGCAGTAGAAAAAGGAGCAGGAACTGGATGAACTGTATACCCACCTCTAGCAAGTAATCT

AGCACACGCTGGACCTTCTGTAGATCTAGCTATCTTCTCCCTTCATTTAGCCGGAGCATC

CTCAATTCTAGGAGCCCTAAACTTTATTACAACTGTTATCAATATACGATGAAATGGTAT

ACGTCTAGAACGAATTCCTTTATTTGTATGAGCCGTAATAATTACAGTAATTTTACTCCT

ATTATCCTTACCGGTATTAGCAGGAGCCATTACTATATTACTAACAGATCGAAACCTAAA

CACCTCCTTCTTTGATCCAGCTGGGGGTGGAGACCCTGTCCTATATCAACATCTATTT

>E17*_1028_Enchytraeus_buchholzi

CACATTATATTTTATTCTAGGAGTGTGGGCCGGAATAATAGGAGCAGCTATAAGCCTATT

AATTCGAATTGAACTTAGACAACCTGGATCATTCTTAGGAAGAGATCAACTATACAACAC

TATTGTAACAGCACATGCATTTCTAATAATTTTCTTCTTAGTTATACCAGTATTTATTGG

GGGCTTTGGTAACTGATTACTCCCATTAATATTGGGGGCCCCTGATATGGCCTTCCCACG

ACTAAACAATATAAGATTTTGATTACTTCCCCCAGCACTTATACTGCTTTTATCATCGGC

AGCAGTAGAAAAAGGGGCAGGTACAGGATGAACTGTGTACCCTCCTCTAGCCAGAAACAT

CGCACATGCAGGCCCATCAGTTGACCTAGCAATTTTTTCTCTTCATTTAGCAGGAGCCTC

ATCAATTTTAGGAGCTGTAAATTTCATCACTACAGTTATTAATATGCGATGACAAGGACT

TACACTAGAACGAATTCCCCTTTTCGTTTGAGCAGTTACAATTACAGTAGTGTTACTACT

CCTATCTTTACCAGTACTAGCTGGAGCAATTACTATGCTACTAACCGATCGAAACCTAAA

TACATCATTTTTCGACCCGGCTGGTGGTGGAGACCCAATTCTCTACCAACATTTATTC

>N11*_1029_Nais_christinae

TACACTTTATCTAATTTTAGGAGTATGAGCAGGAATAGTAGGAACCGGAACAAGATTACT

TATTCGAATTGAATTATCACAACCAGGATCATTTCTTGGAAGAGATCAATTATATAATAC

TCTTGTAACAGCACATGCATTCTTAATAATTTTCTTCTTAGTAATACCTGTATTTATTGG

GGGGTTCGGAAACTGACTTCTTCCACTAATACTAGGAGCTGCTGATATGGCATTTCCTCG

ATTAAACAATCTTAGATTTTGATTACTACCACCTTCATTAATTCTATTAATTTCGTCTGC

AGCAGTAGAAAAAGGTGCAGGAACTGGATGAACTGTATACCCTCCTCTATCTAGAAATCT

AGCTCATGCTGGGCCTTCAGTAGATATGGCTATTTTCTCACTTCATTTAGCAGGTGCTTC

TTCTATTCTTGGAGCAGTAAATTTTATTACAACTGTAATAAACATACGTTGAAACGGAAT

GCGATTAGAACGACTTCCATTATTTGTATGAGCAGTATTTCTTACAGTAATTCTCCTTCT

TCTATCTCTCCCAGTTCTTGCTGGGGCAATCACCATATTACTAACAGATCGAAATCTAAA

CACTTCATTCTTTGATCCTGCTGGTGGTGGAGATCCAATTTTATATCAACATTTATTC

>P1*_1037_Pristina_jenkinae

AACTCTATATTTATCTTCGGGTGTATGAGCAGGAATAGTTGGAACCGGAACAAGACTACT

CATTCGAGTTGAATTAGCTCAACCAGGCTCATTTCTCGGAAGGGACCAACTTTACAATAC

ACTTGTTACTGCACATGCATTCCTAATAATTTTCTTTCTAGTAATGCCAGTATTTATTGG

AGGATTCGGTAATTGACTTCTTCCATTAATACTAGGAGCACCAGACATGGCATTTCCACG

ACTAAACAACATAAGATTTTGACTACTTCCCCCTGCACTAATTATACTAGTAGCTTCAGC

AGCAGTTGAAAAGGGGGCAGGAACAGGGTGAACAGTATATCCCCCACTTGCAAGAAATAT

TGCTCATGCAGGACCATCTGTAGACATAGCAATTTTTTCTCTTCATCTAGCAGGTGCATC

ATCAATCCTAGGGGCAGTAAACTTTATCTCAACTGTCCTAAATATACGAACTAAAGGAAT

ACGACTAGAACGAATTCCTCTATTTGTATGAGCTGTATTCTTAACAGTAATCCTACTACT

TCTGTCACTTCCAGTACTAGCAGGAGCAATTACCATACTTCTTACTGATCGTAACCTAAA

TACTTCATTCTTTGACCCAGCTGGGGGTGGTGACCCAATCCTATATCAACATCTATAT

>N13*_1039_Nais_pseudobtusa

TACACTATATTTAATTTTAGGAGTATGAGCAGGAATAGTAGGAACTGGAACTAGATTACT

TATTCGAATTGAACTATCACAACCAGGATCATTCCTTGGAAGAGATCAATTATATAATAC

TCTTGTAACAGCACATGCATTCTTAATAATTTTCTTCTTAGTAATACCAGTATTTATTGG

AGGATTCGGTAACTGACTGCTTCCACTAATACTAGGTGCTGCCGATATAGCATTCCCACG

ATTAAACAATCTTAGATTTTGACTTCTTCCACCATCATTAATTCTATTAGTTTCTTCTGC

CGCTGTAGAAAAAGGTGCGGGAACAGGATGAACTGTATATCCACCTCTATCAAGAAATCT

AGCGCACGCTGGACCTTCTGTTGATATGGCTATTTTTTCACTTCATTTAGCTGGTGCTTC

TTCTATTTTAGGAGCAGTAAATTTTATCACTACTGTAATAAATATACGATGAAATGGAAT

ACGATTAGAACGACTTCCACTGTTTGTATGAGCTGTATTTCTTACAGTAATTCTTCTTTT

ACTTTCTCTTCCAGTTCTTGCTGGTGCAATTACTATACTATTAACTGATCGAAATCTTAA

TACTTCATTCTTCGATCCTGCTGGAGGTGGAGATCCAATTCTATATCAACATCTATTC

>N15*_1041_Chaetogaster_diastrophus

CACTCTATACTTAATTTTAGGAGTTTGAGCAGGAATAATTGGTACAGGAACTAGAATACT

AATTCGAATTGAACTATCACAACCAGGATCATTCCTTGGGAGAGATCAATTATATAACAC

TCTAGTTACAGCCCATGCATTCCTAATAATTTTCTTCTTAGTGATACCAGTATTCATTGG

TGGATTCGGAAACTGACTTCTTCCTCTAATACTAGGTGCTCCAGATATGGCATTCCCACG

ACTTAATAATTTAAGATTTTGACTTTTACCTCCATCACTAATTTTACTTATTTCATCAGC

AGCGGTGGAAAAAGGAGCAGGAACAGGATGAACTGTATACCCTCCTCTATCTAGAAATCT

TGCCCATGCAGGACCGTCCGTAGACATGGCTATTTTTTCTCTTCACTTAGCAGGTGCTTC

ATCTATTTTAGGAGCAGTGAATTTTATTACAACTGTAATTAACATACGATGAAACGGAAT

ACGACTAGAACGACTTCCTCTATTTGTATGAGCAGTATTCTTAACAGTCATTCTTCTTCT

ACTTTCTCTTCCAGTACTTGCCGGAGCTATTACTATACTATTAACAGATCGAAACCTAAA

TACTTCTTTCTTTGATCCAGCTGGTGGTGGTGACCCTATTCTATACCAACATCTATTT

>E8*_1043_Cernosvitoviella_minor

AACACTTTACTTTATTTTAGGGGTATGAGCAGGAATACTAGGAGCTGCTATAAGACTTCT

AATTCGAATTGAACTAAGACAACCTGGCTCATTCCTAGGGAGAGATCAATTATATAATAC

TATCGTTACAGCACATGCATTCTTAATAATTTTTTTCTTAGTAATACCAGTATTTATTGG

AGGGTTTGGGAACTGACTTTTACCATTAATATTAGGGGCCCCAGACATGGCCTTTCCTCG

ATTAAACAACATAAGATTTTGACTCCTACCTCCATCATTAATACTATTAGTATCTTCAGC

AGCTGTAGAAAAAGGGGCCGGCACTGGATGAACAGTGTACCCCCCTCTTTCTAGAAACTT

AGCACATGCTGGGCCATCAGTAGATTTAGCAATTTTTTCTCTTCATCTTGCAGGAGCTTC

TTCAATTCTTGGAGCTGTAAACTTTATTACTACAGTAGTGAATATACGTTGACAAGGCCT

TCGGCTAGAACGAATTCCCCTATTTGTATGAGCAGTAGTTATTACAGTAGTTCTACTACT

TTTATCTCTACCAGTACTAGCAGGAGCAATTACTATACTATTAACAGACCGAAATTTAAA

TACATCATTCTTTGATCCGGCAGGAGGAGGAGATCCTATTTTATACCAACACTTATTC

>LC4_1048_Helodrilus_oculatus

AACCTTATATTTTATCCTTGGTGTTTGAGCCGGCATAGTAGGAGCTGGCATAAGCCTTCT

CATTCGAATTGAACTAAGACAACCGGGAGCCTTCCTGGGAAGAGATCAACTTTACAATAC

AATTGTTACAGCTCATGCATTCGTAATAATCTTCTTCCTTGTTATGCCCGTATTTATTGG

AGGATTTGGAAATTGGCTTCTTCCATTAATACTGGGTGCCCCTGATATGGCGTTCCCCCG

ACTAAATAACATAAGATTTTGACTACTTCCCCCCTCATTAATCCTCTTAGTTTCCTCTGC

AGCAGTAGAAAAAGGAGCAGGAACCGGCTGAACAATGTATCCACCTTTAGCCAGAAATCT

TGCCCACGCAGGTCCCTCAGTAGATTTAGCCATTTTTTCCCTACACTTAGCAGGGGCATC

TTCAATTCTCGGGGCCATCAATTTTATTACAACAGTTATCAACATACGATGAAGAGGTCT

ACGTCTAGAACGTATTCCTTTATTCGTCTGAGCCGTTGTAATTACGGTAGTACTCCTACT

CCTATCTCTTCCAGTGCTGGCAGGGGCAATTACTATGCTCCTCACAGACCGAAACCTTAA

TACCTCTTTCTTTGACCCTGCTGGAGGGGGGGATCCTATCTTATACCAACATTTATTT

>T17_LN810391_Limnodrilus_hoffmeisteri

CACCCTATACATAATCTTTGGCCTTTGAGCAGGAATAGTAGGCACAGGAACTAGACTTTT

AATTCGATTTGAGCTAGCACAACCCGGCTCATTTCTCGGTAGAGACCAATTATATAACAC

TCTAGTCACGGCTCACGGATTTTTAATAATTTTCTTTATAGTAATACCTATCTTTATTGG

GGGATTTGGAAATTGATTAGTGCCTTTAATACTTGGAGCACCTGATATGGCATTCCCACG

GCTTAATAACCTAAGATTCTGACTAATGCCCCCATCACTAATTCTACTAGTCTCATCAGC

TGCAGTTGAAAAAGGCGCAGGGACAGGGTGAACTGTATACCCCCCCTTAGCAAGAAATCT

AGCTCATTCCGGGCCTTCTGTAGATCTGGCAATTTTTTCACTACACTTAGCAGGAGCCGC

ATCAATCCTGGGAGCAATTAACTTCATCACAACAATAATCAATATACGATGAAAGGGAAT

ACGCTTAGAGCGTATTCCTTTATTTGTGTGATCCGTAATCATCACAGTTATTTTACTTCT

TCTTACCCTTCCAGTTCTTGCCGGAGCTATCACCATACTTTTAACAGATCGAAACCTAAA

CACCTCATTCTTTGATCCGGCAGGTGGAGGCGATCCAGTTTTATACCAACATCTATTT

>T12_LN810418_Tubifex_tubifex

AACCCTATACATAATTTTCGGAATCTGAGCTGGTATAGTTGGAACTGGAACTAGGCTATT

AATTCGCCTTGAACTTGCCCAACCTGGGTCCTTTTTAGGAAGAGACCAATTATATAATAC

CCTGGTAACGGCTCACGCATTTTTAATAATCTTCTTCATAGTGATACCAATCTATATTGG

GGGCTTCGGCAACTGACTAGTACCATTAATACTGGGGGCTCCAGACATGGCCTTCCCACG

ATTAAACAACTTAAGATTCTGACTCCTACCCCCATCATTAATCCTTTTAGTGTCCTCTGC

CGCCGTTGAAAAAGGCGCCGGAACTGGCTGAACTGTTTACCCTCCTCTAGCTAGAAATCT

AGCTCACTCTGGACCCTCAGTAGATTTAGCAATCTTTTCTCTACACTTAGCTGGTGTTGC

ATCAATCCTGGGTGCAATCAACTTCATTACTACAATAATCAACATACGATGAAAAGGTAT

GCGACTAGAACGTATTCCTTTATTCGTATGATCAGTAATTATCACTGTAATTCTACTCCT

ACTCACACTTCCAGTTTTAGCTGGGGCTATCACAATACTTCTAACAGACCGGAATCTAAA

TACATCATTTTTCGATCCTGCTGGAGGGGGTGACCCTGTACTATATCAACACCTATTC

**Supplemental Fig. 1**

>T25_824_Embolocephalus_velutinus_9ind

--CCCTATATATAGTATTTGGTGTATGAGCCGGTATAGTTGGAACCGGCACTAGATTACT

AATCCGCTTTGAGCTCGCGCAACCTGGATCATTCCTAGGTAGTGACCAACTCTACAATAC

GTTAGTTACTGCTCACGCCTTCCTTATAATCTTTTTCCTAGTAATACCTGTATTCATCGG

GGGATTCGGAAATTGACTTCTCCCATTAATATTAGGGGCACCAGATATAGCATTCCCTCG

CTTAAATAATCTAAGATTTTGGCTGCTGCCACCATCTTTAATTCTTCTAGTATCTTCGGC

GGCTGTAGAAAAGGGGGCCGGAACAGGGTGAACAGTGTACCCCCCACTATCAGGTAACTT

AGCCCATTCGGGACCATCAGTAGACTTAGCAATCTTTTCTCTTCATTTAGCTGGTGCATC

GTCCATTTTAGGGGCAATCAACTTCATTACTACAGTAATTAACATACGAAGTAAAGGTAT

GCGGCTGGAACGGGTACCACTATTTGTATGGGCTGTCCTTCTTACAGTAATTCTTCTCCT

GTTAACCCTTCCAGTACTCGCCGGAGCTATTACTATATTACTGACAGACCGCAATCTAAA

TACGTCGTTCTTTGATCCAGCTGGAGGTGGTGACCCAGTACTATACCAACACCTATTC

>T25_749_Embolocephalus_velutinus

AACTCTATATATAGTATTTGGTGTGTGAGCCGGTATAGTTGGAACCGGCACTAGATTACT

AATCCGCTTTGAGCTTGCGCAACCTGGATCATTCCTAGGCAGTGACCAACTCTACAATAC

ATTAGTTACTGCTCACGCCTTCCTAATAATCTTTTTCCTAGTAATACCTGTATTCATCGG

AGGATTCGGAAATTGGCTTCTCCCGCTAATATTAGGGGCACCAGATATAGCATTCCCCCG

CTTAAATAACCTAAGATTTTGGCTGCTGCCACCATCTTTAATTCTTCTAGTGTCTTCGGC

GGCCGTAGAAAAAGGAGCCGGAACAGGATGAACAGTATATCCCCCGCTTTCCGGTAACTT

AGCCCATTCGGGGCCATCAGTAGACTTAGCAATCTTTTCCCTTCACTTAGCCGGTGCGTC

GTCCATTTTAGGGGCAATCAACTTCATTACTACAGTAATTAACATACGAAGTAAAGGCAT

ACGACTAGAGCGGGTACCACTATTTGTATGAGCTGTCCTTCTTACAGTAATTCTTCTCCT

GTTAACCCTCCCAGTACTCGCCGGAGCTATTACTATATTACTGACAGACCGCAATCTAAA

TACGTCATTCTTTGACCCGGCTGGTGGTGGCGACCCAGTACTATACCAACACCTATTC

>T24_822_Spirosperma_ferox_2ind

--CCTTATATATAATTTTTGGTGTATGAGCCGGTATAGTAGGCACCGGAACTAGTCTATT

AATTCGCTTCGAGCTTGCTCAACCAGGGTCATTCCTAGGTAGAGACCAATTATACAATAC

ATTAGTAACCGCACATGCCTTCCTAATAATTTTCTTCTTAGTAATACCAGTATTTATTGG

GGGCTTCGGCAACTGACTAATCCCACTAATATTAGGTGCCCCTGATATAGCATTCCCACG

ACTGAATAATTTAAGATTCTGACTACTTCCTCCGTCCCTTATTCTCCTTGTATCCTCTGC

CGCAGTAGAAAAAGGTGCAGGTACCGGATGAACTGTTTATCCCCCTTTAGCTGGCAACCT

CGCCCACTCCGGCCCTTCAGTAGACTTAGCAATTTTTTCTCTCCATCTAGCCGGTGCATC

TTCTATTTTAGGTGCAATCAACTTTATTACAACTATGGTTAACATACGAAGAAAAGGTAT

ACGCCTGGAACGAGTTCCTCTATTTGTTTGAGCTGTTATTTTAACAGTAATCCTCCTATT

ACTTACACTTCCTGTATTAGCCGGTGCTATCACCATACTTCTAACAGATCGTAATCTCAA

CACGTCATTCTTTGACCCTGCTGGAGGGGGCGACCCTGTACTATACCAAACACCATTC

>T33_995_Tasserkidrilus_kessleri_2ind

AACACTATATATAATCTTTGGATTCTGAGCCGGAATAGTTGGTACTGGAACAAGACTTTT

AATTCGATTTGAATTAGCCCAACCTGGGTCCTTCCTCGGCAGAGATCAACTCTACAATAC

ACTAGTTACAGCCCATGCATTCTTAATAATTTTCTTCCTTGTTATGCCAGTCTTCATTGG

TGGATTTGGCAATTGATTAGTTCCCTTAATACTAGGAGCTCCTGATATAGCATTCCCACG

ATTAAATAATTTAAGATTCTGACTACTACCCCCCTCCCTGATCCTACTAGTATCCTCGGC

CGCCGTAGAAAAGGGGGCAGGAACAGGATGAACAGTTTATCCACCATTAGCTGGCAATTT

AGCCCATTCCGGCCCATCAGTAGACCTGGCTATTTTCTCTCTTCACCTGGCAGGAATCGC

TTCTATTTTAGGGGCAATTAATTTTATTACTACAATAATTAATATACGATGAAAAGGTAT

ACGTCTAGAACGTATCCCTCTATTTGTATGAGCAGTAATCCTGACAGTAATTCTTCTTCT

CCTAACTCTCCCTGTCCTAGCAGGTGCTATTACTATACTCCTAACTGACCGAAATCTAAA

TACATCATTCTTTGACCCTGCTGGGGGTGGTGATCCTGTGCTATATCAACATCTATTT

>T8_913_Psammoryctides_barbatus_2ind

TACTTTATATTTAATTTTTGGCTTATGAGCTGGGATGGTCGGAACTGGGACTAGTTTATT

AATTCGTATAGAACTCGCTCAACCAGGATCATTCCTTGGAAGAGACCAGTTATATAACAC

ACTAGTAACAGCACATGCTTTTCTAATAATCTTCTTCCTAGTAATACCCGTATTTATTGG

TGGGTTTGGTAATTGACTTCTACCTTTAATACTTGGGGCACCAGATATAGCTTTCCCACG

CCTAAACAATTTAAGATTTTGATTATTACCTCCATCCCTTATTCTTCTAGTATCATCCGC

TGCTGTTGAAAAAGGTGCTGGAACAGGATGAACAGTATATCCACCACTAGCTGGTAACCT

GGCACACTCTGGCCCTTCTGTAGACCTGGCTATTTTCTCTCTTCATTTAGCTGGTGCCGC

ATCTATTTTAGGAGCTATTAATTTTATTACTACTATAATTAATATACGGTGAAAAGGTAT

ACGATTAGAGCGAATTCCTTTATTTGTGTGAGCTGTAATCATTACAGTAGTTCTTCTTTT

ATTAACCCTTCCAGTATTAGCCGGTGCAATTACTATACTACTAACGGATCGAAATCTAAA

TACATCATTCTTTGATCCTGCTGGTGGTGGGGATCCTGTTCTTTATCAACACTTATTC

>T8_907_Psammoryctides_barbatus_2ind

TACTTTATATTTAATTTTTGGCTTATGAGCTGGGATGGTCGGAACTGGAACTAGTTTATT

AATTCGTATAGAACTCGCTCAACCGGGATCATTCCTTGGAAGAGACCAGTTATATAACAC

ACTAGTAACAGCACATGCTTTCCTAATAATCTTCTTCCTAGTAATACCCGTATTTATTGG

TGGGTTTGGTAATTGACTTCTACCTTTAATACTTGGGGCACCAGATATAGCTTTTCCACG

CCTAAACAATTTAAGATTTTGATTATTACCTCCATCCCTTATTCTTCTAGTATCATCTGC

TGCTGTTGAAAAAGGTGCTGGAACAGGATGAACAGTATATCCACCACTAGCTGGTAACCT

GGCACACTCTGGCCCTTCTGTAGACCTGGCTATTTTCTCTCTTCATTTAGCTGGTGCCGC

ATCTATTTTAGGAGCCATTAATTTTATTACTACTATAATTAATATACGGTGAAAAGGTAT

ACGATTAGAGCGAATTCCTCTATTTGTATGAGCTGTAATCATTACAGTAATTCTTCTTTT

ATTAACCCTTCCAGTATTAGCAGGTGCAATTACTATACTACTAACGGATCGAAATCTAAA

TACATCATTCTTTGATCCTGCTGGTGGTGGGGATCCTGTTCTTTATCAACACTTATTC

>T8_LN810374_Psammoryctides_barbatus_4ind

TACTTTATATTTAATTTTTGGCTTATGAGCTGGGATGATCGGAACTGGAACTAGTTTATT

AATTCGTATAGAACTCGCTCAACCGGGATCATTCCTTGGAAGAGACCAGTTATATAACAC

ACTAGTAACAGCACATGCTTTCCTAATAATCTTCTTCCTAGTAATACCCGTATTTATTGG

TGGGTTTGGTAATTGACTTCTACCCTTAATACTTGGGGCACCAGATATAGCTTTCCCACG

CCTAAACAATTTAAGATTTTGATTATTACCTCCATCCCTTATTCTTCTAGTATCATCTGC

TGCTGTTGAAAAAGGTGCTGGAACAGGATGAACAGTATATCCACCACTAGCTGGTAACCT

CGCACACTCTGGCCCTTCTGTAGACCTGGCTATTTTCTCTCTTCACTTAGCTGGTGCCGC

ATCTATTTTAGGAGCCATTAATTTTATTACTACTATAATTAATATACGGTGAAAAGGTAT

ACGATTAGAGCGAATTCCTCTATTTGTGTGGGCTGTAATCATTACAGTAATTCTTCTTTT

ATTAACCCTTCCAGTATTAGCCGGTGCAATTACTATACTACTAACGGATCGAAATCTAAA

TACATCATTCTTTGATCCTGCTGGTGGTGGGGATCCTGTTCTTTATCAACACTTATTC

>T11_962_Tubifex_tubifex_2ind

AACTCTATATATTATTTTCGGGATTTGAGCTGGAATGGTCGGGACAGGAACTAGCCTCTT

AATTCGTCTAGAACTGGCTCAACCTGGCTCATTCCTTGGAAGGGATCAACTATACAACAC

CCTAGTTACAGCACACGCATTTCTGATAATTTTCTTCATGGTAATACCTATCTATATCGG

GGGCTTCGGCAATTGACTAGTGCCTTTAATACTGGGGGCCCCTGACATAGCATTCCCACG

ATTAAATAATTTAAGATTTTGACTATTACCTCCTTCCTTAATCCTCCTTGTATCCTCCGC

AGCCGTAGAAAAGGGGGCTGGAACAGGGTGAACAGTATATCCACCCCTAGCTAGAAATCT

GGCTCATTCTGGACCTTCTGTAGACTTAGCTATTTTCTCCCTACATTTAGCGGGAGTAGC

ATCGATTCTAGGGGCTATTAACTTTATTACTACAATAATTAACATGCGGTGAAAAGGGAT

ACGTCTTGAACGAATTCCTCTATTTGTATGAGCCGTAATTCTAACTGTAATCTTACTTCT

ACTAACATTACCAGTTTTAGCGGGTGCTATTACCATGCTACTGACAGATCGAAACCTAAA

CACATCCTTCTTTGACCCTGCTGGGGGTGGTGATCCTGTACTCTATCAACACTTATTC

>T11_852_Tubifex_tubifex

AACTCTATACATCATTTTCGGGATTTGAGCTGGGATGGTCGGGACAGGAACTAGACTCTT

AATTCGCCTAGAACTGGCTCAACCTGGCTCATTCCTTGGAAGAGATCAGCTATACAACAC

CCTAGTTACAGCACATGCATTTCTGATAATTTTCTTCATGGTAATACCTATCTATATTGG

GGGTTTCGGAAATTGGTTAGTACCCTTAATGCTGGGGGCTCCTGACATAGCATTCCCCCG

ATTAAATAATTTAAGATTTTGGTTATTACCCCCTTCCTTAATCCTCCTTGTATCCTCCGC

GGCCGTAGAAAAGGGGGCTGGGACAGGGTGAACAGTATATCCGCCCCTAGCTAGAAATCT

GGCTCATTCTGGGCCTTCTGTAGACTTAGCTATTTTCTCTCTACATTTAGCTGGGGTGGC

ATCGATTCTAGGAGCTATTAACTTTATTACTACAATAATTAACATGCGATGAAAAGGGAT

ACGCCTTGAACGAATTCCCCTATTTGTGTGAGCTGTAATTCTAACCGTAATCTTACTTTT

ACTAACATTACCAGTTTTAGCTGGTGCCATTACCATGCTACTAACAGATCGAAATCTAAA

CACATCCTTCTTTGACCCTGCTGGGGGTGGTGATCCTGTACTCTATCAACACTTATTC

>T11_773_Tubifex_tubifex_14ind

AACTCTATATATCATTTTCGGGATTTGAGCTGGGATGGTCGGGACAGGAACTAGACTCTT

AATTCGTCTAGAACTGGCTCAACCTGGCTCATTCCTTGGAAGGGATCAGCTATACAACAC

CCTAGTTACAGCACATGCATTTCTGATAATTTTCTTCATGGTAATACCTATCTATATTGG

GGGTTTCGGAAATTGGTTAGTACCCTTAATGCTGGGAGCTCCTGACATAGCATTCCCCCG

ATTAAATAATTTAAGATTTTGGTTATTACCCCCTTCCTTAATCCTCCTTGTATCCTCCGC

GGCCGTAGAAAAGGGGGCTGGAACAGGGTGAACAGTATATCCGCCCCTAGCTAGAAATCT

GGCTCATTCTGGGCCTTCTGTAGACTTAGCTATTTTCTCTCTACATTTAGCTGGGGTGGC

ATCGATTCTAGGAGCTATTAACTTTATTACTACAATAATTAACATGCGATGAAAAGGGAT

ACGCCTTGAACGAATTCCCCTATTTGTATGAGCTGTAATTCTAACCGTAATCTTACTTTT

ACTAACATTACCAGTTTTAGCTGGTGCCATTACCATGCTACTAACAGATCGAAATCTAAA

CACATCCTTCTTTGACCCTGCTGGGGGTGGAGATCCTGTGCTCTATCAACACTTATTC

>T6_1003_Lophochaeta_ignota_13ind

AACTCTCTATATAATCTTCGGCCTATGAGCGGGAATAGTCGGCACCGGTACTAGACTACT

AATTCGACTAGAATTAGCTCAACCCGGATCATTCCTTGGTAGTGATCAACTATATAATAC

ACTTGTTACTGCCCATGGATTCCTTATAATTTTCTTCATGGTAATACCAGTATTTATTGG

GGGATTTGGTAACTGACTAGTCCCATTAATACTTGGTGCCCCAGATATAGCCTTCCCACG

AATAAATAATTTAAGATTTTGATTAATACCCCCCTCTTTAATCCTTCTCGTATCTTCAGC

AGCCGTTGAAAAAGGTGCTGGAACAGGCTGAACAGTATATCCTCCATTAGCCGGGAATTT

AGCTCACTCAGGACCATCCGTAGACCTAGCTATTTTCTCACTGCATTTAGCCGGAGCTGC

CTCAATTTTAGGTGCAATTAACTTTATTACGACTATAATTAACATGCGATGAAAAGGAAT

ACGCCTAGAACGCATTCCTCTATTTGTTTGATCCGTAATTATCACAGTAGTCCTACTTTT

ATTAACACTACCAGTTCTAGCAGGGGCCATTACAATATTACTTACAGACCGAAACTTAAA

TACCTCATTCTTTGACCCTGCTGGAGGTGGAGACCCTGTTTTATATCAACATCTATTC

>T32_959_Tubificinae_sp

ATTTTTATATATATTATTTGGGCTATGAGCTGGAATAGTAGGAACTGGTACCAGTCTTCT

AATTCGGTTAGAACTTGCTCAACCAGGCTCATTCCTGGGAAGAGATCAACTTTATAATAC

TATTGTAACAGCACATGCATTCCTGATAATCTTCTACATAGTAATACCAATTTATGTAGG

AGGATTTGGAAACTATTTAATACCTTTAATACTGGGGGCACCAGATATAGCATTCCCACG

ACTAAATAACCTGAGATTCTGATTAATACCGCCATCTTTAATTCTTCTAGTAGCATCAGC

TGCTGTTGAAAAAGGGGCCGGAACTGGATGAACTGTTTACCCTCCTCTAGCTAGAAATCT

GGCACATTCTGGTCCATCAGTAGATCTAGCTATTTTTGCCTTACATCTTGCTGGTGCAGC

CTCTATTATCGGAGCCATTAATTTCATTACAACAATAATTAATATACGCTGAAAAGGGAT

GCGACTAGAGCGTATTCCCCTGTTCGTGTGATCTGTAATTATTACTGTAGTTCTATTATT

ACTTACACTTCCTGTTCTTGCAGGTGCCATTACTATACTCCTAACAGATCGAAATCTAAA

TACATCATTCTTCGATCCTGCTGGTGGCGGTGACCCTGTACTATACCAACATCTATTC

>T12_968_Tubifex_tubifex_4ind

AACTCTATACATAATTTTCGGAATCTGAGCTGGTATGGTTGGAACTGGAACTAGGCTATT

AATTCGCCTTGAACTTGCCCAACCTGGGTCCTTTTTAGGAAGAGATCAATTATATAATAC

TCTGGTAACGGCTCATGCATTTTTAATAATCTTCTTCATGGTGATACCAATCTATATTGG

GGGCTTCGGCAACTGACTAGTACCATTAATACTGGGGGCTCCAGACATGGCCTTCCCACG

ATTAAACAACTTAAGATTCTGACTCCTACCCCCATCATTAATCCTTTTAGTATCCTCTGC

CGCCGTTGAAAAAGGCGCCGGAACTGGCTGAACTGTTTACCCCCCTCTAGCTAGAAATCT

AGCTCACTCTGGGCCCTCAGTAGATTTAGCAATCTTTTCTCTACACTTAGCTGGTGTTGC

ATCAATCCTGGGTGCAATCAACTTCATTACCACAATAATCAACATACGATGAAAAGGGAT

GCGACTAGAACGTATTCCTTTATTTGTATGATCGGTAATTATCACTGTAATTCTACTCCT

ACTCACACTTCCAGTTTTAGCTGGGGCTATCACAATACTTCTAACAGACCGGAATCTAAA

TACATCATTTTTCGATCCTGCTGGAGGGGGTGACCCTGTACTATATCAACACCTATTC

>T12_986_Tubifex_tubifex_2ind

AACCCTATACATAATTTTCGGAATCTGAGCTGGTATGGTTGGAACTGGGACTAGGCTATT

AATTCGCCTTGAACTCGCCCAACCTGGGTCCTTTTTAGGAAGAGACCAATTATATAATAC

CCTGGTAACGGCTCACGCATTTTTAATAATCTTCTTCATGGTGATACCAATCTATATTGG

GGGCTTCGGCAACTGACTAGTACCATTAATACTGGGGGCTCCAGACATGGCCTTCCCACG

ATTAAACAACTTAAGATTCTGACTCCTACCCCCATCATTAATCCTTTTAGTGTCCTCTGC

CGCCGTTGAAAAAGGCGCCGGAACTGGCTGAACTGTTTACCCTCCTCTAGCTAGAAATCT

AGCTCACTCTGGACCCTCAGTAGATTTAGCAATCTTTTCTCTACACTTAGCTGGTGTTGC

ATCAATCCTGGGTGCAATCAACTTCATTACTACAATAATCAACATACGATGAAAAGGTAT

GCGACTAGAACGTATTCCTTTATTCGTATGATCAGTAATTATCACTGTAATTCTACTCCT

GCTTACACTTCCAGTTTTAGCTGGGGCTATCACAATACTTCTAACAGACCGGAATCTAAA

TACATCATTTTTCGATCCTGCTGGAGGGGGTGACCCTGTACTATATCAACACCTATTC

>T12_853_Tubifex_tubifex_4ind

--------------TTTTCGGAATCTGAGCTGGTATGGTTGGAACTGGGACTAGGCTATT

AATTCGCCTTGAACTTGCCCAACCTGGGTCCTTTTTAGGAAGAGATCAATTATATAATAC

CCTGGTAACGGCTCACGCATTTTTAATAATCTTCTTCATAGTAATACCAATCTATATTGG

GGGCTTCGGCAACTGACTAGTACCATTAATACTAGGGGCTCCGGACATGGCCTTCCCACG

ATTAAACAACTTAAGATTCTGACTCCTACCCCCATCACTAATCCTTTTAGTATCCTCTGC

CGCCGTTGAAAAAGGCGCCGGAACTGGCTGAACTGTTTACCCCCCTCTAGCTAGAAATCT

AGCTCACTCTGGGCCCTCAGTAGATTTAGCAATCTTTTCTCTACACTTAGCTGGTGTTGC

ATCAATCCTAGGTGCAATCAACTTCATTACTACAATAATCAACATACGATGAAAAGGAAT

GCGACTAGAGCGTATTCCTTTATTTGTATGATCAGTAATTATCACTGTAATTCTACTCCT

ACTCACACTTCCAGTTTTAGCTGGAGCTATTACAATACTTCTAACAGACCGGAATCTAAA

TACATCATTTTTCGATCCTGCTGGAGGGGGTGACCCTGTACTATATCAACACCTATTC

>T18_858_Limnodrilus_hoffmeisteri_7ind

----------------------------------------------------AGCCTGCT

AATTCGATTCGAATTAGCACAACCCGGTTCATTCCTCGGTAGAGATCAACTCTATAATAC

CTTAGTAACAGCCCACGGTTTCCTTATAATCTTCTTCATGGTAATACCAATTTTTATTGG

TGGCTTCGGAAATTGACTAGTCCCCCTAATGCTAGGAGCTCCTGACATGGCCTTTCCACG

ACTAAATAACCTAAGATTTTGACTAATACCTCCATCACTCATTCTATTAGTTTCATCAGC

CGCGGTCGAAAAGGGAGCGGGGACAGGGTGAACTGTATACCCCCCTCTAGCCAGAAACCT

AGCGCACTCTGGGCCATCTGTGGATCTAGCAATCTTCTCTCTTCACTTAGCCGGGGCTGC

ATCAATTCTAGGTGCCATTAACTTCATTACCACAATAATTAATATACGATGAAAAGGAAT

GCGCCTAGAGCGCATTCCTCTATTTGTATGATCAGTAATCATTACAGTTGTCCTCCTTCT

TCTTACATTACCGGTTTTAGCGGGGGCAATCACCATACTTTTAACAGACCGAAACTTAAA

CACATCATTCTTCGATCCTGCNGGAGGGGGGGACCCGGTACTATATCAACACTTATT-

>T18_768_Limnodrilus_hoffmeisteri_11ind

CACCTTATATATAATCTTCGGCCTATGAGCCGGAATAGTGGGCACAGGAACAAGCCTGCT

AATTCGATTCGAATTAGCACAACCTGGTTCATTCCTCGGAAGAGATCAACTCTATAATAC

CTTAGTGACAGCCCACGGTTTCCTTATAATCTTCTTCATGGTGATACCAATTTTTATCGG

TGGCTTCGGAAATTGACTAGTCCCCCTAATACTAGGAGCTCCTGACATGGCCTTTCCACG

ACTAAATAACCTAAGATTTTGACTAATACCTCCATCACTCATTCTATTAGTTTCATCAGC

CGCAGTCGAAAAGGGAGCGGGGACAGGGTGAACTGTATACCCCCCTCTAGCCAGAAACCT

AGCGCATTCTGGGCCATCTGTGGATCTAGCAATCTTCTCTCTTCACTTAGCCGGGGCTGC

ATCAATTCTAGGTGCTATTAACTTCATTACCACAATAATTAATATACGATGAAAAGGAAT

GCGCCTAGAGCGCATTCCTCTATTTGTATGATCAGTAATCATTACAGTTGTCCTCCTTCT

TCTTACATTACCGGTTTTAGCGGGGGCAATCACCATACTTTTAACAGACCGAAACTTAAA

CACCTCATTCTTCGATCCTGCGGGAGGGGGGGACCCGGTACTATATCAACACTTATTT

>T16_750_Limnodrilus_hoffmeisteri_2ind

CACTCTATACATGGTCTTCGGCCTCTGAGCAGGAATAGTGGGCACAGGAACCAGACTACT

AATTCGATTTGAATTAGCACAACCTGGCTCATTCCTTGGCAGAGATCAATTATATAACAC

CCTAGTTACAGCTCATGGATTTTTAATAATTTTCTTTATAGTTATACCTATTTTCATTGG

GGGCTTCGGTAACTGACTAGTCCCCCTAATACTTGGAGCTCCAGATATAGCCTTCCCACG

ACTCAATAATTTGAGATTTTGATTAATACCCCCATCTCTAATTCTATTAGTCTCCTCGGC

GGCTGTTGAGAAGGGTGCCGGAACAGGATGAACTGTATATCCACCTTTAGCTAGAAACCT

GGCTCATTCTGGGCCCTCTGTAGACCTGGCCATTTTTTCACTTCACTTAGCAGGGGCTGC

ATCAATCTTAGGTGCAATTAACTTCATTACCACAATAATTAATATACGATGAAAAGGAAT

ACGTTTAGAACGTATTCCCCTATTCGTGTGATCAGTTATTATTACGGTTATTTTACTCCT

TCTAACCTTACCAGTTCTAGCAGGGGCAATCACTATACTTCTAACAGACCGAAATCTAAA

CACGTCATTCTTCGACCCTGCGGGTGGTGGCGACCCGGTCCTATATCAACACTTATTC

>T17_755_Limnodrilus_hoffmeisteri_29ind

CACCCTATACATAATCTTTGGCCTTTGAGCAGGAATAGTAGGCACAGGAACTAGACTTTT

AATTCGATTTGAGCTAGCACAACCCGGCTCATTTCTCGGTAGAGACCAATTATATAACAC

TCTAGTCACGGCTCACGGATTTTTAATAATTTTCTTTATAGTAATACCTATCTTTATTGG

GGGATTTGGAAATTGATTAGTGCCTTTAATACTTGGAGCACCTGATATGGCATTCCCACG

GCTTAATAACCTAAGATTCTGACTAATGCCCCCATCACTAATTCTACTAGTCTCATCAGC

TGCAGTTGAAAAAGGCGCAGGGACAGGGTGAACTGTATACCCCCCCTTAGCAAGAAATCT

AGCTCATTCCGGGCCTTCTGTAGATCTGGCAATTTTTTCACTACACTTAGCAGGAGCCGC

ATCAATCCTGGGAGCAATTAACTTCATCACAACAATAATCAATATACGATGAAAGGGAAT

ACGCTTAGAGCGTATTCCTTTATTTGTGTGATCCGTAATCATCACAGTTATTTTACTTCT

TCTTACCCTTCCAGTTCTTGCCGGAGCTATCACCATACTTTTAACAGATCGAAACCTAAA

CACCTCATTCTTTGATCCGGCAGGTGGAGGCGATCCAGTTTTATACCAACATCTATTT

>T17_LN810387_Limnodrilus_hoffmeisteri_4ind

CACCCTATACATAATCTTTGGTCTTTGAGCAGGAATAGTAGGCACAGGAACTAGACTTTT

AATTCGATTTGAACTAGCTCAACCTGGCTCATTTCTTGGTAGAGACCAGTTATATAACAC

CCTGGTTACAGCTCACGGGTTTTTAATAATTTTCTTTATAGTAATACCAATCTTCATTGG

TGGATTTGGAAATTGATTAGTACCTTTAATACTTGGGGCACCTGATATGGCATTCCCACG

ACTTAATAACCTAAGATTCTGACTAATACCCCCATCACTAATTCTACTAGTCTCCTCGGC

TGCAGTTGAAAAAGGTGCGGGAACAGGGTGAACTGTATACCCCCCCCTAGCAAGAAATCT

AGCTCATTCCGGACCTTCTGTGGATCTGGCAATTTTTTCATTACACTTGGCGGGGGCCGC

ATCAATCCTTGGGGCAATCAACTTCATTACAACAATAATTAATATACGATGAAAAGGAAT

ACGTTTAGAGCGTATTCCCTTATTTGTGTGATCTGTAATTATCACGGTTGTTTTACTTCT

TCTCACTCTTCCAGTACTTGCCGGAGCTATTACCATACTTTTAACAGATCGAAACCTAAA

CACCTCATTCTTTGATCCGGCGGGGGGAGGTGATCCAGTTTTATATCAACACCTATTT

>T26_844_Tubifex_sp

AACACTATATATCATTTTTGGGGTATGAGCAGGAATGGTCGGAACAGGTACTAGATTATT

AATTCGACTAGAACTTGCCCAACCTGGCTCATTTCTTGGTAGCGATCAATTATTTAATAC

TCTGGTTACAGCCCATGCATTCCTGATAATCTTCTTCATAGTAATACCAATCTACATCGG

GGGATTCGGAAACTGATTAATTCCCTTAATACTGGGCGCCCCTGACATGGCCTTCCCTCG

TCTAAATAACTTAAGATTCTGACTTCTTCCACCATCTCTAATTCTATGTATTTCCTCGGC

AGCCGTTGAAAAAGGTGCTGGAACTGGGTGAACTGTATACCCCCCACTAGCAAGAAACCT

TGCCCACTCGGGACCATCTGTAGATCTTGCTATTTTCTCCTTACACTTAGCCGGGGTTGC

ATCAATTCTTGGTGCCATTAACTTTATTACAACTATAATCAACATACGATGAAAAGGAAT

ACGACTTGAACGAATCCCTCTATTTGTATGATCTGTAATTATTACAGTAGTTCTCCTACT

TCTTACATTACCAGTATTAGCGGGTGCTATCACTATACTACTAACAGACCGAAACCTAAA

TACCTCATTCTTCGATCCTGCCGGAGGAGGTGACCCAGTTCTTTACCAACATCTATTC

>T14_LN810383_Tubificinae_sp

TATACTGTACATAATCTTCGGCCTTTACGCCGGAGCAGTTGGAACAGGAACAAGAATACT

AATTCGATTTGAACTATCCCAACCTGGGTCATTTCTAGGAAGAGATCAATTATATAACAC

CCTAGTTACAGCTCATGGATTCCTAATAATCTTCTTTATAGTAATACCAATTTTTGTAGG

TGCATTCGGAAATTCGCTAGTTCCCCTAATACTAGGGGCCCCCGATATAGCATTTCCTCG

CCTAAACAACCTAAGATTCTGATTAATACCACCATCATTAATTCTTCTCATTTCCTCTGC

AGCTGTTGAAAAAGGTGCGGGAACTGGGTGAACTGTATATCCACCTTTAGCTAGAAATCT

AGCACATTCAGGACCATCCGTAGATCTTTGCATTTTCTCCTTACACTTAGCCGGAATTTC

ATCAATTATTGGGGCTATTAACTTTATTACTACAATAATCAATATACGATGAAAAGGTAT

GCGCCTAGAACGAATTCCTCTATTCGTGTGATCAGTAATTATTACAGTTGTTCTTCTTCT

ACTTACACTTCCAGTACTAGCCGGCGCAATTACTATACTTCTTACAGATCGAAATCTAAA

TACCTCATTCTTCGACCCGGCGGGCGGGGGTGACCCAGTCCTATACCAACATCTATTC

>T27_789_Tubifex_tubifex_5ind

AACCCTTTACATACTATTTGGTATTTGAGCAGGTATAGTTGGCACCGGAACAAGTCTACT

AATTCGTCTAGAACTAGCTCAACCTGGCTCTTTCCTCGGCAGCGACCAATTATATAATAC

ATTAGTTACAGCACATGCATTCCTTATAATCTTCTTTATGGTAATACCTATTTACATTGG

GGGATTCGGAAATTGACTAGTCCCATTAATACTGGGGGCACCAGACATAGCATTCCCACG

ACTAAATAATCTTAGATTTTGACTTCTACCCCCCTCCCTAATTCTACTTGTATCATCTGC

TGCAGTGGAAAAAGGAGCTGGAACAGGGTGAACTGTCTACCCACCACTAGCCAGTAACTT

AGCACACTCTGGACCCTCAGTAGACTTAGCAATCTTCTCATTACACTTAGCTGGTGTAGC

CTCAATTCTAGGTGCCATTAACTTCATCACTACAATAATTAATATACGTTGAAAAGGTAT

ACGCCTAGAACGAATCCCATTATTTGTTTGATCAGTAATTATTACTGTAATTCTTTTACT

ACTTACACTTCCAGTACTAGCCGGTGCAATTACCATACTACTAACAGACCGAAATCTAAA

TACTTCATTCTTCGACCCTGCCGGTGGGGGAGACCCTGTTCTTTATCAACATCTATTC

>T27_856_Tubifex_tubifex

AACCCTTTATATAGTATTTGGTATTTGAGCAGGTATAGTTGGCACCGGAACAAGTCTACT

GATTCGTCTAGAACTAGCTCAACCTGGCTCTTTCCTCGGCAGCGACCAATTATATAACAC

ATTAGTTACAGCACATGCATTCCTTATAATCTTCTTTATGGTAATACCTATTTACATTGG

GGGATTCGGAAATTGACTAGTCCCACTAATACTGGGGGCACCAGACATAGCATTCCCACG

ACTAAATAATCTTAGATTTTGACTTCTACCCCCCTCCCTAATTCTACTTGTATCATCTGC

TGCAGTAGAAAAAGGAGCTGGAACAGGGTGAACTGTCTACCCACCACTAGCCAGTAACTT

AGCACACTCTGGACCCTCAGTAGACTTAGCAATCTTCTCACTACACTTAGCTGGTGTAGC

CTCAATTCTAGGTGCCATTAACTTCATCACTACAATAATTAATATACGTTGAAAAGGTAT

ACGCCTAGAACGAATCCCATTATTTGTCTGATCAGTAATTATTACTGTAATTCTTTTACT

ACTTACACTTCCAGTACTAGCCGGTGCAATTACCATACTACTAACAGACCGAAATCTAAA

TACTTCATTCTTCGACCCTGCCGGTGGGGGAGACCCTGTTCTCTATCAACATCTATTC

>T10_LN810424_Tubifex_tubifex

AACCCTTTATATTGTATTTGGTATTTGAGCCGGTATAGTAGGCACCGGAACAAGTTTACT

GATTCGTCTAGAATTAGCTCAACCTGGCTCCTTCTTAGGCAGAGATCAATTATATAATAC

CCTAGTTACAGCCCACGCCTTCCTAATGATTTTCTTCATAGTAATGCCAATCTACATTGG

TGGCTTCGGAAACTGATTAGTACCTTTAATGCTGGGGGCACCAGACATGGCATTCCCTCG

ACTAAATAACTTAAGATTTTGATTACTACCCCCATCCTTAATCTTACTCGTATCGTCTGC

TGCAGTAGAAAAGGGGGCCGGAACAGGCTGAACTGTGTACCCTCCCTTAGCCAGAAACTT

AGCTCACTCTGGACCTTCCGTAGACCTGGCGATCTTCTCGCTACATTTAGCAGGTGTAGC

ATCAATCTTAGGGGCCATTAATTTCATTACCACAATAATTAATATACGTTGAAAAGGTAT

GCGCCTAGAACGAATTCCTTTATTTGTATGATCAGTTATTATCACTGTAATCCTTCTATT

ACTCACGCTCCCAGTACTAGCCGGTGCTATTACTATACTTCTTACAGATCGAAACCTAAA

TACCTCATTCTTCGACCCAGCCGGTGGTGGGGACCCTGTCCTTTACCAACACCTATTC

>T10_LN810423_Tubifex_tubifex_3ind

AACCCTTTATATAGTATTTGGTATTTGAGCTGGTATAGTAGGCACTGGAACAAGTTTATT

AATTCGTTTAGAATTAGCTCAACCTGGCTCCTTCTTAGGCAGAGATCAATTATATAACAC

CTTAGTTACAGCCCACGCCTTCCTGATAATCTTCTTTATGGTAATGCCAATCTACATTGG

TGGCTTCGGAAACTGACTAGTACCACTAATGCTAGGGGCACCAGACATAGCATTCCCCCG

ACTAAATAACCTAAGATTTTGACTATTACCTCCATCCCTAATCTTACTTGTATCATCTGC

TGCAGTAGAAAAAGGGGCAGGAACAGGTTGAACTGTATACCCTCCCCTAGCTAGAAATCT

AGCACATTCCGGACCCTCCGTAGACCTGGCTATCTTCTCACTACATTTAGCTGGTGTAGC

ATCAATTCTAGGAGCCATTAATTTCATTACCACAATAATCAATATACGCTGAAAAGGTAT

ACGCCTAGAACGTATTCCTTTATTCGTATGATCAGTTATTATTACTGTAATCCTTCTATT

ACTCACACTCCCAGTACTAGCCGGTGCTATTACTATACTTCTTACAGACCGAAATCTAAA

CACCTCATTCTTCGACCCTGCTGGTGGTGGAGACCCTGTCCTTTACCAACATCTATTC

>T9_LN810420_Tubifex_tubifex_3ind

AACCCTTTATATCGTATTTGGAATTTGAGCTGGAATAGTGGGAACAGGTACAAGCCTCTT

AATCCGCTTAGAATTAGCTCAACCTGGCTCTTTCCTGGGCAGAGACCAACTATATAACAC

TCTAGTTACAGCCCATGCATTCCTGATAATCTTCTTTATAGTAATACCTATCTACATTGG

TGGTTTTGGCAATTGACTGGTCCCACTTATATTAGGGGCGCCCGATATAGCATTTCCACG

ATTAAATAACTTAAGATTTTGACTACTGCCCCCTTCCTTAATTCTTCTAGTATCATCTGC

AGCGGTTGAGAAAGGGGCTGGAACTGGGTGAACCGTTTATCCTCCACTATCAAGAAATCT

TGCACACTCGGGCCCATCCGTAGACCTTGCAATCTTCTCACTCCACTTAGCCGGAGTAGC

CTCAATTTTAGGCGCTATCAATTTCATCACCACAATAATTAACATACGATGAAAAGGTAT

ACGGTTAGAACGAATTCCATTATTCGTGTGATCAGTAATTCTGACAGTAATTCTATTACT

GCTTACCTTACCTGTACTAGCAGGCGCTATTACTATACTCCTAACAGATCGAAACCTAAA

TACATCATTCTTTGATCCTGCGGGTGGTGGTGATCCAGTTCTTTACCAACATCTATTC

>T9_LN810419_Tubifex_tubifex

AACCCTTTATATCGTATTTGGGATTTGAGCTGGAATAGTAGGAACAGGTACAAGCCTCTT

AATCCGCTTAGAATTAGCTCAACCTGGCTCTTTCCTGGGCAGAGACCAACTATATAACAC

TCTAGTTACAGCCCATGCATTCCTGATAATCTTCTTTATAGTAATACCTATCTACATTGG

TGGTTTTGGCAATTGACTGGTCCCACTTATATTAGGGGCACCCGATATAGCATTTCCACG

ATTAAATAACTTAAGATTTTGACTACTACCCCCTTCCTTAATTCTTCTGGTATCATCTGC

AGCGGTTGAAAAAGGGGCTGGAACTGGATGAACCGTTTATCCTCCACTATCAAGAAATCT

TGCGCACTCGGGCCCATCCGTAGACCTTGCAATCTTCTCACTCCACTTAGCCGGGGTAGC

CTCAATTTTAGGCGCTATCAATTTCATCACCACAATAATTAATATACGATGAAAAGGTAT

ACGATTAGAACGAATTCCATTATTCGTGTGATCAGTAATTCTGACAGTAATTCTATTACT

GCTTACCTTACCTGTATTAGCAGGCGCTATTACTATACTCCTAACAGATCGAAACCTAAA

TACATCATTCTTTGATCCTGCGGGTGGTGGTGATCCAGTTCTTTACCAACATCTATTC

>T19_LN810314_Limnodrilus_hoffmeisteri

CACTCTATATATAATTTTTGGTCTCTGAGCTGGAATAGTGGGAACTGGAACAAGACTACT

AATTCGATTTGAATTAGCTCAACCCGGATCATTCTTAGGTAGTGATCAATTATACAATAC

ACTAGTAACAGCCCACGGCTTCTTAATAATTTTTTTTATGGTGATACCAATCTTTATTGG

GGGATTCGGAAATTGACTAATTCCATTAATGCTGGGAGCCCCAGATATGGCCTTTCCTCG

TCTCAATAATCTTAGATTTTGGCTGATACCACCATCATTAATTTTACTAGTATCCTCTGC

TGCAGTAGAAAAAGGCGCAGGTACCGGATGAACTGTATATCCTCCATTAGCTAGAAATCT

AGCACATTCAGGGCCATCTGTAGATCTAGCAATTTTCTCACTACATTTAGCCGGAGTAGC

TTCAATTCTAGGGGCTATCAACTTTATCACAACAATAATTAATATACGATGAAAAGGAAT

GCGATTAGAACGAATCCCATTATTTGTATGATCTGTAATTATTACTGTCATTTTACTCCT

TTTAACCCTACCAGTATTAGCCGGAGCTATTACAATATTACTGACTGACCGAAATCTAAA

TACATCATTCTTCGACCCAGCAGGGGGGGGTGATCCTGTACTTTATCAACACTTATTT

>T15_LN810385_Tubificinae_sp_3ind

CACACTATATGTAATCTTTGGCCTTTGAGCCGGGGCAGTTGGAACTGGAACAAGACTATT

AATTCGATCTGAGCTAGCCCAACCTGGATCATTCCTCGGCAGAGATCAGTTATACAATAC

CTTAGTAACAGCCCATGGATTCCTGATAATCTTCTTTATGGTAATACCAATCTTTATCGG

GGGCTTCGGAAATTGATTAGTCCCTTTAATACTAGGAGCTCCAGATATGGCATTCCCGCG

TCTAAATAATCTGAGATTTTGATTAATACCTCCATCTCTAATTCTTCTTGTTTCCTCTGC

AGCAGTTGAAAAGGGCGCAGGGACAGGATGAACTGTATATCCTCCTCTAGCTAGTAACCT

AGCACATTCAGGACCATCTGTAGATCTTGCCATTTTCTCCTTACATTTAGCAGGAATTGC

ATCAATTCTAGGATCCATCAACTTTATTACCACAATAATTAATATACGATGAAAAGGAAT

GCGCTTAGAGCGCATTCCTTTATTCGTGTGATCAGTAATTATTACTGTTGTCCTTCTCCT

GCTAACTCTACCAGTGTTAGCAGGTGCTATTACTATATTACTCACAGACCGAAATCTAAA

TACTTCGTTCTTTGACCCTGCGGGTGGAGGAGACCCAGTTCTATACCAACACCTATTC

>T20_859_Limnodrilus_hoffmeisteri_7ind

CACCCTTTACATAGTTTTTGGCTTATGAGCCGGAATAGTAGGTACCGGGACAAGATTACT

AATTCGCTTCGAACTGGCACAACCGGGATCATTCCTAGGCAGAGATCAGTTATATAATAC

ATTAGTAACTGCTCATGGTTTCTTAATAATTTTCTTCATGGTGATACCAATCTTTATCGG

TGGTTTTGGAAATTGATTAATTCCTTTAATACTTGGAGCCCCAGATATAGCGTTTCCTCG

ACTAAATAATCTTAGATTTTGATTAATGCCACCCTCTCTGATTCTACTTGTATCGTCAGC

AGCCGTGGAAAAAGGTGCAGGTACCGGATGAACTGTATACCCGCCGCTAGCTAGAAATCT

GGCACATTCAGGTCCATCGGTAGATCTAGCAATTTTTTCTTTACATTTAGCAGGTGCAGC

TTCAATTCTCGGGGCAATTAACTTCATTACTACAATAATTAACATACGGTGAAAAGGAAT

ACGTTTAGAACGTATTCCACTATTTGTGTGATCCGTAATTATTACTGTAGTCCTTCTTCT

ACTAACCCTACCTGTATTAGCAGGAGCCATTACAATATTATTAACAGATCGAAATCTAAA

TACATCATTCTTTGACCCTGCAGGAGGTGGAGACCCTGTACTCTACCAACATCTATTT

>T21_LN810304_Limnodrilus_hoffmeisteri_5ind

TACTCTATATATAATCTTTGGCTTATGAGCAGGAATAGTTGGAACCGGAACAAGCCTATT

AATTCGATTTGAGTTAGCTCAACCAGGCTCTTTCCTAGGCAGAGATCAATTATATAACAC

CTTAGTAACAGCACATGGGTTCTTAATAATTTTCTTCATAGTAATACCAATCTTCATTGG

GGGATTCGGAAACTGATTAATCCCATTAATATTAGGAGCTCCAGATATAGCATTCCCTCG

ACTAAATAATCTTAGATTCTGACTAATGCCCCCATCCTTAATTCTACTTGTGTCATCAGC

AGCTGTAGAAAAAGGAGCGGGTACAGGGTGAACCGTATACCCTCCACTAGCCAGTAATCT

GGCCCACTCCGGGCCATCTGTAGATTTAGCCATTTTCTCCTTACATTTAGCAGGTGCAGC

CTCAATTCTCGGGGCCATCAACTTCATTACCACAATAATTAATATGCGGTGAAAAGGAAT

ACGATTAGAGCGTATTCCCTTATTTGTTTGATCAGTAATTATTACTGTTATCCTTCTACT

TCTAACTCTTCCAGTACTAGCAGGAGCCATTACTATGCTCCTAACGGACCGAAATCTAAA

TACATCATTCTTTGATCCTGCTGGAGGGGGAGATCCTGTTCTATATCAACACTTATTC

>T22_LN810317_Limnodrilus_claparedianus

CACTCTCTACATAGTTTTCGGCCTTTGAGCCGGAATAGTCGGTACTGGAACAAGCCTACT

AATTCGATTTGAGTTAGCTCAACCCGGATCATTCTTAGGCAGGGATCAGTTATATAACAC

TTTAGTGACAGCCCACGGATTCTTAATAATTTTCTTTATAGTAATGCCAATCTTTATTGG

TGGATTTGGAAATTGACTAATTCCCTTAATACTTGGGGCACCAGATATAGCATTCCCACG

ATTAAACAATCTCAGCTTTTGACTAATGCCACCATCATTAATTCTATTAGTATCATCTGC

TGCTGTAGAAAAAGGTGCCGGCACAGGTTGAACTGTATACCCGCCACTGGCAAGAAATTT

AGCACATTCGGGGCCATCTGTAGATTTAGCAATTTTCTCCCTTCATCTTGCTGGTGCAGC

TTCAATTCTAGGGGCAATTAACTTTATTACAACAATAATTAACATGCGATGAAAAGGAAT

ACGCCTCGAACGAATTCCATTATTCGTATGATCTGTAATTATTACTGTTATTCTACTACT

TCTGACCCTTCCAGTACTTGCGGGGGCTATCACGATACTATTAACAGACCGAAATCTAAA

TACATCGTTCTTCGACCCTGCGGGGGGAGGAGACCCTGTACTTTATCAACACTTATTC

>T22_991_Limnodrilus_claparedianus_5ind

CACTCTCTACATAATTTTCGGCCTTTGAGCCGGAATAGTCGGTACTGGAACAAGCCTACT

AATTCGATTTGAGTTAGCTCAACCCGGGTCATTCTTAGGCAGAGACCAGTTATATAACAC

TTTAGTGACAGCCCACGGATTCTTAATAATTTTCTTTATGGTAATGCCAATCTTTATCGG

TGGATTTGGAAATTGACTAATTCCCTTAATACTTGGGGCACCAGATATAGCATTCCCACG

ATTAAATAATCTCAGCTTTTGACTAATGCCACCATCGTTAATTCTATTAGTGTCATCTGC

TGCTGTAGAAAAAGGTGCTGGCACAGGTTGAACTGTATATCCGCCACTGGCAAGAAATTT

AGCACATTCGGGGCCATCTGTAGATTTAGCAATTTTCTCCCTTCATCTTGCTGGTGCAGC

TTCAATTCTAGGGGCAATTAACTTTATTACAACAATAATTAACATGCGATGAAAAGGAAT

ACGCCTCGAACGAATTCCATTATTCGTATGATCTGTAATTATTACTGTTATTCTACTACT

TCTGACCCTTCCGGTACTTGCGGGGGCTATCACGATACTATTAACAGACCGAAATCTAAA

TACATCGTTCTTCGACCCTGCGGGGGGGGGAGACCCTGTACTTTATCAACACTTATTC

>T22_765_Limnodrilus_claparedianus_3ind

CACTCTCTACATAGTTTTCGGCCTTTGAGCCGGAATAGTCGGTACTGGAACAAGCCTACT

AATTCGATTTGAGTTAGCTCAACCCGGATCATTCTTAGGCAGAGACCAGTTATATAACAC

TTTAGTGACAGCCCACGGATTCTTAATAATTTTCTTTATAGTAATGCCAATCTTTATTGG

TGGATTTGGAAATTGATTAGTTCCTTTAATACTTGGGGCACCAGATATAGCATTCCCACG

ATTAAATAATCTCAGCTTTTGACTAATGCCGCCATCATTAATTCTATTAGTGTCATCTGC

TGCTGTAGAAAAAGGTGCCGGTACAGGTTGAACTGTATATCCACCCCTGGCAAGAAATTT

AGCACATTCAGGACCATCTGTAGATTTAGCAATTTTCTCCCTTCATCTTGCTGGTGCAGC

TTCAATTCTAGGGGCAATTAACTTTATTACAACAATAATTAACATGCGATGAAAAGGAAT

ACGCCTCGAACGAATTCCATTATTCGTATGATCTGTAATTATTACTGTTATTCTACTACT

TCTAACCCTTCCAGTACTTGCGGGAGCTATCACGATGCTGTTAACAGACCGAAATCTAAA

TACATCGTTCTTCGACCCTGCGGGGGGGGGAGACCCTGTACTTTATCAACACTTATTC

>T22_1047_Limnodrilus_claparedianus_2ind

CACTCTCTACATAGTTTTCGGCCTTTGAGCCGGAATAGTTGGTACTGGGACAAGCCTACT

AATTCGATTTGAACTAGCTCAACCCGGATCATTTTTAGGCAGGGACCAGTTATATAACAC

TTTAGTGACAGCCCACGGATTCTTAATAATTTTCTTTATAGTGATGCCCATCTTTATTGG

TGGGTTCGGAAATTGATTAATTCCCTTAATACTTGGGGCACCAGATATAGCATTCCCACG

ATTAAATAATCTCAGCTTTTGACTAATGCCACCATCATTAATTCTATTAGTATCATCTGC

TGCTGTAGAAAAAGGTGCCGGCACAGGTTGAACTGTATATCCACCCCTGGCAAGAAATTT

AGCACATTCAGGGCCATCTGTAGATTTAGCAATTTTCTCCCTTCATCTTGCTGGTGCAGC

TTCAATTCTAGGGGCAATTAACTTTATTACAACAATAATTAACATACGATGAAAAGGAAT

GCGCCTCGAACGAATTCCATTATTTGTATGATCTGTAATTATTACTGTTATTCTACTACT

TCTAACCCTTCCAGTACTTGCGGGAGCTATCACGATGCTATTAACAGACCGAAATCTAAA

TACATCATTCTTCGACCCTGCGGGGGGAGGAGACCCTGTACTTTATCAGCACTTATTC

>T5_LN810299_Branchiura_sowerbyi_5ind

AACACTATACATAGTATTCGGCCTATGAGCCGGAATAGTGGGAACAGGAACCAGAATTCT

AATTCGAACTGAACTAACTCAACCAGGATCCTTTCTTGGAAGAGATCAACTATATAACAC

CCTAGTGACAGCTCACGGATTCTTAATAATTTTCTTTATAGTAATACCAATCTTTATTGG

GGGTTTTGGGAACTGATTATTACCTTTAATACTCGGGGCACCAGATATAGCATTTCCCCG

AATAAATAATCTTAGATTCTGATTAATACCACCATCGTTAATTCTTCTTGTATCATCAGC

CGCAGTAGAAAAAGGTGCCGGTACAGGCTGAACAGTATATCCACCACTTTCCAGAAATCT

AGCCCATTCTGGACCATCAGTAGATTTAGCCATTTTTTCACTCCACTTAGCTGGTGCCTC

ATCAATTTTAGGGTCAATTAATTTTATCACCACAATAATTAACATACGATCAAAAGGAAT

ACGACTAGAACGAATCCCCTTATTTGTATGAGCTGTAATTATTACAACCATTCTTCTAGT

ATTAACCCTTCCAGTTCTAGCTGGTGCTATTACAATACTACTTACAGACCGAAATCTTAA

TACGTCATTTTTTGATCCTGCTGGAGGGGGGGATCCTGTTTTATATCAACACCTATTT

>T4_778_Aulodrilus_pluriseta_2ind

AACTCTATACTTCATTTTCGGAATCTGAGCCGGTATAGTTGGTACTGGTACCAGACTATT

AATTCGCTTAGAACTAGCACAACCTGGATCCTTCCTAGGAAGAGATCAACTATATAATAC

ATTAGTAACAGCCCACGCATTTCTAATAATCTTCTTCCTAGTTATACCTGTATTTATCGG

GGGATTCGGAAATTGATTAATCCCATTAATACTAGGTGCTCCCGATATAGCATTTCCACG

ACTCAATAACCTAAGATTTTGACTAATACCACCTTCACTAATTTTACTAGTCTCATCTGC

AGCTGTAGAAAAAGGTGCCGGAACAGGTTGAACAGTATACCCACCACTTGCTGGCAATCT

AGCCCACTCAGGACCTTCTGTAGACCTCGCAATCTTCTCTCTACACTTAGCAGGAGTAGC

ATCAATTTTAGGTGCTATTAACTTCATTACCACCATAATTAACATGCGATGAAAAGGGAT

ACGCCTAGAACGAATTCCTCTATTTGTATGAGCCGTAATTCTTACCGTTGTACTCCTTCT

ATTAACCCTACCAGTTCTTGCAGGAGCAATCACTATACTTTTAACTGACCGCAATCTAAA

CACCTCCTTCTTCGATCCGGCTGGAGGTGGTGACCCTGTTCTATACCAACATCTATTC

>T4_1004_Aulodrilus_pluriseta_2ind

AACTCTATACTTCATTTTTGGAATCTGAGCCGGTATAGTTGGTACCGGTACCAGACTATT

AATCCGCTTAGAACTGGCACAACCTGGATCCTTCTTAGGAAGTGATCAACTATACAATAC

ATTAGTAACAGCTCACGCATTTCTAATAATCTTCTTCCTAGTTATACCTGTATTCATCGG

GGGATTCGGAAATTGATTAATCCCATTAATACTAGGTGCTCCTGATATAGCATTTCCACG

ACTAAATAACCTAAGATTTTGACTAATACCCCCCTCACTAATTCTACTAGTTTCATCAGC

AGCTGTAGAAAAAGGTGCAGGGACAGGTTGAACAGTATATCCACCACTTGCTGGCAATCT

AGCCCATTCAGGACCTTCTGTAGATCTAGCAATCTTCTCTCTACACTTAGCGGGAGTAGC

ATCAATTCTAGGTGCTATTAACTTCATTACCACTATAATCAACATACGATGAAAAGGAAT

GCGCCTAGAACGAATTCCTCTATTTGTCTGAGCCGTAATTCTTACCGTTGTACTCCTTCT

ATTAACCTTACCAGTTCTGGCGGGGGCAATTACTATACTTCTAACTGACCGCAATCTAAA

CACCTCCTTCTTCGATCCAGCTGGGGGCGGTGACCCTGTATTATACCAACATCTATTC

>T4_843_Aulodrilus_pluriseta_2ind

AACTCTATACTTCATTTTCGGAATCTGAGCCGGTATAGTTGGTACCGGTACCAGACTATT

AATTCGCTTAGAACTGGCACAACCTGGATCCTTCTTAGGAAGTGATCAACTATATAATAC

ATTAGTAACAGCTCACGCATTTCTAATAATCTTCTTCCTAGTTATACCTGTATTCATCGG

GGGATTCGGAAATTGATTAATCCCATTAATACTAGGTGCCCCTGATATAGCATTTCCACG

ACTAAATAACCTAAGATTTTGACTAATACCGCCCTCACTAATTCTACTAGTTTCATCCGC

AGCTGTAGAAAAAGGTGCAGGGACAGGTTGAACAGTATATCCACCACTTGCTGGCAATCT

AGCCCATTCAGGGCCTTCAGTAGATCTGGCAATCTTCTCTCTACACTTAGCAGGGGTAGC

ATCAATTCTAGGTGCTATTAACTTCATTACCACTATAATCAACATACGATGAAAAGGAAT

GCGCCTAGAACGAATTCCTCTATTTGTCTGAGCCGTAATTCTTACCGTTGTACTCCTTCT

ATTAACCTTACCAGTTCTGGCAGGGGCAATTACTATACTTCTAACTGACCGCAATCTAAA

CACCTCCTTCTTCGATCCAGCTGGGGGCGGTGACCCTGTATTATACCAACATCTATTC

>T4_942_Aulodrilus_pluriseta_3ind

AACTCTATACTTCATTTTCGGAATCTGGGCCGGTATAGTTGGTACTGGTACCAGACTATT

AATTCGCTTAGAACTGGCACAACCTGGATCCTTCTTAGGAAGTGATCAACTATATAATAC

ATTAGTAACAGCTCACGCATTTCTAATAATCTTCTTCCTAGTTATACCTGTATTCATCGG

GGGATTCGGAAATTGATTAATCCCATTAATACTAGGTGCCCCTGATATAGCATTCCCACG

ACTAAATAACCTAAGATTTTGACTAATACCACCCTCACTAATTCTACTAGTTTCATCCGC

AGCTGTAGAAAAAGGTGCGGGGACAGGTTGAACAGTATATCCACCACTTGCTGGCAATCT

AGCCCATTCAGGACCTTCCGTAGATCTGGCAATCTTCTCTCTACACTTAGCAGGGGTAGC

ATCAATTCTAGGTGCTATTAACTTCATTACCACTATAATCAACATACGATGAAAAGGAAT

GCGCCTAGAACGAATTCCTCTATTTGTCTGAGCCGTAATTCTTACCGTTGTACTCCTTCT

ATTAACCTTACCAGTTCTGGCGGGGGCAATTACTATACTTCTAACTGACCGCAATCTAAA

CACCTCCTTCTTCGATCCAGCTGGGGGCGGTGACCCTGTATTATACCAACATCTATTC

>T23_929_Limnodrilus_udekemianus

CACCCTTTATTTTATCTTCGGCCTATGAGCCGGTATAGTCGGTACCGGAACCAGATTATT

AATTCGATTTGAACTAGCACAACCCGGATCATTTCTAGGTAGAGACCAACTCTATAACAC

ACTAGTAACAGCACATGGATTTCTAATAATTTTCTTCCTTGTAATACCAGTATTTATTGG

GGGATTCGGCAATTGACTAGTACCATTAATACTTGGGGCTCCCGACATAGCATTCCCACG

ACTAAATAATCTTAGATTTTGACTAATACCTCCATCACTTATTCTACTTGTATCCTCAGC

AGCCGTAGAAAAAGGGGCTGGAACAGGGTGAACTGTATATCCTCCATTAGCAGGGAATCT

TGCTCATTCAGGCCCTTCTGTAGACCTTGCCATTTTTTCCCTTCATCTGGCCGGAATTTC

TTCAATTTTAGGGGCTATTAACTTCATCACCACTATAATCAATATACGATGAAAAGGAAT

ACGACTTGAACGAATTCCTTTATTTGTGTGATCTGTAATTATCACTGTAGTCTTACTTCT

ACTAACTCTCCCTGTATTAGCAGGTGCAATTACAATACTTCTAACAGATCGAAATCTGAA

TACATCATTCTTTGATCCTGCCGGAGGAGGTGACCCTGTCCTATATCAACATCTATTC

>T23_1016_Limnodrilus_udekemianus

CACCCTTTACTTTATCTTCGGCCTATGAGCCGGTATAGTTGGTACAGGTACCAGACTATT

AATTCGATTTGAGCTAGCACAACCCGGATCATTCCTAGGCAGAGACCAACTCTATAACAC

GCTAGTAACAGCACATGGATTTCTAATAATCTTCTTCCTTGTAATACCAGTATTTATTGG

GGGATTCGGTAATTGATTAGTACCATTAATACTTGGGGCTCCTGACATAGCATTCCCACG

ATTAAATAATCTAAGATTTTGATTAATACCTCCATCACTTATTCTACTTGTATCCTCAGC

AGCAGTGGAAAAAGGAGCTGGAACAGGATGAACTGTATACCCTCCATTAGCAGGAAATCT

TGCCCATTCCGGACCTTCTGTAGATCTTGCTATTTTCTCTCTTCACCTGGCTGGAATTGC

CTCAATTCTAGGAGCTATCAACTTTATCACCACTATAATTAATATACGATGAAAAGGAAT

ACGACTCGAACGAATTCCTTTATTTGTGTGATCTGTAATTATCACTGTAGTCTTACTTCT

ACTAACTCTCCCTGTATTAGCAGGGGCAATTACAATGCTCCTAACAGATCGAAATCTAAA

TACATCATTTTTTGATCCTGCCGGAGGAGGAGATCCTGTCCTATATCAACACTTATTC

>T23_LN810320_Limnodrilus_udekemianus

----------------------CTGTGAGCCGGTATAGTTGGCACAGGTACCAGACTATT

AATTCGATTTGAACTAGCACAACCCGGATCATTCCTAGGCAGAGACCAACTCTATAATAC

ACTAGTAACAGCACATGGATTTCTAATAATTTTCTTCCTTGTAATACCAGTATTTATTGG

GGGATTCGGTAATTGACTAGTACCATTAATACTTGGGGCCCCTGATATGGCATTTCCACG

ACTAAACAACCTAAGATTTTGATTAATACCTCCATCACTTATTCTACTTGTATCCTCAGC

AGCAGTAGAAAAAGGGGCCGGAACAGGATGAACTGTATATCCGCCATTAGCAGGAAATCT

TGCCCATTCCGGGCCTTCCGTGGACCTCGCTATTTTCTCTCTCCATTTAGCTGGAATTGC

CTCAATTCTAGGAGCTATTAATTTTATCACCACTATAATTAACATACGATGAAAAGGAAT

GCGACTCGAACGAATTCCTCTATTTGTGTGATCCGTAATTATCACTGTAGTCTTACTTCT

ATTAACTCTTCCTGTATTAGCAGGGGCGATTACAATACTCCTAACAGATCGAAATCTAAA

TACATCATTTTTTGATCCTGCCGGAGGAGGGGATCCTGTCCTATATCAACACTTATTT

>T31_847_Potamothrix_heuscheri_4ind

GCTTCTATATATAATATATGGCCTATGAACAGGTATAGTGGGAACCGGGACTAGAATTCT

AATTCGTATAGAACTAGCTCAACCTGGATCATTTCTAGGCAGGGACCAACTATATAATAC

ACTTGTAACTGCTCATGCCTTTTTAATAATTTTCTTCTTAGTAATACCAGTATATATTGG

GGCTTTTGGAAACTTTCTTGTTCCTTTAATACTAGGTGCACCAGATATAGCATTCCCTCG

AATAAACAATCTTAGATTCTGATTAATACCATCTTCTGTAATCTTATGTGTAGCTTCTGC

TGCCGTAGAAAAAGGGGCCGGTACAGGATGAACAGTATATCCTCCATTAGCAAGAAATCT

TGCTCACTCTGGACCATCTGTAGATCTTGCCATTTTCTCTCTTCACTTAGCGGGGATCTC

CTCTATTCTAGGCGCAATTAATTTCATTACTACGATAATTAATATACGATGAAAAGGAAT

GCGCCTAGAACGAATTCCTTTATTTGTATGAGCTACAATTATTACAGTAGTCTTACTACT

ACTAACACTTCCAGTTCTAGCTGGGGCTATTACTATGCTTCTAACAGACCGAAACCTGAA

TACCTCATTCTTTGATCCTGCAGGTGGAGGGGATCCTGTCCTGTACCAACATTTATTC

>T2_838_Tubificinae_sp_8ind

ACTTCTCTATATAATGTATGGCCTATGAACAGGTATGGTAGGAACAGGAACTAGAATTCT

AATTCGAATAGAACTTGCCCAACCAGGATCATTCCTTGGGAGAGACCAGCTATATAATAC

ACTCGTAACTGCCCATGCTTTCTTAATAATTTTCTTCCTAGTAATGCCCGTATACATTGG

TGCTTTTGGAAACTTCCTGGTACCATTAATACTTGGCGCACCAGATATAGCATTTCCACG

AATAAATAATCTCAGATTCTGATTAATACCTTCCTCTGTAATTTTATGTGTAGCATCTGC

TGCCGTTGAAAAAGGAGCCGGTACAGGTTGAACCGTTTACCCTCCATTAGCAAGAAATCT

TGCTCACTCTGGCCCATCTGTAGACTTAGCCATCTTCTCCCTTCACTTAGCAGGTATTTC

CTCTATTTTAAGAGCAATTAACTTCATTACTACAATAATCAATATACGATGAAAAGGAAT

ACGACTTGAACGAATTCCTTTATTTGTATGAGCTACAATCATTACAGTAATTCTACTACT

ATTAACACTTCCAGTTCTAGCTGGGGCTATCACCATATTACTAACAGACCGAAATCTAAA

TACCTCATTCTTTGACCCTGCAGGAGGGGGAGACCCTGTGCTTTATCAACATTTATTC

>T29_848_Potamothrix_vejdovskyi

ATTCCTTTACATAATATATGGTTTATGAACAGGTATAGTAGGAACGGGAACTAGAATTTT

AATCCGGATAGAGCTAGCTCAACCGGGATCATTCCTTGGGAGTGACCAGCTATATAATAC

TCTTGTAACTGCTCACGCTTTTCTGATAATCTTCTTCTTAGTGATACCTGTTTATATCGG

CGCATTTGGGAACTTTCTTATACCTTTAATACTCGGGGCACCTGACATAGCATTTCCACG

ACTTAATAATTTAAGATTTTGATTGATACCTTCATCCGTAATTCTATGCGTAGCCTCTGC

AGCTGTAGAAAAGGGAGCAGGTACAGGCTGAACAGTATACCCTCCTCTAGCTAGGAACCT

TGCTCATTCTGGACCGTCTGTGGATTTAGCCATTTTCTCTTTACATTTAGCAGGTATCGC

CTCTATTTTAGGTGCAATCAATTTTATTACAACCATAATTAATATGCGGTGAATAGGAAT

ACGGTTAGAACGAATTCCTTTATTTGTATGAGCTACAATTATTACAGTTCTCCTTCTATT

ACTTACCCTACCTGTTCTGGCGGGGGCTATTACTATACTCTTAACAGATCGAAATCTAAA

TACCTCATTCTTTGACCCTGCGGGAGGGGGAGACCCTGTACTATATCAGCATCTATTC

>T28_834_Potamothrix_hammoniensis

ACTCCTTTATATAATATATGGACTATGAACAGGTATGGTGGGAACTGGAACTAGAATACT

AATCCGAATAGAATTAGCTCAACCTGGATCCTTCCTTGGAAGGGACCAACTATATAATAC

TCTTGTAACTGCTCATGCCTTCTTAATAATTTTCTTCTTGGTTATACCGGTATATATTGG

TGCCTTCGGAAACTTTCTTGTACCTTTAATACTAGGTGCACCTGATATAGCTTTCCCCCG

AATAAATAATTTAAGATTTTGACTTATACCTTCATCTGTAATTCTATGCGTAGCATCTGC

AGCAGTTGAAAAAGGAGCAGGAACAGGTTGAACAGTATACCCGCCCTTAGCAAGAAATCT

TGCTCACTCGGGACCATCTGTAGATTTAGCAATCTTCTCTTTACATTTAGCCGGAATTTC

CTCTATTTTAGGGGCAATCAATTTTATTACTACAATAATTAACATACGTTGAAAAGGAAT

GCGATTAGAACGAATTCCTTTATTTGTGTGAGCGACAATCATTACTGTTCTTCTTCTATT

ATTAACTCTACCAGTTCTTGCTGGTGCTATTACCATACTATTAACAGATCGAAATCTAAA

TACCTCATTCTTTGATCCTGCTGGTGGTGGGGACCCTGTTCTATATCAACACCTATTC

>T28_855_Potamothrix_hammoniensis

ACTCCTTTATATAATGTACGGACTATGAACAGGTATGGTGGGAACTGGAACTAGAATACT

AATTCGAATAGAACTAGCTCAACCGGGGTCCTTCCTTGGAAGGGACCAACTATACAATAC

CCTTGTAACTGCTCATGCCTTCTTAATAATTTTCTTCTTAGTCATGCCAGTATATATTGG

TGCCTTCGGAAACTTTCTTGTACCTTTAATACTAGGGGCCCCTGATATGGCTTTCCCCCG

AATAAATAATTTAAGATTTTGACTTATACCCTCATCTGTAATTCTATGTGTAGCATCTGC

AGCAGTTGAAAAAGGAGCAGGAACAGGTTGAACAGTATACCCCCCCTTAGCAAGAAATCT

TGCTCACTCGGGGCCATCTGTAGATTTAGCAATCTTCTCTTTACATTTAGCAGGAATTTC

CTCTATTTTAGGAGCAATCAATTTTATTACTACAATAATTAACATGCGTTGAAAAGGTAT

ACGATTAGAACGAATTCCTTTATTTGTGTGAGCAACAATCATTACTGTTCTTCTTCTATT

ATTAACTCTACCCGTTCTTGCTGGTGCTATTACCATACTATTAACAGATCGAAATCTAAA

TACCTCATTCTTCGATCCTGCTGGTGGCGGGGACCCTGTTCTATATCAACACCTATTC

>T30_860_Potamothrix_moldaviensis

ACTTCTTTATATCTTCTCTGGACTATGAACAGGTATGGTAGGAACAGGAACTAGAATTTT

AATTCGTCTGGAACTTGCTCAGCCTGGATCATTCCTAGGTAGTGATCAGTTATATAACAC

TCTCGTAACTGCCCACGCCTTTCTGATAATCTTTTTTTTAGTAATACCTGTATATATTGG

AGCTTTCGGGAACTTCCTAATTCCACTAATACTAGGGGCTCCTGATATAGCTTTCCCACG

ATTAAACAATTTAAGATTTTGACTTATACCCTCATCTGTAATCCTGTGTGTGGCATCTGC

AGCTGTTGAAAAGGGGGCAGGAACAGGTTGAACAGTATACCCTCCCCTAGCAAGAAACCT

TGCCCATTCCGGGCCTTCGGTAGACCTGGCCATTTTCTCTCTTCATCTAGCAGGAATTGC

TTCCATTTTAGGCGCAATTAACTTCATTACAACAATAATTAATATACGTTGAAAAGGAAT

ACGGTTAGAGCGAATCCCCTTATTCGTTTGAGCGACAATCATTACAGTTCTTTTACTACT

ATTAACTCTACCCGTATTAGCGGGCGCTATTACCATACTATTAACAGACCGAAATCTAAA

TACCTCATTCTTCGATCCTGCTGGGGGAGGAGACCCTGTCTTATATCAACACCTATTT

>T30_752_Potamothrix_moldaviensis_3ind

ACTTCTTTATATATTATATGGACTATGAACAGGTATGGTGGGAACAGGAACTAGAATTTT

AATTCGTCTGGAACTTGCTCAGCCTGGGTCATTCCTAGGTAGTGATCAGTTATATAACAC

CCTCGTAACTGCTCACGCCTTTCTGATAATCTTTTTTCTAGTAATACCTGTATATATTGG

AGCTTTCGGGAACTTCCTAATCCCACTAATACTAGGGGCTCCTGATATAGCTTTCCCACG

GTTAAACAATTTAAGATTTTGACTTATACCTTCATCTGTAATCCTCCTTGTGGCATCTGC

AGCTGTTGAAAAGGGAGCAGGAACAGGTTGAACAGTGTACCCTCCCCTAGCAAGAAACCT

TGCCCATTCCGGGCCTTCGGTAGACCTGGCCATTTTCTCTCTCCATCTAGCAGGAATTGC

TTCCATTTTAGGGGCAATTAACTTCATTACAACAATAATTAATATACGTTGAAAAGGGAT

ACGATTAGAGCGAATCCCCTTATTCGTTTGAGCGACAATCATCACAGTTATTTTACTACT

ATTAACTCTACCCGTATTAGCGGGCGCTATTACCATACTATTAACAGACCGAAATCTAAA

TACCTCATTCTTCGATCCTGCTGGGGGAGGAGACCCTGTCTTATATCAACACCTATTT

>T1_LN810329_Tubificinae_sp

ACTCCTTTATATAATATATGGGCTATGAACAGGTATAGTTGGCACAGGGACTAGAATTTT

AATTCGAATAGAACTTGCTCAACCGGGATCATTTTTAGGGAGAGATCAACTATATAACAC

CCTTGTAACTGCCCACGCCTTTCTAATAATTTTCTTTCTCGTAATACCAGTATATATTGG

AGCCTTCGGAAACTTCCTTGTCCCATTAATACTAGGAGCTCCTGATATAGCATTTCCGCG

ATTAAATAATTTAAGATTTTGACTAATACCTTCATCAGTAATCTTATGTGTAGCTTCCGC

CGCAGTTGAAAAGGGGGCGGGCACAGGCTGAACAGTATATCCTCCATTAGCAAGAAACCT

TGCTCATTCCGGACCCTCAGTCGATTTAGCTATTTTCTCGCTTCACTTAGCAGGAATCGC

CTCCATTCTAGGTGCAATCAACTTTATCACAACTATGATCAATATACGATGAAAAGGTAT

ACGACTAGAACGAATTCCTTTATTTGTATGAGCTACAATTATTACAGTTCTTCTCCTTTT

ATTAACTCTTCCTGTCTTAGCCGGTGCTATTACCATACTACTAACAGACCGAAATCTAAA

TACCTCATTCTTTGATCCTGCTGGTGGTGGAGACCCAGTCTTATACCAACATCTATTC

>T7_715_Potamothrix_bavaricus_48ind

ACTCCTTTATATAATGTATGGCCTATGAACAGGCATGGTAGGAACTGGAACTAGACTACT

AATTCGAATGGAACTTGCTCAACCAGGATCATTCCTTGGCAGAGATCAACTCTATAACAC

TCTCGTAACCGCACACGCATTTCTTATAATCTTTTTCCTTGTTATACCTGTATATATTGG

TGCCTTCGGAAACTTCCTCGTCCCATTAATACTTGGCGCCCCTGATATAGCATTTCCACG

GCTAAATAACTTAAGATTTTGACTAATACCCTCATCTGTAATTCTATGCGTGTCATCAGC

TGCTGTTGAAAAAGGAGCCGGTACTGGTTGAACAGTATACCCCCCATTAGCAAGAAATCT

TGCTCATTCAGGCCCATCTGTCGACTTAGCTATTTTCTCTCTTCACTTAGCAGGTATCTC

CTCTATTCTAGGGGCGATTAACTTTATTACCACTATAATTAATATGCGTTGAAAAGGAAT

GCGCCTAGAACGAATTCCATTATTTGTATGAGCTACTATTATTACAGTAGTATTACTCCT

ACTAACTCTTCCTGTCCTGGCCGGAGCTATTACTATACTTCTAACCG-------------

----------------------------------------------------------

>T3_LN810328_Tubificinae_sp

ACTCCTTTACATAATATATGGCCTATGAACCGGAATGGTAGGAACTGGGACTAGATTACT

AATTCGAATAGAACTAGCTCAACCGGGATCATTCCTGGGTAGAGATCAATTATATAATAC

TCTCGTAACTGCCCATGCATTCCTAATAATTTTCTTCCTTGTAATACCAGTATATATCGG

TGCTTTTGGAAACTTCCTAGTTCCATTAATGCTCGGCGCCCCAGATATAGCTTTCCCCCG

AATAAATAATCTAAGATTCTGATTAATACCTTCTTCTGTGATCTTATGCGTAGCATCTGC

TGCTGTGGAAAAAGGGGCTGGAACAGGCTGAACAGTATATCCCCCATTAGCAAGAAATCT

TGCTCATTCTGGGCCCTCTGTGGACCTAGCTATTTTTTCCCTTCATTTAGCGGGAATTTC

ATCTATTTTAGGCGCAATTAACTTCATTACTACTATAATCAATATACGGTGAAAAGGGAT

ACGATTAGAACGAATTCCCCTGTTTGTGTGAAGTGTGATCATCACAGTAGTACTACTACT

ACTAACTCTTCCAGTCTTAGCTGGGGCTATTACTATACTTTTAACTGACCGTAATCTAAA

TACATCATTCTTCGACCCTGCCGGGGGAGGGGACCCTGTATTATATCAACATTTATTC

>T13_LN810298_Tubifex_montanus

GACACTATATATAATCTTCGGATTCTGAGCCGGAATAGTAGGAACCGGAACAAGTTTACT

AATTCGATTTGAACTAGCTCAACCAGGATCTTTCCTGGGCAGGGATCAACTATATAATAC

TTTAGTTACGGCTCATGCCTTCTTAATAATTTTCTTCCTTGTAATGCCCGTCTTTATTGG

GGGGTTTGGCAATTGATTAGTTCCATTAATGCTAGGGGCACCTGACATAGCCTTCCCTCG

TCTAAATAATCTAAGATTTTGATTACTTCCACCATCTTTAATTCTTCTAGTGTCATCAGC

CGCTGTAGAAAAAGGAGCAGGAACTGGATGAACTGTCTACCCACCATTAGCCGGGAACTT

AGCTCATTCAGGCCCTTCAGTAGATTTAGCCATTTTTTCACTTCATTTAGCAGGAATTGC

ATCAATTCTAGGAGCTATTAACTTCATTACTACAATAATCAACATACGATGAAAAGGAAT

GCGTTTAGAACGAATCCCATTATTTGTATGAGCTGTTATTTTAACAGTAATTCTCCTCCT

ACTAACTCTTCCTGTTCTAGCAGGAGCAATTACTATATTACTAACAGATCGAAATCTAAA

TACATCATTTTTTGATCCTGCAGGTGGGGGGGACCCCGTTCTCTACCAACATTTATTC

>LC3_LN810249_Eiseniella_tertraedra

------------CATTTTAGGTATTTGAGCTGGGATAGTGGGAGCTGGTATAAGCTTACT

AATTCGAATCGAATTAAGCCAACCAGGAGCCTTCCTAGGTAGTGACCAATTATATAACAC

TATTGTTACAGCACATGCATTCGTAATAATCTTCTTCCTAGTAATACCCGTATTCATTGG

TGGATTCGGAAACTGATTGCTACCTCTAATACTAGGTGCACCAGATATAGCATTTCCACG

TTTAAACAACATAAGATTTTGACTTTTACCTCCTTCTTTAATTCTACTAGTATCTTCCGC

AGCCGTAGAAAAGGGGGCCGGGACAGGGTGAACTGTTTATCCGCCCTTAGCAAGAAACTT

GGCCCATGCAGGACCATCAGTAGATCTAGCTATTTTCTCCTTACACTTAGCAGGAGCTTC

ATCTATTTTAGGTGCCATCAACTTTATTACTACAGTCATTAATATGCGATGAAGGGGCTT

ACGATTAGAACGAATTCCCCTTTTCGTGTGAGCTGTACTGATTACAGTAATTCTTCTATT

ATTATCGTTGCCCGTGCTAGCAGGAGCAATTACCATACTATTAACCGACCGAAATCTCAA

TACATCATTCTTTGACCCGGCTGGTGGTGGTGACCCAATTCTGTACCAACACCTCTTC

>E3_LN810245_Lumbricillus_rutilus

-ACACTATATTTTATTTTAGGAGTATGAGCTGGAATACTAGGAGCAGCCATAAGACTTTT

AATTCGAATTGAATTAAGACAACCTGGCGCTTTTTTAGGAAGAGATCAGCTTTATAATAC

TATCGTAACAGCTCATGCATTCTTAATAATTTTTTTCTTAGTTATACCAGTATTTATTGG

TGGATTTGGAAATTGATTAATTCCGCTAATATTGGGAGCTCCTGACATAGCATTCCCTCG

TCTTAACAATATAAGATTTTGACTTCTACCTCCAGCTCTTTTACTTCTAGTTTCTTCAGC

AGCAGTAGAAAAAGGTGCTGGGACTGGCTGAACAGTTTACCCACCTCTAGCAAGAAATCT

AGCTCATGCAGGTCCATCCGTAGATTTAGCAATTTTCTCTCTTCATTTAGCCGGTGCCTC

ATCTATTCTTGGAGCAGTAAACTTTATTACTACAGTAGTAAATATACGTTGACAAGGTCT

TCGACTTGAACGAATTCCTCTTTTTGTATGAGCAGTAGTAATTACAACAGTTCTTCTTCT

TCTATCTCTTCCAGTTCTTGCAGGGGCAATTACAATACTACTAACTGATCGAAATCTAAA

CACTTCATTTTTTGACCCAGCCGGAGGTGGAGATCCTGTTCTTTATCAACATTTATTT

>H1*_885_Haplotaxis_gordioides_2ind

AACCCTTTACTTTATCCTAGGCATCTGAGGGGGACTTCTAGGAACAAGAATAAGAATAGT

AATCCGAATTGAACTAAGACAACCAGGGTCATTCCTTGGTAGAGATCAATTATACAATAC

CATTGTTACTGCCCATGCCTTCCTAATAATTTTCTTTCTTGTCATACCAGTATTTATTGG

AGGGTTCGGAAACTGACTCTTACCTCTAATGTTAGGAGCCCCCGACATAGCATTCCCACG

ACTCAACAATATAAGATTTTGACTTCTACCTCCTGCAACAATCCTTCTTGTGTCCTCAGC

AGCAGTAGAAAAAGGAGCAGGAACTGGCTGAACTGTTTACCCGCCCTTAGCCAGAAATCT

TGCACATGCTGGCCCTTCTGTTGATCTCGCCATTTTCTCTCTTCATTTAGCAGGAGTTTC

CTCCATTTTAGGAGCAGTAAATTTCATCACCACTGTTGTCAATATACGATGAAATGGCCT

GCGGTTAGAACGAATTCCTTTATTCGTATGATCCGTAACTATTACAGTGGTTCTTCTCCT

TTTATCTCTACCAGTGCTAGCCGGCGCTATTACCATACTACTAACTGACCGAAATCTTAA

TACATCATTCTTCGACCCAGCTGGAGGAGGAGATCCAGTTCTGTATCAACACCTATTC

>LL3_LN810273_Stylodrilus_heringianus

AACCTTATATTTCATCTTAGGAGTTTGAGCTGGGATAGTAGGAGCAGGGATGAGACTATT

AATTCGAGTTGAATTAACACAACCCGGGTCATTTTTAGGAAGTGACCAACTATATAATAC

TATTGTTACGGCTCACGCATTTATTATAATTTTCTTTATAGTTATACCTATATTTATTGG

TGGTTTTGGTAACTGAATACTACCGTTAATATTAGGAGCACCAGACATAGCATTTCCACG

ACTTAACAACCTAAGATTTTGATTACTACCACCTTCTTTAACTTTATTAGTAGCATCGGC

TGCAGTAGAAAAAGGAGCCGGCACAGGATGAACAGTTTATCCACCTCTATCAAGAAATTT

AGCTCATGCTGGACCCTCAGTAGACCTGGCAATCTTCTCACTCCATCTAGCAGGAGCCTC

CTCCATTTTAGGAGCCATTAACTTTATTACGACTGTAATTAATATACGATGAAATGGACT

ACGACTTGAACGAGTTCCATTATTTGTATGAGCAGTAACAATTACAGTAGTTCTATTACT

ATTATCATTACCAGTACTAGCTGGAGCAATCACTATACTTCTTACAGATCGAAACTTAAA

TACAACATTTTTTGATCCAGCTGGAGGTGGAGACCCCGTACTATATCAACATTTATTT

>N5_LN810257_Ophidonais_serpentina

TACATTATATTTAATCTTAGGAGTATGAGCAGGAATAGTTGGTACAGGAACAAGAATACT

GATTCGAATTGAACTAGCTCAACCAGGAGCTTTTCTAGGAAGAGATCAATTATATAACAC

TCTAGTAACAGCACATGCGTTTTTAATAATTTTCTTTTTAGTTATACCTGTATTTATTGG

CGGATTCGGAAACTGACTTCTTCCATTAATATTAGGTGCTCCAGATATGGCATTCCCACG

ACTAAATAATCTTAGATTCTGACTTCTACCACCATCATTAATTCTATTAATTTCATCTGC

AGCCGTTGAAAAAGGTGCTGGAACAGGATGAACTGTATATCCTCCATTATCAAGAAATCT

AGCTCACGCTGGACCTTCAGTTGACATGGCTATTTTTTCACTACATCTAGCAGGTGCATC

TTCTATTTTAGGTGCAGTTAACTTCATTACTACAGTAATAAACATACGATGAAATGGAAT

ACGACTTGAACGAGTACCATTATTTGTATGAGCTGTAACACTTACTGTAATTCTTCTTCT

TTTATCATTACCTGTATTAGCTGGTGCAATTACCATACTATTAACAGATCGAAATCTAAA

TACCTCATTCTTCGATCCTGCAGGAGGGGGAGACCCAATTTTATACCAACATTTATTC

**Supplemental Fig. 2**

>R1_LN810295_Bothrioneurum_vejdovskyanum_2ind

CACTCTATACCTAATCTTCGGCGTATGAGCTGGAATGGTAGGAACTGGAACAAGACTCCT

AATTCGAATCGAACTAGCTCAACCAGGATCATTCCTAGGCAGAGACCAGCTATTCAACAC

TCTGGTCACAGCCCATGCATTCCTGATAATCTTCTTCTTTGTAATACCAGTATTCATTGG

CGGCTTTGGCAACTACTTAATCCCACTAATACTAGGCGCACCAGACATGGCGTTCCCACG

ACTCAACAACATAAGATTCTGACTCCTACCCCCATCCCTAATTCTCCTAGTATCATCCGC

AGCAGTAGAAAAAGGAGCCGGTACAGGATGAACAGTGTATCCACCCCTAGCAAGAAACCT

TGCACACTCCGGCCCATCAGTAGATCTAGCAATTTTCTCCCTCCACTTAGCCGGGGCATC

CTCAATCCTAGGCGCTATTAACTTCATCACCACAATAATCAACATACGCTGAAACGGACT

TCGACTTGAACGAATCCCCCTATTCGTCTGAGCAGCAGTAATTACAGTAATCCTACTCCT

ACTATCACTTCCAGTACTAGCTGGGGCTATTACCATACTATTAACAGACCGCAACCTAAA

TACATCCTTCTTTGACCCAGCTGGAGGGGGCGACCCTATTCTATACCAACATCTATTC

>R1_LN810296_Bothrioneurum_vejdovskyanum

CACCCTGTACCTAATCTTCGGCGTATGAGCCGGAATAGTGGGAACTGGAACAAGACTCCT

AATTCGAATCGAATTAGCTCAACCAGGATCATTCCTAGGCAGAGACCAACTATTCAACAC

CCTAGTCACAGCCCATGCATTCCTAATAATCTTCTTCTTTGTAATACCAGTATTCATCGG

CGGCTTTGGCAACTACTTAATCCCGCTAATACTAGGCGCACCAGACATAGCATTCCCACG

ACTAAACAACATAAGATTCTGACTCCTACCCCCATCCCTAATTCTCCTAGTATCATCCGC

AGCAGTAGAAAAAGGAGCCGGTACAGGATGAACAGTATATCCACCCCTAGCAAGAAATCT

CGCACACTCCGGTCCATCAGTAGACCTGGCAATTTTCTCCCTTCATTTAGCCGGAGCATC

CTCAATCCTAGGTGCTATCAACTTCATCACCACAATAATCAACATACGCTGAAACGGACT

TCGACTTGAACGAATCCCTCTATTCGTCTGAGCAGCAGTAATCACAGTAATCCTACTCCT

ACTATCACTTCCAGTACTAGCTGGGGCCATTACCATACTATTAACAGACCGCAACCTAAA

TACATCCTTCTTCGACCCAGCTGGTGGGGGCGACCCTATTCTATACCAACATCTATTC

>E6_808_Achaeta_sp

--CATTATATTTTATCCTAGGGATCTGAGCAGGTATAATAGGAGCAGCTATAAGCCTACT

AATTCGATTTGAACTAAGACAACCAGGCTCATTTTTAGGAAGAGATCAACTGTACAACAC

AATTGTAACAGCTCATGCATTTTTAATAATCTTCTTCCTAGTAATACCAGTATTTATTGG

GGGATTTGGTAACTGATTACTACCTCTAATGTTAGGTGCACCAGATATAGCATTCCCACG

ACTTAACAACATAAGATTTTGATTATTACCCCCCTCACTTATACTTCTTCTATCCTCAGC

AGCAGTTGAAAAAGGAGCAGGTACCGGATGAACAGTATACCCACCACTAGCCAGAAACAT

TGCACACGCTGGCCCATCAGTAGACCTAGCAATCTTTTCTCTACATCTTGCAGGGGCATC

CTCAATTCTTGGAGCAGTAAACTTCATTACTACAGTAATCAATATACGATGACAAGGAAT

ACGATTAGAACGAATCCCCCTTTTTGTATGAGCAGTAGTAATTACAGTAGTTCTACTACT

ACTATCTCTACCAGTCCTTGCTGGGGCTATTACTATATTACTTACAGATCGCAATCTAAA

CACATCATTTTTTGACCCGGCAGGTGGTGGAGACCCCATTCTATACCAACATTTATTT

>E14_813_Fridericia_sp_2ind

------------------------------------AATAGGAGCCGCAATAAGATTATT

AATTCGAATTGAACTCAGACAACCAGGCTCCTTCCTTGGAAGAGATCAGCTTTATAACAC

TATCGTAACAGCACATGCATTTCTAATAATCTTTTTCTTAGTAATACCAGTATTTATTGG

AGGCTTTGGTAATTGACTTTTACCATTAATATTAGGAGCCCCAGATATAGCATTCCCCCG

ACTAAATAATATAAGATTCTGATTACTACCCCCATCTTTAATACTTCTTCTATCTTCAGC

TGCAGTAGAAAAAGGTGCTGGCACTGGGTGAACTGTATACCCCCCACTAGCAAGAAATAT

AGCTCATTCTGGGCCATCCGTTGACTTAGCAATTTTTTCTCTACATCTTGCAGGAGCATC

TTCAATTCTAGGCGCAGTAAATTTTATTTCCACAGTTATTAATATGCGATGACAAGGCCT

ACAACTAGAACGAATTCCACTATTTGTATGAGCTGTTACAATCACAGTAGTATTACTACT

ACTATCTCTCCCAGTTCTAGCTGGCGCAATTACTATATTATTAACTGATCGAAATCTAAA

TACATCGTTCTTCGACCCTGCTGGTGGAGGAGATCCAATCCTATATCAACACCTCTTT

>E17_1028_Enchytraeus_buchholzi

CACATTATATTTTATTCTAGGAGTGTGGGCCGGAATAATAGGAGCAGCTATAAGCCTATT

AATTCGAATTGAACTTAGACAACCTGGATCATTCTTAGGAAGAGATCAACTATACAACAC

TATTGTAACAGCACATGCATTTCTAATAATTTTCTTCTTAGTTATACCAGTATTTATTGG

GGGCTTTGGTAACTGATTACTCCCATTAATATTGGGGGCCCCTGATATGGCCTTCCCACG

ACTAAACAATATAAGATTTTGATTACTTCCCCCAGCACTTATACTGCTTTTATCATCGGC

AGCAGTAGAAAAAGGGGCAGGTACAGGATGAACTGTGTACCCTCCTCTAGCCAGAAACAT

CGCACATGCAGGCCCATCAGTTGACCTAGCAATTTTTTCTCTTCATTTAGCAGGAGCCTC

ATCAATTTTAGGAGCTGTAAATTTCATCACTACAGTTATTAATATGCGATGACAAGGACT

TACACTAGAACGAATTCCCCTTTTCGTTTGAGCAGTTACAATTACAGTAGTGTTACTACT

CCTATCTTTACCAGTACTAGCTGGAGCAATTACTATGCTACTAACCGATCGAAACCTAAA

TACATCATTTTTCGACCCGGCTGGTGGTGGAGACCCAATTCTCTACCAACATTTATTC

>E16_954_Henlea_perpusilla

AACNCTATATTTCATTCTAGGCGTATGAGCCAGAATGATAGGAGCAGCCATAAGCCTTCT

AATTCGAATTGAACTAAGTCAACCAGGTTCATTCCTCGGAAGAGACCAACTCTACAACAC

TATTGTTACTGCACATGCATTTCTTATAATTTTTTTCCTAGTTATACCTGTATTTATTGG

GGGGTTCGGAAACTGACTTCTCCCATTAATACTAGGGGCCCCAGATATAGCTTTTCCCCG

ACTAAATAACATAAGTTTTTGACTTCTTCCCCCATCACTTCTTCTTCTACTTTCCTCTGC

AGCTGTGGAAAAAGGAGCCGGAACCGGCTGAACTGTATATCCCCCCCTATCAAGAAACAT

CGCTCATGCAGGCCCATCTGTAGACCTAGCCATTTTCTCCCTCCACTTAGCCGGAGCATC

CTCCATCTTAGGTGCAGTAAACTTTATCACTACTGTTATTAATATACGTTGACAAGGCCT

TCACCTAGAACGAATTCCCCTATTTGTATGAGCTGTAACAATTACAGTAGTCCTTCTCCT

CTTATCTTTACCAGTATTAGCCGGAGCAATTACTATACTACTAACTGACCGAAACCTAAA

CACATCCTTTTTCGACCCAGCTGGAGGAGGTGACCCAATTCTATATCAACATCTATTT

>E16_810_Henlea_perpusilla_3ind

------------------------------------GATAGGAGCAGCCATAAGCCTTCT

AATTCGAATTGAACTAAGTCAACCAGGTTCATTCCTCGGAAGAGATCAACTCTACAACAC

TATTGTTACTGCACATGCATTTCTTATAATTTTTTTCCTAGTTATACCTGTATTTATTGG

GGGGTTCGGAAACTGACTTCTCCCATTAATACTGGGAGCCCCAGATATAGCTTTTCCCCG

ACTAAATAACATAAGATTTTGACTTCTTCCCCCATCACTTCTTCTTCTACTTTCCTCTGC

AGCTGTGGAAAAAGGAGCCGGAACCGGCTGAACTGTATACCCCCCCCTATCAAGAAATAT

CGCTCATGCGGGCCCATCTGTAGACCTAGCCATTTTCTCCCTCCACTTAGCCGGAGCATC

CTCCATCTTAGGTGCAGTAAACTTTATCACTACTGTTATTAACATACGTTGACAAGGCCT

TCACCTAGAACGAATTCCCCTATTTGTATGAGCTGTAACAATTACAGTAGTCCTTCTCCT

CTTATCTTTACCAGTGTTAGCCGGAGCAATTACTATACTACTAACTGACCGAAACCTAAA

CACATCCTTTTTCGACCCAGCTGGAGGAGGTGACCCAATTCTATATCAACACCTATTT

>E2_LN810248_Fridericia_sp

-ACACTATCCTTCATCCTTGGTGTATGAGCTGGCATAATGGGAGCAGCAATAAGCCTCCT

AATTCGAATTGAACTAAGTCAACCAGGATCATTCTTAGGAAGAGATCAACTATATAACAC

TATCGTAACAGCTCACGCATTCCTAATAATTTTCTTCCTAGTGATACCAGTATTTATTGG

TGGCTTCGGCAACTGACTTCTTCCATTAATACTTGGTGCACCAGATATAGCATTTCCTCG

ACTTAACAATATAAGATTTTGACTTCTACCACCTTCCCTAATACTTCTTCTTTCATCAGC

AGCTGTAGAAAAGGGTGCCGGCACAGGTTGAACAGTTTACCCACCACTAGCTAGAAACAT

AGCACACGCTGGCCCATCCGTAGACTTAGCTATTTTCTCACTACACTTAGCAGGAGCATC

ATCTATTTTAGGAGCAGTAAACTTTATCTCAACTGTAATTAACATACGATGACAAGGCCT

TCAATTAGAACGAATTCCTCTATTTGTGTGAGCTGTCACCATTACTGTAGTACTACTACT

TCTCTCTCTTCCAGTTTTAGCAGGAGCCATTACTATATTATTAACAGATCGAAACTTAAA

CACCTCATTCTTCGATCCTGCCGGTGGAGGAGATCCTATTCTATATCAACACTTATTC

>E15_809_Fridericia_sp

--CACTATATTTCATTCTAGGAGTATGAGCAGGCATAATAGGAGCAGCCATAAGACTATT

AATTCGAATTGAATTAAGACAACCAGGCTCCTTTCTAGGCAGAGACCAACTATATAACAC

AATCGTAACTGCCCATGCCTTTCTCATAATTTTTTTTCTAGTAATACCAGTATTTATTGG

GGGATTCGGAAATTGATTACTTCCATTAATACTAGGGGCACCAGACATAGCCTTCCCGCG

ACTCAATAACATAAGATTCTGACTCTTGCCCCCCTCCCTTCTCCTTCTTTTATCTTCTGC

AGCAGTAGAAAAAGGTGCAGGTACAGGTTGAACAGTCTATCCTCCGCTAGCTAGAAATAT

GGCTCACGCCGGACCATCAGTAGATCTAGCTATTTTTTCTCTACATTTAGCCGGAGCCTC

ATCCATTCTAGGGGCAGTAAATTTTATTTCAACCGTCATTAACATACGATGACAAGGTCT

ACAATTAGAACGAATTCCATTATTTGTATGAGCTGTAACCATTACAGTAGTTCTTCTATT

ATTATCTCTACCAGTTCTAGCCGGAGCAATTACTATACTTTTAACAGATCGAAACCTTAA

TACATCATTTTTTGATCCAGCCGGAGGTGGAGATCCAATTTTATACCAACACCTATTT

>E7_819_Achaeta_sp_3ind

--CTCTATNCTTTATCCTCGGAATATGAGCTGGCATAATAGGTGCAGCTATAAGACTCCT

CATCCGATTTGAACTAAGTCAACCCGGATCATTTTTAGGGAGAGACCAATTATATAATAC

CATTGTAACAGCACATGCATTCTTAATAATTTTCTTTCTTGTAATACCAGTATTTATTGG

AGGATTTGGAAATTGACTCATTCCATTAATACTTGGAGCTCCAGATATAGCTTTTCCACG

ATTAAATAATATAAGATTCTGATTACTTCCACCATCCCTCATATTACTTCTTTCTTCTAC

AGCTGTAGAAAAAGGTATAGGAACAGGATGAACAGTATATCCTCCTTTAGCTAGAAACAT

TGCTCATGCAGGACCATCTGTAGATTTAGCCATTTTCTCTCTTCACTTAGCAGGAGCTTC

ATCAATTCTAGGAGCAGTAAATTTCATTACTACTGTAATTAATATACGATGACAAGGTAT

ACGACTAGAACGAATTCCACTATTTGTATGAGCTATAATAATCACAGTAGCCCTCCTACT

TTTAGCACTACCAGTCCTAGCTGGCGCAATTACTATGCTTCTTACTGATCGAAACCTTAA

CACATCATTCTTTGATCCAGCTGGTGGAGGTGACCCTATTCTATATCAACATTTATTT

>E1_LN810247_Enchytraeus_buchholzi

------------------------------------------------------------

-----------------------------CATTTCTAGGAAGAGATCAGTTATATAATAC

AATTGTAACTGCCCATGCATTCCTAATAATTTTCTTTCTAGTAATACCTGTATTTATCGG

GGGATTTGGAAATTGACTATTACCACTAATACTAGGTGCCCCAGATATAGCTTTTCCGCG

ATTAAATAACATAAGATTCTGCATTCTACCTCCAGCACTAATACTACTTCTATCCTCAGC

AGCAGTAGAAAAGGGGGCTGGAACAGGGGGAACAGTTTATCCTCCTTTAGCTAGAAATAT

TGCTCACGCTGGACCATCTGTAGACTTAGCAATTTTCTCTCTACATTTAGCTGGAGCATC

ATCAATTCTAGGAGCTGTTAATTTCATCACTACAGTAATTAACATACGATGACAAGGACT

AACCCTAGAACGAATTCCTTTATTTGTATGAGCCGTAACTATCACAGTAGTTCTTCTTCT

TTTATCTCTTCCAGTATTAGCAGGGGCTATCACTATATTATTAACCGATCGTAATTTAAA

TACCTCATTCTTCGACCCTGCAGGCGGAGGAGATCCTATTCTATATCAACACTTATTC

>E9_694_Globulidrilus_riparius

CACTATATATTTTATCCTAGGTGTTTGAGCCGGAATACTAGGAGCAGCAATAAGACTATT

AATTCGAATTGAATTAAGTCAACCTGGTGCATTTCTTGGAAGAGACCAACTCTATAATAC

TATCGTAACAGCTCATGCATTTCTAATAATTTTCTTCTTAGTTATGCCTGTGTTCATTGG

TGGATTTGGAAACTGATTACTCCCTTTAATGCTAGGAGCACCAGATATAGCCTTTCCACG

TCTAAATAATATAAGATTTTGACTTCTTCCACCTTCACTACTTCTTCTTGTTTCATCTGC

AGCAGTAGAAAAAGGAGCTGGAACTGGTTGAACTGTATATCCCCCTCTATCAAGAAACAT

AGCTCACGCAGGACCATCCGTAGACCTAGCAATTTTTTCCCTTCATTTAGCAGGTGCATC

ATCAATTCTTGGAGCAGTAAATTTTATCACAACAGTAATTAACATACGATGACAAGGATT

GAGATTAGAGCGAATTCCTTTATTTGTATGAGCTGTAACAATCACTGTAGTTCTTCTTCT

TCTATCTCTCCCTGTTCTGGCTGGTGCAATCACCATATTATTAACTGATCGAAATCTAAA

CACTTCATTTTTTGACCCTGCTGGAGGAGGAGATNCAGTACTATATCAACACTTATTT

>E9_696_Globulidrilus_riparius_2ind

CACTATATATTTTATCCTGGGTGTTTGAGCCGGAATACTAGGAGCAGCAATAAGACTATT

AATTCGAATTGAATTAAGTCAACCTGGCGCATTCCTTGGAAGAGACCAACTCTATAATAC

TATCGTAACAGCACATGCATTTCTAATAATTTTCTTCTTAGTTATGCCTGTGTTCATTGG

TGGATTTGGAAACTGATTACTCCCTTTAATGCTAGGAGCACCAGATATAGCCTTTCCACG

TCTAAATAATATAAGATTTTGACTTCTTCCACCTTCACTACTTCTTCTTGTATCATCTGC

AGCAGTAGAAAAAGGAGCTGGAACTGGTTGAACCGTATACCCCCCTCTATCGAGAAACAT

AGCTCATGCAGGACCATCTGTAGACCTAGCAATTTTTTCTCTTCATTTAGCAGGTGCATC

ATCAATTCTTGGGGCAGTAAATTTTATCACAACAGTAATTAACATACGATGACAAGGATT

AAGATTAGAGCGAATTCCTTTATTTGTATGAGCTGTAACAATCACTGTAGTTCTTCTTCT

TCTATCTCTCCCTGTTCTAGCTGGCGCAATCACCATATTATTAACTGATCGAAATCTAAA

CACTTCATTTTTTGACCCTGCTGGAGGAGGAGATCCAGTATTATATCAACACTTATTT

>E9_706_Globulidrilus_riparius

CACTATATATTTTATCCTAGGTGTTTGAGCCGGAATACTAGGAGCAGCAATAAGACTATT

AATTCGAATTGAATTAAGTCAACCTGGTGCATTCCTTGGAAGAGATCAACTCTATAATAC

TATCGTAACAGCTCATGCATTTCTAATAATTTTCTTCTTAGTTATGCCTGTATTCATTGG

TGGATTTGGAAACTGATTACTGCCTTTAATACTAGGGGCACCAGATATAGCCTTTCCACG

TCTAAATAACATAAGATTTTGACTTCTTCCACCTTCACTACTTCTTCTTGTTTCATCTGC

AGCAGTAGAAAAAGGAGCTGGAACTGGTTGAACCGTATATCCTCCTCTATCAAGAAACAT

AGCTCATGCAGGACCATCTGTAGATCTAGCAATTTTTTCTCTTCATTTAGCAGGTGCATC

ATCAATTCTTGGAGCAGTAAATTTTATCACAACAGTAATTAACATACGATGACAAGGATT

AAGATTAGAACGAATTCCTTTATTTGTATGAGCTGTAACAATCACTGTAGTTCTTCTTCT

TCTATCTCTCCCTGTTCTGGCTGGTGCAATCACCATATTATTAACTGATCGAAATCTAAA

CACTTCATTTTTTGATCCTGCTGGTGGAGGAGA-------------------------

>E10_703_Globulidrilus_riparius

CACAATATATTTTATTTTAGGTGTTTGAGCAGGTATACTAGGTGCAGCTATAAGATTACT

AATTCGTATTGAATTAAGCCAACCAGGATCATTTCTTGGTAGAGATCAATTATACAATAC

CATTGTAACCGCTCATGCCTTCTTAATAATCTTCTTCTTAGTAATACCTGTATTTATTGG

AGGATTTGGAAACTGACTACTTCCATTAATACTAGGTGCCCCTGACATAGCATTCCCGCG

ACTAAATAATATAAGATTCTGACTCCTTCCTCCATCACTATTACTACTAGTATCTTCAGC

CGCTGTTGAAAAAGGTGCAGGAACAGGATGAACTGTATACCCACCCCTTTCAAGAAATAT

AGCTCATGCAGGCCCATCTGTAGATCTAGCTATTTTCTCTCTTCATTTAGCTGGTGCGTC

TTCAATTCTAGGTGCTGTAAATTTCATCACTACAGTAATTAATATACGATGACAAGGTCT

TAGATTAGAACGAATCCCTTTATTTGTTTGAGCAGTAACTATTACTGTAGTTCTTCTTCT

ACTATCTCTACCAGTTTTAGCAGGAGCCATTACTATACTTCTAACTGACCGTAATTTAAA

CACCTCATTCTTTGATCCTGCTGGAGGTGGAG--------------------------

>E11_697_Globulidrilus_riparius

CACTATATATTTTATTCTAGGTATTTGAGCAGGTATACTTGGAGCTGCTATAAGGCTTTT

AATTCGAATCGAATTAAGACAACCTGGATCCTTTCTTGGTAGAGATCAGCTTTATAATAC

TATTGTAACAGCTCATGCCTTTCTAATAATCTTCTTCTTAGTTATACCTGTATTTATTGG

GGGTTTCGGAAATTGATTATTACCATTAATACTAGGGGCTCCAGATATAGCATTTCCACG

TTTAAATAATATAAGATTTTGACTTCTACCTCCATCATTATTATTACTTGTATCATCCGC

TGCAGTTGAAAAAGGTGCAGGTACAGGATGAACAGTATATCCACCTCTTTCTAGAAATAT

GGCACATGCAGGACCTTCTGTAGATTTAGCTATTTTTTCTCTTCATCTAGCAGGTGCTTC

ATCAATTTTAGGAGCAGTAAACTTTATTACAACAGTAATTAATATACGATGACAAGGTCT

TAGATTAGAACGAATCCCTCTATTTGTATGGGCAGTAACCATTACTGTAGTACTTCTTCT

ATTATCCCTTCCAGTTTTAGCCGGCGCTATTACTATACTTTTAACTGATCGAAATTTAAA

TACATCATTTTTCGATCCTGCTGGAGGCGGGGACCCTGTTTTATATCAACACTTATTT

>E11_701_Globulidrilus_riparius_2ind

CACTATATATTTTATTCTAGGCATTTGAGCAGGTATACTTGGTGCTGCTATAAGACTTTT

AATTCGAATTGAATTAAGTCAACCAGGATCTTTTCTTGGTAGAGACCAACTATATAATAC

TATTGTAACAGCCCATGCCTTCCTAATAATCTTTTTCCTAGTTATACCTGTATTTATTGG

GGGTTTTGGAAATTGACTACTCCCATTAATACTTGGGGCCCCAGATATAGCATTTCCACG

ACTAAATAATATAAGATTTTGACTTCTACCTCCTTCATTATTACTTCTTGTATCATCTGC

TGCTGTTGAAAAAGGCGCAGGAACAGGATGAACAGTATATCCTCCCCTTTCTAGAAATAT

AGCACATGCTGGACCCTCTGTAGACTTAGCCATTTTCTCCCTTCACTTAGCAGGTGCTTC

TTCAATTCTAGGAGCAGTAAATTTCATTACGACAGTAATTAATATACGATGACAAGGTCT

TAGATTAGAACGAATCCCTTTATTTGTATGAGCAGTAACAATCACTGTAGTACTTCTTCT

TCTATCTCTTCCAGTATTAGCTGGTGCCATTACTATACTTTTAACTGATCGAAATTTAAA

TACATCATTTTTTGATCCAGCTGGAGGAGGTGATCCCGTTTTATACCAACACTTATT-

>E11_702_Globulidrilus_riparius_16ind

CACTATATATTTTATTTTAGGTATTTGAGCAGGTATACTTGGAGCAGCCATAAGACTCTT

AATTCGAATTGAATTAAGACAACCTGGATCTTTCCTTGGTAGAGATCAGTTATATAATAC

AATCGTAACAGCTCACGCCTTTCTAATAATTTTCTTCTTAGTTATACCTGTATTTATTGG

TGGTTTCGGAAATTGATTATTACCTTTAATACTTGGAGCCCCAGATATAGCATTTCCACG

ACTAAATAATATAAGATTTTGACTTCTACCCCCATCATTACTACTTCTTGTATCATCTGC

TGCAGTTGAAAAAGGTGCAGGAACAGGGTGAACAGTATACCCCCCTCTTTCCAGTAATAT

AGCTCATGCAGGGCCTTCTGTTGATTTAGCTATTTTTTCTCTACATTTAGCAGGTGCTTC

TTCAATCTTAGGGGCAGTAAATTTTATTACTACCGTAATTAATATACGATGACAAGGTCT

CAGACTAGAACGAATTCCACTATTTGTATGAGCAGTAACTATTACTGTAGTACTTCTCCT

ATTATCTCTCCCAGTTTTAGCAGGTGCCATTACTATACTTTTAACTGACCGAAATTTAAA

CACATCATTTTTCGATCCTGCTGGAGGGGGTGATCCTGTATTATATCAACATTTATTT

>E3_LN810245_Lumbricillus_rutilus_2ind

-ACACTATATTTTATTTTAGGAGTATGAGCTGGAATACTAGGAGCAGCCATAAGACTTTT

AATTCGAATTGAATTAAGACAACCTGGCGCTTTTTTAGGAAGAGATCAGCTTTATAATAC

TATCGTAACAGCTCATGCATTCTTAATAATTTTTTTCTTAGTTATACCAGTATTTATTGG

TGGATTTGGAAATTGATTAATTCCGCTAATATTGGGAGCTCCTGACATAGCATTCCCTCG

TCTTAACAATATAAGATTTTGACTTCTACCTCCAGCTCTTTTACTTCTAGTTTCTTCAGC

AGCAGTAGAAAAAGGTGCTGGGACTGGCTGAACAGTTTACCCACCTCTAGCAAGAAATCT

AGCTCATGCAGGTCCATCCGTAGATTTAGCAATTTTCTCTCTTCATTTAGCCGGTGCCTC

ATCTATTCTTGGAGCAGTAAACTTTATTACTACAGTAGTAAATATACGTTGACAAGGTCT

TCGACTTGAACGAATTCCTCTTTTTGTATGAGCAGTAGTAATTACAACAGTTCTTCTTCT

TCTATCTCTTCCAGTTCTTGCAGGGGCAATTACAATACTACTAACTGATCGAAATCTAAA

CACTTCATTTTTTGACCCAGCCGGAGGTGGAGATCCTGTTCTTTATCAACATTTATTT

>E13_807_Lumbricillus_sp

--CACTTTATTTTATCCTCGGAGTATGAGCAGGTATAATAGGCGCAGCTATAAGCCTAAT

TATTCGAACAGAACTTAGCCAACCAGGATCATTCTTAGGTAGGGACCAGTTATACAATAC

TGTTGTAACAGCCCACGCATTTTTAATAATTTTCTTTTTAGTTATACCAGTATTCATTGG

GGGCTTTGGTAATTGGCTTTTACCCCTAATACTCGGAGCACCTGATATAGCATTTCCGCG

ACTCAACAATATAAGATTTTGACTTCTTCCCCCTTCACTACTTCTACTTCTCTCATCAGC

CGCAGTAGAAAAAGGTGCTGGAACTGGTTGAACAGTTTACCCACCCCTAGCAAGAAATAT

GGCACATGCAGGACCATCTGTAGATTTAGCAATTTTTTCCCTTCATTTAGCAGGGGCTTC

CTCTATTCTTGGTGCCGTAAATTTTATTTCCACTGTAATCAATATACGATGACAAGGCCT

CCAGTTAGAGCGAATTCCACTATTTGTATGAGCAGTTACAATCACAGTAGTACTTCTTCT

CTTATCTTTACCAGTATTAGCTGGTGCTATTACCATATTACTAACAGATCGAAACCTAAA

TACTTCATTCTTTGACCCTGCAGGTG--------------------------------

>H1_885_Haplotaxis_gordioides_2ind

AACCCTTTACTTTATCCTAGGCATCTGAGGGGGACTTCTAGGAACAAGAATAAGAATAGT

AATCCGAATTGAACTAAGACAACCAGGGTCATTCCTTGGTAGAGATCAATTATACAATAC

CATTGTTACTGCCCATGCCTTCCTAATAATTTTCTTTCTTGTCATACCAGTATTTATTGG

AGGGTTCGGAAACTGACTCTTACCTCTAATGTTAGGAGCCCCCGACATAGCATTCCCACG

ACTCAACAATATAAGATTTTGACTTCTACCTCCTGCAACAATCCTTCTTGTGTCCTCAGC

AGCAGTAGAAAAAGGAGCAGGAACTGGCTGAACTGTTTACCCGCCCTTAGCCAGAAATCT

TGCACATGCTGGCCCTTCTGTTGATCTCGCCATTTTCTCTCTTCATTTAGCAGGAGTTTC

CTCCATTTTAGGAGCAGTAAATTTCATCACCACTGTTGTCAATATACGATGAAATGGCCT

GCGGTTAGAACGAATTCCTTTATTCGTATGATCCGTAACTATTACAGTGGTTCTTCTCCT

TTTATCTCTACCAGTGCTAGCCGGCGCTATTACCATACTACTAACTGACCGAAATCTTAA

TACATCATTCTTCGACCCAGCTGGAGGAGGAGATCCAGTTCTGTATCAACACCTATTC

>E4_LN810244_Marionina_argentea

TCATTATACTTTTATTCTAGGAACTTGAGCAGGGATACTAGGAGCAGCTATAAGCCTCTT

AATTCGATTTGAACTAAGACAACCTGGATCGTTTCTAGGTAGAGATCAACTCTACAACAC

TATCGTTACAGCTCATGCATTCTTAATAATCTTTTTCTTAGTTATACCAGTATTTATTGG

TGGATTTGGAAACTGACTCTTACCCCTTATATTAGGAGCCCCTGACATAGCCTTCCCCCG

CCTTAATAATTTAAGATTTTGATTACTTCCTCCATCTCTCCTACTTTTAGTCTCATCTGC

CGCAGTTGAAAAAGGCGCAGGCACTGGATGAACAGTATACCCGCCCTTGGCCTCCAATAT

TGCCCATTCAGGACCTTCTGTTGATTTAGCCATCTTCTCCCTTCATCTAGCAGGTGCGTC

CTCCATCTTAGGTGCAATTAATTTCATCACAACTGTTATCAATATACGCTGACAAGGCCT

CCAACTAGAACGAATTCCTCTATTTGTCTGAGCCGTTACAATTACAGTAGTTCTTCTTCT

TTTATCACTTCCAGTACTAGCAGGGGCTATTACCATACTACTAACAGATCGAAACCTAAA

CACTTCCTTCTTTGACCCTGCAGGAGGGGGGGACCCTATTTTATATCAACACTTATTT

>P1_1037_Pristina_jenkinae

AACTCTATATTTATCTTCGGGTGTATGAGCAGGAATAGTTGGAACCGGAACAAGACTACT

CATTCGAGTTGAATTAGCTCAACCAGGCTCATTTCTCGGAAGGGACCAACTTTACAATAC

ACTTGTTACTGCACATGCATTCCTAATAATTTTCTTTCTAGTAATGCCAGTATTTATTGG

AGGATTCGGTAATTGACTTCTTCCATTAATACTAGGAGCACCAGACATGGCATTTCCACG

ACTAAACAACATAAGATTTTGACTACTTCCCCCTGCACTAATTATACTAGTAGCTTCAGC

AGCAGTTGAAAAGGGGGCAGGAACAGGGTGAACAGTATATCCCCCACTTGCAAGAAATAT

TGCTCATGCAGGACCATCTGTAGACATAGCAATTTTTTCTCTTCATCTAGCAGGTGCATC

ATCAATCCTAGGGGCAGTAAACTTTATCTCAACTGTCCTAAATATACGAACTAAAGGAAT

ACGACTAGAACGAATTCCTCTATTTGTATGAGCTGTATTCTTAACAGTAATCCTACTACT

TCTGTCACTTCCAGTACTAGCAGGAGCAATTACCATACTTCTTACTGATCGTAACCTAAA

TACTTCATTCTTTGACCCAGCTGGGGGTGGTGACCCAATCCTATATCAACATCTATAT

>LC1_LN810243_Dendrodrilus_rubidus

AACACTATATTTCATTTTAGGCGTCTGAGCTGGCATAGTCGGCGCTGGAATAAGACTTCT

AATTCGAATTGAACTAAGACAGCCGGGAGCCTTTCTAGGAAGAGATCAACTTTATAATAC

AATTGTAACAGCCCATGCATTTGTAATAATCTTCTTTCTAGTTATGCCAGTATTTATTGG

GGGGTTTGGAAATTGACTTCTCCCTTTAATACTAGGTGCTCCTGATATAGCATTCCCCCG

TCTAAATAACATGAGATTCTGACTACTACCCCCTGCACTTATTCTCTTAGTATCTTCTGC

TGCAGTAGAAAAGGGGGCTGGAACTGGATGAACTGTTTACCCCCCCTTATCTAGAAACAT

TGCGCATGCTGGGCCCTCTGTGGATCTAGCAATTTTCTCTCTTCATTTAGCTGGGGCATC

CTCAATTCTCGGTGCCATTAACTTTATTACAACAGTTATTAATATACGCTGAAGGGGTCT

ACGTCTAGAGCGTATTCCATTATTTGTATGAGCAGTCTTAATTACTGTAGTATTACTTCT

TCTCTCTTTACCAGTTCTTGCTGGCGCAATTACTATACTTCTCACAGACCGAAACCTAAA

CACCTCATTCTT----------------------------------------------

>LC4_1048_Helodrilus_oculatus

AACCTTATATTTTATCCTTGGTGTTTGAGCCGGCATAGTAGGAGCTGGCATAAGCCTTCT

CATTCGAATTGAACTAAGACAACCGGGAGCCTTCCTGGGAAGAGATCAACTTTACAATAC

AATTGTTACAGCTCATGCATTCGTAATAATCTTCTTCCTTGTTATGCCCGTATTTATTGG

AGGATTTGGAAATTGGCTTCTTCCATTAATACTGGGTGCCCCTGATATGGCGTTCCCCCG

ACTAAATAACATAAGATTTTGACTACTTCCCCCCTCATTAATCCTCTTAGTTTCCTCTGC

AGCAGTAGAAAAAGGAGCAGGAACCGGCTGAACAATGTATCCACCTTTAGCCAGAAATCT

TGCCCACGCAGGTCCCTCAGTAGATTTAGCCATTTTTTCCCTACACTTAGCAGGGGCATC

TTCAATTCTCGGGGCCATCAATTTTATTACAACAGTTATCAACATACGATGAAGAGGTCT

ACGTCTAGAACGTATTCCTTTATTCGTCTGAGCCGTTGTAATTACGGTAGTACTCCTACT

CCTATCTCTTCCAGTGCTGGCAGGGGCAATTACTATGCTCCTCACAGACCGAAACCTTAA

TACCTCTTTCTTTGACCCTGCTGGAGGGGGGGATCCTATCTTATACCAACATTTATTT

>LC4_LN810242_Helodrilus_oculatus

AACCTTATATTTTATCCTTGGCGTTTGAGCCGGCATAGTAGGAGCTGGCATAAGCCTTCT

CATTCGAATTGAACTAAGACAACCGGGAGCCTTCCTGGGAAGAGATCAACTTTACAATAC

AATTGTTACAGCTCATGCATTCGTAATAATCTTCTTCCTTGTTATGCCCGTATTTATTGG

AGGATTTGGAAATTGACTTCTTCCATTAATACTGGGTGCCCCTGATATGGCGTTCCCCCG

ACTAAATAACATAAGATTTTGACTACTTCCCCCCTCATTAATCCTCTTAGTTTCCTCTGC

AGCAGTAGAGAAAGGAGCAGGAACCGGCTGAACAGTGTATCCACCTTTAGCCAGAAATCT

TGCCCACGCAGGTCCCTCAGTAGATTTAGCCATTTTTTCTCTACACTTAGCAGGGGCATC

TTCAATTCTCGGGGCCATCAATTTTATTACAACAGTTATCAACATACGATGAAGAGGTCT

ACGTCTAGAACGTATTCCTTTATTCATCTGAGCCGTTGTAATTACGGTAGTACTCCTACT

CCTATCTCTTCCAGTGCTGGCAGGGGCAATTACTATGCTCCTCACAGACCGAAACCTTAA

TACCTCTTTCTTTACCCTGCTGGAGGGGG-----------------------------

>LC2_LN810251_Eiseniella_tetraedra_2ind

-ACCCTTTATTTCACTTTAGGTATCTGAGCTGGCATGGTGGGAGCTGGAATAAGCTTACT

AATTCGAATCGAATTAAGACAACCTGGAGCCTTCTTAGGTAGCGACCAATTATACAATAC

TATTGTTACAGCACATGCATTTGTAATAATCTTCTTCCTGGTAATGCCCGTATTCATTGG

TGGATTCGGAAACTGACTGCTACCTCTAATACTAGGTGCACCAGATATAGCATTTCCACG

TTTAAATAACATGAGATTTTGACTTCTGCCTCCTTCCTTAATTCTACTAGTATCCTCTGC

AGCCGTAGAAAAGGGGGCCGGGACAGGATGAACTGTTTATCCTCCCTTAGCAAGAAATTT

AGCTCATGCAGGTCCATCAGTAGACTTAGCTATTTTCTCATTACATCTAGCAGGAGCTTC

ATCTATTTTAGGGGCCATCAACTTTATTACTACAGTCATCAATATGCGATGAAGAGGTTT

ACGATTAGAGCGAATCCCCCTTTTCGTATGAGCTGTATTAATTACAGTGATTCTTCTTCT

ACTATCATTGCCCGTGCTAGCAGGAGCAATCACCATATTACTAACCGACCGAAATCTAAA

TACATCATTCTTCGATCCGGCTGGTGGCGGTGATCCAATTCTATATCAACACCTCTTC

>LC3_961_Eiseniella_tetraedra_2ind

AACCCTTTACTTCATTTTAGGTATTTGAGCTGGGATGGTAGGAGCTGGAATAAGCTTACT

AATTCGAATCGAATTAAGCCAACCTGGAGCCTTTTTAGGTAGTGACCAACTATACAACAC

TATTGTCACAGCACACGCATTCGTAATAATCTTTTTCCTAGTAATACCAGTATTCATTGG

TGGATTCGGAAACTGGTTATTACCTTTAATACTTGGTGCGCCAGATATAGCATTTCCACG

TTTAAATAACATAAGATTTTGACTTTTACCTCCTTCTCTAATTCTATTAGTATCTTCCGC

CGCCGTAGAAAAGGGTGCCGGGACAGGTTGAACTGTTTACCCACCCTTAGCAAGAAACTT

AGCTCATGCAGGACCATCCGTAGATCTAGCTATTTTCTCCTTACATTTAGCGGGAGCTTC

ATCTATTTTAGGGGCCATCAACTTTATTACCACAGTCATTAATATACGATGAAGGGGCTT

ACGATTAGAACGAATCCCCCTTTTCGTCTGAGCTGTGCTAATTACAGTGATTCTTCTACT

ACTATCATTGCCCGTGCTTGCAGGAGCAATTACCATGTTATTAACTGACCGAAATCTCAA

CACATCTTTTTTTGATCCGGCTGGTGGTGGTGACCCAATTCTATATCAACACCTCTTC

>LC3_LN810249_Eiseniella_tetraedra

------------CATTTTAGGTATTTGAGCTGGGATAGTGGGAGCTGGTATAAGCTTACT

AATTCGAATCGAATTAAGCCAACCAGGAGCCTTCCTAGGTAGTGACCAATTATATAACAC

TATTGTTACAGCACATGCATTCGTAATAATCTTCTTCCTAGTAATACCCGTATTCATTGG

TGGATTCGGAAACTGATTGCTACCTCTAATACTAGGTGCACCAGATATAGCATTTCCACG

TTTAAACAACATAAGATTTTGACTTTTACCTCCTTCTTTAATTCTACTAGTATCTTCCGC

AGCCGTAGAAAAGGGGGCCGGGACAGGGTGAACTGTTTATCCGCCCTTAGCAAGAAACTT

GGCCCATGCAGGACCATCAGTAGATCTAGCTATTTTCTCCTTACACTTAGCAGGAGCTTC

ATCTATTTTAGGTGCCATCAACTTTATTACTACAGTCATTAATATGCGATGAAGGGGCTT

ACGATTAGAACGAATTCCCCTTTTCGTGTGAGCTGTACTGATTACAGTAATTCTTCTATT

ATTATCGTTGCCCGTGCTAGCAGGAGCAATTACCATACTATTAACCGACCGAAATCTCAA

TACATCATTCTTTGACCCGGCTGGTGGTGGTGACCCAATTCTGTACCAACACCTCTTC

>E8_693_Cernosvitoviella_minor

AACACTTTACTTTATTTTAGGTGTATGAGCAGGAATACTAGGAGCTGCTATAAGACTTCT

AATTCGAATTGAACTGAGACAACCTGGCTCATTCCTAGGGAGAGATCAATTATATAATAC

TATCGTTACAGCACATGCATTTTTAATAATTTTCTTCTTAGTAATACCAGTATTTATTGG

AGGATTTGGGAACTGACTTTTACCTTTAATATTAGGAGCTCCAGACATGGCTTTTCCTCG

ATTAAACAACATAAGATTTTGACTCCTACCACCATCATTAATATTATTAGTATCCTCAGC

AGCCGTAGAAAAAGGAGCCGGCACTGGATGAACAGTATATCCACCTCTTTCTAGAAATTT

AGCACATGCTGGACCATCAGTAGATTTAGCAATTTTCTCTCTTCATCTTGCAGGAGCTTC

CTCAATTCTTGGGGCCGTAAACTTTATTACTACAGTAGTAAATATACGTTGACAAGGCCT

TCGACTAGAACGAATCCCCCTATTTGTATGAGCAGTAGTAATTACAGTAGTTCTACTACT

CTTATCTCTACCAGTACTAGCAGGAGCAATTACTATACTATTAACAGACCGAAATTTAAA

CACATCATTCTTTGACCCGGCAGGAGGGGGAGACCCTATTTTATACCAACACTTATTC

>E8_1043_Cernosvitoviella_minor

AACACTTTACTTTATTTTAGGGGTATGAGCAGGAATACTAGGAGCTGCTATAAGACTTCT

AATTCGAATTGAACTAAGACAACCTGGCTCATTCCTAGGGAGAGATCAATTATATAATAC

TATCGTTACAGCACATGCATTCTTAATAATTTTTTTCTTAGTAATACCAGTATTTATTGG

AGGGTTTGGGAACTGACTTTTACCATTAATATTAGGGGCCCCAGACATGGCCTTTCCTCG

ATTAAACAACATAAGATTTTGACTCCTACCTCCATCATTAATACTATTAGTATCTTCAGC

AGCTGTAGAAAAAGGGGCCGGCACTGGATGAACAGTGTACCCCCCTCTTTCTAGAAACTT

AGCACATGCTGGGCCATCAGTAGATTTAGCAATTTTTTCTCTTCATCTTGCAGGAGCTTC

TTCAATTCTTGGAGCTGTAAACTTTATTACTACAGTAGTGAATATACGTTGACAAGGCCT

TCGGCTAGAACGAATTCCCCTATTTGTATGAGCAGTAGTTATTACAGTAGTTCTACTACT

TTTATCTCTACCAGTACTAGCAGGAGCAATTACTATACTATTAACAGACCGAAATTTAAA

TACATCATTCTTTGATCCGGCAGGAGGAGGAGATCCTATTTTATACCAACACTTATTC

>N7_820_Vejdovskyella_intermedia

--CATTATATTTAATTTTAGGGGTATGAGCAGGAATAGTTGGAACCGGAACTAGAATATT

AATTCGAGTTGAATTATCACAACCAGGAGCTTTTCTTGGGAGAGACCAATTATATAATAC

ATTAGTAACTGCACACGCATTCTTAATAATCTTTTTCTTAGTAATACCCGTATTTATTGG

TGGATTCGGAAATTGACTATTACCATTAATACTAGGAGCACCGGATATAGCATTCCCACG

ACTTAATAATCTAAGATTTTGACTATTACCACCATCATTAATTCTATTAATTTCTTCAGC

TGCAGTAGAAAAAGGAGCAGGAACAGGCTGAACCGTATATCCACCACTTTCAAGAAATCT

AGCACATGCGGGACCATCAGTAGATATAGCAATTTTTTCACTACATTTAGCTGGTGCATC

ATCTATTCTAGGAGCCGTAAATTTTATTACAACTGTAATAAATATACGATGAAATGGAAT

ACGATTAGAACGAGTACCATTATTCGTTTGATCGGTAATATTAACAGTTATTCTTCTTAT

TCTATCATTACCTGTACTTGCGGGAGCANATACAATATTATTAACAGATCGAAACTTAAA

TACCTCATTCTTCGATCCTGCTGGGGGTGGAGACCCAATTCTTTATCAAACATCATTT

>N1_LN810268_Chaetogaster_diaphanus

CACTCTATATTTAATTTTAGGGGTATGAGCAGGAATAATTGGTACAGGAACTAGAATACT

AATTCGAATTGAACTATCTCAGCCTGGGTCATTTCTAGGAAGAGATCAACTATATAATAC

TTTAGTTACTGCACATGCATTCTTGATAATTTTCTTTTTAGTAATACCTGTATTTATTGG

GGGATTTGGAAATTGACTTCTACCATTAATACTAGGTGCACCAGATATAGCTTTTCCACG

TTTAAATAATTTAAGATTTTGATTATTACCTCCTTCATTAATTTTATTAATTTCATCTGC

TGCTGTAGAAAAAGGAGCTGGAACAGGATGAACAGTATACCCTCCACTATCAAGAAATCT

TGCCCATGCGGGACCATCAGTAGATATAGCAATTTTCTCTCTTCACTTAGCAGGGGCTTC

ATCTATTTTAGGAGCAGTTAACTTTATTGCAACAACAATTAATATGCGATGAAACGGAAT

ACGGCTAGAACGACTACCTTTATTTGTATGGGCAGTTCTACTAACCGTTATTCTTCTTCT

ATTATCACTTCCAGTGCTTGCTGGGGCTATTACAATGTTACTTACAGATCGAAACCTTAA

TACTTCTTTCTTTGATCCGGCTGGTGGTGGAGATCCTATTTTATATCAACATCTATTC

>N9_992_Nais_communis

TACATTATATTTAATTCTAGGAGTATGAGCAGGAATAATCGGAACAGGGACAAGAATACT

AATTCGAATCGAATTAGCTCAACCCGGAGCCTTCTTAGGAAGAGATCAATTATACAACAC

CTTAGTTACTGCACACGCGTTTTTAATAATTTTCTTTCTTGTTATACCTGTATTTATTGG

TGGATTCGGAAATTGACTTCTACCTCTAATACTAGGTGCACCAGATATAGCATTCCCACG

ACTTAATAATCTTAGATTCTGATTACTTCCGCCATCATTAATTCTTTTAGTTTCATCAGC

GGCCGTTGAAAAAGGAGCCGGAACTGGTTGAACTGTTTACCCACCATTATCAAGAAATCT

AGCCCATGCAGGACCATCAGTAGACATGGCTATTTTCTCATTACATTTAGCCGGGGCATC

ATCTATTTTAGGAGCAGTTAATTTTATTACAACAGTAATAAATATGCGATGAAATGGTAT

ACGATTAGAACGACTACCACTATTTGTATGAGCTGTAACACTTACTGTAATCCTTCTACT

ATTATCATTACCAGTTCTAGCAGGAGCAATTACTATGCTATTAACAGACCGTAATTTAAA

TACATCATTCTTCGACCCAGCGGGAGGAGGAGACCCAATTCTATATCAACATTTATTT

>N9_983_Nais_communis_2ind

TACATTATATTTAATTCTAGGAGTATGAGCAGGAATAATCGGAACAGGGACAAGAATACT

AATTCGAATTGAATTAGCTCAACCCGGAGCCTTCTTAGGAAGAGACCAACTATACAACAC

ATTAGTTACTGCACACGCGTTTTTAATAATTTTCTTTCTTGTTATACCTGTATTCATTGG

TGGATTCGGAAACTGACTTCTACCTCTAATACTTGGTGCACCAGATATAGCATTCCCACG

ACTTAATAATCTTAGGTTCTGATTACTCCCCCCATCATTAATTCTTTTAGTTTCATCCGC

GGCCGTTGAAAAAGGAGCCGGAACAGGTTGAACTGTTTACCCACCACTATCAAGAAACCT

GGCCCATGCAGGACCATCTGTAGACATGGCTATTTTCTCACTACATTTAGCCGGGGCATC

ATCTATTTTAGGAGCAGTAAATTTTATTACAACAGTAATAAATATACGATGAAACGGTAT

ACGATTAGAACGACTACCACTATTTGTATGAGCTGTAATACTTACTGTAATCCTTCTACT

ACTATCACTACCAGTTCTAGCAGGAGCAATTACTATACTATTAACAGACCGTAATTTAAA

TACATCATTCTTCGACCCCGCTGGAGGGGGAGACCCAATCTTATATCAACATTTATTC

>N5_966_Ophidonais_serpentina_6ind

------------------AGGAGTATGAGCAGGAATAGTTGGTACAGGAACAAGAATACT

GATTCGAATTGAACTAGCTCAACCAGGAGCTTTTCTAGGAAGAGATCAATTATATAACAC

TCTAGTAACAGCACATGCGTTTTTAATAATTTTCTTTTTAGTTATACCTGTATTTATTGG

CGGATTCGGAAACTGACTTCTTCCATTAATATTAGGTGCTCCAGATATGGCATTCCCACG

ACTAAATAATCTTAGATTCTGACTTCTACCACCATCATTAATTCTATTAATTTCATCTGC

AGCCGTTGAAAAAGGTGCTGGAACAGGATGAACTGTATATCCTCCATTATCAAGAAATCT

AGCTCACGCTGGACCTTCAGTTGACATGGCTATTTTTTCACTACATCTAGCAGGTGCATC

TTCTATTTTAGGTGCAGTTAACTTCATTACTACAGTAATAAACATACGATGAAATGGAAT

ACGACTTGAACGAGTACCATTATTTGTATGAGCTGTAACACTTACTGTAATTCTTCTTCT

TTTATCATTACCTGTATTAGCTGGTGCAATTACCATACTATTAACAGATCGAAATCTAAA

TACCTCATTCTTCGATCCTGCAGGAGGGGGAGACCCAATTTTATACCAACATTTATTC

>N12_984_Nais_stolci_pardalis_2ind

TACACTATATCTAATTTTAGGAGTATGAGCAGGAATAGTAGGAACTGGTACAAGATTATT

AATTCGAATTGAACTATCACAACCAGGATCATTTCTTGGAAGAGATCAACTATATAATAC

TCTCGTAACAGCCCACGCATTCTTAATAATTTTCTTCTTAGTAATACCTGTATTTATTGG

GGGGTTTGGAAACTGACTTCTTCCATTAATACTAGGTGCTGCTGATATGGCATTCCCACG

ACTAAACAACCTTAGATTTTGACTACTACCACCATCACTAATTCTATTAGTTTCTTCTGC

TGCTGTAGAAAAAGGAGCCGGCACAGGATGAACAGTATATCCACCACTATCAAGAAATCT

AGCCCATGCTGGACCTTCAGTAGATATGGCTATTTTTTCACTTCATTTAGCAGGTGCTTC

TTCTATTTTAGGAGCTGTAAACTTTATTACGACTGTAATAAACATGCGTTGAAATGGAAT

ACGATTAGAACGACTACCACTATTTGTATGAGCTGTATTCCTTACAGTAATTCTTCTACT

ATTATCTCTTCCAGTTCTTGCCGGAGCTATTACAATGCTTCTAACAGACCGAAACCTTAA

TACTTCATTCTTCGACCCTGCTGGTGGTGGAGACCCGATCCTTTACCAACACTTATTC

>N10_965_Nais_communis

---------------TTTAGGAGTATGAGCAGGAATAGTAGGAACTGGAACTAGATTACT

TATTCGAATTGAATTATCACAACCAGGATCATTTCTTGGAAGAGATCAATTATATAATAC

TCTTGTAACAGCACACGCGTTCTTAATAATTTTCTTCTTAGTAATACCAGTATTTATTGG

GGGGTTCGGAAACTGACTTCTCCCACTAATACTAGGTGCTGCTGATATAGCATTCCCACG

ACTAAACAATCTTAGATTTTGACTACTACCACCATCATTAATTCTATTAATTTCTTCTGC

TGCTGTAGAAAAAGGTGCAGGAACAGGATGAACTGTTTATCCGCCTCTATCAAGAAATCT

AGCACACGCTGGACCTTCAGTAGACATGGCCATTTTCTCACTTCACTTAGCAGGTGCTTC

TTCTATTTTAGGGGCAGTAAATTTCATTACAACAGTAATAAATATACGATGAAACGGAAT

ACGATTAGAACGACTTCCACTATTCGTATGAGCAGTATTTCTTACAGTAATTCTCCTTCT

TCTATCACTTCCCGTTCTTGCTGGTGCAATTACAATACTATTAACAGATCGAAATCTTAA

TACCTCATTCTTCGATCCTGCTGGTGGTGGAGATCCGATCTTATATCAACACTTATTC

>N11_1029_Nais_christinae

TACACTTTATCTAATTTTAGGAGTATGAGCAGGAATAGTAGGAACCGGAACAAGATTACT

TATTCGAATTGAATTATCACAACCAGGATCATTTCTTGGAAGAGATCAATTATATAATAC

TCTTGTAACAGCACATGCATTCTTAATAATTTTCTTCTTAGTAATACCTGTATTTATTGG

GGGGTTCGGAAACTGACTTCTTCCACTAATACTAGGAGCTGCTGATATGGCATTTCCTCG

ATTAAACAATCTTAGATTTTGATTACTACCACCTTCATTAATTCTATTAATTTCGTCTGC

AGCAGTAGAAAAAGGTGCAGGAACTGGATGAACTGTATACCCTCCTCTATCTAGAAATCT

AGCTCATGCTGGGCCTTCAGTAGATATGGCTATTTTCTCACTTCATTTAGCAGGTGCTTC

TTCTATTCTTGGAGCAGTAAATTTTATTACAACTGTAATAAACATACGTTGAAACGGAAT

GCGATTAGAACGACTTCCATTATTTGTATGAGCAGTATTTCTTACAGTAATTCTCCTTCT

TCTATCTCTCCCAGTTCTTGCTGGGGCAATCACCATATTACTAACAGATCGAAATCTAAA

CACTTCATTCTTTGATCCTGCTGGTGGTGGAGATCCAATTTTATATCAACATTTATTC

>N8_960_Nais_alpina_2ind

TACACTATATTTAATTTTAGGAGTATGAGCAGGAATAGTGGGAACTGGAACAAGATTACT

TATTCGAATTGAACTATCACAACCAGGATCATTTCTTGGAAGAGATCAACTATACAACAC

TCTTGTAACAGCACATGCATTCTTAATAATTTTCTTCTTGGTAATACCAGTATTCATTGG

GGGGTTTGGAAACTGACTTCTTCCATTAATACTAGGTGCTGCCGATATAGCATTTCCACG

ACTTAATAATCTTAGATTTTGATTGCTACCACCATCATTAATTTTATTAATTTCTTCTGC

AGCTGTAGAAAAAGGTGCTGGAACAGGATGAACTGTTTATCCGCCTCTATCAAGAAATCT

AGCACATGCCGGACCTTCTGTAGATATGGCTATTTTTTCACTTCATTTAGCAGGTGCTTC

ATCTATTTTAGGAGCAGTAAATTTTATTACTACAGTAATAAATATACGATGAAATGGAAT

ACGACTAGAACGGCTACCATTATTTGTTTGAGCAGTATTTCTTACAGTAATTCTTCTTTT

ATTATCTCTTCCAGTACTTGCTGGGGCAATTACAATACTATTAACAGATCGAAATCTTAA

TACTTCATTTTTTGATCCTGCTGGAGGTGGGGATCCAATCTTATATCAACATCTATTT

>N14_1010_Uncinais_uncinata

TACACTATACTTAATTTTAGGAGTATGAGCGGGAATAGTAGGAACTGGAACTAGATTACT

TATTCGAATTGAACTATCACAACCAGGATCATTTCTTGGAAGAGATCAATTATATAACAC

TCTTGTAACAGCACATGCATTCTTAATAATTTTCTTCTTAGTAATACCAGTATTTATTGG

GGGATTTGGCAACTGACTTCTCCCATTAATACTAGGTGCTGCCGATATAGCATTTCCTCG

ATTAAATAATCTTAGATTTTGATTACTTCCACCATCATTAATTCTATTAGTTTCTTCTGC

TGCAGTAGAAAAAGGTGCAGGAACAGGATGAACTGTATACCCACCACTATCAAGAAATCT

AGCTCACGCTGGCCCTTCTGTAGATATGGCTATTTTTTCACTACACTTAGCTGGTGCTTC

TTCTATTCTAGGAGCAGTTAATTTTATCACCACTGTAATAAATATACGTTGAAACGGAAT

ACGACTAGAACGACTTCCACTATTTGTTTGATCAGTATTCCTTACAGTAATTCTTCTCTT

ATTATCTCTTCCTGTACTTGCTGGTGCAATTACAATACTATTAACAGATCGAAACCTAAA

TACCTCATTCTTCGATCCTGCTGGTGGTGGAGACCCTATTCTATATCAACATTTATTC

>N13_1039_Nais_pseudobtusa

TACACTATATTTAATTTTAGGAGTATGAGCAGGAATAGTAGGAACTGGAACTAGATTACT

TATTCGAATTGAACTATCACAACCAGGATCATTCCTTGGAAGAGATCAATTATATAATAC

TCTTGTAACAGCACATGCATTCTTAATAATTTTCTTCTTAGTAATACCAGTATTTATTGG

AGGATTCGGTAACTGACTGCTTCCACTAATACTAGGTGCTGCCGATATAGCATTCCCACG

ATTAAACAATCTTAGATTTTGACTTCTTCCACCATCATTAATTCTATTAGTTTCTTCTGC

CGCTGTAGAAAAAGGTGCGGGAACAGGATGAACTGTATATCCACCTCTATCAAGAAATCT

AGCGCACGCTGGACCTTCTGTTGATATGGCTATTTTTTCACTTCATTTAGCTGGTGCTTC

TTCTATTTTAGGAGCAGTAAATTTTATCACTACTGTAATAAATATACGATGAAATGGAAT

ACGATTAGAACGACTTCCACTGTTTGTATGAGCTGTATTTCTTACAGTAATTCTTCTTTT

ACTTTCTCTTCCAGTTCTTGCTGGTGCAATTACTATACTATTAACTGATCGAAATCTTAA

TACTTCATTCTTCGATCCTGCTGGAGGTGGAGATCCAATTCTATATCAACATCTATTC

>N2_LN810267_Nais_bretscheri

TACACTATATTTAATTTTAGGAGTATGAGCAGGAATAGTAGGAACTGGAACAAGACTACT

TATTCGAATTGAACTATCACAACCAGGATCATTTCTTGGAAGAGACCAATTATATAATAC

TCTTGTAACAGCACATGCATTCTTAATAATTTTCTTCTTAGTAATACCTGTATTTATTGG

GGGATTTGGAAATTGACTTCTCCCACTAATACTAGGTGCTGCTGATATAGCATTTCCACG

ACTAAATAATCTTAGATTTTGATTACTACCACCATCATTAATTCTATTAGTTTCTTCTGC

AGCTGTAGAAAAAGGAGCTGGAACAGGATGAACAGTATACCCACCACTATCAAGGAATCT

AGCTCATGCCGGACCATCTGTAGACATGGCTATCTTTTCACTTCACTTGGCAGGTGCCTC

TTCTATTTTAGGGGCAGTAAATTTTATTACAACTGTAATAAATATACGTTGAAATGGTAT

ACGATTAGAACGACTACCATTATTTGTATGAGCTGTATTTCTTACAGTAATCCTTCTGCT

ACTTTCTCTTCCAGTTCTTGCTGGAGCTATTACCATACTACTAACAGACCGAAACTTAAA

TACTTCATTCTTTGATCCTGCTGGAGGAGGAGACCCAATTCTATATCAACATCTATTC

>N15_1041_Chaetogaster_diastrophus

CACTCTATACTTAATTTTAGGAGTTTGAGCAGGAATAATTGGTACAGGAACTAGAATACT

AATTCGAATTGAACTATCACAACCAGGATCATTCCTTGGGAGAGATCAATTATATAACAC

TCTAGTTACAGCCCATGCATTCCTAATAATTTTCTTCTTAGTGATACCAGTATTCATTGG

TGGATTCGGAAACTGACTTCTTCCTCTAATACTAGGTGCTCCAGATATGGCATTCCCACG

ACTTAATAATTTAAGATTTTGACTTTTACCTCCATCACTAATTTTACTTATTTCATCAGC

AGCGGTGGAAAAAGGAGCAGGAACAGGATGAACTGTATACCCTCCTCTATCTAGAAATCT

TGCCCATGCAGGACCGTCCGTAGACATGGCTATTTTTTCTCTTCACTTAGCAGGTGCTTC

ATCTATTTTAGGAGCAGTGAATTTTATTACAACTGTAATTAACATACGATGAAACGGAAT

ACGACTAGAACGACTTCCTCTATTTGTATGAGCAGTATTCTTAACAGTCATTCTTCTTCT

ACTTTCTCTTCCAGTACTTGCCGGAGCTATTACTATACTATTAACAGATCGAAACCTAAA

TACTTCTTTCTTTGATCCAGCTGGTGGTGGTGACCCTATTCTATACCAACATCTATTT

>N4_888_Nais_elinguis_13ind

TACATTATACTTAATTCTAGGAGTATGAGCGGGAATAGTTGGAACTGGAACAAGAATACT

AATTCGAATTGAACTTGCTCAACCAGGAGCCTTCCTCGGAAGAGACCAACTCTATAACAC

CCTAGTTACAGCTCATGCATTTTTAATAATTTTCTTTCTTGTAATGCCAGTATTTATTGG

TGGATTCGGAAATTGACTTCTACCACTAATACTTGGAGCCCCAGATATAGCATTTCCACG

ATTAAATAATCTAAGATTTTGATTACTACCACCATCATTAATTATACTAATTTCATCAGC

CGCAGTTGAAAAAGGTGCAGGAACAGGATGAACTGTTTACCCTCCATTATCAAGAAATTT

AGCTCATGCTGGACCATCAGTTGACATGGCTATTTTTTCTCTTCATTTAGCAGGTGCATC

ATCTATTTTAGGTGCAGTAAATTTTATTACTACAGTAATAAATATACGATGAAACGGTAT

ACGATTAGAACGTTTACCTTTATTTGTATGAGCTGTATTCCTTACTGTAATTCTCCTTCT

ACTATCGCTACCAGTATTAGCTGGCGCTATTACAATACTTCTTACAGACCGTAATTTAAA

TACCTCATTCTTTGATCCAGCAGGAGGAGGAGATCCGATTCTTTATCAACACTTATTT

>N6_LN810254_Piguetiella_blanci_3ind

TACATTATATTTAATTTTAGGAGTATGAGCAGGAATAGTTGGAACAGGAACAAGAATATT

AATTCGAATTGAACTAGCTCAACCAGGATCATTCCTAGGAAGAGATCAGCTATACAATAC

TCTTGTAACAGCACATGCATTCTTAATAATTTTCTTTTTAGTTATACCTGTATTCATTGG

TGGGTTTGGAAACTGACTACTTCCATTAATACTTGGTGCCCCAGATATGGCATTTCCACG

ACTAAACAACTTAAGATTCTGATTATTACCACCTTCACTAATTCTTCTAGTCTCATCAGC

TGCAGTAGAAAAAGGAGCAGGAACAGGCTGAACTGTATATCCACCACTATCTAGAAATCT

AGCACACGCTGGCCCATCAGTTGACATGGCTATTTTCTCTCTTCATTTAGCAGGTGCATC

CTCTATTTTAGGTGCAGTTAATTTTATTACAACAGTAATAAACATACGATGAAATGGAAT

ACGATTAGAACGAGTACCGTTATTTGTATGAGCTGTAACGCTTACCGTTATTCTTCTTCT

ACTATCACTGCCGGTACTTGCAGGTGCAATTACAATACTACTAACAGATCGAAATCTAAA

TACTTCATTCTTCGATCCTGCTGGTGGTGGAGATCCAATTCTATATCAACATTTATTC

>N3_LN810253_Nais_communis

TACATTATATTTAATTTTAGGAGTATGAGCAGGAATGGTTGGAACAGGAACAAGACTATT

AATTCGAATTGAACTTGCCCAACCAGGATCTTTCCTAGGAAGAGATCAATTATATAACAC

TCTTGTGACTGCACATGCATTTTTAATAATTTTCTTCTTAGTTATGCCAGTATTTATTGG

TGGTTTTGGAAACTGACTACTACCTCTAATATTAGGAGCACCTGACATAGCATTTCCACG

ATTAAATAACCTTAGATTTTGATTACTACCACCTTCACTAATTCTATTAGTATCATCTGC

CGCTGTAGAAAAAGGAGCCGGAACAGGATGAACTGTATATCCGCCACTATCAAGAAACTT

AGCACATGCAGGACCATCAGTTGACATGGCTATTTTCTCATTACACTTAGCAGGTGCATC

ATCTATTTTAGGTGCAGTAAACTTTATTACAACAGTAATAAATATACGATGAAATGGAAT

ACGACTAGAACGAGTCCCATTATTTGTATGAGCAGTTCTACTTACCGTAATTCTACTTCT

ACTATCATTACCAGTACTTGCAGGAGCAATTACAATACTACTAACAGATCGAAATCTAAA

TACTTCATTCTTCGATCCAGCAGGAGGGGGAGATCCAATTCTATACCAACATTTATTT

>LL2_1019_Lumbriculus_variegatus_3ind

CACTCTATATTTTATTCTTGGCGTCTGAGCCGGCATAGTAGGAGCAGGAATAAGACTACT

AATCCGAATTGAGCTCACACAACCAGGATCATTTCTAGGCAGAGATCAACTATACAATAC

CATAGTTACCGCACATGCCTTTATTATAATTTTCTTTATAGTAATACCTATATTTATTGG

CGGATTCGGAAATTGATTACTACCATTAATACTAGGTGCTCCAGACATAGCATTTCCACG

ACTAAATAATCTTAGTTTTTGACTACTACCCCCTTCCCTAATTTTATTAGTAAGATCAGC

TGCAGTAGAAAAAGGAGCAGGAACTGGATGAACTGTATACCCACCTCTAGCAAGTAATCT

AGCACACGCTGGACCTTCTGTAGATCTAGCTATCTTCTCCCTTCATTTAGCCGGAGCATC

CTCAATTCTAGGAGCCCTAAACTTTATTACAACTGTTATCAATATACGATGAAATGGTAT

ACGTCTAGAACGAATTCCTTTATTTGTATGAGCCGTAATAATTACAGTAATTTTACTCCT

ATTATCCTTACCGGTATTAGCAGGAGCCATTACTATATTACTAACAGATCGAAACCTAAA

CACCTCCTTCTTTGATCCAGCTGGGGGTGGAGACCCTGTCCTATATCAACATCTATTT

>E12_806_Fridericia_sp

--CACTCTTCTTCATTTTAGGAGTTTGAGCAGGAATAATAGGAGCCGCTATAAGACTTCT

AATTCGAATTGAACTTAGACAACCAGGATCATTTCTTGGCAGAGACCAACTATATAATAC

AATTGTTACAGCTCATGCATTTCTTATAATCTTTTTTTTAGTGATACCAGTATTTATTGG

GGGATTTGGAAACTGACTCTTACCATTAATATTAGGTGCACCAGATATAGCATTCCCACG

TCTAAATAATATAAGATTTTGATTACTTCCTCCGTCTCTAATACTTCTTCTATCATCTGC

CGCAGTAGAAAAAGGTGCAGGTACAGGATGAACAGTATACCCACCATTAGCAAGAAATAT

AGCCCACGCAGGTCCATCAGTAGATTTAGCTATTTTTTCTCTACATTTAGCAGGAGCTTC

ATCTATTCTAGGAGCCGTTAATTTTATTTCAACAGTAATCAATATACGCTGACAAGGTCT

TCAACTTGAACGAATTCCATTATTTGTATGAGCTGTCACTATTACTGTTGTACTCTTATT

ATTATCCTTACCAGTATTAGCTGGTGCTATTACCATACTTCTCACAGATCGTAACCTAAA

TACATCTTTCTTCGACCCCGCTGGCGGAGGAGACCCTATTTTTGTATCAACACTATTT

>E5_817_Marionina_argentea

--CTCTATATTTTATTTTAGGAACTTGAGCTGGGATATTAGGAGCAGCTATAAGATTATT

AATTCGTTTTGAACTAGGTCAACCTGGCTCATTTTTAGGTAGAGATCAATTATATAATAC

TATTGTAACTGCTCATGCCTTTTTAATAATTTTTTTTTTAGTGATACCTGTCTTTATTGG

AGGATTTGGAAATTGATTATTACCACTAATATTAGGTGCGCCAGACATGGCTTTCCCTCG

ACTTAATAATTTGAGATTTTGGCTATTACCCCCATCTTTGCTACTTTTAGTGTCTTCAGC

TGCGGTAGAAAAGGGGGCGGGAACAGGTTGGACAGTGTACCCACCACTTTCGTCAAATAT

TGCCCATTCAGGTCCATCTGTAGACTTAGCTATCTTTTCTCTTCATCTAGCAGGAGCATC

ATCTATTTTAGGGGCCATTAATTTTATTACAACTGTAATTAATATACGATGACGGGGATT

GCAATTAGAGCGTATTCCTTTATTTGTTTGAGCAGTTACTATTACAGTTGTGCTATTATT

ACTATCTTTACCTGTATTAGCTGGAGCTATTACTATATTGTTAACTGATCGTAATTTAAA

TACATCATTTTTTGATCCTGCTGGAGGAGGGGACCCAATTTTATATCAACACTTATTT

>LL1_LN810271_Lumbriculidae_sp_2ind

GACTCTATACTTCATTTTAGGAGTTTGAGCAGGAATAGTAGGAGCTGGTATAAGCCTTTT

AATTCGAATTGAATTGACCCAGCCAGGGTCATTCCTAGGAAGAGATCAACTATATAATAC

TATAGTAACAGCTCATGCTTTTATCATAATTTTTTTTATAGTAATACCCATATTTATTGG

GGGGTTTGGAAATTGAATACTCCCATTAATACTAGGAGCACCAGATATAGCTTTCCCTCG

ATTAAATAATCTAAGTTTTTGGTTACTTCCTCCATCACTTATTCTACTAGTTTCTTCTGC

TGCAGTAGAAAAAGGTGCTGGTACTGGATGAACAGTATACCCCCCATTAGCAAGAAATCT

TGCACATAGAGGGCCTTCAGTTGATTTAGCAATCTTTTCATTACATCTTGCTGGTGCCTC

GTCTATTTTAGGAGCTTTAAATTTTATTACTACCGTAGTAAATATGCGATGAAACGGGCT

ACGATTAGAGCGAATTCCTTTATTCGTTTGAGCTGTGACAATTACAGTGGTTCTATTACT

TTTGTCACTTCCTGTACTTGCAGGCGCAATTACAATACTTCTTACTGATCGAAACTTAAA

TACTTCTTTTTTTGACCCAGCAGGGGGTGGAGACCCAGTTCTATACCAGCATTTATTC

>LL3_682_Lumbriculidae_sp

GACCTTATATTTCATCTTAGGAGTTTGAGCTGGAATAGTGGGAGCAGGCATAAGACTATT

AATTCGAGTTGAATTAACACAACCTGGGTCATTTTTAGGAAGTGACCAACTATACAATAC

TATTGTTACGGCTCACGCCTTTATTATAATTTTCTTTATAGTCATACCTATATTTATTGG

TGGTTTTGGTAACTGAATACTACCATTAATATTAGGAGCACCAGATATAGCATTCCCACG

GCTTAACAACCTAAGATTTTGGTTACTTCCACCATCTTTAACTTTATTAGTAGCATCCGC

TGCAGTTGAAAAAGGAGCAGGTACAGGATGAACAGTTTACCCGCCTCTATCAAGAAATTT

AGCCCATGCAGGTCCATCAGTAGATCTAGCAATCTTTTCACTTCATCTAGCCGGAGCATC

CTCCATTCTAGGAGCTATTAACTTTATTACAACAGTAATTAATATACGATGAAATGGACT

ACGACTTGAACGAGTTCCATTATTTGTATGAGCAGTAACAATTACAGTAGTACTCTTACT

ATTATCTTTACCAGTACTAGCTGGGGCAATCACTATACTTCTTACAGATCGAAACTTAAA

TACAACATTTTTTGATCCAGCGGGAGGTGGAGACCCAGTACTA---------------

>LL3_957_Stylodrilus_heringianus_3ind

AACCTTATATTTCATCTTAGGAGTTTGAGCTGGGATAGTAGGAGCAGGGATGAGACTATT

AATTCGAGTTGAATTAACACAACCCGGGTCATTTTTAGGAAGTGACCAACTATATAATAC

TATTGTTACGGCTCACGCATTTATTATAATTTTCTTTATAGTTATACCTATATTTATTGG

TGGTTTTGGTAACTGAATACTACCGTTAATATTAGGAGCACCAGACATAGCATTTCCACG

ACTTAACAACCTAAGATTTTGATTACTACCACCTTCTTTAACTTTATTAGTAGCATCGGC

TGCAGTAGAAAAAGGAGCCGGCACAGGATGAACAGTTTATCCACCTCTATCAAGAAATTT

AGCTCATGCTGGACCCTCAGTAGACCTGGCAATCTTCTCACTCCATCTAGCAGGAGCCTC

CTCCATTTTAGGAGCCATTAACTTTATTACGACTGTAATTAATATACGATGAAATGGACT

ACGACTTGAACGAGTTCCATTATTTGTATGAGCAGTAACAATTACAGTAGTTCTATTACT

ATTATCATTACCAGTACTAGCTGGAGCAATCACTATACTTCTTACAGATCGAAACTTAAA

TACAACATTTTTTGATCCAGCTGGAGGTGGAGACCCCGTACTATATCAACATTTATTT

>LL3_918_Stylodrilus_heringianus_48ind

AACCTTATATTTCATCTTAGGAGTTTGAGCTGGAATAGTAGGAGCAGGAATAAGACTATT

AATTCGAGTTGAATTAACACAACCAGGATCATTTTTAGGAAGTGATCAATTATATAATAC

TATTGTTACGGCCCACGCCTTTATTATAATTTTCTTTATAGTTATACCTATATTTATTGG

TGGCTTTGGTAACTGAATATTACCATTAATATTAGGAGCACCAGATATAGCATTCCCACG

ACTTAACAACCTAAGATTTTGGTTACTCCCGCCTTCTTTAACTTTATTAGTAGCATCGGC

TGCAGTTGAAAAGGGAGCCGGCACAGGATGAACAGTTTATCCACCTCTATCAAGAAATTT

AGCTCATGCAGGTCCATCAGTAGACCTAGCAATCTTTTCACTACATCTAGCTGGGGCATC

CTCCATTCTAGGAGCTATTAACTTTATTACTACGGTAATTAATATACGATGAAATGGACT

CCGACTTGAACGAGTTCCATTATTTGTATGAGCAGTAACAATTACAGTTGTTCTATTACT

ATTATCGTTACCAGTACTAGCTGGGGCAATCACTATACTTCTTACAGATCGAAACTTAAA

TACAACATTTTTCGATCCGGCTGGTGGTGGAGACCCAGTATTATATCAACATTTATTT
