# Supplementary material for: Cytochrome c oxidase barcodes for aquatic oligochaete identification: development of a Swiss reference database
Source: PeerJ. 2017 Dec 6;5:e4122. doi: 10.7717/peerj.4122 (PMC5723135; doi:10.7717/peerj.4122)
Supplement: File S2 — All the sequences (COI and ITS2) obtained in the present work. [file peerj-05-4122-s006.docx]

**Supplemental File S2 : Raw data 2.** All the sequences (COI and ITS2) obtained in the present work

**COI sequences**

>819_Achaeta_sp_E7

-----CTCTATNCTTTATCCTCGGAATATGAGCTGGCATAATAGGTGCAGCTATAAGACT

CCTCATCCGATTTGAACTAAGTCAACCCGGATCATTTTTAGGGAGAGACCAATTATATAA

TACCATTGTAACAGCACATGCATTCTTAATAATTTTCTTTCTTGTAATACCAGTATTTAT

TGGAGGATTTGGAAATTGACTCATTCCATTAATACTTGGAGCTCCAGATATAGCTTTTCC

ACGATTAAATAATATAAGATTCTGATTACTTCCACCATCCCTCATATTACTTCTTTCTTC

TACAGCTGTAGAAAAAGGTATAGGAACAGGATGAACAGTATATCCTCCTTTAGCTAGAAA

CATTGCTCATGCAGGACCATCTGTAGATTTAGCCATTTTCTCTCTTCACTTAGCAGGAGC

TTCATCAATTCTAGGAGCAGTAAATTTCATTACTACTGTAATTAATATACGATGACAAGG

TATACGACTAGAACGAATTCCACTATTTGTATGAGCTATAATAATCACAGTAGCCCTCCT

ACTTTTAGCACTACCAGTCCTAGCTGGCGCAATTACTATGCTTCTTACTGATCGAAACCT

TAACACATCATTCTTTGATCCAGCTGGTGGAGGTGACCCTATTCTATATCAACATTTATT

T--------------------------------------------

>783_Tubifex_tubifex_T27

-----CCCTTTACATACTATTTGGTATTTGAGCAGGTATAGTTGGCACCGGAACAAGTCT

ACTAATTCGTCTAGAACTAGCTCAACCTGGCTCTTTCCTCGGCAGCGACCAATTATATAA

TACATTAGTTACAGCACATGCATTCCTTATAATCTTCTTTATGGTAATACCTATTTACAT

TGGGGGATTCGGAAATTGACTAGTCCCATTAATACTGGGGGCACCAGACATAGCATTCCC

ACGACTAAATAATCTTAGATTTTGACTTCTACCCCCCTCCCTAATTCTACTTGTATCATC

TGCTGCAGTGGAAAAAGGAGCTGGAACAGGGTGAACTGTCTACCCACCACTAGCCAGTAA

CTTAGCACACTCTGGACCCTCAGTAGACTTAGCAATCTTCTCATTACACTTAGCTGGTGT

AGCCTCAATTCTAGGTGCCATTAACTTCATCACTACAATAATTAATATACGTTGAAAAGG

TATACGCCTAGAACGAATCCCATTATTTGTTTGATCAGTAATTATTACTGTAATTCTTTT

ACTACTTACACTTCCAGTACTAGCCGGTGCAATTACCATACTACTAACAGACCGAAATCT

AAATACTTCATTCTTCGACCCTGCCGGTGGGGGAGACCCTGTTCTTTATCAAAAATCATT

C--------------------------------------------

>784_Tubifex_tubifex_T27

---AACCCTTTACATACTATTTGGTATTTGAGCAGGTATAGTTGGCACCGGAACAAGTCT

ACTAATTCGTCTAGAACTAGCTCAACCTGGCTCTTTCCTCGGCAGCGACCAATTATATAA

TACATTAGTTACAGCACATGCATTCCTTATAATCTTCTTTATGGTAATACCTATTTACAT

TGGGGGATTCGGAAATTGACTAGTCCCATTAATACTGGGGGCACCAGACATAGCATTCCC

ACGACTAAATAATCTTAGATTTTGACTTCTACCCCCCTCCCTAATTCTACTTGTATCATC

TGCTGCAGTGGAAAAAGGAGCTGGAACAGGGTGAACTGTCTACCCACCACTAGCCAGTAA

CTTAGCACACTCTGGACCCTCAGTAGACTTAGCAATCTTCTCATTACACTTAGCTGGTGT

AGCCTCAATTCTAGGTGCCATTAACTTCATCACTACAATAATTAATATACGTTGAAAAGG

TATACGCCTAGAACGAATCCCATTATTTGTTTGATCAGTAATTATTACTGTAATTCTTTT

ACTACTTACACTTCCAGTACTAGCCGGTGCAATTACCATACTACTAACAGACCGAAATCT

AAATACTTCATTCTTCGACCCTGCCGGTGGGGGAGACCCTGTTCTTTATCAAAATCTATT

C--------------------------------------------

>786_Tubifex_tubifex_T27

---AACCCTTTACATACTATTTGGTATTTGAGCAGGTATAGTTGGCACCGGAACAAGTCT

ACTAATTCGTCTAGAACTAGCTCAACCTGGCTCTTTCCTCGGCAGCGACCAATTATATAA

TACATTAGTTACAGCACATGCATTCCTTATAATCTTCTTTATGGTAATACCTATTTACAT

TGGGGGATTCGGAAATTGACTAGTCCCATTAATACTGGGGGCACCAGACATAGCATTCCC

ACGACTAAATAATCTTAGATTTTGACTTCTACCCCCCTCCCTAATTCTACTTGTATCATC

TGCTGCAGTGGAAAAAGGAGCTGGAACAGGGTGAACTGTCTACCCACCACTAGCCAGTAA

CTTAGCACACTCTGGACCCTCAGTAGACTTAGCAATCTTCTCATTACACTTAGCTGGTGT

AGCCTCAATTCTAGGTGCCATTAACTTCATCACTACAATAATTAATATACGTTGAAAAGG

TATACGCCTAGAACGAATCCCATTATTTGTTTGATCAGTAATTATTACTGTAATTCTTTT

ACTACTTACACTTCCAGTACTAGCCGGTGCAATTACCATACTACTAACAGACCGAAATCT

AAATACTTCATTCTTCGACCCTGCCGGTGGGGGAGACCCTGTTCTTTATCAACATCTATT

C--------------------------------------------

>787_Limnodrilus_hoffmeisteri_T18

--------------------------------------------------------GCCT

GCTAATTCGATTCGAATTAGCACAACCTGGTTCATTCCTCGGTAGAGATCAACTCTATAA

TACCTTAGTGACAGCCCACGGTTTCCTTATAATCTTCTTCATGGTAATACCAATTTTTAT

TGGTGGCTTCGGAAATTGACTAGTCCCCCTAATACTAGGAGCTCCTGACATGGCCTTTCC

ACGACTAAATAACCTAAGATTTTGACTAATACCTCCATCACTCATTCTATTAGTTTCATC

AGCCGCAGTCGAAAAGGGAGCGGGGACAGGGTGAACTGTATACCCCCCTCTAGCCAGAAA

CCTAGCGCATTCTGGGCCATCTGTGGATCTAGCAATCTTCTCTCTTCACTTAGCCGGGGC

TGCATCAATTCTAGGTGCTATTAACTTCATTACCACAATAATTAATATACGATGAAAAGG

AATGCGCCTAGAGCGCATTCCTCTATTTGTATGATCAGTAATCATTACAGTTGTCCTCCT

TCTTCTTACATTACCGGTTTTAGCGGGGGCAATCACCATACTTTTAACAGACCGAAACTT

AAACACCTCATGGTCATACAACATCATAAA------------------------------

---------------------------------------------

>789_Tubifex_tubifex_T27

---AACCCTTTACATACTATTTGGTATTTGAGCAGGTATAGTTGGCACCGGAACAAGTCT

ACTAATTCGTCTAGAACTAGCTCAACCTGGCTCTTTCCTCGGCAGCGACCAATTATATAA

TACATTAGTTACAGCACATGCATTCCTTATAATCTTCTTTATGGTAATACCTATTTACAT

TGGGGGATTCGGAAATTGACTAGTCCCATTAATACTGGGGGCACCAGACATAGCATTCCC

ACGACTAAATAATCTTAGATTTTGACTTCTACCCCCCTCCCTAATTCTACTTGTATCATC

TGCTGCAGTGGAAAAAGGAGCTGGAACAGGGTGAACTGTCTACCCACCACTAGCCAGTAA

CTTAGCACACTCTGGACCCTCAGTAGACTTAGCAATCTTCTCATTACACTTAGCTGGTGT

AGCCTCAATTCTAGGTGCCATTAACTTCATCACTACAATAATTAATATACGTTGAAAAGG

TATACGCCTAGAACGAATCCCATTATTTGTTTGATCAGTAATTATTACTGTAATTCTTTT

ACTACTTACACTTCCAGTACTAGCCGGTGCAATTACCATACTACTAACAGACCGAAATCT

AAATACTTCATTCTTCGACCCTGCCGGTGGGGGAGACCCTGTTCTTTATCAACATCTATT

C--------------------------------------------

>792_Limnodrilus_hoffmeisteri_T18

-----------------------------------------------------CAAGCCT

GCTAATTCGATTCGAATTAGCACAACCTGGTTCATTCCTCGGTAGAGATCAACTCTATAA

TACCTTAGTGACAGCCCACGGTTTCCTTATAATCTTCTTCATGGTAATACCAATTTTTAT

TGGTGGCTTCGGAAATTGACTAGTCCCCCTAATACTAGGAGCTCCTGACATGGCCTTTCC

ACGACTAAATAACCTAAGATTTTGACTAATACCTCCATCACTCATTCTATTAGTTTCATC

AGCCGCAGTCGAAAAGGGAGCGGGGACAGGGTGAACTGTATACCCCCCTCTAGCCAGAAA

CCTAGCGCATTCTGGGCCATCTGTGGATCTAGCAATCTTCTCTCTTCACTTAGCCGGGGC

TGCATCAATTCTAGGTGCTATTAACTTCATTACCACAATAATTAATATACGATGAAAAGG

AATGCGCCTAGAGCGCATTCCTCTATTTGTATGATCAGTAATCATTACAGTTGTCCTCCT

TCTTCTTACATTACCGGTTTTAGCGGGGGCAATCACCATACTTTTAACAGACCGAAACTT

AAACACCTCATTCTTCGATCCTGCGG----------------------------------

---------------------------------------------

>793_Tubifex_tubifex_T27

---AACCCTTTACATACTATTTGGTATTTGAGCAGGTATAGTTGGCACCGGAACAAGTCT

ACTAATTCGTCTAGAACTAGCTCAACCTGGCTCTTTCCTCGGCAGCGACCAATTATATAA

TACATTAGTTACAGCACATGCATTCCTTATAATCTTCTTTATGGTAATACCTATTTACAT

TGGGGGATTCGGAAATTGACTAGTCCCATTAATACTGGGGGCACCAGACATAGCATTCCC

ACGACTAAATAATCTTAGATTTTGACTTCTACCCCCCTCCCTAATTCTACTTGTATCATC

TGCTGCAGTGGAAAAAGGAGCTGGAACAGGGTGAACTGTCTACCCACCACTAGCCAGTAA

CTTAGCACACTCTGGACCCTCAGTAGACTTAGCAATCTTCTCATTACACTTAGCTGGTGT

AGCCTCAATTCTAGGTGCCATTAACTTCATCACTACAATAATTAATATACGTTGAAAAGG

TATACGCCTAGAACGAATCCCATTATTTGTTTGATCAGTAATTATTACTGTAATTCTTTT

ACTACTTACACTTCCAGTACTAGCCGGTGCAATTACCATACTACTAACAGACCGAAATCT

AAATACTTCATTCTTCGA------------------------------------------

---------------------------------------------

>806_Fridericia_sp_E12

-----CACTCTTCTTCATTTTAGGAGTTTGAGCAGGAATAATAGGAGCCGCTATAAGACT

TCTAATTCGAATTGAACTTAGACAACCAGGATCATTTCTTGGCAGAGACCAACTATATAA

TACAATTGTTACAGCTCATGCATTTCTTATAATCTTTTTTTTAGTGATACCAGTATTTAT

TGGGGGATTTGGAAACTGACTCTTACCATTAATATTAGGTGCACCAGATATAGCATTCCC

ACGTCTAAATAATATAAGATTTTGATTACTTCCTCCGTCTCTAATACTTCTTCTATCATC

TGCCGCAGTAGAAAAAGGTGCAGGTACAGGATGAACAGTATACCCACCATTAGCAAGAAA

TATAGCCCACGCAGGTCCATCAGTAGATTTAGCTATTTTTTCTCTACATTTAGCAGGAGC

TTCATCTATTCTAGGAGCCGTTAATTTTATTTCAACAGTAATCAATATACGCTGACAAGG

TCTTCAACTTGAACGAATTCCATTATTTGTATGAGCTGTCACTATTACTGTTGTACTCTT

ATTATTATCCTTACCAGTATTAGCTGGTGCTATTACCATACTTCTCACAGATCGTAACCT

AAATACATCTTTCTTCGACCCCGCTGGCGGAGGAGACCCTATTTTTGTATCAACACTATT

T--------------------------------------------

>807_Lumbricillus_sp_E13

-----CACTTTATTTTATCCTCGGAGTATGAGCAGGTATAATAGGCGCAGCTATAAGCCT

AATTATTCGAACAGAACTTAGCCAACCAGGATCATTCTTAGGTAGGGACCAGTTATACAA

TACTGTTGTAACAGCCCACGCATTTTTAATAATTTTCTTTTTAGTTATACCAGTATTCAT

TGGGGGCTTTGGTAATTGGCTTTTACCCCTAATACTCGGAGCACCTGATATAGCATTTCC

GCGACTCAACAATATAAGATTTTGACTTCTTCCCCCTTCACTACTTCTACTTCTCTCATC

AGCCGCAGTAGAAAAAGGTGCTGGAACTGGTTGAACAGTTTACCCACCCCTAGCAAGAAA

TATGGCACATGCAGGACCATCTGTAGATTTAGCAATTTTTTCCCTTCATTTAGCAGGGGC

TTCCTCTATTCTTGGTGCCGTAAATTTTATTTCCACTGTAATCAATATACGATGACAAGG

CCTCCAGTTAGAGCGAATTCCACTATTTGTATGAGCAGTTACAATCACAGTAGTACTTCT

TCTCTTATCTTTACCAGTATTAGCTGGTGCTATTACCATATTACTAACAGATCGAAACCT

AAATACTTCATTCTTTGACCCTGCAGGTG-------------------------------

---------------------------------------------

>808_Achaeta_sp_E6

-----CATTATATTTTATCCTAGGGATCTGAGCAGGTATAATAGGAGCAGCTATAAGCCT

ACTAATTCGATTTGAACTAAGACAACCAGGCTCATTTTTAGGAAGAGATCAACTGTACAA

CACAATTGTAACAGCTCATGCATTTTTAATAATCTTCTTCCTAGTAATACCAGTATTTAT

TGGGGGATTTGGTAACTGATTACTACCTCTAATGTTAGGTGCACCAGATATAGCATTCCC

ACGACTTAACAACATAAGATTTTGATTATTACCCCCCTCACTTATACTTCTTCTATCCTC

AGCAGCAGTTGAAAAAGGAGCAGGTACCGGATGAACAGTATACCCACCACTAGCCAGAAA

CATTGCACACGCTGGCCCATCAGTAGACCTAGCAATCTTTTCTCTACATCTTGCAGGGGC

ATCCTCAATTCTTGGAGCAGTAAACTTCATTACTACAGTAATCAATATACGATGACAAGG

AATACGATTAGAACGAATCCCCCTTTTTGTATGAGCAGTAGTAATTACAGTAGTTCTACT

ACTACTATCTCTACCAGTCCTTGCTGGGGCTATTACTATATTACTTACAGATCGCAATCT

AAACACATCATTTTTTGACCCGGCAGGTGGTGGAGACCCCATTCTATACCAACATTTATT

T--------------------------------------------

>809_Fridericia_sp_E15

-----CACTATATTTCATTCTAGGAGTATGAGCAGGCATAATAGGAGCAGCCATAAGACT

ATTAATTCGAATTGAATTAAGACAACCAGGCTCCTTTCTAGGCAGAGACCAACTATATAA

CACAATCGTAACTGCCCATGCCTTTCTCATAATTTTTTTTCTAGTAATACCAGTATTTAT

TGGGGGATTCGGAAATTGATTACTTCCATTAATACTAGGGGCACCAGACATAGCCTTCCC

GCGACTCAATAACATAAGATTCTGACTCTTGCCCCCCTCCCTTCTCCTTCTTTTATCTTC

TGCAGCAGTAGAAAAAGGTGCAGGTACAGGTTGAACAGTCTATCCTCCGCTAGCTAGAAA

TATGGCTCACGCCGGACCATCAGTAGATCTAGCTATTTTTTCTCTACATTTAGCCGGAGC

CTCATCCATTCTAGGGGCAGTAAATTTTATTTCAACCGTCATTAACATACGATGACAAGG

TCTACAATTAGAACGAATTCCATTATTTGTATGAGCTGTAACCATTACAGTAGTTCTTCT

ATTATTATCTCTACCAGTTCTAGCCGGAGCAATTACTATACTTTTAACAGATCGAAACCT

TAATACATCATTTTTTGATCCAGCCGGAGGTGGAGATCCAATTTTATACCAACACCTATT

T--------------------------------------------

>810_Henlea_perpusilla_E16

---------------------------------------GATAGGAGCAGCCATAAGCCT

TCTAATTCGAATTGAACTAAGTCAACCAGGTTCATTCCTCGGAAGAGATCAACTCTACAA

CACTATTGTTACTGCACATGCATTTCTTATAATTTTTTTCCTAGTTATACCTGTATTTAT

TGGGGGGTTCGGAAACTGACTTCTCCCATTAATACTGGGAGCCCCAGATATAGCTTTTCC

CCGACTAAATAACATAAGATTTTGACTTCTTCCCCCATCACTTCTTCTTCTACTTTCCTC

TGCAGCTGTGGAAAAAGGAGCCGGAACCGGCTGAACTGTATACCCCCCCCTATCAAGAAA

TATCGCTCATGCGGGCCCATCTGTAGACCTAGCCATTTTCTCCCTCCACTTAGCCGGAGC

ATCCTCCATCTTAGGTGCAGTAAACTTTATCACTACTGTTATTAACATACGTTGACAAGG

CCTTCACCTAGAACGAATTCCCCTATTTGTATGAGCTGTAACAATTACAGTAGTCCTTCT

CCTCTTATCTTTACCAGTGTTAGCCGGAGCAATTACTATACTACTAACTGACCGAAACCT

AAACACATCCTTTTTCGACCCAGCTGGAGGAGGTGACCCAATTCTATATCAACACCTATT

T--------------------------------------------

>811_Fridericia_sp_E14

--------------------------------------------GAGCCGCAATAAGATT

ATTAATTCGAATTGAACTCAGACAACCAGGCTCCTTCCTTGGAAGAGATCAGCTTTATAA

CACTATCGTAACAGCACATGCATTTCTAATAATCTTTTTCTTAGTAATACCAGTATTTAT

TGGAGGCTTTGGTAATTGACTTTTACCATTAATATTAGGAGCCCCAGATATAGCATTCCC

CCGACTAAATAATATAAGATTCTGATTACTACCCCCATCTTTAATACTTCTTCTATCTTC

AGCTGCAGTAGAAAAAGGTGCTGGCACTGGGTGAACTGTATACCCCCCACTAGCAAGAAA

TATAGCTCATTCTGGGCCATCCGTTGACTTAGCAATTTTTTCTCTACATCTTGCAGGAGC

ATCTTCAATTCTAGGCGCAGTAAATTTTATTTCCACAGTTATTAATATGCGATGACAAGG

CCTACAACTAGAACGAATTCCACTATTTGTATGAGCTGTTACAATCACAGTAGTATTACT

ACTACTATCTCTCCCAGTTCTAGCTGGCGCAATTACTATATTATTAACTGATCGAAATCT

AAATACATCGNTCTTCGACCCTGCTGGTGGAGGAGATCCAATCCTATATCAACACCTCTT

T--------------------------------------------

>813_Fridericia_sp_E14

---------------------------------------AATAGGAGCCGCAATAAGATT

ATTAATTCGAATTGAACTCAGACAACCAGGCTCCTTCCTTGGAAGAGATCAGCTTTATAA

CACTATCGTAACAGCACATGCATTTCTAATAATCTTTTTCTTAGTAATACCAGTATTTAT

TGGAGGCTTTGGTAATTGACTTTTACCATTAATATTAGGAGCCCCAGATATAGCATTCCC

CCGACTAAATAATATAAGATTCTGATTACTACCCCCATCTTTAATACTTCTTCTATCTTC

AGCTGCAGTAGAAAAAGGTGCTGGCACTGGGTGAACTGTATACCCCCCACTAGCAAGAAA

TATAGCTCATTCTGGGCCATCCGTTGACTTAGCAATTTTTTCTCTACATCTTGCAGGAGC

ATCTTCAATTCTAGGCGCAGTAAATTTTATTTCCACAGTTATTAATATGCGATGACAAGG

CCTACAACTAGAACGAATTCCACTATTTGTATGAGCTGTTACAATCACAGTAGTATTACT

ACTACTATCTCTCCCAGTTCTAGCTGGCGCAATTACTATATTATTAACTGATCGAAATCT

AAATACATCGTTCTTCGACCCTGCTGGTGGAGGAGATCCAATCCTATATCAACACCTCTT

T--------------------------------------------

>814_Henlea_perpusilla_E16

-----CACTATATTTCATTCTAGGCGTATGAGCCGGAATGATAGGAGCAGCCATAAGCCT

TCTAATTCGAATTGAACTAAGTCAACCAGGTTCATTCCTCGGAAGAGATCAACTCTACAA

CACTATTGTTACTGCACATGCATTTCTTATAATTTTTTTCCTAGTTATACCTGTATTTAT

TGGGGGGTTCGGAAACTGACTTCTCCCATTAATACTGGGAGCCCCAGATATAGCTTTTCC

CCGACTAAATAACATAAGATTTTGACTTCTTCCCCCATCACTTCTTCTTCTACTTTCCTC

TGCAGCTGTGGAAAAAGGAGCCGGAACCGGCTGAACTGTATACCCCCCCCTATCAAGAAA

TATCGCTCATGCGGGCCCATCTGTAGACCTAGCCATTTTCTCCCTCCACTTAGCCGGAGC

ATCCTCCATCTTAGGTGCAGTAAACTTTATCACTACTGTTATTAACATACGTTGACAAGG

CCTTCACCTAGAACGAATTCCCCTATTTGTATGAGCTGTAACAATTACAGTAGTCCTTCT

CCTCTTATCTTTACCAGTGTTAGCCGGAGCAATTACTATACTACTAACTGACCGAAACCT

AAACACATCCTTTTTCGACCCAGCTGGAGGAGGTGACCCAATTCTATATCAACACCTATT

T--------------------------------------------

>817_Marionina_argentea_E5

-----CTCTATATTTTATTTTAGGAACTTGAGCTGGGATATTAGGAGCAGCTATAAGATT

ATTAATTCGTTTTGAACTAGGTCAACCTGGCTCATTTTTAGGTAGAGATCAATTATATAA

TACTATTGTAACTGCTCATGCCTTTTTAATAATTTTTTTTTTAGTGATACCTGTCTTTAT

TGGAGGATTTGGAAATTGATTATTACCACTAATATTAGGTGCGCCAGACATGGCTTTCCC

TCGACTTAATAATTTGAGATTTTGGCTATTACCCCCATCTTTGCTACTTTTAGTGTCTTC

AGCTGCGGTAGAAAAGGGGGCGGGAACAGGTTGGACAGTGTACCCACCACTTTCGTCAAA

TATTGCCCATTCAGGTCCATCTGTAGACTTAGCTATCTTTTCTCTTCATCTAGCAGGAGC

ATCATCTATTTTAGGGGCCATTAATTTTATTACAACTGTAATTAATATACGATGACGGGG

ATTGCAATTAGAGCGTATTCCTTTATTTGTTTGAGCAGTTACTATTACAGTTGTGCTATT

ATTACTATCTTTACCTGTATTAGCTGGAGCTATTACTATATTGTTAACTGATCGTAATTT

AAATACATCATTTTTTGATCCTGCTGGAGGAGGGGACCCAATTTTATATCAACACTTATT

T--------------------------------------------

>818_Lumbriculidae_sp_LL3

------------------------------------------------------------

------------------------ACCAGGATCATTTTTAGGAAGTGATCAATTATATAA

TACTATTGTTACGGCTCACGCCTTTATTATAATTTTCTTTATAGTTATACCTATATTTAT

TGGTGGCTTTGGTAACTGAATATTACCATTAATATTAGGAGCACCAGATATGGCATTCCC

ACGACTTAACAACCTAAGATTTTGGTTACTCCCGCCTTCTTTAACTTTATTAGTAGCATC

GGCTGCAGTTGAAAAGGGAGCCGGCACAGGATGAACAGTTTATCCACCTCTATCAAGAAA

TTTAGCTCATGCAGGTCCATCAGTAGACCTAGCAATCTTTTCACTACATCTAGCTGGGGC

ATCCTCCATTCTAGGAGCTATTAACTTTATTACTACGGTAATTAATATACGATGAAATGG

ACTCCGACTTGAACGAGTTCCATTATTTGTATGAGCAGTAACAATTACAGTTGTTCTATT

ACTATTATCATTACCAGTACTAGCTGGGGCAATCACTATACTTCTTACAGATCGAAACTT

AAATACAACATTTTTCGATCCGGCTGGTGGTGGAGACCCAGTATTATATCAAACATTATT

T--------------------------------------------

>820_Vejdovskyella_intermedia_N7

-----CATTATATTTAATTTTAGGGGTATGAGCAGGAATAGTTGGAACCGGAACTAGAAT

ATTAATTCGAGTTGAATTATCACAACCAGGAGCTTTTCTTGGGAGAGACCAATTATATAA

TACATTAGTAACTGCACACGCATTCTTAATAATCTTTTTCTTAGTAATACCCGTATTTAT

TGGTGGATTCGGAAATTGACTATTACCATTAATACTAGGAGCACCGGATATAGCATTCCC

ACGACTTAATAATCTAAGATTTTGACTATTACCACCATCATTAATTCTATTAATTTCTTC

AGCTGCAGTAGAAAAAGGAGCAGGAACAGGCTGAACCGTATATCCACCACTTTCAAGAAA

TCTAGCACATGCGGGACCATCAGTAGATATAGCAATTTTTTCACTACATTTAGCTGGTGC

ATCATCTATTCTAGGAGCCGTAAATTTTATTACAACTGTAATAAATATACGATGAAATGG

AATACGATTAGAACGAGTACCATTATTCGTTTGATCGGTAATATTAACAGTTATTCTTCT

TATTCTATCATTACCTGTACTTGCGGGAGCANATACAATATTATTAACAGATCGAAACTT

AAATACCTCATTCTTCGATCCTGCTGGGGGTGGAGACCCAATTCTTTATCAAACATCATT

T--------------------------------------------

>821_Embolocephalus_velutinus_T25

-----------------------------------GTATAGTTGGAACCGGCACTAGATT

ACTAATCCGCTTTGAGCTCGCGCAACCTGGATCATTCCTAGGTAGTGACCAACTCTACAA

TACGTTAGTTACTGCTCACGCCTTCCTTATAATCTTTTTCCTAGTAATACCTGTATTCAT

CGGGGGATTCGGAAATTGACTTCTCCCATTAATATTAGGGGCACCAGATATAGCATTCCC

TCGCTTAAATAATCTAAGATTTTGGCTGCTGCCACCATCTTTAATTCTTCTAGTATCTTC

GGCGGCTGTAGAAAAGGGGGCCGGAACAGGGTGAACAGTGTACCCCCCACTATCAGGTAA

CTTAGCCCATTCGGGACCATCAGTAGACTTAGCAATCTTTTCTCTTCATTTAGCTGGTGC

ATCGTCCATTTTAGGGGCAATCAACTTCATTACTACAGTAATTAACATACGAAGTAAAGG

TATGCGGCTGGAACGGGTACCACTATTTGTATGGGCTGTCCTTCTTACAGTAATTCTTCT

CCTGTTAACCCTTCCAGTACTCGCCGGAGCTATTACTATATTACTGACAGACCGCAATCT

AAATACGTCGTTCTTTGATCCAGCTGGAGGTGGTGACCCAGTACTATACCAACACCTATT

C--------------------------------------------

>822_Spirosperma_ferox_T24

-----CCTTATATATAATTTTTGGTGTATGAGCCGGTATAGTAGGCACCGGAACTAGTCT

ATTAATTCGCTTCGAGCTTGCTCAACCAGGGTCATTCCTAGGTAGAGACCAATTATACAA

TACATTAGTAACCGCACATGCCTTCCTAATAATTTTCTTCTTAGTAATACCAGTATTTAT

TGGGGGCTTCGGCAACTGACTAATCCCACTAATATTAGGTGCCCCTGATATAGCATTCCC

ACGACTGAATAATTTAAGATTCTGACTACTTCCTCCGTCCCTTATTCTCCTTGTATCCTC

TGCCGCAGTAGAAAAAGGTGCAGGTACCGGATGAACTGTTTATCCCCCTTTAGCTGGCAA

CCTCGCCCACTCCGGCCCTTCAGTAGACTTAGCAATTTTTTCTCTCCATCTAGCCGGTGC

ATCTTCTATTTTAGGTGCAATCAACTTTATTACAACTATGGTTAACATACGAAGAAAAGG

TATACGCCTGGAACGAGTTCCTCTATTTGTTTGAGCTGTTATTTTAACAGTAATCCTCCT

ATTACTTACACTTCCTGTATTAGCCGGTGCTATCACCATACTTCTAACAGATCGTAATCT

CAACACGTCATTCTTTGACCCTGCTGGAGGGGGCGACCCTGTACTATACCAAACACCATT

C--------------------------------------------

>823_Spirosperma_ferox_T24

-----CCTTATATATAATTTTTGGTGTATGAGCCGGTATAGTAGGCACCGGAACTAGTCT

ATTAATTCGCTTCGAACTTGCTCAACCAGGGTCATTCCTAGGTAGAGACCAATTATACAA

TACATTAGTAACCGCACATGCCTTCCTAATAATTTTCTTCTTAGTAATACCAGTATTTAT

TGGGGGCTTCGGCAACTGACTAATCCCACTAATATTAGGTGCCCCTGATATAGCATTCCC

ACGACTGAATAATTTAAGATTCTGACTACTTCCTCCGTCCCTTATTCTCCTTGTATCCTC

TGCCGCAGTAGAAAAAGGTGCAGGTACCGGATGAACTGTTTATCCCCCTTTAGCTGGCAA

CCTCGCCCACTCCGGCCCTTCAGTAGACTTAGCAATTTTTTCTCTCCATCTAGCCGGTGC

ATCTTCTATTTTAGGTGCAATCAACTTTATTACAACTATGGTTAACATACGAAGAAAAGG

TATACGCCTGGAACGAGTTCCTCTATTTGTTTGAGCTGTTATTTTAACAGTAATCCTCCT

ATTACTTACACTTCCTGTATTAGCCGGTGCTATCACCATACTTCTAACAGATCGTAATCT

CAACACGTCATTCTTTGACCCTGCTGGAGGGGGCGACCCTGTACTATACCAACACCTATT

C--------------------------------------------

>824_Embolocephalus_velutinus_T25

-----CCCTATATATAGTATTTGGTGTATGAGCCGGTATAGTTGGAACCGGCACTAGATT

ACTAATCCGCTTTGAGCTCGCGCAACCTGGATCATTCCTAGGTAGTGACCAACTCTACAA

TACGTTAGTTACTGCTCACGCCTTCCTTATAATCTTTTTCCTAGTAATACCTGTATTCAT

CGGGGGATTCGGAAATTGACTTCTCCCATTAATATTAGGGGCACCAGATATAGCATTCCC

TCGCTTAAATAATCTAAGATTTTGGCTGCTGCCACCATCTTTAATTCTTCTAGTATCTTC

GGCGGCTGTAGAAAAGGGGGCCGGAACAGGGTGAACAGTGTACCCCCCACTATCAGGTAA

CTTAGCCCATTCGGGACCATCAGTAGACTTAGCAATCTTTTCTCTTCATTTAGCTGGTGC

ATCGTCCATTTTAGGGGCAATCAACTTCATTACTACAGTAATTAACATACGAAGTAAAGG

TATGCGGCTGGAACGGGTACCACTATTTGTATGGGCTGTCCTTCTTACAGTAATTCTTCT

CCTGTTAACCCTTCCAGTACTCGCCGGAGCTATTACTATATTACTGACAGACCGCAATCT

AAATACGTCGTTCTTTGATCCAGCTGGAGGTGGTGACCCAGTACTATACCAACACCTATT

C--------------------------------------------

>825_Embolocephalus_velutinus_T25

-----CCCTATATATAGTATTTGGTGTATGAGCCGGTATAGTTGGAACCGGCACTAGATT

ACTAATCCGCTTTGAGCTCGCGCAACCTGGATCATTCCTAGGTAGTGACCAACTCTACAA

TACGTTAGTTACTGCTCACGCCTTCCTTATAATCTTTTTCCTAGTAATACCTGTATTCAT

CGGGGGATTCGGAAATTGACTTCTCCCATTAATATTAGGGGCACCAGATATAGCATTCCC

TCGCTTAAATAATCTAAGATTTTGGCTGCTGCCACCATCTTTAATTCTTCTAGTATCTTC

GGCGGCTGTAGAAAAGGGGGCCGGAACAGGGTGAACAGTGTACCCCCCACTATCAGGTAA

CTTAGCCCATTCGGGACCATCAGTAGACTTAGCAATCTTTTCTCTTCATTTAGCTGGTGC

ATCGTCCATTTTAGGGGCAATCAACTTCATTACTACAGTAATTAACATACGAAGTAAAGG

TATGCGGCTGGAACGGGTACCACTATTTGTATGGGCTGTCCTTCTTACAGTAATTCTTCT

CCTGTTAACCCTTCCAGTACTCGCCGGAGCTATTACTATATTACTGACAGACCGCAATCT

AAATACGTCGTTCTTTGATCCAGCTGGAGGTGGTGACCCAGTACTATACCAACACCTATT

C--------------------------------------------

>826_Embolocephalus_velutinus_T25

------CCCTATATATAGTATTGGTGTATGAGCCGGTATAGTTGGAACCGGCACTAGATT

ACTAATCCGCTTTGAGCTCGCGCAACCTGGATCATTCCTAGGTAGTGACCAACTCTACAA

TACGTTAGTTACTGCTCACGCCTTCCTTATAATCTTTTTCCTAGTAATACCTGTATTCAT

CGGGGGATTCGGAAATTGACTTCTCCCATTAATATTAGGGGCACCAGATATAGCATTCCC

TCGCTTAAATAATCTAAGATTTTGGCTGCTGCCACCATCTTTAATTCTTCTAGTATCTTC

GGCGGCTGTAGAAAAGGGGGCCGGAACAGGGTGAACAGTGTACCCCCCACTATCGGGTAA

CTTAGCCCATTCGGGACCATCAGTAGACTTAGCAATCTTTTCTCTTCATTTAGCTGGTGC

ATCGTCCATTTTAGGGGCAATCAACTTCATTACTACAGTAATTAACATACGAAGTAAAGG

TATGCGGCTGGAACGGGTACCACTATTTGTATGGGCTGTCCTTCTTACAGTAATTCTTCT

CCTGTTAACCCTTCCAGTACTCGCCGGAGCTATTACTATATTACTGACAGACCGCAATCT

AAATACGTCGTTCTTTGATCCAGCTGGAGGTGGTGACCCAGTACTATACCAACACCTATT

C--------------------------------------------

>812_Achaeta_sp_E7

----------------------------------------------GCAGNTATAAGACT

CCTCATTCGAATTGAACTAAGTnANCCCGGATCATTTTTAGGGAGAGANNAATTATATAA

TACCATTGTAACAGCACATGCATTCTTAATAATTTTCTTTCTTGTAATACCAGTATTTAT

TGGAGGATTTGGAAATTGACTCATTCCATTAATACTTGGAGCTCCANATATAGCTTTTCC

ACGATTAAATAATATAAGATTCTGATTACTTCCACCATCCCTCATATTACTTCTTTCTTC

TACAGCTGTAGAAAAAGGTATAGGAACAGGATGAACAGTATATCCTCCTTTAGCTAGAAA

CATTGCTCATGCAGGACCATCTGTAGATTTAGCCATTTTCTCTCTTCACTTAGCAGGAGC

TTCATCAATTCTAGGAGCAGTAAATTTCATTACTACTGTAATTAATATACGATGACAAGG

TATACGACTAGAACGAATTCCACTATTTGTATGAGCTATAATAATCACAGTAGCCCTCCT

ACTTTTAGCACTACCAGTCCTAGCTGGCGCAATTACTATGCTTCTTACTGATCGAAACCT

TAACACATCATTCTTTGATCCAGCTGGTGGAGGTGACCCTATTCTATATCAACATTTATT

T--------------------------------------------

>750_Limnodrilus_hoffmeisteri_T16

---CACTCTATACATGGTCTTCGGCCTCTGAGCAGGAATAGTGGGCACAGGAACCAGACT

ACTAATTCGATTTGAATTAGCACAACCTGGCTCATTCCTTGGCAGAGATCAATTATATAA

CACCCTAGTTACAGCTCATGGATTTTTAATAATTTTCTTTATAGTTATACCTATTTTCAT

TGGGGGCTTCGGTAACTGACTAGTCCCCCTAATACTTGGAGCTCCAGATATAGCCTTCCC

ACGACTCAATAATTTGAGATTTTGATTAATACCCCCATCTCTAATTCTATTAGTCTCCTC

GGCGGCTGTTGAGAAGGGTGCCGGAACAGGATGAACTGTATATCCACCTTTAGCTAGAAA

CCTGGCTCATTCTGGGCCCTCTGTAGACCTGGCCATTTTTTCACTTCACTTAGCAGGGGC

TGCATCAATCTTAGGTGCAATTAACTTCATTACCACAATAATTAATATACGATGAAAAGG

AATACGTTTAGAACGTATTCCCCTATTCGTGTGATCAGTTATTATTACGGTTATTTTACT

CCTTCTAACCTTACCAGTTCTAGCAGGGGCAATCACTATACTTCTAACAGACCGAAATCT

AAACACGTCATTCTTCGACCCTGCGGGTGGTGGCGACCCGGTCCTATATCAACACTTATT

C--------------------------------------------

>752_Potamothrix_moldaviensis_T30

---ACTTCTTTATATATTATATGGACTATGAACAGGTATGGTGGGAACAGGAACTAGAAT

TTTAATTCGTCTGGAACTTGCTCAGCCTGGGTCATTCCTAGGTAGTGATCAGTTATATAA

CACCCTCGTAACTGCTCACGCCTTTCTGATAATCTTTTTTCTAGTAATACCTGTATATAT

TGGAGCTTTCGGGAACTTCCTAATCCCACTAATACTAGGGGCTCCTGATATAGCTTTCCC

ACGGTTAAACAATTTAAGATTTTGACTTATACCTTCATCTGTAATCCTCCTTGTGGCATC

TGCAGCTGTTGAAAAGGGAGCAGGAACAGGTTGAACAGTGTACCCTCCCCTAGCAAGAAA

CCTTGCCCATTCCGGGCCTTCGGTAGACCTGGCCATTTTCTCTCTCCATCTAGCAGGAAT

TGCTTCCATTTTAGGGGCAATTAACTTCATTACAACAATAATTAATATACGTTGAAAAGG

GATACGATTAGAGCGAATCCCCTTATTCGTTTGAGCGACAATCATCACAGTTATTTTACT

ACTATTAACTCTACCCGTATTAGCGGGCGCTATTACCATACTATTAACAGACCGAAATCT

AAATACCTCATTCTTCGATCCTGCTGGGGGAGGAGACCCTGTCTTATATCAACACCTATT

T--------------------------------------------

>753_Potamothrix_moldaviensis_T30

---ACTTCTTTATATATTATATGGACTATGAACAGGTATGGTGGGAACAGGAACTAGAAT

TTTAATTCGTCTGGAACTTGCTCAGCCTGGGTCATTCCTAGGTAGTGATCAGTTATATAA

CACCCTCGTAACTGCTCACGCCTTTCTGATAATCTTTTTTCTAGTAATACCTGTATATAT

TGGAGCTTTCGGGAACTTCCTAATCCCACTAATACTAGGGGCTCCTGATATAGCTTTCCC

ACGGTTAAACAATTTAAGATTTTGACTTATACCTTCATCTGTAATCCTCCTTGTGGCATC

TGCAGCTGTTGAAAAGGGAGCAGGAACAGGTTGAACAGTGTACCCTCCCCTAGCAAGAAA

CCTTGCCCATTCCGGGCCTTCGGTAGACCTGGCCATTTTCTCTCTCCATCTAGCAGGAAT

TGCTTCCATTTTAGGGGCAATTAACTTCATTACAACAATAATTAATATACGTTGAAAAGG

GATACGATTAGAGCGAATCCCCTTATTCGTTTGAGCGACAATCATCACAGTTATTTTACT

ACTATTAACTCTACCCGTATTAGCGGGCGCTATTACCATACTATTAACAGACCGAAATCT

AAATACCTCATTCTTCGATCCTGCTGGGGGAGGAGACCCTGTCTTATATCAACACCTATT

T--------------------------------------------

>754_Limnodrilus_hoffmeisteri_T17

---CACCCTATACATAATCTTTGGCCTTTGAGCAGGAATAGTAGGCACAGGAACTAGACT

TTTAATTCGATTTGAGCTAGCACAACCCGGCTCATTTCTCGGTAGAGACCAATTATATAA

CACTCTAGTCACGGCTCACGGATTTTTAATAATTTTCTTTATAGTAATACCTATCTTTAT

TGGGGGATTTGGAAATTGATTAGTGCCTTTAATACTTGGAGCACCTGATATGGCATTCCC

ACGGCTTAATAACCTAAGATTCTGACTAATGCCCCCATCACTAATTCTACTAGTCTCATC

AGCTGCAGTTGAAAAAGGCGCAGGGACAGGGTGAACTGTATACCCCCCCTTAGCAAGAAA

TCTAGCTCATTCCGGGCCTTCTGTAGATCTGGCAATTTTTTCACTACACTTAGCAGGAGC

CGCATCAATCCTGGGAGCAATTAACTTCATCACAACAATAATCAATATACGATGAAAGGG

AATACGCTTAGAGCGTATTCCTTTATTTGTGTGATCCGTAATCATCACAGTTATTTTACT

TCTTCTTACCCTTCCAGTTCTTGCCGGAGCTATCACCATACTTTTAACAGATCGAAACCT

AAACACCTCATTCTTTGATCCGGCAGGTGGAGGCGATCCAGTTTTATACCAACATCTATT

T--------------------------------------------

>755_Limnodrilus_hoffmeisteri_T17

---CACCCTATACATAATCTTTGGCCTTTGAGCAGGAATAGTAGGCACAGGAACTAGACT

TTTAATTCGATTTGAGCTAGCACAACCCGGCTCATTTCTCGGTAGAGACCAATTATATAA

CACTCTAGTCACGGCTCACGGATTTTTAATAATTTTCTTTATAGTAATACCTATCTTTAT

TGGGGGATTTGGAAATTGATTAGTGCCTTTAATACTTGGAGCACCTGATATGGCATTCCC

ACGGCTTAATAACCTAAGATTCTGACTAATGCCCCCATCACTAATTCTACTAGTCTCATC

AGCTGCAGTTGAAAAAGGCGCAGGGACAGGGTGAACTGTATACCCCCCCTTAGCAAGAAA

TCTAGCTCATTCCGGGCCTTCTGTAGATCTGGCAATTTTTTCACTACACTTAGCAGGAGC

CGCATCAATCCTGGGAGCAATTAACTTCATCACAACAATAATCAATATACGATGAAAGGG

AATACGCTTAGAGCGTATTCCTTTATTTGTGTGATCCGTAATCATCACAGTTATTTTACT

TCTTCTTACCCTTCCAGTTCTTGCCGGAGCTATCACCATACTTTTAACAGATCGAAACCT

AAACACCTCATTCTTTGATCCGGCAGGTGGAGGCGATCCAGTTTTATACCAACATCTATT

T--------------------------------------------

>757_Limnodrilus_hoffmeisteri_T18

---CACCTTATATATAATCTTCGGCCTATGAGCCGGAATAGTGGGCACAGGAACAAGCCT

GCTAATTCGATTCGAATTAGCACAACCTGGTTCATTCCTCGGAAGAGATCAACTCTATAA

TACCTTAGTGACAGCCCACGGTTTCCTTATAATCTTCTTCATGGTGATACCAATTTTTAT

TGGTGGCTTCGGAAATTGACTAGTCCCCCTAATACTAGGAGCTCCTGACATGGCCTTTCC

ACGACTAAATAACCTAAGATTTTGACTAATACCTCCATCACTCATTCTATTAGTTTCATC

AGCCGCAGTCGAAAAGGGAGCGGGGACAGGGTGAACTGTATACCCCCCTCTAGCCAGAAA

CCTAGCGCATTCTGGGCCATCTGTGGATCTAGCAATCTTCTCTCTTCACTTAGCCGGGGC

TGCATCAATTCTAGGTGCTATTAACTTCATTACCACAATAATTAATATACGATGAAAAGG

AATGCGCCTAGAGCGCATTCCTCTATTTGTATGATCAGTAATCATTACAGTTGTCCTCCT

TCTTCTTACATTACCGGTTTTAGCGGGGGCAATCACCATACTTTTAACAGACCGAAACTT

AAACACCTCATTCTTCGATCCTGCGGAGGGGGGGNACCCGGTACTATATCAACACTTATT

T--------------------------------------------

>758_Limnodrilus_hoffmeisteri_T17

---CACCCTATACATAATCTTTGGCCTTTGAGCAGGAATAGTAGGCACAGGAACTAGACT

TTTAATTCGATTTGAGCTAGCACAACCCGGCTCATTTCTCGGTAGAGACCAATTATATAA

CACTCTAGTCACGGCTCACGGATTTTTAATAATTTTCTTTATAGTAATACCTATCTTTAT

TGGGGGATTTGGAAATTGATTAGTGCCTTTAATACTTGGAGCACCTGATATGGCATTCCC

ACGGCTTAATAACCTAAGATTCTGACTAATGCCCCCATCACTAATTCTACTAGTCTCATC

AGCTGCAGTTGAAAAAGGCGCAGGGACAGGGTGAACTGTATACCCCCCCTTAGCAAGAAA

TCTAGCTCATTCCGGGCCTTCTGTAGATCTGGCAATTTTTTCACTACACTTAGCAGGAGC

CGCATCAATCCTGGGAGCAATTAACTTCATCACAACAATAATCAATATACGATGAAAGGG

AATACGCTTAGAGCGTATTCCTTTATTTGTGTGATCCGTAATCATCACAGTTATTTTACT

TCTTCTTACCCTTCCAGTTCTTGCCGGAGCTATCACCATACTTTTAACAGATCGAAACCT

AAACACCTCATTCTTTGATCCGGCAGGTGGAGGCGATCCAGTTTTATACCAACATCTATT

---------------------------------------------

>759_Limnodrilus_hoffmeisteri_T17

---CACCCTATACATAATCTTTGGCCTTTGAGCAGGAATAGTAGGCACAGGAACTAGACT

TTTAATTCGATTTGAGCTAGCACAACCCGGCTCATTTCTCGGTAGAGACCAATTATATAA

CACTCTAGTCACGGCTCACGGATTTTTAATAATTTTCTTTATAGTAATACCTATCTTTAT

TGGGGGATTTGGAAATTGATTAGTGCCTTTAATACTTGGAGCACCTGATATGGCATTCCC

ACGGCTTAATAACCTAAGATTCTGACTAATGCCCCCATCACTAATTCTACTAGTCTCATC

AGCTGCAGTTGAAAAAGGCGCAGGGACAGGGTGAACTGTATACCCCCCCTTAGCAAGAAA

TCTAGCTCATTCCGGGCCTTCTGTAGATCTGGCAATTTTTTCACTACACTTAGCAGGAGC

CGCATCAATCCTGGGAGCAATTAACTTCATCACAACAATAATCAATATACGATGAAAGGG

AATACGCTTAGAGCGTATTCCTTTATTTGTGTGATCCGTAATCATCACAGTTATTTTACT

TCTTCTTACCCTTCCAGTTCTTGCCGGAGCTATCACCATACTTTTAACAGATCGAAACCT

AAACACCTCATTCTTTGATCCGGCANGTGGAGGCGATCCAGTTTTATACCAACATCTATT

---------------------------------------------

>760_Tubifex_tubifex_T12

---AACCCTATACATAATTTTCGGAATCTGAGCTGGTATGGTTGGAACTGGGACTAGGCT

ATTAATTCGCCTTGAACTTGCCCAACCTGGGTCCTTTTTAGGAAGAGATCAACTATATAA

TACCCTGGTAACGGCTCACGCATTTTTAATAATCTTCTTCATAGTAATACCAATCTATAT

TGGGGGCTTCGGCAACTGACTAGTACCATTAATACTGGGGGCTCCGGACATGGCCTTCCC

ACGATTAAACAACTTAAGATTCTGACTCCTACCCCCATCATTAATCCTTTTAGTATCCTC

TGCCGCCGTTGAAAAAGGCGCCGGAACTGGTTGAACTGTTTACCCCCCTCTAGCTAGAAA

TCTAGCTCACTCTGGGCCCTCAGTAGATTTAGCAATCTTTTCTCTACACTTAGCTGGTGT

TGCATCAATCCTAGGTGCAATCAACTTCATTACTACAATAATCAACATACGATGAAAAGG

GATGCGACTAGAGCGTATTCCTTTATTTGTATGATCAGTAATTATCACTGTAATTCTACT

CCTACTCACACTTCCAGTTTTAGCTGGAGCTATTACAATACTTCTAACAGACCGGAATCT

AAATACATCATTTTTCGATCCTGCTGGAGGGGGTGACCCTGTACTATATCAACACCTATT

C--------------------------------------------

>763_Limnodrilus_hoffmeisteri_T18

---CACCTTATATATAATCTTCGGCCTATGAGCCGGAATAGTGGGCACAGGAACAAGCCT

GCTAATTCGATTCGAATTAGCACAACCTGGTTCATTCCTCGGTAGAGATCAACTCTATAA

TACCTTAGTGACAGCCCACGGTTTCCTTATAATCTTCTTCATGGTAATACCAATTTTTAT

TGGTGGCTTCGGAAATTGACTAGTCCCCCTAATGCTAGGAGCTCCTGACATGGCCTTTCC

ACGACTAAATAACCTAAGATTTTGACTAATACCTCCATCACTCATTCTATTAGTTTCATC

AGCCGCAGTCGAAAAGGGAGCGGGGACAGGGTGAACTGTATACCCCCCTCTAGCCAGAAA

CCTAGCGCACTCTGGGCCATCTGTGGATCTAGCAATCTTCTCTCTTCACTTAGCCGGGGC

TGCATCAATTCTAGGTGCCATTAACTTCATTACCACAATAATTAATATACGATGAAAAGG

AATGCGCCTAGAGCGCATTCCTCTATTTGTATGATCAGTAATCATTACAGTTATCCTCCT

TCTTCTTACATTACCGGTTTTAGCGGGGGCAATCACCATACTTTTAACAGACCGAAACTT

AAACACATCATTCTTCGATCCTGCGGGAGGGGGGGACCCGGTCCTATATCAACACTTATT

---------------------------------------------

>764_Limnodrilus_hoffmeisteri_T18

---CACCTTATATATAATCTTCGGCCTATGAGCCGGAATAGTGGGCACAGGAACAAGCCT

GCTAATTCGATTCGAATTAGCACAACCTGGTTCATTCCTCGGTAGAGATCAACTCTATAA

TACCTTAGTGACAGCCCACGGTTTCCTTATAATCTTCTTCATGGTAATACCAATTTTTAT

TGGTGGCTTCGGAAATTGACTAGTCCCCCTAATGCTAGGAGCTCCTGACATGGCCTTTCC

ACGACTAAATAACCTAAGATTTTGACTAATACCTCCATCACTCATTCTATTAGTTTCATC

AGCCGCAGTCGAAAAGGGAGCGGGGACAGGGTGAACTGTATACCCCCCTCTAGCCAGAAA

CCTAGCGCACTCTGGGCCATCTGTGGATCTAGCAATCTTCTCTCTTCACTTAGCCGGGGC

TGCATCAATTCTAGGTGCCATTAACTTCATTACCACAATAATTAATATACGATGAAAAGG

AATGCGCCTAGAGCGCATTCCTCTATTTGTATGATCAGTAATCATTACAGTTATCCTCCT

TCTTCTTACATTACCGGTTTTAGCGGGGGCAATCACCATACTTTTAACAGACCGAAACTT

AAACACATCATTCTTCGATCCTGCGGGAGGGGGGGACCCGGTACTATCTCAACACTTATT

---------------------------------------------

>765_Limnodrilus_claparedianus_T22

---CACTCTCTACATAGTTTTCGGCCTTTGAGCCGGAATAGTCGGTACTGGAACAAGCCT

ACTAATTCGATTTGAGTTAGCTCAACCCGGATCATTCTTAGGCAGAGACCAGTTATATAA

CACTTTAGTGACAGCCCACGGATTCTTAATAATTTTCTTTATAGTAATGCCAATCTTTAT

TGGTGGATTTGGAAATTGATTAGTTCCTTTAATACTTGGGGCACCAGATATAGCATTCCC

ACGATTAAATAATCTCAGCTTTTGACTAATGCCGCCATCATTAATTCTATTAGTGTCATC

TGCTGCTGTAGAAAAAGGTGCCGGTACAGGTTGAACTGTATATCCACCCCTGGCAAGAAA

TTTAGCACATTCAGGACCATCTGTAGATTTAGCAATTTTCTCCCTTCATCTTGCTGGTGC

AGCTTCAATTCTAGGGGCAATTAACTTTATTACAACAATAATTAACATGCGATGAAAAGG

AATACGCCTCGAACGAATTCCATTATTCGTATGATCTGTAATTATTACTGTTATTCTACT

ACTTCTAACCCTTCCAGTACTTGCGGGAGCTATCACGATGCTGTTAACAGACCGAAATCT

AAATACATCGTTCTTCGACCCTGCGGGGGGGGGAGACCCTGTACTTTATCAACACTTATT

C--------------------------------------------

>766_Limnodrilus_hoffmeisteri_T18

---CACCTTATATATAATCTTCGGCCTATGAGCCGGAATAGTGGGCACAGGAACAAGCCT

GCTAATTCGATTCGAATTAGCACAACCTGGTTCATTCCTCGGTAGAGATCAACTCTATAA

TACCTTAGTGACAGCCCACGGTTTCCTTATAATCTTCTTCATGGTAATACCAATTTTTAT

TGGTGGCTTCGGAAATTGACTAGTCCCCCTAATGCTAGGAGCTCCTGACATGGCCTTTCC

ACGACTAAATAACCTAAGATTTTGACTAATACCTCCATCACTCATTCTATTAGTTTCATC

AGCCGCAGTCGAAAAGGGAGCGGGGACAGGGTGAACTGTATACCCCCCTCTAGCCAGAAA

CCTAGCGCACTCTGGGCCATCTGTGGATCTAGCAATCTTCTCTCTTCACTTAGCCGGGGC

TGCATCAATTCTAGGTGCCATTAACTTCATTACCACAATAATTAATATACGATGAAAAGG

AATGCGCCTAGAGCGCATTCCTCTATTTGTATGATCAGTAATCATTACAGTTATCCTCCT

TCTTCTTACATTACCGGTTTTAGCGGGGGCAATCACCATACTTTTAACAGACCGAAACTT

AAACACATCATTCTTCGATCCTGCGGGAGGGGGGGACCCGGTACTATATCAACACTTATT

T--------------------------------------------

>767_Limnodrilus_claparedianus_T22

---CACTCTCTACATAGTTTTCGGCCTTTGAGCCGGAATAGTCGGTACTGGAACAAGCCT

ACTAATTCGATTTGAGTTAGCTCAACCCGGATCATTCTTAGGCAGAGACCAGTTATATAA

CACTTTAGTGACAGCCCACGGATTCTTAATAATTTTCTTTATAGTAATGCCAATCTTTAT

TGGTGGATTTGGAAATTGATTAGTTCCTTTAATACTTGGGGCACCAGATATAGCATTCCC

ACGATTAAATAATCTCAGCTTTTGACTAATGCCGCCATCATTAATTCTATTAGTGTCATC

TGCTGCTGTAGAAAAAGGTGCCGGTACAGGTTGAACTGTATATCCACCCCTGGCAAGAAA

TTTAGCACATTCAGGACCATCTGTAGATTTAGCAATTTTCTCCCTTCATCTTGCTGGTGC

AGCTTCAATTCTAGGGGCAATTAACTTTATTACAACAATAATTAACATGCGATGAAAAGG

AATACGCCTCGAACGAATTCCATTATTCGTATGATCTGTAATTATTACTGTTATTCTACT

ACTTCTAACCCTTCCAGTACTTGCGGGAGCTATCACGATGCTGTTAACAGACCGAAATCT

AAATACATCGTTCTTCGACCCTGCGGGGGGGGGAGACCCTGTACTTTATCAACACTTATT

C--------------------------------------------

>768_Limnodrilus_hoffmeisteri_T18

---CACCTTATATATAATCTTCGGCCTATGAGCCGGAATAGTGGGCACAGGAACAAGCCT

GCTAATTCGATTCGAATTAGCACAACCTGGTTCATTCCTCGGAAGAGATCAACTCTATAA

TACCTTAGTGACAGCCCACGGTTTCCTTATAATCTTCTTCATGGTGATACCAATTTTTAT

CGGTGGCTTCGGAAATTGACTAGTCCCCCTAATACTAGGAGCTCCTGACATGGCCTTTCC

ACGACTAAATAACCTAAGATTTTGACTAATACCTCCATCACTCATTCTATTAGTTTCATC

AGCCGCAGTCGAAAAGGGAGCGGGGACAGGGTGAACTGTATACCCCCCTCTAGCCAGAAA

CCTAGCGCATTCTGGGCCATCTGTGGATCTAGCAATCTTCTCTCTTCACTTAGCCGGGGC

TGCATCAATTCTAGGTGCTATTAACTTCATTACCACAATAATTAATATACGATGAAAAGG

AATGCGCCTAGAGCGCATTCCTCTATTTGTATGATCAGTAATCATTACAGTTGTCCTCCT

TCTTCTTACATTACCGGTTTTAGCGGGGGCAATCACCATACTTTTAACAGACCGAAACTT

AAACACCTCATTCTTCGATCCTGCGGGAGGGGGGGACCCGGTACTATATCAACACTTATT

T--------------------------------------------

>769_Limnodrilus_hoffmeisteri_T18

---CACCTTATATATAATCTTCGGCCTATGAGCCGGAATAGTGGGCACAGGAACAAGCCT

GCTAATTCGATTCGAATTAGCACAACCTGGTTCATTCCTCGGAAGAGATCAACTCTATAA

TACCTTAGTGACAGCCCACGGTTTCCTTATAATCTTCTTCATGGTGATACCAATTTTTAT

CGGTGGCTTCGGAAATTGACTAGTCCCCCTAATACTAGGAGCTCCTGACATGGCCTTTCC

ACGACTAAATAACCTAAGATTTTGACTAATACCTCCATCACTCATTCTATTAGTTTCATC

AGCCGCAGTCGAAAAGGGAGCGGGGACAGGGTGAACTGTATACCCCCCTCTAGCCAGAAA

CCTAGCGCATTCTGGGCCATCTGTGGATCTAGCAATCTTCTCTCTTCACTTAGCCGGGGC

TGCATCAATTCTAGGTGCTATTAACTTCATTACCACAATAATTAATATACGATGAAAAGG

AATGCGCCTAGAGCGCATTCCTCTATTTGTATGATCAGTAATCATTACAGTTGTCCTCCT

TCTTCTTACATTACCGGTTTTAGCGGGGGCAATCACCATACTTTTAACAGACCGAAACTT

AAACACCTCATTCTTCGATCCTGCGGGAGGGGGGGACCCGGTACTATATCAACACTTATT

T--------------------------------------------

>770_Limnodrilus_hoffmeisteri_T17

---CACCCTATACATAATCTTTGGCCTTTGAGCAGGAATAGTAGGCACAGGAACTAGACT

TTTAATTCGATTTGAGCTAGCACAACCCGGCTCATTTCTCGGTAGAGACCAATTATATAA

CACTCTAGTCACGGCTCACGGATTTTTAATAATTTTCTTTATAGTAATACCTATCTTTAT

TGGGGGATTTGGAAATTGATTAGTGCCTTTAATACTTGGAGCACCTGATATGGCATTCCC

ACGGCTTAATAACCTAAGATTCTGACTAATGCCCCCATCACTAATTCTACTAGTCTCATC

AGCTGCAGTTGAAAAAGGCGCAGGGACAGGGTGAACTGTATACCCCCCCTTAGCAAGAAA

TCTAGCTCATTCCGGGCCTTCTGTAGATCTGGCAATTTTTTCACTACACTTAGCAGGAGC

CGCATCAATCCTGGGAGCAATTAACTTCATCACAACAATAATCAATATACGATGAAAGGG

AATACGCTTAGAGCGTATTCCTTTATTTGTGTGATCCGTAATCATCACAGTTATTTTACT

TCTTCTTACCCTTCCAGTTCTTGCCGGAGCTATCACCATACTTTTAACAGATCGAAACCT

AAACACCTCATTCTTTGATCCGGCAGGTGGAGGCGATCCAGTTTTATACCAACATCTATT

T--------------------------------------------

>772_Limnodrilus_claparedianus_T22

---CACTCTCTACATAGTTTTCGGCCTTTGAGCCGGAATAGTCGGTACTGGAACAAGCCT

ACTAATTCGATTTGAGTTAGCTCAACCCGGATCATTCTTAGGCAGAGACCAGTTATATAA

CACTTTAGTGACAGCCCACGGATTCTTAATAATTTTCTTTATAGTAATGCCAATCTTTAT

TGGTGGATTTGGAAATTGATTAGTTCCTTTAATACTTGGGGCACCAGATATAGCATTCCC

ACGATTAAATAATCTCAGCTTTTGACTAATGCCGCCATCATTAATTCTATTAGTGTCATC

TGCTGCTGTAGAAAAAGGTGCCGGTACAGGTTGAACTGTATATCCACCCCTGGCAAGAAA

TTTAGCACATTCAGGACCATCTGTAGATTTAGCAATTTTCTCCCTTCATCTTGCTGGTGC

AGCTTCAATTCTAGGGGCAATTAACTTTATTACAACAATAATTAACATGCGATGAAAAGG

AATACGCCTCGAACGAATTCCATTATTCGTATGATCTGTAATTATTACTGTTATTCTACT

ACTTCTAACCCTTCCAGTACTTGCGGGAGCTATCACGATGCTGTTAACAGACCGAAATCT

AAATACATCGTTCTTCGACCCTGCGGGGGGGGGAGACCCTGTACTTTATCAACACTTATT

C--------------------------------------------

>773_Tubifex_tubifex_T11

---AACTCTATATATCATTTTCGGGATTTGAGCTGGGATGGTCGGGACAGGAACTAGACT

CTTAATTCGTCTAGAACTGGCTCAACCTGGCTCATTCCTTGGAAGGGATCAGCTATACAA

CACCCTAGTTACAGCACATGCATTTCTGATAATTTTCTTCATGGTAATACCTATCTATAT

TGGGGGTTTCGGAAATTGGTTAGTACCCTTAATGCTGGGAGCTCCTGACATAGCATTCCC

CCGATTAAATAATTTAAGATTTTGGTTATTACCCCCTTCCTTAATCCTCCTTGTATCCTC

CGCGGCCGTAGAAAAGGGGGCTGGAACAGGGTGAACAGTATATCCGCCCCTAGCTAGAAA

TCTGGCTCATTCTGGGCCTTCTGTAGACTTAGCTATTTTCTCTCTACATTTAGCTGGGGT

GGCATCGATTCTAGGAGCTATTAACTTTATTACTACAATAATTAACATGCGATGAAAAGG

GATACGCCTTGAACGAATTCCCCTATTTGTATGAGCTGTAATTCTAACCGTAATCTTACT

TTTACTAACATTACCAGTTTTAGCTGGTGCCATTACCATGCTACTAACAGATCGAAATCT

AAACACATCCTTCTTTGACCCTGCTGGGGGTGGAGATCCTGTGCTCTATCAACACTTATT

C--------------------------------------------

>775_Tubifex_tubifex_T11

---AACTCTATATATCATTTTCGGGATTTGAGCTGGGATGGTCGGGACAGGAACTAGACT

CTTAATTCGTCTAGAACTGGCTCAACCTGGCTCATTCCTTGGAAGGGATCAGCTATACAA

CACCCTAGTTACAGCACATGCATTTCTGATAATTTTCTTCATGGTAATACCTATCTATAT

TGGGGGTTTCGGAAATTGGTTAGTACCCTTAATGCTGGGAGCTCCTGACATAGCATTCCC

CCGATTAAATAATTTAAGATTTTGGTTATTACCCCCTTCCTTAATCCTCCTTGTATCCTC

CGCGGCCGTAGAAAAGGGGGCTGGAACAGGGTGAACAGTATATCCGCCCCTAGCTAGAAA

TCTGGCTCATTCTGGGCCTTCTGTAGACTTAGCTATTTTCTCTCTACATTTAGCTGGGGT

GGCATCGATTCTAGGAGCTATTAACTTTATTACTACAATAATTAACATGCGATGAAAAGG

GATACGCCTTGAACGAATTCCCCTATTTGTATGAGCTGTAATTCTAACCGTAATCTTACT

TTTACTAACATTACCAGTTTTAGCTGGTGCCATTACCATGCTACTAACAGATCGAAATCT

AAACACATCCTTCTTTGACCCTGCTGGGGGTGGAGATCCTGTGCTCTATCAACACTTATT

C--------------------------------------------

>777_Limnodrilus_hoffmeisteri_T17

---CACCCTATACATAATCTTTGGCCTTTGAGCAGGAATAGTAGGCACAGGAACTAGACT

TTTAATTCGATTTGAGCTAGCACAACCCGGCTCATTTCTCGGTAGAGACCAATTATATAA

CACTCTAGTCACGGCTCACGGATTTTTAATAATTTTCTTTATAGTAATACCTATCTTTAT

TGGGGGATTTGGAAATTGATTAGTGCCTTTAATACTTGGAGCACCTGATATGGCATTCCC

ACGGCTTAATAACCTAAGATTCTGACTAATGCCCCCATCACTAATTCTACTAGTCTCATC

AGCTGCAGTTGAAAAAGGCGCAGGGACAGGGTGAACTGTATACCCCCCCTTAGCAAGAAA

TCTAGCTCATTCCGGGCCTTCTGTAGATCTGGCAATTTTTTCACTACACTTAGCAGGAGC

CGCATCAATCCTGGGAGCAATTAACTTCATCACAACAATAATCAATATACGATGAAAGGG

AATACGCTTAGAGCGTATTCCTTTATTTGTGTGATCCGTAATCATCACAGTTATTTTACT

TCTTCTTACCCTTCCAGTTCTTGCCGGAGCTATCACCATACTTTTAACAGATCGAAACCT

AAACACCTCATTCTTTGATCCGGCAGGTGGAGGCGATCCAGTTTTATACCAACATCTATT

T--------------------------------------------

>778_Aulodrilus_pluriseta_T4

---AACTCTATACTTCATTTTCGGAATCTGAGCCGGTATAGTTGGTACTGGTACCAGACT

ATTAATTCGCTTAGAACTAGCACAACCTGGATCCTTCCTAGGAAGAGATCAACTATATAA

TACATTAGTAACAGCCCACGCATTTCTAATAATCTTCTTCCTAGTTATACCTGTATTTAT

CGGGGGATTCGGAAATTGATTAATCCCATTAATACTAGGTGCTCCCGATATAGCATTTCC

ACGACTCAATAACCTAAGATTTTGACTAATACCACCTTCACTAATTTTACTAGTCTCATC

TGCAGCTGTAGAAAAAGGTGCCGGAACAGGTTGAACAGTATACCCACCACTTGCTGGCAA

TCTAGCCCACTCAGGACCTTCTGTAGACCTCGCAATCTTCTCTCTACACTTAGCAGGAGT

AGCATCAATTTTAGGTGCTATTAACTTCATTACCACCATAATTAACATGCGATGAAAAGG

GATACGCCTAGAACGAATTCCTCTATTTGTATGAGCCGTAATTCTTACCGTTGTACTCCT

TCTATTAACCCTACCAGTTCTTGCAGGAGCAATCACTATACTTTTAACTGACCGCAATCT

AAACACCTCCTTCTTCGATCCGGCTGGAGGTGGTGACCCTGTTCTATACCAACATCTATT

C--------------------------------------------

>779_Aulodrilus_pluriseta_T4

---AACTCTATACTTCATTTTCGGAATCTGAGCCGGTATAGTTGGTACTGGTACCAGACT

ATTAATTCGCTTAGAACTAGCACAACCTGGATCCTTCCTAGGAAGAGATCAACTATATAA

TACATTAGTAACAGCCCACGCATTTCTAATAATCTTCTTCCTAGTTATACCTGTATTTAT

CGGGGGATTCGGAAATTGATTAATCCCATTAATACTAGGTGCTCCCGATATAGCATTTCC

ACGACTCAATAACCTAAGATTTTGACTAATACCACCTTCACTAATTTTACTAGTCTCATC

TGCAGCTGTAGAAAAAGGTGCCGGAACAGGTTGAACAGTATACCCACCACTTGCTGGCAA

TCTAGCCCACTCAGGACCTTCTGTAGACCTCGCAATCTTCTCTCTACACTTAGCAGGAGT

AGCATCAATTTTAGGTGCTATTAACTTCATTACCACCATAATTAACATGCGATGAAAAGG

GATACGCCTAGAACGAATTCCTCTATTTGTATGAGCCGTAATTCTTACCGTTGTACTCCT

TCTATTAACCCTACCAGTTCTTGCAGGAGCAATCACTATACTTTTAACTGACCGCAATCT

AAACACCTCCTTCTTCGATCCGGCTGGAGGTGGTGACCCTGTTCTATACCAACATCTATC

---------------------------------------------

>794_Embolocephalus_velutinus_T25

---AACCCTATATATAGTATTTGGTGTATGAGCCGGTATAGTTGGAACCGGCACTAGATT

ACTAATCCGCTTTGAGCTCGCGCAACCTGGATCATTCCTAGGTAGTGACCAACTCTACAA

TACGTTAGTTACTGCTCACGCCTTCCTTATAATCTTTTTCCTAGTAATACCTGTATTCAT

CGGGGGATTCGGAAATTGACTTCTCCCATTAATATTAGGGGCACCAGATATAGCATTCCC

TCGCTTAAATAATCTAAGATTTTGGCTGCTGCCACCATCTTTAATTCTTCTAGTATCTTC

GGCGGCTGTAGAAAAGGGGGCCGGAACAGGGTGAACAGTGTACCCCCCACTATCAGGTAA

CTTAGCCCATTCGGGACCATCAGTAGACTTAGCAATCTTTTCTCTTCATTTAGCTGGTGC

ATCGTCCATTTTAGGGGCAATCAACTTCATTACTACAGTAATTAACATACGAAGTAAAGG

TATGCGGCTGGAACGGGTACCACTATTTGTATGGGCTGTCCTTCTTACAGTAATTCTTCT

CCTGTTAACCCTTCCAGTACTCGCCGGAGCTATTACTATATTACTGACAGACCGCAATCT

AAATACGTCGTTCTTTGATCCAGCTGGAGGTGGTGACCCAGTACTATACCAACACCTATT

C--------------------------------------------

>795_Embolocephalus_velutinus_T25

---AACCCTATATATAGTATTTGGTGTATGAGCCGGTATAGTTGGAACCGGCACTAGATT

ACTAATCCGCTTTGAGCTCGCGCAACCTGGATCATTCCTAGGTAGTGACCAACTCTACAA

TACGTTAGTTACTGCTCACGCCTTCCTTATAATCTTTTTCCTAGTAATACCTGTATTCAT

CGGGGGATTCGGAAATTGACTTCTCCCATTAATATTAGGGGCACCAGATATAGCATTCCC

TCGCTTAAATAATCTAAGATTTTGGCTGCTGCCACCATCTTTAATTCTTCTAGTATCTTC

GGCGGCTGTAGAAAAGGGGGCCGGAACAGGGTGAACAGTGTACCCCCCACTATCAGGTAA

CTTAGCCCATTCGGGACCATCAGTAGACTTAGCAATCTTTTCTCTTCATTTAGCTGGTGC

ATCGTCCATTTTAGGGGCAATCAACTTCATTACTACAGTAATTAACATACGAAGTAAAGG

TATGCGGCTGGAACGGGTACCACTATTTGTATGGGCTGTCCTTCTTACAGTAATTCTTCT

CCTGTTAACCCTTCCAGTACTCGCCGGAGCTATTACTATATTACTGACAGACCGCAATCT

AAATACGTCGTTCTTTGATCCAGCTGGAGGTGGTGACCCAGTACTATACCAACACCTATT

C--------------------------------------------

>749_Embolocephalus_velutinus_T25

---AACTCTATATATAGTATTTGGTGTGTGAGCCGGTATAGTTGGAACCGGCACTAGATT

ACTAATCCGCTTTGAGCTTGCGCAACCTGGATCATTCCTAGGCAGTGACCAACTCTACAA

TACATTAGTTACTGCTCACGCCTTCCTAATAATCTTTTTCCTAGTAATACCTGTATTCAT

CGGAGGATTCGGAAATTGGCTTCTCCCGCTAATATTAGGGGCACCAGATATAGCATTCCC

CCGCTTAAATAACCTAAGATTTTGGCTGCTGCCACCATCTTTAATTCTTCTAGTGTCTTC

GGCGGCCGTAGAAAAAGGAGCCGGAACAGGATGAACAGTATATCCCCCGCTTTCCGGTAA

CTTAGCCCATTCGGGGCCATCAGTAGACTTAGCAATCTTTTCCCTTCACTTAGCCGGTGC

GTCGTCCATTTTAGGGGCAATCAACTTCATTACTACAGTAATTAACATACGAAGTAAAGG

CATACGACTAGAGCGGGTACCACTATTTGTATGAGCTGTCCTTCTTACAGTAATTCTTCT

CCTGTTAACCCTCCCAGTACTCGCCGGAGCTATTACTATATTACTGACAGACCGCAATCT

AAATACGTCATTCTTTGACCCGGCTGGTGGTGGCGACCCAGTACTATACCAACACCTATT

C--------------------------------------------

>827_Stylodrilus_heringianus_LL3

---AACCTTATATTTCATCTTAGGAGTTTGAGCTGGAATAGTAGGAGCAGGAATAAGACT

ATTAATTCGAGTTGAATTAACACAACCAGGATCATTTTTAGGAAGTGATCAATTATATAA

TACTATTGTTACGGCTCACGCCTTTATTATAATTTTCTTTATAGTTATACCTATATTTAT

TGGTGGCTTTGGTAACTGAATATTACCATTAATATTAGGAGCACCAGATATGGCATTCCC

ACGACTTAACAACCTAAGATTTTGGTTACTCCCGCCTTCTTTAACTTTATTAGTAGCATC

GGCTGCAGTTGAAAAGGGAGCCGGCACAGGATGAACAGTTTATCCACCTCTATCAAGAAA

TTTAGCTCATGCAGGTCCATCAGTAGACCTAGCAATCTTTTCACTACATCTAGCTGGGGC

ATCCTCCATTCTAGGAGCTATTAACTTTATTACTACGGTAATTAATATACGATGAAATGG

ACTCCGACTTGAACGAGTTCCATTATTTGTATGAGCAGTAACAATTACAGTTGTTCTATT

ACTATTATCATTACCAGTACTAGCTGGGGCAATCACTATACTTCTTACAGATCGAAACTT

AAATACAACATTTTTCGATCCGGCTGGTGGTGGAGACCCAGTATTATATCAACATTTATT

T--------------------------------------------

>830_Lumbriculidae_sp_LL3

---AACCTTATATTTCATCTTAGGAGTTTGAGCTGGAATAGTAGGAGCAGGAATAAGACT

ATTAATTCGAGTTGAATTAACACAACCAGGATCATTTTTAGGAAGTGATCAATTATATAA

TACTATTGTTACGGCCCACGCCTTTATTATAATTTTCTTTATAGTTATACCTATATTTAT

TGGTGGCTTTGGTAACTGAATATTACCATTAATATTAGGAGCACCAGATATGGCATTCCC

ACGACTTAACAACCTAAGATTTTGGTTACTCCCGCCTTCTTTAACTTTATTAGTAGCATC

GGCTACAGTTGAAAAGGGAGCCGGCACAGGATGAACAGTTTATCCACCTCTATCAAGAAA

TTTAGCTCATGCAGGTCCATCAGTAGACCTAGCAATCTTTTCACTACATCTAGCTGGGGC

ATCCTCCATTCTAGGAGCTATTAACTTTATTACTACGGTAATTAATATACGATGAAATGG

ACTCCGACTTGAACGAGTTCCATTATTTGTATGAGCAGTAACAATTACAGTTGTTCTATT

ACTATTATCGTTACCAGTACTAGCTGGGGCAATCACTATACTTCTTACAGATCGAAACTT

AAATACAACATTTTTCGATCCGGCTGGTGGTGGAGACCCAGTATTATATCAACATTTATT

T--------------------------------------------

>832_Lumbriculidae_sp_LL3

---AACCTTATATTTCATCTTAGGAGTTTGAGCTGGAATAGTAGGAGCAGGAATAAGACT

ATTAATTCGAGTTGAATTAACACAACCAGGATCATTTTTAGGAAGTGATCAATTATATAA

TACTATTGTTACCGCCCACGCCTTTATTATAATTTTCTTTATAGTTATACCTATATTTAT

TGGTGGCTTTGGTAACTGAATATTACCATTAATATTAGGAGCACCAGATATAGCATTCCC

ACGACTTAACAACCTAAGATTTTGGTTACTCCCACCTTCTTTAACTCTATTAGTAGCATC

GGCTGCAGTTGAAAAGGGAGCCGGCACAGGATGAACAGTTTATCCACCTCTATCAAGAAA

TTTAGCTCATGCAGGTCCATCAGTAGACCTAGCAATCTTTTCACTACATCTAGCTGGGGC

ATCCTCCATTCTAGGAGCTATTAACTTTATTACTACGGTAATTAATATACGATGAAATGG

ACTCCGACTTGAACGAGTTCCATTATTTGTATGAGCAGTAACAATTACAGTTGTTCTATT

ACTATTATCGTTACCAGTACTAGCTGGGGCAATCACTATACTTCTTACAGATCGAAACTT

AAATACAACATTTTTCGATCCGGCTGGTGGTGGAGACCCAGTATTATATCAACATTTATT

T--------------------------------------------

>834_Potamothrix_hammoniensis_T28

---ACTCCTTTATATAATATATGGACTATGAACAGGTATGGTGGGAACTGGAACTAGAAT

ACTAATCCGAATAGAATTAGCTCAACCTGGATCCTTCCTTGGAAGGGACCAACTATATAA

TACTCTTGTAACTGCTCATGCCTTCTTAATAATTTTCTTCTTGGTTATACCGGTATATAT

TGGTGCCTTCGGAAACTTTCTTGTACCTTTAATACTAGGTGCACCTGATATAGCTTTCCC

CCGAATAAATAATTTAAGATTTTGACTTATACCTTCATCTGTAATTCTATGCGTAGCATC

TGCAGCAGTTGAAAAAGGAGCAGGAACAGGTTGAACAGTATACCCGCCCTTAGCAAGAAA

TCTTGCTCACTCGGGACCATCTGTAGATTTAGCAATCTTCTCTTTACATTTAGCCGGAAT

TTCCTCTATTTTAGGGGCAATCAATTTTATTACTACAATAATTAACATACGTTGAAAAGG

AATGCGATTAGAACGAATTCCTTTATTTGTGTGAGCGACAATCATTACTGTTCTTCTTCT

ATTATTAACTCTACCAGTTCTTGCTGGTGCTATTACCATACTATTAACAGATCGAAATCT

AAATACCTCATTCTTTGATCCTGCTGGTGGTGGGGACCCTGTTCTATATCAACACCTATT

C--------------------------------------------

>837_Tubificinae_sp_T2

---ACTTCTCTATATAATGTATGGCCTATGAACAGGTATGGTAGGAACAGGAACTAGAAT

TCTAATTCGAATAGAACTTGCCCAACCAGGATCATTCCTTGGGAGAGACCAGCTATATAA

TACACTCGTAACTGCCCATGCTTTCTTAATAATTTTCTTCCTAGTAATGCCCGTATACAT

TGGTGCTTTTGGAAACTTCCTGGTACCATTAATACTTGGCGCACCAGATATAGCATTTCC

ACGAATAAATAATCTCAGATTCTGATTAATACCTTCCTCTGTAATTTTATGTGTAGCATC

TGCTGCCGTTGAAAAAGGAGCCGGTACAGGTTGAACCGTTTACCCTCCATTAGCAAGAAA

TCTTGCTCACTCTGGCCCATCTGTAGACTTAGCCATCTTCTCCCTTCACTTAGCAGGTAT

TTCCTCTATTTTAAGAGCAATTAACTTCATTACTACAATAATCAATATACGATGAAAAGG

AATACGACTTGAACGAATTCCTTTATTTGTATGAGCTACAATCATTACAGTAATTCTACT

ACTATTAACACTTCCAGTTCTAGCTGGGGCTATCACCATATTACTAACAGACCGAAATCT

AAATACCTCATTCTTTGACCCTGCAGGAGGGGGAGACCCTGTGCTTTATCAACATTTATT

C--------------------------------------------

>838_Tubificinae_sp_T2

---ACTTCTCTATATAATGTATGGCCTATGAACAGGTATGGTAGGAACAGGAACTAGAAT

TCTAATTCGAATAGAACTTGCCCAACCAGGATCATTCCTTGGGAGAGACCAGCTATATAA

TACACTCGTAACTGCCCATGCTTTCTTAATAATTTTCTTCCTAGTAATGCCCGTATACAT

TGGTGCTTTTGGAAACTTCCTGGTACCATTAATACTTGGCGCACCAGATATAGCATTTCC

ACGAATAAATAATCTCAGATTCTGATTAATACCTTCCTCTGTAATTTTATGTGTAGCATC

TGCTGCCGTTGAAAAAGGAGCCGGTACAGGTTGAACCGTTTACCCTCCATTAGCAAGAAA

TCTTGCTCACTCTGGCCCATCTGTAGACTTAGCCATCTTCTCCCTTCACTTAGCAGGTAT

TTCCTCTATTTTAAGAGCAATTAACTTCATTACTACAATAATCAATATACGATGAAAAGG

AATACGACTTGAACGAATTCCTTTATTTGTATGAGCTACAATCATTACAGTAATTCTACT

ACTATTAACACTTCCAGTTCTAGCTGGGGCTATCACCATATTACTAACAGACCGAAATCT

AAATACCTCATTCTTTGACCCTGCAGGAGGGGGAGACCCTGTGCTTTATCAACATTTATT

C--------------------------------------------

>839_Embolocephalus_velutinus_T25

---AACCCTATATATAGTATTTGGTGTATGAGCCGGTATAGTTGGAACCGGCACTAGATT

ACTAATCCGCTTTGAGNTCGCGCAACCTGGATCATTCCTAGGTAGTGACCAACTCTACAA

TACGTTAGTTACTGCTCACGCCTTCCTTATAATCTTTTTCCTAGTAATACCTGTATTCAT

CGGGGGATTCGGAAATTGACTTCTCCCATTAATATTAGGGGCACCAGATATAGCATTCCC

TCGCTTAAATAATCTAAGATTTTGGCTGCTGCCACCATCTTTAATTCTTCTAGTATCTTC

GGCGGCTGTAGAAAAGGGGGCCGGAACAGGGTGAACAGTGTACCCCCCACTATCAGGTAA

CTTAGCCCATTCGGGACCATCAGTAGACTTAGCAATCTTTTCTCTTCATTTAGCTGGTGC

ATCGTCCATTTTAGGGGCAATCAACTTCATTACTACAGTAATTAACATACGAAGTAAAGG

TATGCGGCTGGAACGGGTACCACTATTTGTATGGGCTGTCCTTCTTACAGTAATTCTTCT

CCTGTTAACCCTTCCAGTACTCGCCGGAGCTATTACTATATTACTGACAGACCGCAATCT

AAATACGTCGTTCTTTGATCCAGCTGGAGGTGGTGACCCAGTACTATACCAACACCTATT

C--------------------------------------------

>840_Embolocephalus_velutinus_T25

---AACCCTATATATAGTATTTGGTGTATGAGCCGGTATAGTTGGAACCGGCACTAGATT

ACTAATCCGCTTTGAGCTCGCGCAACCTGGATCATTCCTAGGTAGTGACCAACTCTACAA

TACGTTAGTTACTGCTCACGCCTTCCTTATAATCTTTTTCCTAGTAATACCTGTATTCAT

CGGGGGATTCGGAAATTGACTTCTCCCATTAATATTAGGGGCACCAGATATAGCATTCCC

TCGCTTAAATAATCTAAGATTTTGGCTGCTGCCACCATCTTTAATTCTTCTAGTATCTTC

GGCGGCTGTAGAAAAGGGGGCCGGAACAGGGTGAACAGTGTACCCCCCACTATCAGGTAA

CTTAGCCCATTCGGGACCATCAGTAGACTTAGCAATCTTTTCTCTTCATTTAGCTGGTGC

ATCGTCCATTTTAGGGGCAATCAACTTCATTACTACAGTAATTAACATACGAAGTAAAGG

TATGCGGCTGGAACGGGTACCACTATTTGTATGGGCTGTCCTTCTTACAGTAATTCTTCT

CCTGTTAACCCTTCCAGTACTCGCCGGAGCTATTACTATATTACTGACAGACCGCAATCT

AAATACGTCGTTCTTTGATCCAGCTGGAGGTGGTGACCCAGTACTATACCAACACCTATT

C--------------------------------------------

>841_Embolocephalus_velutinus_T25

---AACCCTATATATAGTATTTGGTGTATGAGCCGGTATAGTTGGAACCGGCACTAGATT

ACTAATCCGCTTTGAGCTCGCGCAACCTGGATCATTCCTAGGTAGTGACCAACTCTACAA

TACGTTAGTTACTGCTCACGCCTTCCTTATAATCTTTTTCCTAGTAATACCTGTATTCAT

CGGGGGATTCGGAAATTGACTTCTCCCATTAATATTAGGGGCACCAGATATAGCATTCCC

TCGCTTAAATAATCTAAGATTTTGGCTGCTGCCACCATCTTTAATTCTTCTAGTATCTTC

GGCGGCTGTAGAAAGGGGGGCCGGAACAGGGTGAACAGTGTACCCCCCACTATCAGGTAA

CTTAGCCCATTCGGGACCATCAGTAGACTTAGCAATCTTTTCTCTTCATTTAGCTGGTGC

ATCGTCCATTTTAGGGGCAATCAACTTCATTACTACAGTAATTAACATACGAAGTAAAGG

TATGCGGCTGGAACGGGTACCACTATTTGTATGGGCTGTCCTTCTTACAGTAATTCTTCT

CCTGTTAACCCTTCCAGTACTCGCCGGAGCTATTACTATATTACTGACAGACCGCAATCT

AAATACGTCGTTCTTTGATCCAGCTGGAGGTGGTGACCCAGTACTATACCAACACCTATT

C--------------------------------------------

>842_Limnodrilus_hoffmeisteri_T20

---CACCCTTTACATAGTTTTTGGCTTATGAGCCGGAATAGTAGGTACCGGGACAAGATT

ACTAATTCGCTTCGAACTGGCACAACCGGGATCATTCCTAGGCAGAGATCAGTTATATAA

TACATTAGTAACTGCTCATGGTTTCTTAATAATTTTCTTCATGGTGATACCAATCTTTAT

CGGTGGTTTTGGAAATTGATTAATTCCTTTAATACTTGGAGCCCCAGATATAGCGTTTCC

TCGACTAAATAATCTTAGATTTTGATTAATGCCACCCTCTCTGATTCTACTTGTATCGTC

AGCAGCCGTGGAAAAAGGTGCAGGTACCGGATGAACTGTATACCCGCCGCTAGCTAGAAA

TCTGGCACATTCAGGTCCATCGGTAGATCTAGCAATTTTTTCTTTACATTTAGCAGGTGC

AGCTTCAATTCTCGGGGCAATTAACTTCATTACTACAATAATTAACATACGGTGAAAAGG

AATACGTTTAGAACGTATTCCACTATTTGTGTGATCCGTAATTATTACTGTAGTCCTTCT

TCTACTAACCCTACCTGTATTAGCAGGAGCCATTACAATATTATTAACAGATCGAAATCT

AAATACATCATTCTTTGACCCTGCAGGAGGTGGAGACCCTGTACTCTACCAACATCTATT

T--------------------------------------------

>843_Aulodrilus_pluriseta_T4

---AACTCTATACTTCATTTTCGGAATCTGAGCCGGTATAGTTGGTACCGGTACCAGACT

ATTAATTCGCTTAGAACTGGCACAACCTGGATCCTTCTTAGGAAGTGATCAACTATATAA

TACATTAGTAACAGCTCACGCATTTCTAATAATCTTCTTCCTAGTTATACCTGTATTCAT

CGGGGGATTCGGAAATTGATTAATCCCATTAATACTAGGTGCCCCTGATATAGCATTTCC

ACGACTAAATAACCTAAGATTTTGACTAATACCGCCCTCACTAATTCTACTAGTTTCATC

CGCAGCTGTAGAAAAAGGTGCAGGGACAGGTTGAACAGTATATCCACCACTTGCTGGCAA

TCTAGCCCATTCAGGGCCTTCAGTAGATCTGGCAATCTTCTCTCTACACTTAGCAGGGGT

AGCATCAATTCTAGGTGCTATTAACTTCATTACCACTATAATCAACATACGATGAAAAGG

AATGCGCCTAGAACGAATTCCTCTATTTGTCTGAGCCGTAATTCTTACCGTTGTACTCCT

TCTATTAACCTTACCAGTTCTGGCAGGGGCAATTACTATACTTCTAACTGACCGCAATCT

AAACACCTCCTTCTTCGATCCAGCTGGGGGCGGTGACCCTGTATTATACCAACATCTATT

C--------------------------------------------

>844_Tubifex_sp_T26

---AACACTATATATCATTTTTGGGGTATGAGCAGGAATGGTCGGAACAGGTACTAGATT

ATTAATTCGACTAGAACTTGCCCAACCTGGCTCATTTCTTGGTAGCGATCAATTATTTAA

TACTCTGGTTACAGCCCATGCATTCCTGATAATCTTCTTCATAGTAATACCAATCTACAT

CGGGGGATTCGGAAACTGATTAATTCCCTTAATACTGGGCGCCCCTGACATGGCCTTCCC

TCGTCTAAATAACTTAAGATTCTGACTTCTTCCACCATCTCTAATTCTATGTATTTCCTC

GGCAGCCGTTGAAAAAGGTGCTGGAACTGGGTGAACTGTATACCCCCCACTAGCAAGAAA

CCTTGCCCACTCGGGACCATCTGTAGATCTTGCTATTTTCTCCTTACACTTAGCCGGGGT

TGCATCAATTCTTGGTGCCATTAACTTTATTACAACTATAATCAACATACGATGAAAAGG

AATACGACTTGAACGAATCCCTCTATTTGTATGATCTGTAATTATTACAGTAGTTCTCCT

ACTTCTTACATTACCAGTATTAGCGGGTGCTATCACTATACTACTAACAGACCGAAACCT

AAATACCTCATTCTTCGATCCTGCCGGAGGAGGTGACCCAGTTCTTTACCAACATCTATT

C--------------------------------------------

>845_Potamothrix_heuscheri_T31

---GCTTCTATATATAATATATGGCCTATGAACAGGTATAGTGGGAACCGGGACTAGAAT

TCTAATTCGTATAGAACTAGCTCAACCTGGATCATTTCTAGGCAGGGACCAACTATATAA

TACACTTGTAACTGCTCATGCCTTTTTAATAATTTTCTTCTTAGTAATACCAGTATATAT

TGGGGCTTTTGGAAACTTTCTTGTTCCTTTAATACTAGGTGCACCAGATATAGCATTCCC

TCGAATAAACAATCTTAGATTCTGATTAATACCATCTTCTGTAATCTTATGTGTAGCTTC

TGCTGCCGTAGAAAAAGGGGCCGGTACAGGATGAACAGTATATCCTCCATTAGCAAGAAA

TCTTGCTCACTCTGGACCATCTGTAGATCTTGCCATTTTCTCTCTTCACTTAGCGGGGAT

CTCCTCTATTCTAGGCGCAATTAATTTCATTACTACGATAATTAATATACGATGAAAAGG

AATGCGCCTAGAACGAATTCCTTTATTTGTATGAGCTACAATTATTACAGTAGTCTTACT

ACTACTAACACTTCCAGTTCTAGCTGGGGCTATTACTATGCTTCTAACAGACCGAAACCT

GAATACCTCATTCTTTGATCCTGCAGGTGGAGGGGATCCTGTCCTGTACCAACATTTATT

C--------------------------------------------

>847_Potamothrix_heuscheri_T31

---GCTTCTATATATAATATATGGCCTATGAACAGGTATAGTGGGAACCGGGACTAGAAT

TCTAATTCGTATAGAACTAGCTCAACCTGGATCATTTCTAGGCAGGGACCAACTATATAA

TACACTTGTAACTGCTCATGCCTTTTTAATAATTTTCTTCTTAGTAATACCAGTATATAT

TGGGGCTTTTGGAAACTTTCTTGTTCCTTTAATACTAGGTGCACCAGATATAGCATTCCC

TCGAATAAACAATCTTAGATTCTGATTAATACCATCTTCTGTAATCTTATGTGTAGCTTC

TGCTGCCGTAGAAAAAGGGGCCGGTACAGGATGAACAGTATATCCTCCATTAGCAAGAAA

TCTTGCTCACTCTGGACCATCTGTAGATCTTGCCATTTTCTCTCTTCACTTAGCGGGGAT

CTCCTCTATTCTAGGCGCAATTAATTTCATTACTACGATAATTAATATACGATGAAAAGG

AATGCGCCTAGAACGAATTCCTTTATTTGTATGAGCTACAATTATTACAGTAGTCTTACT

ACTACTAACACTTCCAGTTCTAGCTGGGGCTATTACTATGCTTCTAACAGACCGAAACCT

GAATACCTCATTCTTTGATCCTGCAGGTGGAGGGGATCCTGTCCTGTACCAACATTTATT

C--------------------------------------------

>848_Potamothrix_vejdovskyi_T29

---ATTCCTTTACATAATATATGGTTTATGAACAGGTATAGTAGGAACGGGAACTAGAAT

TTTAATCCGGATAGAGCTAGCTCAACCGGGATCATTCCTTGGGAGTGACCAGCTATATAA

TACTCTTGTAACTGCTCACGCTTTTCTGATAATCTTCTTCTTAGTGATACCTGTTTATAT

CGGCGCATTTGGGAACTTTCTTATACCTTTAATACTCGGGGCACCTGACATAGCATTTCC

ACGACTTAATAATTTAAGATTTTGATTGATACCTTCATCCGTAATTCTATGCGTAGCCTC

TGCAGCTGTAGAAAAGGGAGCAGGTACAGGCTGAACAGTATACCCTCCTCTAGCTAGGAA

CCTTGCTCATTCTGGACCGTCTGTGGATTTAGCCATTTTCTCTTTACATTTAGCAGGTAT

CGCCTCTATTTTAGGTGCAATCAATTTTATTACAACCATAATTAATATGCGGTGAATAGG

AATACGGTTAGAACGAATTCCTTTATTTGTATGAGCTACAATTATTACAGTTCTCCTTCT

ATTACTTACCCTACCTGTTCTGGCGGGGGCTATTACTATACTCTTAACAGATCGAAATCT

AAATACCTCATTCTTTGACCCTGCGGGAGGGGGAGACCCTGTACTATATCAGCATCTATT

C--------------------------------------------

>850_Limnodrilus_hoffmeisteri_T18

-----------------------------------------------CAGGAACAAGCCT

GCTAATTCGATTCGAATTAGCACAACCTGGTTCATTCCTCGGTAGAGATCAACTCTATAA

TACCTTAGTGACAGCCCACGGTTTCCTTATAATCTTCTTCATGGTAATACCAATTTTTAT

TGGTGGCTTCGGAAATTGACTAGTCCCCCTAATGCTAGGAGCTCCTGACATGGCCTTTCC

ACGACTAAATAACCTAAGATTTTGACTAATACCTCCATCACTCATTCTATTAGTTTCATC

AGCCGCAGTCGAAAAGGGAGCGGGGACAGGGTGAACTGTATACCCCCCTCTAGCCAGAAA

CCTAGCGCACTCTGGGCCATCTGTGGATCTAGCAATCTTCTCTCTTCACTTAGCCGGGGC

TGCATCAATTCTAGGTGCCATTAACTTCATTACCACAATAATTAATATACGATGAAAAGG

AATGCGCCTAGAGCGCATTCCTCTATTTGTATGATCAGTAATCATTACAGTTGTCCTCCT

TCTTCTTACATTACCGGTTTTAGCAGGGGCAATCACCATACTTTTAACAGACCGAAACTT

AAACACATCATTCTTCGATCCTGCGGGAGGGGGGGACCCGGTACTATATCAACACTTATT

---------------------------------------------

>851_Potamothrix_heuscheri_T31

---GCTTCTATATATGATATATGGCCTATGAACAGGTATAGTGGGAACCGGGACTAGAAT

TCTAATTCGTATAGAACTAGCTCAACCTGGATCATTTCTAGGCAGGGACCAACTATATAA

TACACTTGTAACTGCTCATGCCTTTTTAATAATTTTCTTCTTAGTAATACCAGTATATAT

TGGGGCTTTTGGAAACTTTCTTGTTCCTTTAATACTAGGTGCACCAGATATAGCATTCCC

TCGAATAAACAATCTTAGATTCTGATTAATACCATCTTCTGTAATCTTATGTGTAGCTTC

TGCTGCCGTAGAAAAAGGGGCCGGTACAGGATGAACAGTATATCCTCCATTAGCAAGAAA

TCTTGCTCACTCTGGACCATCTGTAGATCTTGCCATTTTCTCTCTTCACTTAGCGGGGAT

CTCCTCTATTCTAGGCGCAATTAATTTCATTACTACGATAATTAATATACGATGAAAAGG

AATGCGCCTAGAACGAATTCCTTTATTTGTATGAGCTACAATTATTACAGTAGTCTTACT

ACTACTAACACTTCCAGTTCTAGCCGGGGCTATTACTATGCTTCTAACAGACCGAAACCT

GAATACCTCATTCTTTGATCCTGCGGGTGGAGGNGATCCTGTCCTGTACCAACATTTATT

C--------------------------------------------

>852_Tubifex_tubifex_T11

---AACTCTATACATCATTTTCGGGATTTGAGCTGGGATGGTCGGGACAGGAACTAGACT

CTTAATTCGCCTAGAACTGGCTCAACCTGGCTCATTCCTTGGAAGAGATCAGCTATACAA

CACCCTAGTTACAGCACATGCATTTCTGATAATTTTCTTCATGGTAATACCTATCTATAT

TGGGGGTTTCGGAAATTGGTTAGTACCCTTAATGCTGGGGGCTCCTGACATAGCATTCCC

CCGATTAAATAATTTAAGATTTTGGTTATTACCCCCTTCCTTAATCCTCCTTGTATCCTC

CGCGGCCGTAGAAAAGGGGGCTGGGACAGGGTGAACAGTATATCCGCCCCTAGCTAGAAA

TCTGGCTCATTCTGGGCCTTCTGTAGACTTAGCTATTTTCTCTCTACATTTAGCTGGGGT

GGCATCGATTCTAGGAGCTATTAACTTTATTACTACAATAATTAACATGCGATGAAAAGG

GATACGCCTTGAACGAATTCCCCTATTTGTGTGAGCTGTAATTCTAACCGTAATCTTACT

TTTACTAACATTACCAGTTTTAGCTGGTGCCATTACCATGCTACTAACAGATCGAAATCT

AAACACATCCTTCTTTGACCCTGCTGGGGGTGGTGATCCTGTACTCTATCAACACTTATT

C--------------------------------------------

>853_Tubifex_tubifex_T12

-----------------TTTTCGGAATCTGAGCTGGTATGGTTGGAACTGGGACTAGGCT

ATTAATTCGCCTTGAACTTGCCCAACCTGGGTCCTTTTTAGGAAGAGATCAATTATATAA

TACCCTGGTAACGGCTCACGCATTTTTAATAATCTTCTTCATAGTAATACCAATCTATAT

TGGGGGCTTCGGCAACTGACTAGTACCATTAATACTAGGGGCTCCGGACATGGCCTTCCC

ACGATTAAACAACTTAAGATTCTGACTCCTACCCCCATCACTAATCCTTTTAGTATCCTC

TGCCGCCGTTGAAAAAGGCGCCGGAACTGGCTGAACTGTTTACCCCCCTCTAGCTAGAAA

TCTAGCTCACTCTGGGCCCTCAGTAGATTTAGCAATCTTTTCTCTACACTTAGCTGGTGT

TGCATCAATCCTAGGTGCAATCAACTTCATTACTACAATAATCAACATACGATGAAAAGG

AATGCGACTAGAGCGTATTCCTTTATTTGTATGATCAGTAATTATCACTGTAATTCTACT

CCTACTCACACTTCCAGTTTTAGCTGGAGCTATTACAATACTTCTAACAGACCGGAATCT

AAATACATCATTTTTCGATCCTGCTGGAGGGGGTGACCCTGTACTATATCAACACCTATT

C--------------------------------------------

>854_Potamothrix_heuscheri_T31

---GCTTCTATATATAATATATGGCCTATGAACAGGTATAGTGGGAACCGGGACTAGAAT

TCTAATTCGTATAGAACTAGCTCAACCTGGATCATTTCTAGGCAGGGACCAACTATATAA

TACACTTGTAACTGCTCATGCCTTTTTAATAATTTTCTTCTTAGTAATACCAGTATATAT

TGGGGCTTTTGGAAACTTTCTTGTTCCTTTAATACTAGGTGCACCAGATATAGCATTCCC

TCGAATAAACAATCTTAGATTCTGATTAATACCATCTTCTGTAATCTTATGTGTAGCTTC

TGCTGCCGTAGAAAAAGGGGCCGGTACAGGATGAACAGTATATCCTCCATTAGCAAGAAA

TCTTGCTCACTCTGGACCATCTGTAGATCTTGCCATTTTCTCTCTTCACTTAGCGGGGAT

CTCCTCTATTCTAGGCGCAATTAATTTCATTACTACGATAATTAATATACGATGAAAAGG

AATGCGCCTAGAACGAATTCCTTTATTTGTATGAGCTACAATTATTACAGTAGTCTTACT

ACTACTAACACTTCCAGTTCTAGCTGGGGCTATTACTATGCTTCTAACAGACCGAAACCT

GAATACCTCATTCTTTGATCCTGCGGGTGGAGGGGATCCTGTCCTGTACCAACATTTATT

C--------------------------------------------

>855_Potamothrix_hammoniensis_T28

---ACTCCTTTATATAATGTACGGACTATGAACAGGTATGGTGGGAACTGGAACTAGAAT

ACTAATTCGAATAGAACTAGCTCAACCGGGGTCCTTCCTTGGAAGGGACCAACTATACAA

TACCCTTGTAACTGCTCATGCCTTCTTAATAATTTTCTTCTTAGTCATGCCAGTATATAT

TGGTGCCTTCGGAAACTTTCTTGTACCTTTAATACTAGGGGCCCCTGATATGGCTTTCCC

CCGAATAAATAATTTAAGATTTTGACTTATACCCTCATCTGTAATTCTATGTGTAGCATC

TGCAGCAGTTGAAAAAGGAGCAGGAACAGGTTGAACAGTATACCCCCCCTTAGCAAGAAA

TCTTGCTCACTCGGGGCCATCTGTAGATTTAGCAATCTTCTCTTTACATTTAGCAGGAAT

TTCCTCTATTTTAGGAGCAATCAATTTTATTACTACAATAATTAACATGCGTTGAAAAGG

TATACGATTAGAACGAATTCCTTTATTTGTGTGAGCAACAATCATTACTGTTCTTCTTCT

ATTATTAACTCTACCCGTTCTTGCTGGTGCTATTACCATACTATTAACAGATCGAAATCT

AAATACCTCATTCTTCGATCCTGCTGGTGGCGGGGACCCTGTTCTATATCAACACCTATT

C--------------------------------------------

>856_Tubifex_tubifex_T27

---AACCCTTTATATAGTATTTGGTATTTGAGCAGGTATAGTTGGCACCGGAACAAGTCT

ACTGATTCGTCTAGAACTAGCTCAACCTGGCTCTTTCCTCGGCAGCGACCAATTATATAA

CACATTAGTTACAGCACATGCATTCCTTATAATCTTCTTTATGGTAATACCTATTTACAT

TGGGGGATTCGGAAATTGACTAGTCCCACTAATACTGGGGGCACCAGACATAGCATTCCC

ACGACTAAATAATCTTAGATTTTGACTTCTACCCCCCTCCCTAATTCTACTTGTATCATC

TGCTGCAGTAGAAAAAGGAGCTGGAACAGGGTGAACTGTCTACCCACCACTAGCCAGTAA

CTTAGCACACTCTGGACCCTCAGTAGACTTAGCAATCTTCTCACTACACTTAGCTGGTGT

AGCCTCAATTCTAGGTGCCATTAACTTCATCACTACAATAATTAATATACGTTGAAAAGG

TATACGCCTAGAACGAATCCCATTATTTGTCTGATCAGTAATTATTACTGTAATTCTTTT

ACTACTTACACTTCCAGTACTAGCCGGTGCAATTACCATACTACTAACAGACCGAAATCT

AAATACTTCATTCTTCGACCCTGCCGGTGGGGGAGACCCTGTTCTCTATCAACATCTATT

C--------------------------------------------

>857_Tubifex_tubifex_T11

---AACTCTATATATCATTTTCGGGATTTGAGCTGGGATGGTCGGGACAGGAACTAGACT

CTTAATTCGTCTAGAACTGGCTCAACCTGGCTCATTCCTTGGAAGGGATCAGCTATACAA

CACCCTAGTTACAGCACATGCATTTCTGATAATTTTCTTCATGGTAATACCTATCTATAT

TGGGGGTTTCGGAAATTGGTTAGTACCCTTAATGCTGGGAGCTCCTGACATAGCATTCCC

CCGATTAAATAATTTAAGATTTTGGTTATTACCCCCTTCCTTAATCCTCCTTGTATCCTC

CGCGGCCGTAGAAAAGGGGGCTGGAACAGGGTGAACAGTATATCCGCCCCTAGCTAGAAA

TCTGGCTCATTCTGGGCCTTCTGTAGACTTAGCTATTTTCTCTCTACATTTAGCTGGGGT

GGCATCGATTCTAGGAGCTATTAACTTTATTACTACAATAATTAACATGCGATGAAAAGG

GATACGCCTTGAACGAATTCCCCTATTTGTATGAGCTGTAATTCTAACCGTAATCTTACT

TTTACTAACATTACCAGTTTTAGCTGGTGCCATTACCATGCTACTAACAGATCGAAATCT

AAACACATCCTTCTTTGACCCTGCTGGGGGTGGAGATCCTGTGCTCTATCAACACTTATT

C--------------------------------------------

>858_Limnodrilus_hoffmeisteri_T18

-------------------------------------------------------AGCCT

GCTAATTCGATTCGAATTAGCACAACCCGGTTCATTCCTCGGTAGAGATCAACTCTATAA

TACCTTAGTAACAGCCCACGGTTTCCTTATAATCTTCTTCATGGTAATACCAATTTTTAT

TGGTGGCTTCGGAAATTGACTAGTCCCCCTAATGCTAGGAGCTCCTGACATGGCCTTTCC

ACGACTAAATAACCTAAGATTTTGACTAATACCTCCATCACTCATTCTATTAGTTTCATC

AGCCGCGGTCGAAAAGGGAGCGGGGACAGGGTGAACTGTATACCCCCCTCTAGCCAGAAA

CCTAGCGCACTCTGGGCCATCTGTGGATCTAGCAATCTTCTCTCTTCACTTAGCCGGGGC

TGCATCAATTCTAGGTGCCATTAACTTCATTACCACAATAATTAATATACGATGAAAAGG

AATGCGCCTAGAGCGCATTCCTCTATTTGTATGATCAGTAATCATTACAGTTGTCCTCCT

TCTTCTTACATTACCGGTTTTAGCGGGGGCAATCACCATACTTTTAACAGACCGAAACTT

AAACACATCATTCTTCGATCCTGCNGGAGGGGGGGACCCGGTACTATATCAACACTTATT

---------------------------------------------

>859_Limnodrilus_hoffmeisteri_T20

---CACCCTTTACATAGTTTTTGGCTTATGAGCCGGAATAGTAGGTACCGGGACAAGATT

ACTAATTCGCTTCGAACTGGCACAACCGGGATCATTCCTAGGCAGAGATCAGTTATATAA

TACATTAGTAACTGCTCATGGTTTCTTAATAATTTTCTTCATGGTGATACCAATCTTTAT

CGGTGGTTTTGGAAATTGATTAATTCCTTTAATACTTGGAGCCCCAGATATAGCGTTTCC

TCGACTAAATAATCTTAGATTTTGATTAATGCCACCCTCTCTGATTCTACTTGTATCGTC

AGCAGCCGTGGAAAAAGGTGCAGGTACCGGATGAACTGTATACCCGCCGCTAGCTAGAAA

TCTGGCACATTCAGGTCCATCGGTAGATCTAGCAATTTTTTCTTTACATTTAGCAGGTGC

AGCTTCAATTCTCGGGGCAATTAACTTCATTACTACAATAATTAACATACGGTGAAAAGG

AATACGTTTAGAACGTATTCCACTATTTGTGTGATCCGTAATTATTACTGTAGTCCTTCT

TCTACTAACCCTACCTGTATTAGCAGGAGCCATTACAATATTATTAACAGATCGAAATCT

AAATACATCATTCTTTGACCCTGCAGGAGGTGGAGACCCTGTACTCTACCAACATCTATT

T--------------------------------------------

>860_Potamothrix_moldaviensis_T30

---ACTTCTTTATATCTTCTCTGGACTATGAACAGGTATGGTAGGAACAGGAACTAGAAT

TTTAATTCGTCTGGAACTTGCTCAGCCTGGATCATTCCTAGGTAGTGATCAGTTATATAA

CACTCTCGTAACTGCCCACGCCTTTCTGATAATCTTTTTTTTAGTAATACCTGTATATAT

TGGAGCTTTCGGGAACTTCCTAATTCCACTAATACTAGGGGCTCCTGATATAGCTTTCCC

ACGATTAAACAATTTAAGATTTTGACTTATACCCTCATCTGTAATCCTGTGTGTGGCATC

TGCAGCTGTTGAAAAGGGGGCAGGAACAGGTTGAACAGTATACCCTCCCCTAGCAAGAAA

CCTTGCCCATTCCGGGCCTTCGGTAGACCTGGCCATTTTCTCTCTTCATCTAGCAGGAAT

TGCTTCCATTTTAGGCGCAATTAACTTCATTACAACAATAATTAATATACGTTGAAAAGG

AATACGGTTAGAGCGAATCCCCTTATTCGTTTGAGCGACAATCATTACAGTTCTTTTACT

ACTATTAACTCTACCCGTATTAGCGGGCGCTATTACCATACTATTAACAGACCGAAATCT

AAATACCTCATTCTTCGATCCTGCTGGGGGAGGAGACCCTGTCTTATATCAACACCTATT

T--------------------------------------------

>866_Potamothrix_moldaviensis_T30

---ACTTCTTTATATATTATATGGACTATGAACAGGTATGGTGGGAACAGGAACTAGAAT

TTTAATTCGTCTGGAACTTGCTCAGCCTGGGTCATTCCTAGGTAGTGATCAGTTATATAA

CACCCTCGTAACTGCTCACGCCTTTCTGATAATCTTTTTTCTAGTAATACCTGTATATAT

TGGAGCTTTCGGGAACTTCCTAATCCCACTAATACTAGGGGCTCCTGATATAGCTTTCCC

ACGGTTAAACAATTTAAGATTTTGACTTATACCTTCATCTGTAATCCTCCTTGTGGCATC

TGCAGCTGTTGAAAAGGGAGCAGGAACAGGTTGAACAGTGTACCCTCCCCTAGCAAGAAA

CCTTGCCCATTCCGGGCCTTCGGTAGACCTGGCCATTTTCTCTCTCCATCTAGCAGGAAT

TGCTTCCATTTTAGGGGCAATTAACTTCATTACAACAATAATTAATATACGTTGAAAAGG

GATACGATTAGAGCGAATCCCCTTATTCGTTTGAGCGACAATCATCACAGTTATTTTACT

ACTATTAACTCTACCCGTATTAGCGGGCGCTATTACCATACTATTAACAGACCGAAATCT

AAATACCTCATTCTTCGATCCTGCTGGGGGAGGAGACCCTGTCTTATATCAACACCTATT

T--------------------------------------------

>867_Limnodrilus_hoffmeisteri_T17

---------------------------------------------------AACTAGACT

TTTAATTCGATTTGAGCTAGCACAACCCGGCTCATTTCTCGGTAGAGACCAATTATATAA

CACTCTAGTCACGGCTCACGGATTTTTAATAATTTTCTTTATAGTAATACCTATCTTTAT

TGGGGGATTTGGAAATTGATTAGTGCCTTTAATACTTGGAGCACCTGATATGGCATTCCC

ACGGCTTAATAACCTAAGATTCTGACTAATGCCCCCATCACTAATTCTACTAGTCTCATC

AGCTGCAGTTGAAAAAGGCGCAGGGACAGGGTGAACTGTATACCCCCCCTTAGCAAGAAA

TCTAGCTCATTCCGGGCCTTCTGTAGATCTGGCAATTTTTTCACTACACTTAGCAGGAGC

CGCATCAATCCTGGGAGCAATTAACTTCATCACAACAATAATCAATATACGATGAAAGGG

AATACGCTTAGAGCGTATTCCTTTATTTGTGTGATCCGTAATCATCACAGTTATTTTACT

TCTTCTTACCCTTCCAGTTCTTGCCGGAGCTATCACCATACTTTTAACAGATCGAAACCT

AAACACCTCATTCTTTGATCCGGCAGTGNGAGGCGATCCAGTTTTATACCAATATCTATT

T--------------------------------------------

>868_Lumbriculidae_sp_LL3

------------------------------------------------------------

----------------TTAACACAACCAGGATCATTTTTAGGAAGTGATCAATTATATAA

TACTATTGTTACGGCTCACGCCTTTATTATAATTTTCTTTATAGTTATACCTATATTTAT

TGGTGGCTTTGGTAACTGAATATTACCATTAATATTAGGAGCACCAGATATGGCATTCCC

ACGACTTAACAACCTAAGATTTTGGTTACTCCCGCCTTCTTTAACTTTATTAGTAGCATC

GGCTGCAGTTGAAAAGGGAGCCGGCACAGGATGAACAGTTTATCCACCTCTATCAAGAAA

TTTAGCTCATGCAGGTCCATCAGTAGACCTAGCAATCTTTTCACTACATCTAGCTGGGGC

ATCCTCCATTCTAGGAGCTATTAACTTTATTACTACGGTAATTAATATACGATGAAATGG

ACTCCGACTTGAACGAGTTCCATTATTTGTATGAGCAGTAACAATTACAGTTGTTCTATT

ACTATTATCATTACCAGTACTAGCTGGGGCAATCACTATACTTCTTACAGATCGAAACTT

AAATACAACATTTTTCGATCCGGCTGGTGGTGGAGACCCAGTATTATATCAACATTTATT

T--------------------------------------------

>836_Limnodrilus_hoffmeisteri_T18

-------------------------CTATGAGCTGGAATAGTAGGCACAGGAACAAGCCT

GCTAATTCGATTCGAATTAGCACAACCTGGTTCATTCCTCGGTAGAGATCAACTCTATAA

TACCTTAGTGACAGCCCACGGTTTCCTTATAATCTTCTTCATGGTAATACCAATTTTTAT

TGGTGGCTTCGGAAATTGACTAGTCCCCCTAATACTAGGAGCTCCTGACATGGCCTTTCC

ACGACTAAATAACCTAAGATTTTGACTAATACCTCCATCACTCATTCTATTAGTTTCATC

AGCCGCAGTCGAAAAGGGAGCGGGGACAGGGTGAACTGTATACCCCCCTCTAGCCAGAAA

CCTAGCGCATTCTGGGCCATCTGTGGATCTAGCAATCTTCTCTCTTCACTTAGCCGGGGC

TGCATCAATTCTAGGTGCTATTAACTTCATTACCACAATAATTAATATACGATGAAAAGG

AATGCGCCTAGAGCGCATTCCTCTATTTGTATGATCAGTAATCATTACAGTTGTCCTCCT

TCTTCTTACATTACCGGTTTTAGCGGGGGCAATCACCATACTTTTAACAGACCGAAACTT

AAACACCTCATTCTTCGATCCTGCGGGGGGGGGGGNCCCGGTACTATATAAAAAACTATT

T--------------------------------------------

>874_Lumbriculidae_sp_LL3

---AACCTTATATTTCATCTTAGGAGTTTGAGCTGGAATAGTAGGAGCAGGAATAAGACT

ATTAATTCGAGTTGAATTAACACAACCAGGATCATTTTTAGGAAGTGATCAATTATATAA

TACTATTGTTACGGCCCACGCCTTTATTATAATTTTCTTTATAGTTATACCTATATTTAT

TGGTGGCTTTGGTAACTGAATATTACCATTAATATTAGGAGCACCAGATATAGCATTCCC

ACGACTTAACAACCTAAGATTTTGGTTACTCCCGCCTTCTTTAACTTTATTAGTAGCATC

GGCTGCAGTTGAAAAGGGAGCCGGCACAGGATGAACAGTTTATCCACCTCTATCAAGAAA

TTTAGCTCATGCAGGTCCATCAGTAGACCTAGCAATCTTTTCACTACATCTAGCTGGGGC

ATCCTCCATTCTAGGAGCTATTAACTTTATTACTACGGTAATTAATATACGATGAAATGG

ACTCCGACTTGAACGAGTTCCATTATTTGTATGAGCAGTAACAATTACAGTTGTTCTATT

ACTATTATCGTTACCAGTACTAGCTGGGGCAATCACTATACTTCTTACAGATCGAAACTT

AAATACAACATTTTTCGATCCGGCTGGTGGTGGAGACCCAGTATTATATCAACATTTATT

T--------------------------------------------

>880_Lumbriculidae_sp_LL3

---AACCTTATATTTCATCTTAGGAGTTTGAGCTGGAATAGTAGGAGCAGGAATAAGACT

ATTAATTCGAGTTGAATTAACACAACCAGGATCATTTTTAGGAAGTGATCAATTATATAA

TACTATTGTTACGGCCCACGCCTTTATTATAATTTTCTTTATAGTTATACCTATATTTAT

TGGTGGCTTTGGTAACTGAATATTACCATTAATATTAGGAGCACCAGATATAGCATTCCC

ACGACTTAACAACCTAAGATTTTGGTTACTCCCGCCTTCTTTAACTTTATTAGTAGCATC

GGCTGCAGTTGAAAAGGGAGCCGGCACAGGATGAACAGTTTATCCACCTCTATCAAGAAA

TTTAGCTCATGCAGGTCCATCAGTAGACCTAGCAATCTTTTCACTACATCTAGCTGGGGC

ATCCTCCATTCTAGGAGCTATTAACTTTATTACTACGGTAATTAATATACGATGAAATGG

ACTCCGACTTGAACGAGTTCCATTATTTGTATGAGCAGTAACAATTACAGTTGTTCTATT

ACTATTATCGTTACCAGTACTAGCTGGGGCAATCACTATACTTCTTACAGATCGAAACTT

AAATACAACATTTTTCGATCCGGCTGGTGGTGGAGACCCAGTATTATATCAACATTTATT

T--------------------------------------------

>885_Haplotaxis_gordioides_H1

---AACCCTTTACTTTATCCTAGGCATCTGAGGGGGACTTCTAGGAACAAGAATAAGAAT

AGTAATCCGAATTGAACTAAGACAACCAGGGTCATTCCTTGGTAGAGATCAATTATACAA

TACCATTGTTACTGCCCATGCCTTCCTAATAATTTTCTTTCTTGTCATACCAGTATTTAT

TGGAGGGTTCGGAAACTGACTCTTACCTCTAATGTTAGGAGCCCCCGACATAGCATTCCC

ACGACTCAACAATATAAGATTTTGACTTCTACCTCCTGCAACAATCCTTCTTGTGTCCTC

AGCAGCAGTAGAAAAAGGAGCAGGAACTGGCTGAACTGTTTACCCGCCCTTAGCCAGAAA

TCTTGCACATGCTGGCCCTTCTGTTGATCTCGCCATTTTCTCTCTTCATTTAGCAGGAGT

TTCCTCCATTTTAGGAGCAGTAAATTTCATCACCACTGTTGTCAATATACGATGAAATGG

CCTGCGGTTAGAACGAATTCCTTTATTCGTATGATCCGTAACTATTACAGTGGTTCTTCT

CCTTTTATCTCTACCAGTGCTAGCCGGCGCTATTACCATACTACTAACTGACCGAAATCT

TAATACATCATTCTTCGACCCAGCTGGAGGAGGAGATCCAGTTCTGTATCAACACCTATT

C--------------------------------------------

>888_Nais_elinguis_N4

---TACATTATACTTAATTCTAGGAGTATGAGCGGGAATAGTTGGAACTGGAACAAGAAT

ACTAATTCGAATTGAACTTGCTCAACCAGGAGCCTTCCTCGGAAGAGACCAACTCTATAA

CACCCTAGTTACAGCTCATGCATTTTTAATAATTTTCTTTCTTGTAATGCCAGTATTTAT

TGGTGGATTCGGAAATTGACTTCTACCACTAATACTTGGAGCCCCAGATATAGCATTTCC

ACGATTAAATAATCTAAGATTTTGATTACTACCACCATCATTAATTATACTAATTTCATC

AGCCGCAGTTGAAAAAGGTGCAGGAACAGGATGAACTGTTTACCCTCCATTATCAAGAAA

TTTAGCTCATGCTGGACCATCAGTTGACATGGCTATTTTTTCTCTTCATTTAGCAGGTGC

ATCATCTATTTTAGGTGCAGTAAATTTTATTACTACAGTAATAAATATACGATGAAACGG

TATACGATTAGAACGTTTACCTTTATTTGTATGAGCTGTATTCCTTACTGTAATTCTCCT

TCTACTATCGCTACCAGTATTAGCTGGCGCTATTACAATACTTCTTACAGACCGTAATTT

AAATACCTCATTCTTTGATCCAGCAGGAGGAGGAGATCCGATTCTTTATCAACACTTATT

T--------------------------------------------

>892_Haplotaxis_gordioides_H1

---AACCCTTTCTTTTATCCTAGGCATCTGAGGGGGACTTCTAGGAACAAGAATAAGAAT

AGTAATCCGAATTGAACTAAGACAACCAGGGTCATTCCTTGGTAGAGATCAATTATACAA

TACCATTGTTACTGCCCATGCCTTCCTAATAATTTTCTTTCTTGTCATACCAGTATTTAT

TGGAGGGTTCGGAAACTGACTCTTACCTCTAATGTTAGGAGCCCCCGACATAGCATTCCC

ACGACTCAACAATATAAGATTTTGACTTCTACCTCCTGCAACAATCCTTCTTGTGTCCTC

AGCAGCAGTAGAAAAAGGAGCAGGAACTGGCTGAACTGTTTACCCGCCCTTAGCCAGAAA

TCTTGCACATGCTGGCCCTTCTGTTGATCTCGCCATTTTCTCTCTTCATTTAGCAGGAGT

TTCCTCCATTTTAGGAGCAGTAAATTTCATCACCACTGTTGTCAATATACGATGAAATGG

CCTGCGGTTAGAACGAATTCCTTTATTCGTATGATCCGTAACTATTACAGTGGTTCTTCT

CCTTTTATCTCTACCAGTGCTAGCCGGCGCTATTACCATACTACTAACTGACCGAAATCT

TAATACATCATTCTTCGACCCAGCTGGAGGAGGAGATCCAGTTCTGTATCAACACCTATT

C--------------------------------------------

>899_Stylodrilus_heringianus_LL3

---AACCTTATATTTCATCTTAGGAGTTTGAGCTGGAATAGTAGGAGCAGGAATAAGACT

ATTAATTCGAGTTGAATTAACACAACCAGGATCATTTTTAGGAAGTGATCAATTATATAA

TACTATTGTTACGGCCCACGCCTTTATTATAATTTTCTTTATAGTTATACCTATATTTAT

TGGTGGCTTTGGTAACTGAATATTACCATTAATATTAGGAGCACCAGATATGGCATTCCC

ACGACTTAACAACCTAAGATTTTGGTTACTTCCGCCTTCTTTAACTTTATTAGTAGCATC

GGCTGCAGTTGAAAAGGGAGCCGGCACAGGATGAACAGTTTATCCACCTCTATCAAGAAA

TTTAGCTCATGCAGGTCCATCAGTAGACCTAGCAATCTTTTCACTACATCTAGCTGGGGC

ATCCTCCATTCTAGGAGCTATTAACTTTATTACTACGGTAATTAATATACGATGAAATGG

ACTCCGACTTGAACGAGTTCCATTATTTGTATGAGCAGTAACAATTACAGTTGTTCTATT

ACTATTATCGTTACCAGTACTAGCTGGGGCAATCACTATACTTCTTACAGATCGAAACTT

AAATACAACATTTTTCGATCCGGCTGGTGGTGGAGACCCAGTATTATATCAACATTTATT

T--------------------------------------------

>907_Psammoryctides_barbatus_T8

---TACTTTATATTTAATTTTTGGCTTATGAGCTGGGATGGTCGGAACTGGAACTAGTTT

ATTAATTCGTATAGAACTCGCTCAACCGGGATCATTCCTTGGAAGAGACCAGTTATATAA

CACACTAGTAACAGCACATGCTTTCCTAATAATCTTCTTCCTAGTAATACCCGTATTTAT

TGGTGGGTTTGGTAATTGACTTCTACCTTTAATACTTGGGGCACCAGATATAGCTTTTCC

ACGCCTAAACAATTTAAGATTTTGATTATTACCTCCATCCCTTATTCTTCTAGTATCATC

TGCTGCTGTTGAAAAAGGTGCTGGAACAGGATGAACAGTATATCCACCACTAGCTGGTAA

CCTGGCACACTCTGGCCCTTCTGTAGACCTGGCTATTTTCTCTCTTCATTTAGCTGGTGC

CGCATCTATTTTAGGAGCCATTAATTTTATTACTACTATAATTAATATACGGTGAAAAGG

TATACGATTAGAGCGAATTCCTCTATTTGTATGAGCTGTAATCATTACAGTAATTCTTCT

TTTATTAACCCTTCCAGTATTAGCAGGTGCAATTACTATACTACTAACGGATCGAAATCT

AAATACATCATTCTTTGATCCTGCTGGTGGTGGGGATCCTGTTCTTTATCAACACTTATT

C--------------------------------------------

>913_Psammoryctides_barbatus_T8

---TACTTTATATTTAATTTTTGGCTTATGAGCTGGGATGGTCGGAACTGGGACTAGTTT

ATTAATTCGTATAGAACTCGCTCAACCAGGATCATTCCTTGGAAGAGACCAGTTATATAA

CACACTAGTAACAGCACATGCTTTTCTAATAATCTTCTTCCTAGTAATACCCGTATTTAT

TGGTGGGTTTGGTAATTGACTTCTACCTTTAATACTTGGGGCACCAGATATAGCTTTCCC

ACGCCTAAACAATTTAAGATTTTGATTATTACCTCCATCCCTTATTCTTCTAGTATCATC

CGCTGCTGTTGAAAAAGGTGCTGGAACAGGATGAACAGTATATCCACCACTAGCTGGTAA

CCTGGCACACTCTGGCCCTTCTGTAGACCTGGCTATTTTCTCTCTTCATTTAGCTGGTGC

CGCATCTATTTTAGGAGCTATTAATTTTATTACTACTATAATTAATATACGGTGAAAAGG

TATACGATTAGAGCGAATTCCTTTATTTGTGTGAGCTGTAATCATTACAGTAGTTCTTCT

TTTATTAACCCTTCCAGTATTAGCCGGTGCAATTACTATACTACTAACGGATCGAAATCT

AAATACATCATTCTTTGATCCTGCTGGTGGTGGGGATCCTGTTCTTTATCAACACTTATT

C--------------------------------------------

>919_Psammoryctides_barbatus_T8

---TACTTTATATTTAATTTTTGGCTTATGAGCTGGGATGGTCGGAACTGGAACTAGTTT

ATTAATTCGTATAGAACTCGCTCAACCGGGATCATTCCTTGGAAGAGACCAGTTATATAA

CACACTAGTAACAGCACATGCTTTCCTAATAATCTTCTTCCTAGTAATACCCGTATTTAT

TGGTGGGTTTGGTAATTGACTTCTACCTTTAATACTTGGGGCACCAGATATAGCTTTTCC

ACGCCTAAACAATTTAAGATTTTGATTATTACCTCCATCCCTTATTCTTCTAGTATCATC

TGCTGCTGTTGAAAAAGGTGCTGGAACAGGATGAACAGTATATCCACCACTAGCTGGTAA

CCTGGCACACTCTGGCCCTTCTGTAGACCTGGCTATTTTCTCTCTTCATTTAGCTGGTGC

CGCATCTATTTTAGGAGCCATTAATTTTATTACTACTATAATTAATATACGGTGAAAAGG

TATACGATTAGAGCGAATTCCTCTATTTGTATGAGCTGTAATCATTACAGTAATTCTTCT

TTTATTAACCCTTCCAGTATTAGCAGGTGCAATTACTATACTACTAACGGATCGAAATCT

AAATACATCATTCTTTGATCCTGCTGGTGGTGGGGATCCTGTTCTTTATCAACACTTATT

C--------------------------------------------

>918_Stylodrilus_heringianus_LL3

---AACCTTATATTTCATCTTAGGAGTTTGAGCTGGAATAGTAGGAGCAGGAATAAGACT

ATTAATTCGAGTTGAATTAACACAACCAGGATCATTTTTAGGAAGTGATCAATTATATAA

TACTATTGTTACGGCCCACGCCTTTATTATAATTTTCTTTATAGTTATACCTATATTTAT

TGGTGGCTTTGGTAACTGAATATTACCATTAATATTAGGAGCACCAGATATAGCATTCCC

ACGACTTAACAACCTAAGATTTTGGTTACTCCCGCCTTCTTTAACTTTATTAGTAGCATC

GGCTGCAGTTGAAAAGGGAGCCGGCACAGGATGAACAGTTTATCCACCTCTATCAAGAAA

TTTAGCTCATGCAGGTCCATCAGTAGACCTAGCAATCTTTTCACTACATCTAGCTGGGGC

ATCCTCCATTCTAGGAGCTATTAACTTTATTACTACGGTAATTAATATACGATGAAATGG

ACTCCGACTTGAACGAGTTCCATTATTTGTATGAGCAGTAACAATTACAGTTGTTCTATT

ACTATTATCGTTACCAGTACTAGCTGGGGCAATCACTATACTTCTTACAGATCGAAACTT

AAATACAACATTTTTCGATCCGGCTGGTGGTGGAGACCCAGTATTATATCAACATTTATT

T--------------------------------------------

>916_Stylodrilus_heringianus_LL3

---AACCTTATATTTCATCTTAGGAGTTTGAGCTGGAATAGTAGGAGCAGGAATAAGACT

ATTAATTCGAGTTGAATTAACACAACCAGGATCATTTTTAGGAAGTGATCAATTATATAA

TACTATTGTTACGGCCCACGCCTTTATTATAATTTTCTTTATAGTTATACCTATATTTAT

TGGTGGCTTTGGTAACTGAATATTACCATTAATATTAGGAGCACCAGATATAGCATTCCC

ACGACTTAACAACCTAAGATTTTGGTTACTCCCGCCTTCTTTAACTTTATTAGTAGCATC

GGCTGCAGTTGAAAAGGGAGCCGGCACAGGATGAACAGTTTATCCACCTCTATCAAGAAA

TTTAGCTCATGCAGGTCCATCAGTAGACCTAGCAATCTTTTCACTACATCTAGCTGGGGC

ATCCTCCATTCTAGGAGCTATTAACTTTATTACTACGGTAATTAATATACGATGAAATGG

ACTCCGACTTGAACGAGTTCCATTATTTGTATGAGCAGTAACAATTACAGTTGTTCTATT

ACTATTATCGTTACCAGTACTAGCTGGGGCAATCACTATACTTCTTACAGATCGAAACTT

AAATACAACATTTTTCGATCCGGCTGGTGGTGGAGACCCAGTATTATATCAACATTTATT

T--------------------------------------------

>917_Stylodrilus_heringianus_LL3

---AACCTTATATTTCATCTTAGGAGTTTGAGCTGGAATAGTAGGAGCAGGAATAAGACT

ATTAATTCGAGTTGAATTAACACAACCAGGATCATTTTTAGGAAGTGATCAATTATATAA

TACTATTGTTACGGCTCACGCCTTTATTATAATTTTCTTTATAGTTATACCTATATTTAT

TGGTGGCTTTGGTAACTGAATATTACCATTAATATTAGGAGCACCAGATATGGCATTCCC

ACGACTTAACAACCTAAGATTTTGGTTACTCCCGCCTTCTTTAACTTTATTAGTAGCATC

GGCTGCAGTTGAAAAAGGAGCCGGCACAGGATGAACAGTTTATCCACCTCTATCAAGAAA

CTTAGCTCATGCAGGTCCATCAGTAGACCTAGCAATCTTTTCACTACATCTAGCTGGGGC

ATCCTCCATTCTAGGAGCTATTAACTTTATTACTACGGTAATTAATATACGATGAAATGG

ACTCCGACTTGAACGAGTTCCATTATTTGTATGAGCAGTAACAATTACAGTTGTTCTATT

ACTATTATCATTACCAGTACTAGCTGGAGCAATCACTATACTTCTTACAGATCGAAACTT

AAATACAACATTTTTCGATCCGGCTGGTGGTGGAGACCCAGTATTATATCAACATTTATT

T--------------------------------------------

>688_Globulidrilus_riparius_E11

---CACTATATATTTTATTTTAGGTATTTGAGCAGGTATACTTGGAGCAGCCATAAGACT

CTTAATTCGAATTGAATTAAGACAACCTGGATCTTTCCTTGGTAGAGATCAGTTATATAA

TACAATCGTAACAGCTCACGCCTTTCTAATAATTTTCTTCTTAGTTATACCTGTATTTAT

TGGTGGTTTCGGAAATTGATTATTACCTTTAATACTTGGAGCCCCAGATATAGCATTTCC

ACGACTAAATAATATAAGATTTTGACTTCTACCCCCATCATTACTACTTCTTGTATCATC

TGCTGCAGTTGAAAAAGGTGCAGGAACAGGGTGAACAGTATACCCCCCTCTTTCCAGTAA

TATAGCTCATGCAGGGCCTTCTGTTGATTTAGCTATTTTTTCTCTACATTTAGCAGGTGC

TTCTTCAATCTTAGGGGCAGTAAATTTTATTACTACCGTAATTAATATACGATGACAAGG

TCTCAGACTAGAACGAATTCCACTATTTGTATGAGCAGTAACTATTACTGTAGTACTTCT

CCTATTATCTCTCCCAGTTTTAGCAGGTGCCATTACTATACTTTTAACTGACCGAAATTT

AAACACATCATTTTTCGATCCTGCTGGAGGGGGTGATCCTGTATTATATCAACATTTATT

---------------------------------------------

>689_Globulidrilus_riparius_E11

---CACTATATATTTTATTTTAGGTATTTGAGCAGGTATACTTGGAGCAGCCATAAGACT

CTTAATTCGAATTGAATTAAGACAACCTGGATCTTTCCTTGGTAGAGATCAGTTATATAA

TACAATCGTAACAGCTCACGCCTTTCTAATAATTTTCTTCTTAGTTATACCTGTATTTAT

TGGTGGTTTCGGAAATTGATTATTACCTTTAATACTTGGAGCCCCAGATATAGCATTTCC

ACGACTAAATAATATAAGATTTTGACTTCTACCCCCATCATTACTACTTCTTGTATCATC

TGCTGCAGTTGAAAAAGGTGCAGGAACAGGGTGAACAGTATACCCCCCTCTTTCCAGTAA

TATAGCTCATGCAGGGCCTTCTGTTGATTTAGCTATTTTTTCTCTACATTTAGCAGGTGC

TTCTTCAATCTTAGGGGCAGTAAATTTTATTACTACCGTAATTAATATACGATGACAAGG

TCTCAGACTAGAACGAATTCCACTATTTGTATGAGCAGTAACTATTACTGTAGTACTTCT

CCTATTATCTCTCCCAGTTTTAGCAGGTGCCATTACTATACTTTTAACTGACCGAAATTT

AAACACATCATTTTTCGATCCTGCTGGAGGGGGTGATCCTGTATTATATCAACATTTATT

---------------------------------------------

>690_Globulidrilus_riparius_E11

---CACTATATATTTTATTCTAGGCATTTGAGCAGGTATACTTGGTGCTGCTATAAGACT

TTTAATTCGAATTGAATTAAGTCAACCAGGATCTTTTCTTGGTAGAGACCAACTATATAA

TACTATTGTAACAGCCCATGCCTTCCTAATAATCTTTTTCCTAGTTATACCTGTATTTAT

TGGGGGTTTTGGAAATTGACTACTCCCATTAATACTTGGGGCCCCAGATATAGCATTTCC

ACGACTAAATAATATAAGATTTTGACTTCTACCTCCTTCATTATTACTTCTTGTATCATC

TGCTGCTGTTGAAAAAGGCGCAGGAACAGGATGAACAGTATATCCTCCCCTTTCTAGAAA

TATAGCACATGCTGGACCCTCTGTAGACTTAGCCATTTTCTCCCTTCACTTAGCAGGTGC

TTCTTCAATTCTAGGAGCAGTAAATTTCATTACGACAGTAATTAATATACGATGACAAGG

TCTTAGATTAGAACGAATCCCTTTATTTGTATGAGCAGTAACAATCACTGTAGTACTTCT

TCTTCTATCTCTTCCAGTATTAGCTGGTGCCATTACTATACTTTTAACTGATCGAAATTT

AAATACATCATTTTTTGATCCAGCTGGAGGAGGTGATCCCGTTTTATACCAACACTTATT

T--------------------------------------------

>691_Potamothrix_bavaricus_T7

-----------ATATAATGTATGGCCTATGAACAGGCATGGTAGGAACTGGAACTAGACT

ACTAATTCGAATGGAACTTGCTCAACCAGGATCATTCCTTGGCAGAGATCAACTCTATAA

CACTCTCGTAACCGCACACGCATTTCTTATAATCTTTTTCCTTGTTATACCTGTATATAT

TGGTGCCTTCGGAAACTTCCTCGTCCCATTAATACTTGGCGCCCCTGATATAGCATTTCC

ACGGCTAAATAACTTAAGATTTTGACTAATACCCTCATCTGTAATTCTATGCGTGTCATC

AGCTGCTGTTGAAAAAGGAGCCGGTACTGGTTGAACAGTATACCCCCCATTAGCAAGAAA

TCTTGCTCATTCAGGCCCATCTGTCGACTTAGCTATTTTCTCTCTTCACTTAGCAGGTAT

CTCCTCTATTCTAGGGGCGATTAACTTTATTACCACTATAATTAATATGCGTTGAAAAGG

AATGCGCCTAGAACGAATTCCATTATTTGTATGAGCTACTATTATTACAGTAGTATTACT

CCTACTAACTCTTCCTGTCCTGGCCGGAGCTATTACTATACTTCTAACCGACCGTAATCT

AAATACCTCATTCTTCGACCCTGCTGGAGGGGGGTGACCCCTGTCCC-------------

---------------------------------------------

>693_Cernosvitoviella_minor_E8

---AACACTTTACTTTATTTTAGGTGTATGAGCAGGAATACTAGGAGCTGCTATAAGACT

TCTAATTCGAATTGAACTGAGACAACCTGGCTCATTCCTAGGGAGAGATCAATTATATAA

TACTATCGTTACAGCACATGCATTTTTAATAATTTTCTTCTTAGTAATACCAGTATTTAT

TGGAGGATTTGGGAACTGACTTTTACCTTTAATATTAGGAGCTCCAGACATGGCTTTTCC

TCGATTAAACAACATAAGATTTTGACTCCTACCACCATCATTAATATTATTAGTATCCTC

AGCAGCCGTAGAAAAAGGAGCCGGCACTGGATGAACAGTATATCCACCTCTTTCTAGAAA

TTTAGCACATGCTGGACCATCAGTAGATTTAGCAATTTTCTCTCTTCATCTTGCAGGAGC

TTCCTCAATTCTTGGGGCCGTAAACTTTATTACTACAGTAGTAAATATACGTTGACAAGG

CCTTCGACTAGAACGAATCCCCCTATTTGTATGAGCAGTAGTAATTACAGTAGTTCTACT

ACTCTTATCTCTACCAGTACTAGCAGGAGCAATTACTATACTATTAACAGACCGAAATTT

AAACACATCATTCTTTGACCCGGCAGGAGGGGGAGACCCTATTTTATACCAACACTTATT

C--------------------------------------------

>694_Globulidrilus_riparius_E9

---CACTATATATTTTATCCTAGGTGTTTGAGCCGGAATACTAGGAGCAGCAATAAGACT

ATTAATTCGAATTGAATTAAGTCAACCTGGTGCATTTCTTGGAAGAGACCAACTCTATAA

TACTATCGTAACAGCTCATGCATTTCTAATAATTTTCTTCTTAGTTATGCCTGTGTTCAT

TGGTGGATTTGGAAACTGATTACTCCCTTTAATGCTAGGAGCACCAGATATAGCCTTTCC

ACGTCTAAATAATATAAGATTTTGACTTCTTCCACCTTCACTACTTCTTCTTGTTTCATC

TGCAGCAGTAGAAAAAGGAGCTGGAACTGGTTGAACTGTATATCCCCCTCTATCAAGAAA

CATAGCTCACGCAGGACCATCCGTAGACCTAGCAATTTTTTCCCTTCATTTAGCAGGTGC

ATCATCAATTCTTGGAGCAGTAAATTTTATCACAACAGTAATTAACATACGATGACAAGG

ATTGAGATTAGAGCGAATTCCTTTATTTGTATGAGCTGTAACAATCACTGTAGTTCTTCT

TCTTCTATCTCTCCCTGTTCTGGCTGGTGCAATCACCATATTATTAACTGATCGAAATCT

AAACACTTCATTTTTTGACCCTGCTGGAGGAGGAGATNCAGTACTATATCAACACTTATT

T--------------------------------------------

>695_Globulidrilus_riparius_E11

---CACTATATATTTTATTTTAGGTATTTGAGCAGGTATACTTGGAGCAGCCATAAGACT

CTTAATTCGAATTGAATTAAGACAACCTGGATCTTTCCTTGGTAGAGATCAGTTATATAA

TACAATCGTAACAGCTCACGCCTTTCTAATAATTTTCTTCTTAGTTATACCTGTATTTAT

TGGTGGTTTCGGAAATTGATTATTACCTTTAATACTTGGAGCCCCAGATATAGCATTTCC

ACGACTAAATAATATAAGATTTTGACTTCTACCCCCATCATTACTACTTCTTGTATCATC

TGCTGCAGTTGAAAAAGGTGCAGGAACAGGGTGAACAGTATACCCCCCTCTTTCCAGTAA

TATAGCTCATGCAGGGCCTTCTGTTGATTTAGCTATTTTTTCTCTACATTTAGCAGGTGC

TTCTTCAATCTTAGGGGCAGTAAATTTTATTACTACCGTAATTAATATACGATGACAAGG

TCTCAGACTAGAACGAATTCCACTATTTGTATGAGCAGTAACTATTACTGTAGTACTTCT

CCTATTATCTCTCCCAGTTTTAGCAGGTGCCATTACTATACTTTTAACTGACCGAAATTT

AAACACATCATTTTTCGATCCTGCTGGAGGGGGTGATCCTGTATTATATCAACATTTATT

---------------------------------------------

>696_Globulidrilus_riparius_E9

---CACTATATATTTTATCCTGGGTGTTTGAGCCGGAATACTAGGAGCAGCAATAAGACT

ATTAATTCGAATTGAATTAAGTCAACCTGGCGCATTCCTTGGAAGAGACCAACTCTATAA

TACTATCGTAACAGCACATGCATTTCTAATAATTTTCTTCTTAGTTATGCCTGTGTTCAT

TGGTGGATTTGGAAACTGATTACTCCCTTTAATGCTAGGAGCACCAGATATAGCCTTTCC

ACGTCTAAATAATATAAGATTTTGACTTCTTCCACCTTCACTACTTCTTCTTGTATCATC

TGCAGCAGTAGAAAAAGGAGCTGGAACTGGTTGAACCGTATACCCCCCTCTATCGAGAAA

CATAGCTCATGCAGGACCATCTGTAGACCTAGCAATTTTTTCTCTTCATTTAGCAGGTGC

ATCATCAATTCTTGGGGCAGTAAATTTTATCACAACAGTAATTAACATACGATGACAAGG

ATTAAGATTAGAGCGAATTCCTTTATTTGTATGAGCTGTAACAATCACTGTAGTTCTTCT

TCTTCTATCTCTCCCTGTTCTAGCTGGCGCAATCACCATATTATTAACTGATCGAAATCT

AAACACTTCATTTTTTGACCCTGCTGGAGGAGGAGATCCAGTATTATATCAACACTTATT

T--------------------------------------------

>697_Globulidrilus_riparius_E11

---CACTATATATTTTATTCTAGGTATTTGAGCAGGTATACTTGGAGCTGCTATAAGGCT

TTTAATTCGAATCGAATTAAGACAACCTGGATCCTTTCTTGGTAGAGATCAGCTTTATAA

TACTATTGTAACAGCTCATGCCTTTCTAATAATCTTCTTCTTAGTTATACCTGTATTTAT

TGGGGGTTTCGGAAATTGATTATTACCATTAATACTAGGGGCTCCAGATATAGCATTTCC

ACGTTTAAATAATATAAGATTTTGACTTCTACCTCCATCATTATTATTACTTGTATCATC

CGCTGCAGTTGAAAAAGGTGCAGGTACAGGATGAACAGTATATCCACCTCTTTCTAGAAA

TATGGCACATGCAGGACCTTCTGTAGATTTAGCTATTTTTTCTCTTCATCTAGCAGGTGC

TTCATCAATTTTAGGAGCAGTAAACTTTATTACAACAGTAATTAATATACGATGACAAGG

TCTTAGATTAGAACGAATCCCTCTATTTGTATGGGCAGTAACCATTACTGTAGTACTTCT

TCTATTATCCCTTCCAGTTTTAGCCGGCGCTATTACTATACTTTTAACTGATCGAAATTT

AAATACATCATTTTTCGATCCTGCTGGAGGCGGGGACCCTGTTTTATATCAACACTTATT

T--------------------------------------------

>698_Globulidrilus_riparius_E9

---CACTATATATTTTATCCTAGGTGTTTGAGCCGGAATACTAGGAGCAGCAATAAGACT

ATTAATTCGAATTGAATTAAGTCAACCTGGTGCATTCCTTGGAAGAGACCAACTCTATAA

TACTATCGTAACAGCACATGCATTTCTAATAATTTTCTTCTTAGTTATGCCTGTGTTCAT

TGGTGGATTTGGAAACTGATTACTCCCTTTAATGCTAGGAGCACCAGATATAGCCTTTCC

ACGTCTAAATAATATAAGATTTTGACTTCTTCCACCTTCACTACTTCTTCTTGTATCATC

TGCAGCAGTAGAAAAAGGAGCTGGAACTGGTTGAACCGTATACCCCCCTCTATCGAGAAA

CATAGCTCATGCAGGACCATCTGTAGACCTAGCAATTTTTTCTCTTCATTTAGCAGGTGC

ATCATCAATTCTTGGGGCAGTAAATTTTATCACAACAGTAATTAACATACGATGACAAGG

ATTAAGATTAGAACGAATTCCTTTATTTGTATGAGCTGTAACAATCACTGTAGTTCTTCT

TCTTCTATCTCTCCCTGTTCTAGCTGGCGCAATCACCATATTATTAACTGATCGAAATCT

AAACACTTCATTTTTTGACCCTGCTGGAGGAGGAGATCCAGTATTATATCAACACTTATT

---------------------------------------------

>699_Globulidrilus_riparius_E11

---CACTATATATTTTATTTTAGGTATTTGAGCAGGTATACTTGGAGCAGCCATAAGACT

CTTAATTCGAATTGAATTAAGACAACCTGGATCTTTCCTTGGTAGAGATCAGTTATATAA

TACAATCGTAACAGCTCACGCCTTTCTAATAATTTTCTTCTTAGTTATACCTGTATTTAT

TGGTGGTTTCGGAAATTGATTATTACCTTTAATACTTGGAGCCCCAGATATAGCATTTCC

ACGACTAAATAATATAAGATTTTGACTTCTACCCCCATCATTACTACTTCTTGTATCATC

TGCTGCAGTTGAAAAAGGTGCAGGAACAGGGTGAACAGTATACCCCCCTCTTTCCAGTAA

TATAGCTCATGCAGGGCCTTCTGTTGATTTAGCTATTTTTTCTCTACATTTAGCAGGTGC

TTCTTCAATCTTAGGGGCAGTAAATTTTATTACTACCGTAATTAATATACGATGACAAGG

TCTCAGACTAGAACGAATTCCACTATTTGTATGAGCAGTAACTATTACTGTAGTACTTCT

CCTATTATCTCTCCCAGTTTTAGCAGGTGCCATTACTATACTTTTAACTGACCGAAATTT

AAACACATCATTTTTCGATCCTGCTGGAGGGGGTGATCCTGTATTATATCAACATTTATT

---------------------------------------------

>700_Globulidrilus_riparius_E11

---CACTATATATTTTATTTTAGGTATTTGAGCAGGTATACTTGGAGCAGCCATAAGACT

CTTAATTCGAATTGAATTAAGACAACCTGGATCTTTCCTTGGTAGAGATCAGTTATATAA

TACAATCGTAACAGCTCACGCCTTTCTAATAATTTTCTTCTTAGTTATACCTGTATTTAT

TGGTGGTTTCGGAAATTGATTATTACCTTTAATACTTGGAGCCCCAGATATAGCATTTCC

ACGACTAAATAATATAAGATTTTGACTTCTACCCCCATCATTACTACTTCTTGTATCATC

TGCTGCAGTTGAAAAAGGTGCAGGAACAGGGTGAACAGTATACCCCCCTCTTTCCAGTAA

TATAGCTCATGCAGGGCCTTCTGTTGATTTAGCTATTTTTTCTCTACATTTAGCAGGTGC

TTCTTCAATCTTAGGGGCAGTAAATTTTATTACTACCGTAATTAATATACGATGACAAGG

TCTCAGACTAGAACGAATTCCACTATTTGTATGAGCAGTAACTATTACTGTAGTACTTCT

CCTATTATCTCTCCCAGTTTTAGCAGGTGCCATTACTATACTTTTAACTGACCGAAATTT

AAACACATCATTTTTCGATCCTGCTGGAGGGGGTGATCCTG-------------------

---------------------------------------------

>701_Globulidrilus_riparius_E11

---CACTATATATTTTATTCTAGGCATTTGAGCAGGTATACTTGGTGCTGCTATAAGACT

TTTAATTCGAATTGAATTAAGTCAACCAGGATCTTTTCTTGGTAGAGACCAACTATATAA

TACTATTGTAACAGCCCATGCCTTCCTAATAATCTTTTTCCTAGTTATACCTGTATTTAT

TGGGGGTTTTGGAAATTGACTACTCCCATTAATACTTGGGGCCCCAGATATAGCATTTCC

ACGACTAAATAATATAAGATTTTGACTTCTACCTCCTTCATTATTACTTCTTGTATCATC

TGCTGCTGTTGAAAAAGGCGCAGGAACAGGATGAACAGTATATCCTCCCCTTTCTAGAAA

TATAGCACATGCTGGACCCTCTGTAGACTTAGCCATTTTCTCCCTTCACTTAGCAGGTGC

TTCTTCAATTCTAGGAGCAGTAAATTTCATTACGACAGTAATTAATATACGATGACAAGG

TCTTAGATTAGAACGAATCCCTTTATTTGTATGAGCAGTAACAATCACTGTAGTACTTCT

TCTTCTATCTCTTCCAGTATTAGCTGGTGCCATTACTATACTTTTAACTGATCGAAATTT

AAATACATCATTTTTTGATCCAGCTGGAGGAGGTGATCCCGTTTTATACCAACACTTATT

---------------------------------------------

>702_Globulidrilus_riparius_E11

---CACTATATATTTTATTTTAGGTATTTGAGCAGGTATACTTGGAGCAGCCATAAGACT

CTTAATTCGAATTGAATTAAGACAACCTGGATCTTTCCTTGGTAGAGATCAGTTATATAA

TACAATCGTAACAGCTCACGCCTTTCTAATAATTTTCTTCTTAGTTATACCTGTATTTAT

TGGTGGTTTCGGAAATTGATTATTACCTTTAATACTTGGAGCCCCAGATATAGCATTTCC

ACGACTAAATAATATAAGATTTTGACTTCTACCCCCATCATTACTACTTCTTGTATCATC

TGCTGCAGTTGAAAAAGGTGCAGGAACAGGGTGAACAGTATACCCCCCTCTTTCCAGTAA

TATAGCTCATGCAGGGCCTTCTGTTGATTTAGCTATTTTTTCTCTACATTTAGCAGGTGC

TTCTTCAATCTTAGGGGCAGTAAATTTTATTACTACCGTAATTAATATACGATGACAAGG

TCTCAGACTAGAACGAATTCCACTATTTGTATGAGCAGTAACTATTACTGTAGTACTTCT

CCTATTATCTCTCCCAGTTTTAGCAGGTGCCATTACTATACTTTTAACTGACCGAAATTT

AAACACATCATTTTTCGATCCTGCTGGAGGGGGTGATCCTGTATTATATCAACATTTATT

T--------------------------------------------

>703_Globulidrilus_riparius_E10

---CACAATATATTTTATTTTAGGTGTTTGAGCAGGTATACTAGGTGCAGCTATAAGATT

ACTAATTCGTATTGAATTAAGCCAACCAGGATCATTTCTTGGTAGAGATCAATTATACAA

TACCATTGTAACCGCTCATGCCTTCTTAATAATCTTCTTCTTAGTAATACCTGTATTTAT

TGGAGGATTTGGAAACTGACTACTTCCATTAATACTAGGTGCCCCTGACATAGCATTCCC

GCGACTAAATAATATAAGATTCTGACTCCTTCCTCCATCACTATTACTACTAGTATCTTC

AGCCGCTGTTGAAAAAGGTGCAGGAACAGGATGAACTGTATACCCACCCCTTTCAAGAAA

TATAGCTCATGCAGGCCCATCTGTAGATCTAGCTATTTTCTCTCTTCATTTAGCTGGTGC

GTCTTCAATTCTAGGTGCTGTAAATTTCATCACTACAGTAATTAATATACGATGACAAGG

TCTTAGATTAGAACGAATCCCTTTATTTGTTTGAGCAGTAACTATTACTGTAGTTCTTCT

TCTACTATCTCTACCAGTTTTAGCAGGAGCCATTACTATACTTCTAACTGACCGTAATTT

AAACACCTCATTCTTTGATCCTGCTGGAGGTGGAG-------------------------

---------------------------------------------

>704_Globulidrilus_riparius_E11

---CACTATATATTTTATTTTAGGTATTTGAGCAGGTATACTTGGAGCAGCCATAAGACT

CTTAATTCGAATTGAATTAAGACAACCTGGATCTTTCCTTGGTAGAGATCAGTTATATAA

TACAATCGTAACAGCTCACGCCTTTCTAATAATTTTCTTCTTAGTTATACCTGTATTTAT

TGGTGGTTTCGGAAATTGATTATTACCTTTAATACTTGGAGCCCCAGATATAGCATTTCC

ACGACTAAATAATATAAGATTTTGACTTCTACCCCCATCATTACTACTTCTTGTATCATC

TGCTGCAGTTGAAAAAGGTGCAGGAACAGGGTGAACAGTATACCCCCCTCTTTCCAGTAA

TATAGCTCATGCAGGGCCTTCTGTTGATTTAGCTATTTTTTCTCTACATTTAGCAGGTGC

TTCTTCAATCTTAGGGGCAGTAAATTTTATTACTACCGTAATTAATATACGATGACAAGG

TCTCAGACTAGAACGAATTCCACTATTTGTATGAGCAGTAACTATTACTGTAGTACTTCT

CCTATTATCTCTCCCAGTTTTAGCAGGTGCCATTACTATACTTTTAACTGACCGAAATTT

AAACACATCATTTTTCGATCCTGCTGGAGGGGGTGATCCTGTATATA-------------

---------------------------------------------

>706_Globulidrilus_riparius_E9

---CACTATATATTTTATCCTAGGTGTTTGAGCCGGAATACTAGGAGCAGCAATAAGACT

ATTAATTCGAATTGAATTAAGTCAACCTGGTGCATTCCTTGGAAGAGATCAACTCTATAA

TACTATCGTAACAGCTCATGCATTTCTAATAATTTTCTTCTTAGTTATGCCTGTATTCAT

TGGTGGATTTGGAAACTGATTACTGCCTTTAATACTAGGGGCACCAGATATAGCCTTTCC

ACGTCTAAATAACATAAGATTTTGACTTCTTCCACCTTCACTACTTCTTCTTGTTTCATC

TGCAGCAGTAGAAAAAGGAGCTGGAACTGGTTGAACCGTATATCCTCCTCTATCAAGAAA

CATAGCTCATGCAGGACCATCTGTAGATCTAGCAATTTTTTCTCTTCATTTAGCAGGTGC

ATCATCAATTCTTGGAGCAGTAAATTTTATCACAACAGTAATTAACATACGATGACAAGG

ATTAAGATTAGAACGAATTCCTTTATTTGTATGAGCTGTAACAATCACTGTAGTTCTTCT

TCTTCTATCTCTCCCTGTTCTGGCTGGTGCAATCACCATATTATTAACTGATCGAAATCT

AAACACTTCATTTTTTGATCCTGCTGGTGGAGGAGA------------------------

---------------------------------------------

>707_Globulidrilus_riparius_E11

---CACTATATATTTTATTTTAGGTATTTGAGCAGGTATACTTGGAGCAGCCATAAGACT

CTTAATTCGAATTGAATTAAGACAACCTGGATCTTTCCTTGGTAGAGATCAGTTATATAA

TACAATCGTAACAGCTCACGCCTTTCTAATAATTTTCTTCTTAGTTATACCTGTATTTAT

TGGTGGTTTCGGAAATTGATTATTACCTTTAATACTTGGAGCCCCAGATATAGCATTTCC

ACGACTAAATAATATAAGATTTTGACTTCTACCCCCATCATTACTACTTCTTGTATCATC

TGCTGCAGTTGAAAAAGGTGCAGGAACAGGGTGAACAGTATACCCCCCTCTTTCCAGTAA

TATAGCTCATGCAGGGCCTTCTGTTGATTTAGCTATTTTTTCTCTACATTTAGCAGGTGC

TTCTTCAATCTTAGGGGCAGTAAATTTTATTACTACCGTAATTAATATACGATGACAAGG

TCTCAGACTAGAACGAATTCCACTATTTGTATGAGCAGTAACTATTACTGTAGTACTTCT

CCTATTATCTCTCCCAGTTTTAGCAGGTGCCATTACTATACTTTTAACTGACCGAAATTT

AAACACATCATTTTTCGATCCTGCTGGA--------------------------------

---------------------------------------------

>708_Globulidrilus_riparius_E11

---CACTATATATTTTATTTTAGGTATTTGAGCAGGTATACTTGGAGCAGCCATAAGACT

CTTAATTCGAATTGAATTAAGACAACCTGGATCTTTCCTTGGTAGAGATCAGTTATATAA

TACAATCGTAACAGCTCACGCCTTTCTAATAATTTTCTTCTTAGTTATACCTGTATTTAT

TGGTGGTTTCGGAAATTGATTATTACCTTTAATACTTGGAGCCCCAGATATAGCATTTCC

ACGACTAAATAATATAAGATTTTGACTTCTACCCCCATCATTACTACTTCTTGTATCATC

TGCTGCAGTTGAAAAAGGTGCAGGAACAGGGTGAACAGTATACCCCCCTCTTTCCAGTAA

TATAGCTCATGCAGGGCCTTCTGTTGATTTAGCTATTTTTTCTCTACATTTAGCAGGTGC

TTCTTCAATCTTAGGGGCAGTAAATTTTATTACTACCGTAATTAATATACGATGACAAGG

TCTCAGACTAGAACGAATTCCACTATTTGTATGAGCAGTAACTATTACTGTAGTACTTCT

CCTATTATCTCTCCCAGTTTTAGCAGGTGCCATTACTATACTTTTAACTGACCGAAATTT

AAACACATCATTTTTCGATCCTGCTGGAGGGGGTGATCC---------------------

---------------------------------------------

>709_Globulidrilus_riparius_E11

---CACTATATATTTTATTTTAGGTATTTGAGCAGGTATACTTGGAGCAGCCATAAGACT

CTTAATTCGAATTGAATTAAGACAACCTGGATCTTTGCTTGGTAGAGATCAGTTATATAA

TACAATCGTAACAGCTCACGCCTTTCTAATAATTTTCTTCTTAGTTATACCTGTATTTAT

TGGTGGTTTCGGAAATTGATTATTACCTTTAATACTTGGAGCCCCAGATATAGCATTTCC

ACGACTAAATAATATAAGATTTTGACTTCTACCCCCATCATTACTACTTCTTGTATCATC

TGCTGCAGTTGAAAAAGGTGCAGGAACAGGGTGAACAGTATACCCCCCTCTTTCCAGTAA

TATAGCTCATGCAGGGCCTTNGNNNGANNNNGCTATTTTTNCTCTACATTTAGCAGGTGC

TTCTTCAATCTTAGGGGCAGTAAATTTTTTTACTACCGTAATTAATATACGATGACAAGG

TCTCAGACTAGAACGAATTCCACTATTTGTATGAGCAGTAACTATTACTGTAGTACTTCT

CCTATTATCTCTCCCAG-------------------------------------------

------------------------------------------------------------

---------------------------------------------

>710_Globulidrilus_riparius_E11

---CACTATATATTTTATTTTAGGTATTTGAGCAGGTATACTTGGAGCAGCCATAAGACT

CTTAATTCGAATTGAATTAAGACAACCTGGATCTTTCCTTGGTAGAGATCAGTTATATAA

TACAATCGTAACAGCTCACGCCTTTCTAATAATTTTCTTCTTAGTTATACCTGTATTTAT

TGGTGGTTTCGGAAATTGATTATTACCTTTAATACTTGGAGCCCCAGATATAGCATTTCC

ACGACTAAATAATATAAGATTTTGACTTCTACCCCCATCATTACTACTTCTTGTATCATC

TGCTGCAGTTGAAAAAGGTGCAGGAACAGGGTGAACAGTATACCCCCCTCTTTCCAGTAA

TATAGCTCATGCAGGGCCTTCTGTTGATTTAGCTATTTTTTCTCTACATTTAGCAGGTGC

TTCTTCAATCTTAGGGGCAGTAAATTTTATTACTACCGTAATTAATATACGATGACAAGG

TCTCAGACTAGAACGAATTCCACTATTTGTATGAGCAGTAACTATTACTGTAGTACTTCT

CCTATTATCTCTCCCAGTTTTAGCAGGTGCCATTACTATACTTTTAACTGACCGAAATTT

AAACACATCATTTTTCGATCCTGCTGGAGGGGGTGATCCTG-------------------

---------------------------------------------

>711_Globulidrilus_riparius_E11

---CACTATATATTTTATTTTAGGTATTTGAGCAGGTATACTTGGAGCAGCCATAAGACT

CTTAATTCGAATTGAATTAAGACAACCTGGATCTTTCCTTGGTAGAGATCAGTTATATAA

TACAATCGTAACAGCTCACGCCTTTCTAATAATTTTCTTCTTAGTTATACCTGTATTTAT

TGGTGGTTTCGGAAATTGATTATTACCTTTAATACTTGGAGCCCCAGATATAGCATTTCC

ACGACTAAATAATATAAGATTTTGACTTCTACCCCCATCATTACTACTTCTTGTATCATC

TGCTGCAGTTGAAAAAGGTGCAGGAACAGGGTGAACAGTATACCCCCCTCTTTCCAGTAA

TATAGCTCATGCAGGGCCTTNTGTTGATTTAGCTATTTTTTCTCTACATTTAGCAGGTGC

TTCTTCAATCTTAGGGGCAGTAAATTTTATTACTACCGTAATTAATATACGATGACAAGG

TCTCAGACTAGAACGAATTCCACTATTTGTATGAGCAGTAACTATTACTGTAGTACTTCT

CCTATTATCTCTCCCAGTTTTAGCAGGTGCCATTACTATACTTTTAACTGACCGAAATTT

AAACACATCATTTTTCGATCCTGCTGGAGGG-----------------------------

---------------------------------------------

>712_Globulidrilus_riparius_E11

---CACTATATATTTTATTTTAGGTATTTGAGCAGGTATACTTGGAGCAGCCATAAGACT

CTTAATTCGAATTGAATTAAGACAACCTGGATCTTTCCTTGGTAGAGATCAGTTATATAA

TACAATCGTAACAGCTCACGCCTTTCTAATAATTTTCTTCTTAGTTATACCTGTATTTAT

TGGTGGTTTCGGAAATTGATTATTACCTTTAATACTTGGAGCCCCAGATATAGCATTTCC

ACGACTAAATAATATAAGATTTTGACTTCTACCCCCATCATTACTACTTCTTGTATCATC

TGCTGCAGTTGAAAAAGGTGCAGGAACAGGGTGAACAGTATACCCCCCTCTTTCCAGTAA

TATAGCTCATGCAGGGCCTNCTGTNGATTTAGCTATTTTTTCTCTACATTTAGCAGGTGC

TTCTTCAATCTTAGGGGCAGTAAATTTTATTACTACCGTAATTAATATACGATGACAAGG

TCTCAGACTAGAACGAATTCCACTATTTGTATGAGCAGTAACTATTACTGTAGTACTTCT

CCTATTATCTCTCCCAGTTTTAGCAGGTGCCATTACTATACTTTTAACTGACCGAAATTT

AAACACATCATTTTTCGATCCTGCTGGAGGGGGT--------------------------

---------------------------------------------

>713_Globulidrilus_riparius_E11

---CACTATATATTTTATTTTAGGTATTTGAGCAGGTATACTTGGAGCAGCCATAAGACT

CTTAATTCGAATTGAATTAAGACAACCTGGATCTTTCCTTGGTAGAGATCAGTTATATAA

TACAATCGTAACAGCTCACGCCTTTCTAATAATTTTCTTCTTAGTTATACCTGTATTTAT

TGGTGGTTTCGGAAATTGATTATTACCTTTAATACTTGGAGCCCCAGATATAGCATTTCC

ACGACTAAATAATATAAGATTTTGACTTCTACCCCCATCATTACTACTTCTTGTATCATC

TGCTGCAGTTGAAAAAGGTGCAGGAACAGGGTGAACAGTATACCCCCCTCTTTCCAGTAA

TATAGCTCATGCAGGGCCTTCTGTTGATTTAGCTATTTTTTCTCTACATTTAGCAGGTGC

TTCTTCAATCTTAGGGGCAGTAAATTTTATTACTACCGTAATTAATATACGATGACAAGG

TCTCAGACTAGAACGAATTCCACTATTTGTATGAGCAGTAACTATTACTGTAGTACTTCT

CCTATTATCTCTCCCAGTTTTAGCAGGTGCCATTACTATACTTTTAACTGACCGAAATTT

AAACACATCATTTTTCGATCCTGCTGGAGGGGGTGATCCTGTATTT--------------

---------------------------------------------

>715_Potamothrix_bavaricus_T7

---ACTCCTTTATATAATGTATGGCCTATGAACAGGCATGGTAGGAACTGGAACTAGACT

ACTAATTCGAATGGAACTTGCTCAACCAGGATCATTCCTTGGCAGAGATCAACTCTATAA

CACTCTCGTAACCGCACACGCATTTCTTATAATCTTTTTCCTTGTTATACCTGTATATAT

TGGTGCCTTCGGAAACTTCCTCGTCCCATTAATACTTGGCGCCCCTGATATAGCATTTCC

ACGGCTAAATAACTTAAGATTTTGACTAATACCCTCATCTGTAATTCTATGCGTGTCATC

AGCTGCTGTTGAAAAAGGAGCCGGTACTGGTTGAACAGTATACCCCCCATTAGCAAGAAA

TCTTGCTCATTCAGGCCCATCTGTCGACTTAGCTATTTTCTCTCTTCACTTAGCAGGTAT

CTCCTCTATTCTAGGGGCGATTAACTTTATTACCACTATAATTAATATGCGTTGAAAAGG

AATGCGCCTAGAACGAATTCCATTATTTGTATGAGCTACTATTATTACAGTAGTATTACT

CCTACTAACTCTTCCTGTCCTGGCCGGAGCTATTACTATACTTCTAACCG----------

------------------------------------------------------------

---------------------------------------------

>716_Globulidrilus_riparius_E11

---CACTATATATTTTATTTTAGGTATTTGAGCAGGTATACTTGGAGCAGCCATAAGACT

CTTAATTCGAATTGAATTAAGACAACCTGGATCTTTCCTTGGTAGAGATCAGTTATATAA

TACAATCGTAACAGCTCACGCCTTTCTAATAATTTTCTTCTTAGTTATACCTGTATTTAT

TGGTGGTTTCGGAAATTGATTATTACCTTTAATACTTGGAGCCCCAGATATAGCATTTCC

ACGACTAAATAATATAAGATTTTGACTTCTACCCCCATCATTACTACTTCTTGTATCATC

TGCTGCAGTTGAAAAAGGTGCAGGAACAGGGTGAACAGTATACCCCCCTCTTTCCAGTAA

TATAGCTCATGCAGGGCCTTCTGTTGATTTAGCTATTTTTTCTCTACATTTAGCAGGTGC

TTCTTCAATCTTAGGGGCAGTAAATTTTATTNCTACCGTAATTAATATACGATGACAAGG

TCTCAGACTAGAACGAATTCCACTATTTGTATGAGCAGTAACTATTACTGTAGTACTTCT

CCTATTATCTCTCCCAGTTTTAGCAGGTGCCATTACTATACTTTTAACTGACCGAAATTT

AAACACATCATTTTT---------------------------------------------

---------------------------------------------

>717_Globulidrilus_riparius_E11

---CACTATATATTTTATTTTAGGTATTTGAGCAGGTATACTTGGAGCAGCCATAAGACT

CTTAATTCGAATTGAATTAAGACAACCTGGATCTTTCCTTGGTAGAGATCAGTTATATAA

TACAATCGTAACAGCTCACGCCTTTCTAATAATTTTCTTCTTAGTTATACCTGTATTTAT

TGGTGGTTTCGGAAATTGATTATTACCTTTAATACTTGGAGCCCCAGATATAGCATTTCC

ACGACTAAATAATATAAGATTTTGACTTCTACCCCCATCATTACTACTTCTTGTATCATC

TGCTGCAGTTGAAAAAGGTGCAGGAACAGGGTGAACAGTATACCCCCCTCTTTCCAGTAA

TATAGCTCATGCAGGGCCTTCTGTTGATTTAGCTATTTTTTCTCTACATTTAGCAGGTGC

TTCTTCAATCTTAGGGGCAGTAAATTTTATTACTACCGTAATTAATATACGATGACAAGG

TCTCAGACTAGAACGAATTCCACTATTTGTATGAGCAGTAACTATTACTGTAGTACTTCT

CCTATTATCTCTCCCAGTTTTAGCAGGTGCCATTACTATACTTTTAACTGACCGAAATTT

AAACACATCATTTTTCGATCCTGCTGGAGGGGGTGATC----------------------

---------------------------------------------

>678_Lumbriculidae_sp_LL3

----------------------------TGAGCTGGAATAGTAGGAGCAGGAATAAGACT

ATTAATTCGAGTTGAATTAACACAACCAGGATCATTTTTAGGAAGTGATCAATTATATAA

TACTATTGTTACGGCCCACGCCTTTATTATAATTTTCTTTATAGTTATACCTATATTTAT

TGGTGGATTTGGTAACTGAATATTACCATTAATATTAGGAGCACCAGATATAGCATTCCC

ACGACTTAACAACCTAAGATTTTGGTTACTCCCGCCTTCTTTAACTTTATTAGTAGCATC

GGCTGCAGTTGAAAAGGGAGCCGGCACAGGATGAACAGTTTATCCACCTCTATCAAGAAA

TTTAGCTCATGCAGGTCCATCAGTAGACCTAGCAATCTTTTCACTACATCTAGCTGGGGC

ATCCTCCATTCTAGGAGCTATTAACTTTATTACTACGGTAATTAATATACGATGAAATGG

ACTCCGACTTGAACGAGTTCCATTATTTGTATGAGCAGTAACAATTACAGTTGTTCTATT

ACTATTATCGTTACCAGTACTAGCTGGGGCAATCACTATACTTCTTACAGATCGAAACTT

AAATACAACATTTTTCGATCCCGCTGGTGGTGGAGA------------------------

---------------------------------------------

>679_Stylodrilus_heringianus_LL3

-----------------TCTTAGGAGTTTGAGCTGGAATAGTAGGAGCAGGAATAAGACT

ATTAATTCGAGTTGAATTAACACAACCAGGATCATTTTTAGGAAGTGATCAATTATATAA

TACTATTGTTACGGCCCACGCCTTTATTATAATTTTCTTTATAGTTATACCTATATTTAT

TGGTGGATTTGGTAACTGAATATTACCATTAATATTAGGAGCACCAGATATAGCATTCCC

ACGACTTAACAACCTAAGATTTTGGTTACTCCCGCCTTCTTTAACTTTATTAGTAGCATC

GGCTGCAGTTGAAAAGGGAGCCGGCACAGGATGAACAGTTTATCCACCTCTATCAAGAAA

TTTAGCTCATGCAGGTCCATCAGTAGACCTAGCAATCTTTTCACTACATCTAGCTGGGGC

ATCCTCCATTCTAGGAGCTATTAACTTTATTACTACGGTAATTAATATACGATGAAATGG

ACTCCGACTTGAACGAGTTCCATTATTTGTATGAGCAGTAACAATTACAGTTGTTCTATT

ACTATTATCGTTACCAGTACTAGCTGGGGCAATCACTATACTTCTTACAGATCGAAACTT

AAATACAACATTTTTCGATCCCGCTGGTGGTGGAGACCCAGTATTATATCAACATTTATT

---------------------------------------------

>680_Lumbriculidae_sp_LL3

---AACCTTATATTTCATCTTAGGAGTTTGAGCTGGAATAGTAGGAGCAGGAATAAGACT

ATTAATTCGAGTTGAATTAACACAACCAGGATCATTTTTAGGAAGTGATCAATTATATAA

TACTATTGTTACGGCCCACGCCTTTATTATAATTTTCTTTATAGTTATACCTATATTTAT

TGGTGGATTTGGTAACTGAATATTACCATTAATATTAGGAGCACCAGATATAGCATTCCC

ACGACTTAACAACCTAAGATTTTGGTTACTCCCGCCTTCTTTAACTTTATTAGTAGCATC

GGCTGCAGTTGAAAAGGGAGCCGGCACAGGATGAACAGTTTATCCACCTCTATCAAGAAA

TTTAGCTCATGCAGGTCCATCAGTAGACCTAGCAATCTTTTCACTACATCTAGCTGGGGC

ATCCTCCATTCTAGGAGCTATTAACTTTATTACTACGGTAATTAATATACGATGAAATGG

ACTCCGACTTGAACGAGTTCCATTATTTGTATGAGCAGTAACAATTACAGTTGTTCTATT

ACTATTATCGTTACCAGTACTAGCTGGGGCAATCACTATACTTCTTACAGATCGAAACTT

AAATACAACATTTTTCGATGACGCTGGTGGTGGAGACCCAGTATTAT-------------

----------------------------------------------

>681_Lumbriculidae_sp_LL3

----------------------GGAGTTTGAGCTGGAATAGTAGGAGCAGGAATAAGACT

ATTAATTCGAGTTGAATTAACACAACCAGGATCATTTTTAGGAAGTGATCAATTATATAA

TACTATTGTTACGGCTCACGCCTTTATTATAATTTTCTTTATAGTTATACCTATATTTAT

TGGTGGCTTTGGTAACTGAATATTACCATTAATATTAGGAGCACCAGATATGGCATTCCC

ACGACTTAACAACCTAAGATTTTGGTTACTCCCGCCTTCTTTAACTTTATTAGTAGCATC

GGCTGCAGTTGAAAAAGGAGCCGGCACAGGATGAACAGTTTATCCACCTCTATCAAGAAA

CTTAGCTCATGCAGGTCCATCAGTAGACCTAGCAATCTTTTCACTACATCTAGCTGGGGC

ATCCTCCATTCTAGGAGCTATTAACTTTATTACTACGGTAATTAATATACGATGAAATGG

ACTCCGACTTGAACGAGTTCCATTATTTGTATGAGCAGTAACAATTACAGTTGTTCTATT

ACTATTATCATTACCAGTACTAGCTGGAGCAATCACTATACTTCTTACAGATCGAAACTT

AAATACAACATTTTTCGATCCGGCTGGTGGTGGAGACCCAGTATTATATCAACATTTATT

T--------------------------------------------

>682_Lumbriculidae_LL3

---GACCTTATATTTCATCTTAGGAGTTTGAGCTGGAATAGTGGGAGCAGGCATAAGACT

ATTAATTCGAGTTGAATTAACACAACCTGGGTCATTTTTAGGAAGTGACCAACTATACAA

TACTATTGTTACGGCTCACGCCTTTATTATAATTTTCTTTATAGTCATACCTATATTTAT

TGGTGGTTTTGGTAACTGAATACTACCATTAATATTAGGAGCACCAGATATAGCATTCCC

ACGGCTTAACAACCTAAGATTTTGGTTACTTCCACCATCTTTAACTTTATTAGTAGCATC

CGCTGCAGTTGAAAAAGGAGCAGGTACAGGATGAACAGTTTACCCGCCTCTATCAAGAAA

TTTAGCCCATGCAGGTCCATCAGTAGATCTAGCAATCTTTTCACTTCATCTAGCCGGAGC

ATCCTCCATTCTAGGAGCTATTAACTTTATTACAACAGTAATTAATATACGATGAAATGG

ACTACGACTTGAACGAGTTCCATTATTTGTATGAGCAGTAACAATTACAGTAGTACTCTT

ACTATTATCTTTACCAGTACTAGCTGGGGCAATCACTATACTTCTTACAGATCGAAACTT

AAATACAACATTTTTTGATCCAGCGGGAGGTGGAGACCCAGTACTA--------------

---------------------------------------------

>683_Lumbriculidae_sp_LL3

-----------------TCTTAGGAGTTTGAGCTGGAATAGTAGGAGCAGGAATAAGACT

ATTAATTCGAGTTGAATTAACACAACCAGGATCATTTTTAGGAAGTGATCAATTATATAA

TACTATTGTTACGGCTCACGCCTTTATTATAATTTTCTTTATAGTTATACCTATATTTAT

TGGTGGCTTTGGTAACTGAATATTACCATTAATATTAGGAGCACCAGATATGGCATTCCC

ACGACTTAACAACCTAAGATTTTGGTTACTCCCGCCTTCTTTAACTTTATTAGTAGCATC

GGCTGCAGTTGAAAAGGGAGCCGGCACAGGATGAACAGTTTATCCACCTCTATCAAGAAA

TTTAGCTCATGCAGGTCCATCAGTAGACCTAGCAATCTTTTCACTACATCTAGCTGGGGC

ATCCTCCATTCTAGGAGCTATTAACTTTATTACTACGGTAATTAATATACGATGAAATGG

ACTCCGACTTGAACGAGTTCCATTATTTGTATGAGCAGTAACAATTACAGTTGTTCTATT

ACTATTATCATTACCAGTACTAGCTGGGGCAATCACTATACTTCTTACAGATCGAAACTT

AAATACAACATTTTTCGATCCGGCTGGTGGTGGNGAC-----------------------

---------------------------------------------

>684_Lumbriculidae_sp_LL3

---AACCTTATATTTCATCTTAGGAGTTTGAGCTGGAATAGTAGGAGCAGGAATAAGACT

ATTAATTCGAGTTGAATTAACACAACCAGGATCATTTTTAGGAAGTGATCAATTATATAA

TACTATTGTTACGGCTCACGCCTTTATTATAATTTTCTTTATAGTTATACCTATATTTAT

TGGTGGCTTTGGTAACTGAATATTACCATTAATATTAGGAGCACCAGATATGGCATTCCC

ACGACTTAACAACCTAAGATTTTGGTTACTCCCGCCTTCTTTAACTTTATTAGTAGCATC

GGCTGCAGTTGAAAAAGGAGCCGGCACAGGATGAACAGTTTATCCACCTCTATCAAGAAA

CTTAGCTCATGCAGGTCCATCAGTAGACCTAGCAATCTTTTCACTACATCTAGCTGGGGC

ATCCTCCATTCTAGGAGCTATTAACTTTATTACTACGGTAATTAATATACGATGAAATGG

ACTCCGACTTGAACGAGTTCCATTATTTGTATGAGCAGTAACAATTACAGTTGTTCTATT

ACTATTATCATTACCAGTACTAGCTGGAGCAATCACTATACTTCTTACAGATCGAAACTT

AAATACAACATTTTTCGATCCGGCTGGTGGTGGAGACCCAGTATTAT-------------

----------------------------------------------

>685_Lumbriculidae_sp_LL3

---AACCTTATATTTCATCTTAGGAGTTTGAGCTGGAATAGTAGGAGCAGGAATAAGACT

ATTAATTCGAGTTGAATTAACACAACCAGGATCATTTTTAGGAAGTGATCAATTATATAA

TACTATTGTTACGGCCCACGCCTTTATTATAATTTTCTTTATAGTTATACCTATATTTAT

TGGTGGATTTGGTAACTGAATATTACCATTAATATTAGGAGCACCAGATATAGCATTCCC

ACGACTTAACAACCTAAGATTTTGGTTACTCCCGCCTTCTTTAACTTTATTAGTAGCATC

GGCTGCAGTTGAAAAGGGAGCCGGCACAGGATGAACAGTTTATCCACCTCTATCAAGAAA

TTTAGCTCATGCAGGTCCATCAGTAGACCTAGCAATCTTTTCACTACATCTAGCTGGGGC

ATCCTCCATTCTAGGAGCTATTAACTTTATTACTACGGTAATTAATATACGATGAAATGG

ACTCCGACTTGAACGAGTTCCATTATTTGTATGAGCAGTAACAATTACAGTTGTTCTATT

ACTATTATCGTTACCAGTACTAGCTGGGGCAATCACTATACTTCTTACAGATCGAAACTT

AAATACAACATTTTTCGATCCCGCTGGTGGTGGAGACCCAGTATTATATCAACATTTATT

---------------------------------------------

>882_Stylodrilus_heringianus_LL3

---AACCTTATATTTCATCTTAGGAGTTTGAGCTGGAATAGTAGGAGCAGGAATAAGACT

ATTAATTCGAGTTGAATTAACACAACCAGGATCATTTTTAGGAAGTGATCAATTATATAA

TACTATTGTTACGGCCCACGCCTTTATTATAATTTTCTTTATAGTTATACCTATATTTAT

TGGTGGCTTTGGTAACTGAATATTACCATTAATATTAGGAGCACCAGATATAGCATTCCC

ACGACTTAACAACCTAAGATTTTGGTTACTCCCGCCTTCTTTAACTTTATTAGTAGCATC

GGCTGCAGTTGAAAAGGGAGCCGGCACAGGATGAACAGTTTATCCACCTCTATCAAGAAA

TTTAGCTCATGCAGGTCCATCAGTAGACCTAGCAATCTTTTCACTACATCTAGCTGGGGC

ATCCTCCATTCTAGGAGCTATTAACTTTATTACTACGGTAATTAATATACGATGAAATGG

ACTCCGACTTGAACGAGTTCCATTATTTGTATGAGCAGTAACAATTACAGTTGTTCTATT

ACTATTATCGTTACCAGTACTAGCTGGGGCAATCACTATACTTCTTACAGATCGAAACTT

AAATACAACATTTTTCGATCCGGCTGGTGGTGGAGACCCAGTATTATATCAACATTTATT

T--------------------------------------------

>896_Stylodrilus_heringianus_LL3

---AACCTTATATTTCATCTTAGGAGTTTGAGCTGGAATAGTAGGAGCAGGAATAAGACT

ATTAATTCGAGTTGAATTAACACAACCAGGATCATTTTTAGGAAGTGATCAATTATATAA

TACTATTGTTACGGCCCACGCCTTTATTATAATTTTCTTTATAGTTATACCTATATTTAT

TGGTGGCTTTGGTAACTGAATATTACCATTAATATTAGGAGCACCAGATATAGCATTCCC

ACGACTTAACAACCTAAGATTTTGGTTACTCCCGCCTTCTTTAACTTTATTAGTAGCATC

GGCTGCAGTTGAAAAGGGAGCCGGCACAGGATGAACAGTTTATCCACCTCTATCAAGAAA

TTTAGCTCATGCAGGTCCATCAGTAGACCTAGCAATCTTTTCACTACATCTAGCTGGGGC

ATCCTCCATTCTAGGAGCTATTAACTTTATTACTACGGTAATTAATATACGATGAAATGG

ACTCCGACTTGAACGAGTTCCATTATTTGTATGAGCAGTAACAATTACAGTTGTTCTATT

ACTATTATCGTTACCAGTACTAGCTGGGGCAATCACTATACTTCTTACAGATCGAAACTT

AAATACAACATTTTTCGATCCGGCTGGTGGTGGAGACCCAGTATTATATCAACATTTATT

T--------------------------------------------

>897_Stylodrilus_heringianus_LL3

---AACCTTATATTTCATCTTAGGAGTTTGAGCTGGAATAGTAGGAGCAGGAATAAGACT

ATTAATTCGAGTTGAATTAACACAACCAGGATCATTTTTAGGAAGTGATCAATTATATAA

TACTATTGTTACGGCTCACGCCTTTATTATAATTTTCTTTATAGTTATACCTATATTTAT

TGGTGGCTTTGGTAACTGAATATTACCATTAATATTAGGAGCACCAGATATGGCATTCCC

ACGACTTAACAACCTAAGATTTTGGTTACTCCCGCCTTCTTTAACTTTATTAGTAGCATC

GGCTGCAGTTGAAAAAGGAGCCGGCACAGGATGAACAGTTTATCCACCTCTATCAAGAAA

CTTAGCTCATGCAGGTCCATCAGTAGACCTAGCAATCTTTTCACTACATCTAGCTGGGGC

ATCCTCCATTCTAGGAGCTATTAACTTTATTACTACGGTAATTAATATACGATGAAATGG

ACTCCGACTTGAACGAGTTCCATTATTTGTATGAGCAGTAACAATTACAGTTGTTCTATT

ACTATTATCATTACCAGTACTAGCTGGAGCAATCACTATACTTCTTACAGATCGAAACTT

AAATACAACATTTTTCGATCCGGCTGGTGGTGGAGACCCAGTATTATATCAACATTTATT

T--------------------------------------------

>929_Limnodrilus_udekemianus_T23

---CACCCTTTATTTTATCTTCGGCCTATGAGCCGGTATAGTCGGTACCGGAACCAGATT

ATTAATTCGATTTGAACTAGCACAACCCGGATCATTTCTAGGTAGAGACCAACTCTATAA

CACACTAGTAACAGCACATGGATTTCTAATAATTTTCTTCCTTGTAATACCAGTATTTAT

TGGGGGATTCGGCAATTGACTAGTACCATTAATACTTGGGGCTCCCGACATAGCATTCCC

ACGACTAAATAATCTTAGATTTTGACTAATACCTCCATCACTTATTCTACTTGTATCCTC

AGCAGCCGTAGAAAAAGGGGCTGGAACAGGGTGAACTGTATATCCTCCATTAGCAGGGAA

TCTTGCTCATTCAGGCCCTTCTGTAGACCTTGCCATTTTTTCCCTTCATCTGGCCGGAAT

TTCTTCAATTTTAGGGGCTATTAACTTCATCACCACTATAATCAATATACGATGAAAAGG

AATACGACTTGAACGAATTCCTTTATTTGTGTGATCTGTAATTATCACTGTAGTCTTACT

TCTACTAACTCTCCCTGTATTAGCAGGTGCAATTACAATACTTCTAACAGATCGAAATCT

GAATACATCATTCTTTGATCCTGCCGGAGGAGGTGACCCTGTCCTATATCAACATCTATT

C--------------------------------------------

>879_Stylodrilus_heringianus_LL3

---AACCTTATATTTCATCTTAGGAGTTTGAGCTGGAATAGTAGGAGCAGGAATAAGACT

ATTAATTCGAGTTGAATTAACACAACCAGGATCATTTTTAGGAAGTGATCAATTATATAA

TACTATTGTTACGGCCCACGCCTTTATTATAATTTTCTTTATAGTTATACCTATATTTAT

TGGTGGCTTTGGTAACTGAATATTACCATTAATATTAGGAGCACCAGATATAGCATTCCC

ACGACTTAACAACCTAAGATTTTGGTTACTCCCGCCTTCTTTAACTTTATTAGTAGCATC

GGCTGCAGTTGAAAAGGGAGCCGGCACAGGATGAACAGTTTATCCACCTCTATCAAGAAA

TTTAGCTCATGCAGGTCCATCAGTAGACCTAGCAATCTTTTCACTACATCTAGCTGGGGC

ATCCTCCATTCTAGGAGCTATTAACTTTATTACTACGGTAATTAATATACGATGAAATGG

ACTCCGACTTGAACGAGTTCCATTATTTGTATGAGCAGTAACAATTACAGTTGTTCTATT

ACTATTATCGTTACCAGTACTAGCTGGGGCAATCACTATACTTCTTACAGATCGAAACTT

AAATACAACATTTTTCGATCCGGCTGGTGGTGGAGACCCAGTATTATATCAACATTTATT

T--------------------------------------------

>941_Limnodrilus_hoffmeisteri_T18

-------------ATAATCTTCGGCCTATGAGCCGGAATAGTGGGCACAGGAACAAGCCT

GCTAATTCGATTCGAATTAGCACAACCTGGTTCATTCCTCGGTAGAGATCAACTCTATAA

TACCTTAGTGACAGCCCACGGTTTCCTTATAATCTTCTTCATGGTAATACCAATTTTTAT

TGGTGGCTTCGGAAATTGACTAGTCCCCCTAATGCTAGGAGCTCCTGACATGGCCTTTCC

ACGACTAAATAACCTAAGATTTTGACTAATACCTCCATCACTCATTCTATTAGTTTCATC

AGCCGCAGTCGAAAAGGGAGCGGGGACAGGGTGAACTGTATACCCCCCTCTAGCCAGAAA

CCTAGCGCACTCTGGGCCATCTGTGGATCTAGCAATCTTCTCTCTTCACTTAGCCGGGGC

TGCATCAATTCTAGGTGCCATTAACTTCATTACCACAATAATTAATATACGATGAAAAGG

AATGCGCCTAGAGCGCATTCCTCTATTTGTATGATCAGTAATCATTACAGTTATCCTCCT

TCTTCTTACATTACCGGTTTTAGCGGGGGCAATCACCATACTTTTAACAGACCGAAACTT

AAACACATCATTCTTCGATCCTGCGGGAGGGGGGGACCCGGTACTATATCAACACTTATT

---------------------------------------------

>942_Aulodrilus_pluriseta_T4

---AACTCTATACTTCATTTTCGGAATCTGGGCCGGTATAGTTGGTACTGGTACCAGACT

ATTAATTCGCTTAGAACTGGCACAACCTGGATCCTTCTTAGGAAGTGATCAACTATATAA

TACATTAGTAACAGCTCACGCATTTCTAATAATCTTCTTCCTAGTTATACCTGTATTCAT

CGGGGGATTCGGAAATTGATTAATCCCATTAATACTAGGTGCCCCTGATATAGCATTCCC

ACGACTAAATAACCTAAGATTTTGACTAATACCACCCTCACTAATTCTACTAGTTTCATC

CGCAGCTGTAGAAAAAGGTGCGGGGACAGGTTGAACAGTATATCCACCACTTGCTGGCAA

TCTAGCCCATTCAGGACCTTCCGTAGATCTGGCAATCTTCTCTCTACACTTAGCAGGGGT

AGCATCAATTCTAGGTGCTATTAACTTCATTACCACTATAATCAACATACGATGAAAAGG

AATGCGCCTAGAACGAATTCCTCTATTTGTCTGAGCCGTAATTCTTACCGTTGTACTCCT

TCTATTAACCTTACCAGTTCTGGCGGGGGCAATTACTATACTTCTAACTGACCGCAATCT

AAACACCTCCTTCTTCGATCCAGCTGGGGGCGGTGACCCTGTATTATACCAACATCTATT

C--------------------------------------------

>943_Nais_elinguis_N4

---TACATTATACTTAATTCTAGGAGTATGAGCGGGAATAGTTGGAACTGGAACAAGAAT

ACTAATTCGAATTGAACTTGCTCAACCAGGAGCCTTCCTCGGAAGAGACCAACTCTATAA

CACCCTAGTTACAGCTCATGCATTTTTAATAATTTTCTTTCTTGTAATGCCAGTATTTAT

TGGTGGATTCGGAAATTGACTTCTACCACTAATACTTGGAGCCCCAGATATAGCATTTCC

ACGATTAAATAATCTAAGATTTTGATTACTACCACCATCATTAATTCTACTAATTTCATC

AGCCGCAGTTGAAAAAGGTGCAGGAACAGGATGAACTGTTTACCCTCCATTATCAAGAAA

TTTAGCTCATGCTGGACCATCAGTTGACATGGCTATTTTTTCTCTTCATTTAGCAGGTGC

ATCATCTATTTTAGGTGCAGTAAATTTTATTACTACAGTAATAAATATACGATGAAACGG

TATACGATTAGAACGTTTACCTTTATTTGTATGAGCTGTATTCCTTACTGTAATTCTCCT

TCTACTATCGCTACCAGTATTAGCTGGCGCTATTACAATACTTCTTACAGACCGTAATTT

AAATACCTCATTCTTTGATCCAGCAGGAGGAGGAGATCCGATTCTTTATCAACACTTATT

T--------------------------------------------

>954_Henlea_perpusilla_E16

---AACNCTATATTTCATTCTAGGCGTATGAGCCAGAATGATAGGAGCAGCCATAAGCCT

TCTAATTCGAATTGAACTAAGTCAACCAGGTTCATTCCTCGGAAGAGACCAACTCTACAA

CACTATTGTTACTGCACATGCATTTCTTATAATTTTTTTCCTAGTTATACCTGTATTTAT

TGGGGGGTTCGGAAACTGACTTCTCCCATTAATACTAGGGGCCCCAGATATAGCTTTTCC

CCGACTAAATAACATAAGTTTTTGACTTCTTCCCCCATCACTTCTTCTTCTACTTTCCTC

TGCAGCTGTGGAAAAAGGAGCCGGAACCGGCTGAACTGTATATCCCCCCCTATCAAGAAA

CATCGCTCATGCAGGCCCATCTGTAGACCTAGCCATTTTCTCCCTCCACTTAGCCGGAGC

ATCCTCCATCTTAGGTGCAGTAAACTTTATCACTACTGTTATTAATATACGTTGACAAGG

CCTTCACCTAGAACGAATTCCCCTATTTGTATGAGCTGTAACAATTACAGTAGTCCTTCT

CCTCTTATCTTTACCAGTATTAGCCGGAGCAATTACTATACTACTAACTGACCGAAACCT

AAACACATCCTTTTTCGACCCAGCTGGAGGAGGTGACCCAATTCTATATCAACATCTATT

T--------------------------------------------

>955_Tubifex_tubifex_T11

---AACTCTATATATCATTTTCGGGATTTGAGCTGGGATGGTCGGGACAGGAACTAGACT

CTTAATTCGTCTAGAACTGGCTCAACCTGGCTCATTCCTTGGAAGGGATCAGCTATACAA

CACCCTAGTTACAGCACATGCATTTCTGATAATTTTCTTCATGGTAATACCTATCTATAT

TGGGGGTTTCGGAAATTGGTTAGTACCCTTAATGCTGGGAGCTCCTGACATAGCATTCCC

CCGATTAAATAATTTAAGATTTTGGTTATTACCCCCTTCCTTAATCCTCCTTGTATCCTC

CGCGGCCGTAGAAAAGGGGGCTGGAACAGGGTGAACAGTATATCCGCCCCTAGCTAGAAA

TCTGGCTCATTCTGGGCCTTCTGTAGACTTAGCTATTTTCTCTCTACATTTAGCTGGGGT

GGCATCGATTCTAGGAGCTATTAACTTTATTACTACAATAATTAACATGCGATGAAAAGG

GATACGCCTTGAACGAATTCCCCTATTTGTATGAGCTGTAATTCTAACCGTAATCTTACT

TTTACTAACATTACCAGTTTTAGCTGGTGCCATTACCATGCTACTAACAGATCGAAATCT

AAACACATCCTTCTTTGACCCTGCTGGGGGTGGAGATCCTGTGCTCTATCAACACTTATT

C--------------------------------------------

>956_Tubifex_tubifex_T11

---AACTCTATATATTATTTTCGGGATTTGAGCTGGAATGGTCGGGACAGGAACTAGCCT

CTTAATTCGTCTAGAACTGGCTCAACCTGGCTCATTCCTTGGAAGGGATCAACTATACAA

CACCCTAGTTACAGCACACGCATTTCTGATAATTTTCTTCATGGTAATACCTATCTATAT

CGGGGGCTTCGGCAATTGACTAGTGCCTTTAATACTGGGGGCCCCTGACATAGCATTCCC

ACGATTAAATAATTTAAGATTTTGACTATTACCTCCTTCCTTAATCCTCCTTGTATCCTC

CGCAGCCGTAGAAAAGGGGGCTGGAACAGGGTGAACAGTATATCCACCCCTAGCTAGAAA

TCTGGCTCATTCTGGNCCTTCTGTAGACTTAGCTATTTTCTCCCTACATTTAGCGGGAGT

AGCATCGATTCTAGGGGCTATTAACTTTATTACTACAATAATTAACATGCGGTGAAAAGG

GATACGTCTTGAACGAATTCCTCTATTTGTATGAGCCGTAATTCTAACTGTAATCTTACT

TCTACTAACATTACCAGTTTTAGCGGGTGCTATTACCATGCTACTGACAGATCGAAACCT

AAACACATCCTTCTTTGACCCTGCTGGGGGTGGTGATCCTGTACTCTATCAACACTTATT

C--------------------------------------------

>957_Stylodrilus_heringianus_LL3

---AACCTTATATTTCATCTTAGGAGTTTGAGCTGGGATAGTAGGAGCAGGGATGAGACT

ATTAATTCGAGTTGAATTAACACAACCCGGGTCATTTTTAGGAAGTGACCAACTATATAA

TACTATTGTTACGGCTCACGCATTTATTATAATTTTCTTTATAGTTATACCTATATTTAT

TGGTGGTTTTGGTAACTGAATACTACCGTTAATATTAGGAGCACCAGACATAGCATTTCC

ACGACTTAACAACCTAAGATTTTGATTACTACCACCTTCTTTAACTTTATTAGTAGCATC

GGCTGCAGTAGAAAAAGGAGCCGGCACAGGATGAACAGTTTATCCACCTCTATCAAGAAA

TTTAGCTCATGCTGGACCCTCAGTAGACCTGGCAATCTTCTCACTCCATCTAGCAGGAGC

CTCCTCCATTTTAGGAGCCATTAACTTTATTACGACTGTAATTAATATACGATGAAATGG

ACTACGACTTGAACGAGTTCCATTATTTGTATGAGCAGTAACAATTACAGTAGTTCTATT

ACTATTATCATTACCAGTACTAGCTGGAGCAATCACTATACTTCTTACAGATCGAAACTT

AAATACAACATTTTTTGATCCAGCTGGAGGTGGAGACCCCGTACTATATCAACATTTATT

T--------------------------------------------

>958_Tubifex_tubifex_T11

---AACTCTATATATCATTTTCGGGATTTGAGCTGGGATGGTCGGGACAGGAACTAGACT

CTTAATTCGTCTAGAACTGGCTCAACCTGGCTCATTCCTTGGAAGGGATCAGCTATACAA

CACCCTAGTTACAGCACATGCATTTCTGATAATTTTCTTCATGGTAATACCTATCTATAT

TGGGGGTTTCGGAAATTGGTTAGTACCCTTAATGCTGGGAGCTCCTGACATAGCATTCCC

CCGATTAAATAATTTAAGATTTTGGTTATTACCCCCTTCCTTAATCCTCCTTGTATCCTC

CGCGGCCGTAGAAAAGGGGGCTGGAACAGGGTGAACAGTATATCCGCCCCTAGCTAGAAA

TCTGGCTCATTCTGGGCCTTCTGTAGACTTAGCTATTTTCTCTCTACATTTAGCTGGGGT

GGCATCGATTCTAGGAGCTATTAACTTTATTACTACAATAATTAACATGCGATGAAAAGG

GATACGCCTTGAACGAATTCCCCTATTTGTATGAGCTGTAATTCTAACCGTAATCTTACT

TTTACTAACATTACCAGTTTTAGCTGGTGCCATTACCATGCTACTAACAGATCGAAATCT

AAACACATCCTTCTTTGACCCTGCTGGGGGTGGAGATCCTGTGCTCTATCAACACTTATT

C--------------------------------------------

>959_Tubificinae_sp_T32

---ATTTTTATATATATTATTTGGGCTATGAGCTGGAATAGTAGGAACTGGTACCAGTCT

TCTAATTCGGTTAGAACTTGCTCAACCAGGCTCATTCCTGGGAAGAGATCAACTTTATAA

TACTATTGTAACAGCACATGCATTCCTGATAATCTTCTACATAGTAATACCAATTTATGT

AGGAGGATTTGGAAACTATTTAATACCTTTAATACTGGGGGCACCAGATATAGCATTCCC

ACGACTAAATAACCTGAGATTCTGATTAATACCGCCATCTTTAATTCTTCTAGTAGCATC

AGCTGCTGTTGAAAAAGGGGCCGGAACTGGATGAACTGTTTACCCTCCTCTAGCTAGAAA

TCTGGCACATTCTGGTCCATCAGTAGATCTAGCTATTTTTGCCTTACATCTTGCTGGTGC

AGCCTCTATTATCGGAGCCATTAATTTCATTACAACAATAATTAATATACGCTGAAAAGG

GATGCGACTAGAGCGTATTCCCCTGTTCGTGTGATCTGTAATTATTACTGTAGTTCTATT

ATTACTTACACTTCCTGTTCTTGCAGGTGCCATTACTATACTCCTAACAGATCGAAATCT

AAATACATCATTCTTCGATCCTGCTGGTGGCGGTGACCCTGTACTATACCAACATCTATT

C--------------------------------------------

>960_Nais_alpina_N8

---TACACTATATTTAATTTTAGGAGTATGAGCAGGAATAGTGGGAACTGGAACAAGATT

ACTTATTCGAATTGAACTATCACAACCAGGATCATTTCTTGGAAGAGATCAACTATACAA

CACTCTTGTAACAGCACATGCATTCTTAATAATTTTCTTCTTGGTAATACCAGTATTCAT

TGGGGGGTTTGGAAACTGACTTCTTCCATTAATACTAGGTGCTGCCGATATAGCATTTCC

ACGACTTAATAATCTTAGATTTTGATTGCTACCACCATCATTAATTTTATTAATTTCTTC

TGCAGCTGTAGAAAAAGGTGCTGGAACAGGATGAACTGTTTATCCGCCTCTATCAAGAAA

TCTAGCACATGCCGGACCTTCTGTAGATATGGCTATTTTTTCACTTCATTTAGCAGGTGC

TTCATCTATTTTAGGAGCAGTAAATTTTATTACTACAGTAATAAATATACGATGAAATGG

AATACGACTAGAACGGCTACCATTATTTGTTTGAGCAGTATTTCTTACAGTAATTCTTCT

TTTATTATCTCTTCCAGTACTTGCTGGGGCAATTACAATACTATTAACAGATCGAAATCT

TAATACTTCATTTTTTGATCCTGCTGGAGGTGGGGATCCAATCTTATATCAACATCTATT

T--------------------------------------------

>961_Eiseniella_tetraedra_LC3

---AACCCTTTACTTCATTTTAGGTATTTGAGCTGGGATGGTAGGAGCTGGAATAAGCTT

ACTAATTCGAATCGAATTAAGCCAACCTGGAGCCTTTTTAGGTAGTGACCAACTATACAA

CACTATTGTCACAGCACACGCATTCGTAATAATCTTTTTCCTAGTAATACCAGTATTCAT

TGGTGGATTCGGAAACTGGTTATTACCTTTAATACTTGGTGCGCCAGATATAGCATTTCC

ACGTTTAAATAACATAAGATTTTGACTTTTACCTCCTTCTCTAATTCTATTAGTATCTTC

CGCCGCCGTAGAAAAGGGTGCCGGGACAGGTTGAACTGTTTACCCACCCTTAGCAAGAAA

CTTAGCTCATGCAGGACCATCCGTAGATCTAGCTATTTTCTCCTTACATTTAGCGGGAGC

TTCATCTATTTTAGGGGCCATCAACTTTATTACCACAGTCATTAATATACGATGAAGGGG

CTTACGATTAGAACGAATCCCCCTTTTCGTCTGAGCTGTGCTAATTACAGTGATTCTTCT

ACTACTATCATTGCCCGTGCTTGCAGGAGCAATTACCATGTTATTAACTGACCGAAATCT

CAACACATCTTTTTTTGATCCGGCTGGTGGTGGTGACCCAATTCTATATCAACACCTCTT

C--------------------------------------------

>962_Tubifex_tubifex_T11

---AACTCTATATATTATTTTCGGGATTTGAGCTGGAATGGTCGGGACAGGAACTAGCCT

CTTAATTCGTCTAGAACTGGCTCAACCTGGCTCATTCCTTGGAAGGGATCAACTATACAA

CACCCTAGTTACAGCACACGCATTTCTGATAATTTTCTTCATGGTAATACCTATCTATAT

CGGGGGCTTCGGCAATTGACTAGTGCCTTTAATACTGGGGGCCCCTGACATAGCATTCCC

ACGATTAAATAATTTAAGATTTTGACTATTACCTCCTTCCTTAATCCTCCTTGTATCCTC

CGCAGCCGTAGAAAAGGGGGCTGGAACAGGGTGAACAGTATATCCACCCCTAGCTAGAAA

TCTGGCTCATTCTGGACCTTCTGTAGACTTAGCTATTTTCTCCCTACATTTAGCGGGAGT

AGCATCGATTCTAGGGGCTATTAACTTTATTACTACAATAATTAACATGCGGTGAAAAGG

GATACGTCTTGAACGAATTCCTCTATTTGTATGAGCCGTAATTCTAACTGTAATCTTACT

TCTACTAACATTACCAGTTTTAGCGGGTGCTATTACCATGCTACTGACAGATCGAAACCT

AAACACATCCTTCTTTGACCCTGCTGGGGGTGGTGATCCTGTACTCTATCAACACTTATT

C--------------------------------------------

>963_Lumbriculidae_sp_LL3

---AACCTTATATTTCATCTTAGGAGTTTGAGCTGGAATAGTAGGAGCAGGAATAAGACT

ATTAATTCGAGTTGAATTAACACAACCAGGATCATTTTTAGGAAGTGATCAATTATATAA

TACTATTGTTACGGCCCACGCCTTTATTATAATTTTCTTTATAGTTATACCTATATTTAT

TGGTGGCTTTGGTAACTGAATATTACCATTAATATTAGGAGCACCAGATATAGCATTCCC

ACGACTTAACAACCTAAGATTTTGGTTACTCCCGCCTTCTTTAACTTTATTAGTAGCATC

GGCTGCAGTTGAAAAGGGAGCCGGCACAGGATGAACAGTTTATCCACCTCTATCAAGAAA

TTTAGCTCATGCAGGTCCATCAGTAGACCTAGCAATCTTTTCACTACATCTAGCTGGGGC

ATCCTCCATTCTAGGAGCTATTAACTTTATTACTACGGTAATTAATATACGATGAAATGG

ACTCCGACTTGAACGAGTTCCATTATTTGTATGAGCAGTAACAATTACAGTTGTTCTATT

ACTATTATCGTTACCAGTACTAGCTGGGGCAATCACTATACTTCTTACAGATCGAAACTT

AAATACAACATTTTTCGATCCGGCTGGTGGTGGAGACCCAGTATTATATCAACATTTATT

T--------------------------------------------

>964_Tubifex_tubifex_T11

---AACTCTATATATCATTTTCGGGATTTGAGCTGGGATGGTCGGGACAGGAACTAGACT

CTTAATTCGTCTAGAACTGGCTCAACCTGGCTCATTCCTTGGAAGGGATCAGCTATACAA

CACCCTAGTTACAGCACATGCATTTCTGATAATTTTCTTCATGGTAATACCTATCTATAT

TGGGGGTTTCGGAAATTGGTTAGTACCCTTAATGCTGGGAGCTCCTGACATAGCATTCCC

CCGATTAAATAATTTAAGATTTTGGTTATTACCCCCTTCCTTAATCCTCCTTGTATCCTC

CGCGGCCGTAGAAAAGGGGGCTGGAACAGGGTGAACAGTATATCCGCCCCTAGCTAGAAA

TCTGGCTCATTCTGGGCCTTCTGTAGACTTAGCTATTTTCTCTCTACATTTAGCTGGGGT

GGCATCGATTCTAGGAGCTATTAACTTTATTACTACAATAATTAACATGCGATGAAAAGG

GATACGCCTTGAACGAATTCCCCTATTTGTATGAGCTGTAATTCTAACCGTAATCTTACT

TTTACTAACATTACCAGTTTTAGCTGGTGCCATTACCATGCTACTAACAGATCGAAATCT

AAACACATCCTTCTTTGACCCTGCTGGGGGTGGAGATCCTGTGCTCTATCAACACTTATT

C--------------------------------------------

>1000_Tubifex_tubifex_T11

---AACTCTATATATCATTTTCGGGATTTGAGCTGGGATGGTCGGGACAGGAACTAGACT

CTTAATTCGTCTAGAACTGGCTCAACCTGGCTCATTCCTTGGAAGGGATCAGCTATACAA

CACCCTAGTTACAGCACATGCATTTCTGATAATTTTCTTCATGGTAATACCTATCTATAT

TGGGGGTTTCGGAAATTGGTTAGTACCCTTAATGCTGGGAGCTCCTGACATAGCATTCCC

CCGATTAAATAATTTAAGATTTTGGTTATTACCCCCTTCCTTAATCCTCCTTGTATCCTC

CGCGGCCGTAGAAAAGGGGGCTGGAACAGGGTGAACAGTATATCCGCCCCTAGCTAGAAA

TCTGGCTCATTCTGGGCCTTCTGTAGACTTAGCTATTTTCTCTCTACATTTAGCTGGGGT

GGCATCGATTCTAGGAGCTATTAACTTTATTACTACAATAATTAACATGCGATGAAAAGG

GATACGCCTTGAACGAATTCCCCTATTTGTATGAGCTGTAATTCTAACCGTAATCTTACT

TTTACTAACATTACCAGTTTTAGCTGGTGCCATTACCATGCTACTAACAGATCGAAATCT

AAACACATCCTTCTTTGACCCTGCTGGGGGTGGAGATCCTGTGCTCTATCAACACTTATT

C--------------------------------------------

>1001_Limnodrilus_hoffmeisteri_T17

---CACCCTATACATAATCTTTGGCCTTTGAGCAGGAATAGTAGGCACAGGAACTAGACT

TTTAATTCGATTTGAGCTAGCACAACCCGGCTCATTTCTCGGTAGAGACCAATTATATAA

CACTCTAGTCACGGCTCACGGATTTTTAATAATTTTCTTTATAGTAATACCTATCTTTAT

TGGGGGATTTGGAAATTGATTAGTGCCTTTAATACTTGGAGCACCTGATATGGCATTCCC

ACGGCTTAATAACCTAAGATTCTGACTAATGCCCCCATCACTAATTCTACTAGTCTCATC

AGCTGCAGTTGAAAAAGGCGCAGGGACAGGGTGAACTGTATACCCCCCCTTAGCAAGAAA

TCTAGCTCATTCCGGGCCTTCTGTAGATCTGGCAATTTTTTCACTACACTTAGCAGGAGC

CGCATCAATCCTGGGAGCAATTAACTTCATCACAACAATAATCAATATACGATGAAAGGG

AATACGCTTAGAGCGTATTCCTTTATTTGTGTGATCCGTAATCATCACAGTTATTTTACT

TCTTCTTACCCTTCCAGTTCTTGCCGGAGCTATCACCATACTTTTAACAGATCGAAACCT

AAACACCTCATTCTTTGATCCGGCAGGTGGAGGCGATCCAGTTTTATACCAACATCTATT

T--------------------------------------------

>1002_Tubifex_tubifex_T12

---AACTCTATACATAATTTTCGGAATCTGAGCTGGTATGGTTGGAACTGGAACTAGGCT

ATTAATTCGCCTTGAACTTGCCCAACCCGGGTCCTTTTTAGGAAGAGATCAATTATATAA

TACTCTGGTAACGGCTCATGCATTTTTAATAATCTTCTTCATGGTGATACCAATCTATAT

TGGGGGCTTCGGCAACTGACTAGTACCATTAATACTGGGGGCTCCAGACATGGCCTTCCC

ACGATTAAACAACTTAAGATTCTGACTCCTACCCCCATCATTAATCCTTTTAGTATCCTC

TGCCGCCGTTGAAAAAGGCGCCGGAACTGGCTGAACTGTTTACCCCCCTCTAGCTAGAAA

TCTAGCTCACTCTGGGCCCTCAGTAGATTTAGCAATCTTTTCTCTACACTTAGCTGGTGT

TGCATCAATCCTGGGTGCAATCAACTTCATTACCACAATAATCAACATACGATGAAAAGG

GATGCGACTAGAACGTATTCCTTTATTTGTATGATCAGTAATTATCACTGTAATTCTACT

CCTACTCACACTTCCAGTTTTAGCTGGGGCTATCACAATACTTCTAACAGACCGGAATCT

AAATACATCATTTTTCGATCCTGCTGGAGGGGGTGACCCTGTACTATATCAACACCTATT

C--------------------------------------------

>1003_Lophochaeta_ignota_T6

---AACTCTCTATATAATCTTCGGCCTATGAGCGGGAATAGTCGGCACCGGTACTAGACT

ACTAATTCGACTAGAATTAGCTCAACCCGGATCATTCCTTGGTAGTGATCAACTATATAA

TACACTTGTTACTGCCCATGGATTCCTTATAATTTTCTTCATGGTAATACCAGTATTTAT

TGGGGGATTTGGTAACTGACTAGTCCCATTAATACTTGGTGCCCCAGATATAGCCTTCCC

ACGAATAAATAATTTAAGATTTTGATTAATACCCCCCTCTTTAATCCTTCTCGTATCTTC

AGCAGCCGTTGAAAAAGGTGCTGGAACAGGCTGAACAGTATATCCTCCATTAGCCGGGAA

TTTAGCTCACTCAGGACCATCCGTAGACCTAGCTATTTTCTCACTGCATTTAGCCGGAGC

TGCCTCAATTTTAGGTGCAATTAACTTTATTACGACTATAATTAACATGCGATGAAAAGG

AATACGCCTAGAACGCATTCCTCTATTTGTTTGATCCGTAATTATCACAGTAGTCCTACT

TTTATTAACACTACCAGTTCTAGCAGGGGCCATTACAATATTACTTACAGACCGAAACTT

AAATACCTCATTCTTTGACCCTGCTGGAGGTGGAGACCCTGTTTTATATCAACATCTATT

C--------------------------------------------

>1004_Aulodrilus_pluriseta_T4

---AACTCTATACTTCATTTTTGGAATCTGAGCCGGTATAGTTGGTACCGGTACCAGACT

ATTAATCCGCTTAGAACTGGCACAACCTGGATCCTTCTTAGGAAGTGATCAACTATACAA

TACATTAGTAACAGCTCACGCATTTCTAATAATCTTCTTCCTAGTTATACCTGTATTCAT

CGGGGGATTCGGAAATTGATTAATCCCATTAATACTAGGTGCTCCTGATATAGCATTTCC

ACGACTAAATAACCTAAGATTTTGACTAATACCCCCCTCACTAATTCTACTAGTTTCATC

AGCAGCTGTAGAAAAAGGTGCAGGGACAGGTTGAACAGTATATCCACCACTTGCTGGCAA

TCTAGCCCATTCAGGACCTTCTGTAGATCTAGCAATCTTCTCTCTACACTTAGCGGGAGT

AGCATCAATTCTAGGTGCTATTAACTTCATTACCACTATAATCAACATACGATGAAAAGG

AATGCGCCTAGAACGAATTCCTCTATTTGTCTGAGCCGTAATTCTTACCGTTGTACTCCT

TCTATTAACCTTACCAGTTCTGGCGGGGGCAATTACTATACTTCTAACTGACCGCAATCT

AAACACCTCCTTCTTCGATCCAGCTGGGGGCGGTGACCCTGTATTATACCAACATCTATT

C--------------------------------------------

>1005_Lophochaeta_ignota_T6

---AACTCTCTATATAATCTTCGGCCTATGAGCGGGAATAGTCGGCACCGGTACTAGACT

ACTAATTCGACTAGAATTAGCTCAACCCGGATCATTCCTTGGTAGTGATCAACTATATAA

TACACTTGTTACTGCCCATGGATTCCTTATAATTTTCTTCATGGTAATACCAGTATTTAT

TGGGGGATTTGGTAACTGACTAGTCCCATTAATACTTGGTGCCCCAGATATAGCCTTCCC

ACGAATAAATAATTTAAGATTTTGATTAATACCCCCCTCTTTAATCCTTCTCGTATCTTC

AGCAGCCGTTGAAAAAGGTGCTGGAACAGGCTGAACAGTATATCCTCCATTAGCCGGGAA

TTTAGCTCACTCAGGACCATCCGTAGACCTAGCTATTTTCTCACTGCATTTAGCCGGAGC

TGCCTCAATTTTAGGTGCAATTAACTTTATTACGACTATAATTAACATGCGATGAAAAGG

AATACGCCTAGAACGCATTCCTCTATTTGTTTGATCCGTAATTATCACAGTAGTCCTACT

TTTATTAACACTACCAGTTCTAGCAGGGGCCATTACAATATTACTTACAGACCGAAACTT

AAATACCTCATTCTTTGACCCTGCTGGAGGTGGAGACCCTGTTTTATATCAACATCTATT

C--------------------------------------------

>1006_Aulodrilus_pluriseta_T4

---AACTCTATACTTCATTTTTGGAATCTGAGCCGGTATAGTTGGTACCGGTACCAGACT

ATTAATCCGCTTAGAACTGGCACAACCTGGATCCTTCTTAGGAAGTGATCAACTATACAA

TACATTAGTAACAGCTCACGCATTTCTAATAATCTTCTTCCTAGTTATACCTGTATTCAT

CGGGGGATTCGGAAATTGATTAATCCCATTAATACTAGGTGCTCCTGATATAGCATTTCC

ACGACTAAATAACCTAAGATTTTGACTAATACCCCCCTCACTAATTCTACTAGTTTCATC

AGCAGCTGTAGAAAAAGGTGCAGGGACAGGTTGAACAGTATATCCACCACTTGCTGGCAA

TCTAGCCCATTCAGGACCTTCTGTAGATCTAGCAATCTTCTCTCTACACTTAGCGGGAGT

AGCATCAATTCTAGGTGCTATTAACTTCATTACCACTATAATCAACATACGATGAAAAGG

AATGCGCCTAGAACGAATTCCTCTATTTGTCTGAGCCGTAATTCTTACCGTTGTACTCCT

TCTATTAACCTTACCAGTTCTGGCGGGGGCAATTACTATACTTCTAACTGACCGCAATCT

AAACACCTCCTTCTTCGATCCAGCTGGGGGCGGTGACCCTGTATTATACCAACATCTATT

C--------------------------------------------

>1007_Lumbriculidae_sp_LL3

---AACCTTATATTTCATCTTAGGAGTTTGAGCTGGAATAGTAGGAGCAGGAATAAGACT

ATTAATTCGAGTTGAATTAACACAACCAGGATCATTTTTAGGAAGTGATCAATTATATAA

TACTATTGTTACGGCCCACGCCTTTATTATAATTTTCTTTATAGTTATACCTATATTTAT

TGGTGGCTTTGGTAACTGAATATTACCATTAATATTAGGAGCACCAGATATAGCATTCCC

ACGACTTAACAACCTAAGATTTTGGTTACTCCCGCCTTCTTTAACTTTATTAGTAGCATC

GGCTGCAGTTGAAAAGGGAGCCGGCACAGGATGAACAGTTTATCCACCTCTATCAAGAAA

TTTAGCTCATGCAGGTCCATCAGTAGACCTAGCAATCTTTTCACTACATCTAGCTGGGGC

ATCCTCCATTCTAGGAGCTATTAACTTTATTACTACGGTAATTAATATACGATGAAATGG

ACTCCGACTTGAACGAGTTCCATTATTTGTATGAGCAGTAACAATTACAGTTGTTCTATT

ACTATTATCGTTACCAGTACTAGCTGGGGCAATCACTATACTTCTTACAGATCGAAACTT

AAATACAACATTTTTCGATCCGGCTGGTGGTGGAGACCCAGTATTATATCAACATTTATT

T--------------------------------------------

>1008_Stylodrilus_heringianus_LL3

---AACCTTATATTTCATCTTAGGAGTTTGAGCTGGAATAGTAGGAGCAGGAATAAGACT

ATTAATTCGAGTTGAATTAACACAACCAGGATCATTTTTAGGAAGTGATCAATTATATAA

TACTATTGTTACGGCTCACGCCTTTATTATAATTTTCTTTATAGTTATACCTATATTTAT

TGGTGGCTTTGGTAACTGAATATTACCATTAATATTAGGAGCACCAGATATGGCATTCCC

ACGACTTAACAACCTAAGATTTTGGTTACTCCCGCCTTCTTTAACTTTATTAGTAGCATC

GGCTGCAGTTGAAAAAGGAGCCGGCACAGGATGAACAGTTTATCCACCTCTATCAAGAAA

CTTAGCTCATGCAGGTCCATCAGTAGACCTAGCAATCTTTTCACTACATCTAGCTGGGGC

ATCCTCCATTCTAGGAGCTATTAACTTTATTACTACGGTAATTAATATACGATGAAATGG

ACTCCGACTTGAACGAGTTCCATTATTTGTATGAGCAGTAACAATTACAGTTGTTCTATT

ACTATTATCATTACCAGTACTAGCTGGAGCAATCACTATACTTCTTACAGATCGAAACTT

AAATACAACATTTTTCGATCCGGCTGGTGGTGGAGACCCAGTATTATATCAACATTTATT

T--------------------------------------------

>1009_Lumbriculidae_sp_LL3

---AACCTTATATTTCATCTTAGGAGTTTGAGCTGGAATAGTAGGAGCAGGAATAAGACT

ATTAATTCGAGTTGAATTAACACAACCAGGATCATTTTTAGGAAGTGATCAATTATATAA

TACTATTGTTACGGCCCACGCCTTTATTATAATTTTCTTTATAGTTATACCTATATTTAT

TGGTGGCTTTGGTAACTGAATATTACCATTAATATTAGGAGCACCAGATATAGCATTCCC

ACGACTTAACAACCTAAGATTTTGGTTACTCCCGCCTTCTTTAACTTTATTAGTAGCATC

GGCTGCAGTTGAAAAGGGAGCCGGCACAGGATGAACAGTTTATCCACCTCTATCAAGAAA

TTTAGCTCATGCAGGTCCATCAGTAGACCTAGCAATCTTTTCACTACATCTAGCTGGGGC

ATCCTCCATTCTAGGAGCTATTAACTTTATTACTACGGTAATTAATATACGATGAAATGG

ACTCCGACTTGAACGAGTTCCATTATTTGTATGAGCAGTAACAATTACAGTTGTTCTATT

ACTATTATCGTTACCAGTACTAGCTGGGGCAATCACTATACTTCTTACAGATCGAAACTT

AAATACAACATTTTTCGATCCGGCTGGTGGTGGAGACCCAGTATTATATCAACATTTATT

T--------------------------------------------

>965_Nais_communis_N10

------------------TTTAGGAGTATGAGCAGGAATAGTAGGAACTGGAACTAGATT

ACTTATTCGAATTGAATTATCACAACCAGGATCATTTCTTGGAAGAGATCAATTATATAA

TACTCTTGTAACAGCACACGCGTTCTTAATAATTTTCTTCTTAGTAATACCAGTATTTAT

TGGGGGGTTCGGAAACTGACTTCTCCCACTAATACTAGGTGCTGCTGATATAGCATTCCC

ACGACTAAACAATCTTAGATTTTGACTACTACCACCATCATTAATTCTATTAATTTCTTC

TGCTGCTGTAGAAAAAGGTGCAGGAACAGGATGAACTGTTTATCCGCCTCTATCAAGAAA

TCTAGCACACGCTGGACCTTCAGTAGACATGGCCATTTTCTCACTTCACTTAGCAGGTGC

TTCTTCTATTTTAGGGGCAGTAAATTTCATTACAACAGTAATAAATATACGATGAAACGG

AATACGATTAGAACGACTTCCACTATTCGTATGAGCAGTATTTCTTACAGTAATTCTCCT

TCTTCTATCACTTCCCGTTCTTGCTGGTGCAATTACAATACTATTAACAGATCGAAATCT

TAATACCTCATTCTTCGATCCTGCTGGTGGTGGAGATCCGATCTTATATCAACACTTATT

C--------------------------------------------

>966_Ophidonais_serpentina_N5

---------------------AGGAGTATGAGCAGGAATAGTTGGTACAGGAACAAGAAT

ACTGATTCGAATTGAACTAGCTCAACCAGGAGCTTTTCTAGGAAGAGATCAATTATATAA

CACTCTAGTAACAGCACATGCGTTTTTAATAATTTTCTTTTTAGTTATACCTGTATTTAT

TGGCGGATTCGGAAACTGACTTCTTCCATTAATATTAGGTGCTCCAGATATGGCATTCCC

ACGACTAAATAATCTTAGATTCTGACTTCTACCACCATCATTAATTCTATTAATTTCATC

TGCAGCCGTTGAAAAAGGTGCTGGAACAGGATGAACTGTATATCCTCCATTATCAAGAAA

TCTAGCTCACGCTGGACCTTCAGTTGACATGGCTATTTTTTCACTACATCTAGCAGGTGC

ATCTTCTATTTTAGGTGCAGTTAACTTCATTACTACAGTAATAAACATACGATGAAATGG

AATACGACTTGAACGAGTACCATTATTTGTATGAGCTGTAACACTTACTGTAATTCTTCT

TCTTTTATCATTACCTGTATTAGCTGGTGCAATTACCATACTATTAACAGATCGAAATCT

AAATACCTCATTCTTCGATCCTGCAGGAGGGGGAGACCCAATTTTATACCAACATTTATT

C--------------------------------------------

>967_Nais_elinguis_N4

---TACATTATACTTATTTCTAGGAGTATGAGCGGGAATAGTTGGAACTGGAACAAGAAT

ACTAATTCGAATTGAACTTGCTCAACCAGGAGCCTTCCTCGGAAGAGACCAACTCTATAA

CACCCTAGTTACAGCTCATGCATTTTTAATAATTTTCTTTCTTGTAATGCCAGTATTTAT

TGGTGGATTCGGAAATTGACTTCTACCACTAATACTTGGAGCCCCAGATATAGCATTTCC

ACGATTAAATAATCTAAGATTTTGATTACTACCACCATCATTAATTCTACTAATTTCATC

AGCCGCAGTTGAAAAAGGTGCAGGAACAGGATGAACTGTTTACCCTCCATTATCAAGAAA

TTTAGCTCATGCTGGACCATCAGTTGACATGGCTATTTTTTCTCTTCATTTAGCAGGTGC

ATCATCTATTTTAGGTGCAGTAAATTTTATTACTACAGTAATAAATATACGATGAAACGG

TATACGATTAGAACGTTTACCTTTATTTGTATGAGCTGTATTCCTTACTGTAATTCTCCT

TCTACTATCGCTACCAGTATTAGCTGGCGCTATTACAATACTTCTTACAGACCGTAATTT

AAATACCTCATTCTTTGATCCAGCAGGAGGAGGAGATCCGATTCTTTATCAACACTTATT

T--------------------------------------------

>968_Tubifex_tubifex_T12

---AACTCTATACATAATTTTCGGAATCTGAGCTGGTATGGTTGGAACTGGAACTAGGCT

ATTAATTCGCCTTGAACTTGCCCAACCTGGGTCCTTTTTAGGAAGAGATCAATTATATAA

TACTCTGGTAACGGCTCATGCATTTTTAATAATCTTCTTCATGGTGATACCAATCTATAT

TGGGGGCTTCGGCAACTGACTAGTACCATTAATACTGGGGGCTCCAGACATGGCCTTCCC

ACGATTAAACAACTTAAGATTCTGACTCCTACCCCCATCATTAATCCTTTTAGTATCCTC

TGCCGCCGTTGAAAAAGGCGCCGGAACTGGCTGAACTGTTTACCCCCCTCTAGCTAGAAA

TCTAGCTCACTCTGGGCCCTCAGTAGATTTAGCAATCTTTTCTCTACACTTAGCTGGTGT

TGCATCAATCCTGGGTGCAATCAACTTCATTACCACAATAATCAACATACGATGAAAAGG

GATGCGACTAGAACGTATTCCTTTATTTGTATGATCGGTAATTATCACTGTAATTCTACT

CCTACTCACACTTCCAGTTTTAGCTGGGGCTATCACAATACTTCTAACAGACCGGAATCT

AAATACATCATTTTTCGATCCTGCTGGAGGGGGTGACCCTGTACTATATCAACACCTATT

C--------------------------------------------

>969_Tubifex_tubifex_T11

---AACTCTATATATCATTTTCGGGATTTGAGCTGGGATGGTCGGGACAGGAACTAGACT

CTTAATTCGTCTAGAACTGGCTCAACCTGGCTCATTCCTTGGAAGGGATCAGCTATACAA

CACCCTAGTTACAGCACATGCATTTCTGATAATTTTCTTCATGGTAATACCTATCTATAT

TGGGGGTTTCGGAAATTGGTTAGTACCCTTAATGCTGGGAGCTCCTGACATAGCATTCCC

CCGATTAAATAATTTAAGATTTTGGTTATTACCCCCTTCCTTAATCCTCCTTGTATCCTC

CGCGGCCGTAGAAAAGGGGGCTGGAACAGGGTGAACAGTATATCCGCCCCTAGCTAGAAA

TCTGGCTCATTCTGGGCCTTCTGTAGACTTAGCTATTTTCTCTCTACATTTAGCTGGGGT

GGCATCGATTCTAGGAGCTATTAACTTTATTACTACAATAATTAACATGCGATGAAAAGG

GATACGCCTTGAACGAATTCCCCTATTTGTATGAGCTGTAATTCTAACCGTAATCTTACT

TTTACTAACATTACCAGTTTTAGCTGGTGCCATTACCATGCTACTAACAGATCGAAATCT

AAACACATCCTTCTTTGACCCTGCTGGGGGTGGAGATCCTGTGCTCTATCAACACTTATT

C--------------------------------------------

>970_Tubifex_tubifex_T12

---AACTCTATACATAATTTTCGGAATCTGAGCTGGTATGGTTGGAACTGGAACTAGGCT

ATTAATTCGCCTTGAACTTGCCCAACCCGGGTCCTTTTTAGGAAGAGATCAATTATATAA

TACTCTGGTAACGGCTCATGCATTTTTAATAATCTTCTTCATGGTGATACCAATCTATAT

TGGGGGCTTCGGCAACTGACTAGTACCATTAATACTGGGGGCTCCAGACATGGCCTTCCC

ACGATTAAACAACTTAAGATTCTGACTCCTACCCCCATCATTAATCCTTTTAGTATCCTC

TGCCGCCGTTGAAAAAGGCGCCGGAACTGGCTGAACTGTTTACCCCCCTCTAGCTAGAAA

TCTAGCTCACTCTGGGCCCTCAGTAGATTTAGCAATCTTTTCTCTACACTTAGCTGGTGT

TGCATCAATCCTGGGTGCAATCAACTTCATTACCACAATAATCAACATACGATGAAAAGG

GATGCGACTAGAACGTATTCCTTTATTTGTATGATCAGTAATTATCACTGTAATTCTACT

CCTACTCACACTTCCAGTTTTAGCTGGGGCTATCACAATACTTCTAACAGACCGGAATCT

AAATACATCATTTTTCGATCCTGCTGGAGGGGGTGACCCTGTACTATATCAACACCTATT

C--------------------------------------------

>971_Tubifex_tubifex_T11

---AACTCTATATATCATTTTCGGGATTTGAGCTGGGATGGTCGGGACAGGAACTAGACT

CTTAATTCGTCTAGAACTGGCTCAACCTGGCTCATTCCTTGGAAGGGATCAGCTATACAA

CACCCTAGTTACAGCACATGCATTTCTGATAATTTTCTTCATGGTAATACCTATCTATAT

TGGGGGTTTCGGAAATTGGTTAGTACCCTTAATGCTGGGAGCTCCTGACATAGCATTCCC

CCGATTAAATAATTTAAGATTTTGGTTATTACCCCCTTCCTTAATCCTCCTTGTATCCTC

CGCGGCCGTAGAAAAGGGGGCTGGAACAGGGTGAACAGTATATCCGCCCCTAGCTAGAAA

TCTGGCTCATTCTGGGCCTTCTGTAGACTTAGCTATTTTCTCTCTACATTTAGCTGGGGT

GGCATCGATTCTAGGAGCTATTAACTTTATTACTACAATAATTAACATGCGATGAAAAGG

GATACGCCTTGAACGAATTCCCCTATTTGTATGAGCTGTAATTCTAACCGTAATCTTACT

TTTACTAACATTACCAGTTTTAGCTGGTGCCATTACCATGCTACTAACAGATCGAAATCT

AAACACATCCTTCTTTGACCCTGCTGGGGGTGGAGATCCTGTGCTCTATCAACACTTATT

C--------------------------------------------

>972_Limnodrilus_hoffmeisteri_T17

---CACCCTATACATAATCTTTGGCCTTTGAGCAGGAATAGTAGGCACAGGAACTAGACT

TTTAATTCGATTTGAGCTAGCACAACCCGGCTCATTTCTCGGTAGAGACCAATTATATAA

CACTCTAGTCACGGCTCACGGATTTTTAATAATTTTCTTTATAGTAATACCTATCTTTAT

TGGGGGATTTGGAAATTGATTAGTGCCTTTAATACTTGGAGCACCTGATATGGCATTCCC

ACGGCTTAATAACCTAAGATTCTGACTAATGCCCCCATCACTAATTCTACTAGTCTCATC

AGCTGCAGTTGAAAAAGGCGCAGGGACAGGGTGAACTGTATACCCCCCCTTAGCCAGAAA

TCTAGCTCATTCCGGGCCTTCTGTAGATCTGGCAATTTTTTCACTACACTTAGCAGGAGC

CGCATCAATCCTGGGAGCAATTAACTTCATCACAACAATAATCAATATACGATGAAAGGG

AATACGCTTAGAGCGTATTCCTTTATTTGTGTGATCCGTAATCATCACAGTTATTTTACT

TCTTCTTACCCTTCCAGTTCTTGCCGGAGCTATCACCATACTTTTAACAGATCGAAACCT

AAACACCTCATTCTTTGATCCGGCAGGTGGAGGCGA------------------------

---------------------------------------------

>973_Lophochaeta_ignota_T6

---AACTCTCTATATAATCTTCGGCCTATGAGCGGGAATAGTCGGCACCGGTACTAGACT

ACTAATTCGACTAGAATTAGCTCAACCCGGATCATTCCTTGGTAGTGATCAACTATATAA

TACACTTGTTACTGCCCATGGATTCCTTATAATTTTCTTCATGGTAATACCAGTATTTAT

TGGGGGATTTGGTAACTGACTAGTCCCATTAATACTTGGTGCCCCAGATATAGCCTTCCC

ACGAATAAATAATTTAAGATTTTGATTAATACCCCCCTCTTTAATCCTTCTCGTATCTTC

AGCAGCCGTTGAAAAAGGTGCTGGAACAGGCTGAACAGTATATCCTCCATTAGCCGGGAA

TTTAGCTCACTCAGGACCATCCGTAGACCTAGCTATTTTCTCACTGCATTTAGCCGGAGC

TGCCTCAATTTTAGGTGCAATTAACTTTATTACGACTATAATTAACATGCGATGAAAAGG

AATACGCCTAGAACGCATTCCTCTATTTGTTTGATCCGTAATTATCACAGTAGTCCTACT

TTTATTAACACTACCAGTTCTAGCAGGGGCCATTACAATATTACTTACAGACCGAAACTT

AAATACCTCATTCTTTGACCCTGCTGGAGGTGGAGACCCTGTTTTATATCAACATCTATT

C--------------------------------------------

>974_Lophochaeta_ignota_T6

---AACTCTCTATATAATCTTCGGCCTATGAGCGGGAATAGTCGGCACCGGTACTAGACT

ACTAATTCGACTAGAATTAGCTCAACCCGGATCATTCCTTGGTAGTGATCAACTATATAA

TACACTTGTTACTGCCCATGGATTCCTTATAATTTTCTTCATGGTAATACCAGTATTTAT

TGGGGGATTTGGTAACTGACTAGTCCCATTAATACTTGGTGCCCCAGATATAGCCTTCCC

ACGAATAAATAATTTAAGATTTTGATTAATACCCCCCTCTTTAATCCTTCTCGTATCTTC

AGCAGCCGTTGAAAAAGGTGCTGGAACAGGCTGAACAGTATATCCTCCATTAGCCGGGAA

TTTAGCTCACTCAGGACCATCCGTAGACCTAGCTATTTTCTCACTGCATTTAGCCGGAGC

TGCCTCAATTTTAGGTGCAATTAACTTTATTACGACTATAATTAACATGCGATGAAAAGG

AATACGCCTAGAACGCATTCCTCTATTTGTTTGATCCGTAATTATCACAGTAGTCCTACT

TTTATTAACACTACCAGTTCTAGCAGGGGCCATTACAATATTACTTACAGACCGAAACTT

AAATACCTCATTCTTTGACCCTGCTGGAGGTGGAGACCCTGTTTTATATCAACATCTATT

C--------------------------------------------

>975_Aulodrilus_pluriseta_T4

---AACTCTATACTTCATTTTCGGAATCTGGGCCGGTATAGTTGGTACTGGTACCAGACT

ATTAATTCGCTTAGAACTGGCACAACCTGGATCCTTCTTAGGAAGTGATCAACTATATAA

TACATTAGTAACAGCTCACGCATTTCTAATAATCTTCTTCCTAGTTATACCTGTATTCAT

CGGGGGATTCGGAAATTGATTAATCCCATTAATACTAGGTGCCCCTGATATAGCATTCCC

ACGACTAAATAACCTAAGATTTTGACTAATACCACCCTCACTAATTCTACTAGTTTCATC

CGCAGCTGTAGAAAAAGGTGCGGGGACAGGTTGAACAGTATATCCACCACTTGCTGGCAA

TCTAGCCCATTCAGGACCTTCCGTAGATCTGGCAATCTTCTCTCTACACTTAGCAGGGGT

AGCATCAATTCTAGGTGCTATTAACTTCATTACCACTATAATCAACATACGATGAAAAGG

AATGCGCCTAGAACGAATTCCTCTATTTGTCTGAGCCGTAATTCTTACCGTTGTACTCCT

TCTATTAACCTTACCAGTTCTGGCGGGGGCAATTACTATACTTCTAACTGACCGCAATCT

AAACACCTCCTTCTTCGATCCAGCTGGGGGCGGTGACCCTGTATTATACCAACATCTATT

C--------------------------------------------

>976_Aulodrilus_pluriseta_T4

---AACTCTATACTTCATTTTCGGGATCTGAGCCGGTATAGTTGGTACCGGTACCAGACT

ATTAATTCGCTTAGAACTGGCACAACCTGGATCCTTCTTAGGAAGTGATCAACTATATAA

TACATTAGTAACAGCTCACGCATTTCTAATAATCTTCTTCCTAGTTATACCTGTATTCAT

CGGGGGATTCGGAAATTGATTAATCCCATTAATACTAGGTGCCCCTGATATAGCATTTCC

ACGACTAAATAACCTAAGATTTTGACTAATACCACCCTCACTAATTCTACTAGTTTCATC

CGCAGCTGTAGAAAAAGGTGCAGGGACAGGTTGAACAGTATATCCACCACTTGCTGGCAA

TCTAGCCCATTCAGGACCCTCCGTAGATCTGGCAATCTTCTCTCTACACTTAGCAGGGGT

AGCATCAATTCTAGGTGCTATTAACTTCATTACCACTATAATCAACATACGATGAAAAGG

AATGCGCCTAGAACGAATTCCTCTATTTGTCTGAGCCGTAATTCTTACCGTTGTACTCCT

TCTATTAACCTTACCAGTTCTGGCAGGGGCAATTACTATACTTCTAACTGACCGCAATCT

AAACACCTCCTTCTTCGATCCAGCTGGGGG------------------------------

---------------------------------------------

>977_Potamothrix_bavaricus_T7

---ACTCCTTTATATAATGTATGGCCTATGAACAGGCATGGTAGGAACTGGAACTAGACT

ACTAATTCGAATGGAACTTGCTCAACCAGGATCATTCCTTGGCAGAGATCAGCTCTATAA

CACTCTCGTAACCGCACACGCATTTCTTATAATCTTTTTCCTTGTTATACCTGTATATAT

TGGTGCCTTCGGAAACTTCCTCGTCCCATTAATACTCGGCGCCCCTGATATAGCATTTCC

ACGGCTAAATAACTTAAGATTTTGACTAATACCCTCATCTGTAATTCTATGCGTGTCATC

AGCTGCTGTTGAAAAAGGAGCCGGTACTGGTTGAACAGTATACCCCCCATTAGCAAGAAA

TCTTGCTCATTCAGGCCCATCTGTCGACTTAGCTATTTTCTCTCTTCACTTAGCAGGTAT

CTCCTCTATTCTAGGGGCGATTAACTTTATTACCACTATAATTAATATGCGTTGAAAAGG

GATGCGCCTAGAACGAATTCCATTATTTGTATGAGCTACTATTATTACAGTAGTATTACT

CCTACTAACTCTTCCTGTCCTGGCCGGAGCTATTACTATACTTCTAACCGACCGTAATCT

AAATACCTCATTCTTCGACCCTGCTGGAGGGGGTGACCCTGTCCTATACCAACACCTGTT

C--------------------------------------------

>978_Potamothrix_bavaricus_T7

---ACTCCTTTATATAATGTATGGCCTATGAACAGGCATGGTAGGAACTGGAACTAGACT

ACTAATTCGAATGGAACTTGCTCAACCAGGATCATTCCTTGGCAGAGATCAGCTCTATAA

CACTCTCGTAACCGCACACGCATTTCTTATAATCTTTTTCCTTGTTATACCTGTATATAT

TGGTGCCTTCGGAAACTTCCTCGTCCCATTAATACTCGGCGCCCCTGATATAGCATTTCC

ACGGCTAAATAACTTAAGATTTTGACTAATACCCTCATCTGTAATTCTATGCGTGTCATC

AGCTGCTGTTGAAAAAGGAGCCGGTACTGGTTGAACAGTATACCCCCCATTAGCAAGAAA

TCTTGCTCATTCAGGCCCATCTGTCGACTTAGCTATTTTCTCTCTTCACTTAGCAGGTAT

CTCCTCTATTCTAGGGGCGATTAACTTTATTACCACTATAATTAATATGCGTTGAAAAGG

AATGCGCCTAGAACGAATTCCATTATTTGTATGAGCTACTATTATTACAGTAGTATTACT

CCTACTAACTCTTCCTGTCCTGGCCGGAGCTATTACTATACTTCTAACCGACCGTAATCT

AAATACCTCATTCTTCGACCCTGCTGGAGGGGGTGACCCTGTCCTATACCAACACCTGTT

C--------------------------------------------

>979_Lophochaeta_ignota_T6

---AACTCTCTATATATTCTTCGGCCTATGAGCGGGAATAGTCGGCACCGGTACTAGACT

ACTAATTCGACTAGAATTAGCTCAACCCGGATCATTCCTTGGTAGTGATCAACTATATAA

TACACTTGTTACTGCCCATGGATTCCTTATAATTTTCTTCATGGTAATACCAGTATTTAT

TGGGGGATTTGGTAACTGACTAGTCCCATTAATACTTGGTGCCCCAGATATAGCCTTCCC

ACGAATAAATAATTTAAGATTTTGATTAATACCCCCCTCTTTAATCCTTCTCGTATCTTC

AGCAGCCGTTGAAAAAGGTGCTGGAACAGGCTGAACAGTATATCCTCCATTAGCCGGGAA

TTTAGCTCACTCAGGACCATCCGTAGACCTAGCTATTTTCTCACTGCATTTAGCCGGAGC

TGCCTCAATTTTAGGTGCAATTAACTTTATTACGACTATAATTAACATGCGATGAAAAGG

AATACGCCTAGAACGCATTCCTCTATTTGTTTGATCCGTAATTATCACAGTAGTCCTACT

TTTATTAACACTACCAGTTCTAGCAGGGGCCATTACAATATTACTTACAGACCGAAACTT

AAATACCTCATTCTTTGACCCTGCTGGAGGTGGAGACCCTGTTTTATATCAACATCTATT

C--------------------------------------------

>980_Lophochaeta_ignota_T6

---AACTCTCTATATAATCTTCGGCCTATGAGCGGGAATAGTCGGCACCGGTACTAGACT

ACTAATTCGACTAGAATTAGCTCAACCCGGATCATTCCTTGGTAGTGATCAACTATATAA

TACACTTGTTACTGCCCATGGATTCCTTATAATTTTCTTCATGGTAATACCAGTATTTAT

TGGGGGATTTGGTAACTGACTAGTCCCATTAATACTTGGTGCCCCAGATATAGCCTTCCC

ACGAATAAATAATTTAAGATTTTGATTAATACCCCCCTCTTTAATCCTTCTCGTATCTTC

AGCAGCCGTTGAAAAAGGTGCTGGAACAGGCTGAACAGTATATCCTCCATTAGCCGGGAA

TTTAGCTCACTCAGGACCATCCGTAGACCTAGCTATTTTCTCACTGCATTTAGCCGGAGC

TGCCTCAATTTTAGGTGCAATTAACTTTATTACGACTATAATTAACATGCGATGAAAAGG

AATACGCCTAGAACGCATTCCTCTATTTGTTTGATCCGTAATTATCACAGTAGTCCTACT

TTTATTAACACTACCAGTTCTAGCAGGGGCCATTACAATATTACTTACAGACCGAAACTT

AAATACCTCATTCTTTGACCCTGCTGGAGGTGGAGACCCTGTTTTATATCAACATCTATT

C--------------------------------------------

>981_Lophochaeta_ignota_T6

---AACTCTCTATATATTCTTCGGCCTATGAGCGGGAATAGTCGGCACCGGTACTAGACT

ACTAATTCGACTAGAATTAGCTCAACCCGGATCATTCCTTGGTAGTGATCAACTATATAA

TACACTTGTTACTGCCCATGGATTCCTTATAATTTTCTTCATGGTAATACCAGTATTTAT

TGGGGGATTTGGTAACTGACTAGTCCCATTAATACTTGGTGCCCCAGATATAGCCTTCCC

ACGAATAAATAATTTAAGATTTTGATTAATACCCCCCTCTTTAATCCTTCTCGTATCTTC

AGCAGCCGTTGAAAAAGGTGCTGGAACAGGCTGAACAGTATATCCTCCATTAGCCGGGAA

TTTAGCTCACTCAGGACCATCCGTAGACCTAGCTATTTTCTCACTGCATTTAGCCGGAGC

TGCCTCAATTTTAGGTGCAATTAACTTTATTACGACTATAATTAACATGCGATGAAAAGG

AATACGCCTAGAACGCATTCCTCTATTTGTTTGATCCGTAATTATCACAGTAGTCCTACT

TTTATTAACACTACCAGTTCTAGCAGGGGCCATTACAATATTACTTACAGACCGAAACTT

AAATACCTCATTCTTTGACCCTGCTGGAGGTGGAGACCCTGTTTTATATCAACATCTATT

C--------------------------------------------

>982_Lophochaeta_ignota_T6

---AACTCTCTATATAATCTTCGGCCTATGAGCGGGAATAGTCGGCACCGGTACTAGACT

ACTAATTCGACTAGAATTAGCTCAACCCGGATCATTCCTTGGTAGTGATCAACTATATAA

TACACTTGTTACTGCCCATGGATTCCTTATAATTTTCTTCATGGTAATACCAGTATTTAT

TGGGGGATTTGGTAACTGACTAGTCCCATTAATACTTGGTGCCCCAGATATAGCCTTCCC

ACGAATAAATAATTTAAGATTTTGATTAATACCCCCCTCTTTAATCCTTCTCGTATCTTC

AGCAGCCGTTGAAAAAGGTGCTGGAACAGGCTGAACAGTATATCCTCCATTAGCCGGGAA

TTTAGCTCACTCAGGACCATCCGTAGACCTAGCTATTTTCTCACTGCATTTAGCCGGAGC

TGCCTCAATTTTAGGTGCAATTAACTTTATTACGACTATAATTAACATGCGATGAAAAGG

AATACGCCTAGAACGCATTCCTCTATTTGTTTGATCCGTAATTATCACAGTAGTCCTACT

TTTATTAACACTACCAGTTCTAGCAGGGGCCATTACAATATTACTTACAGACCGAAACTT

AAATACCTCATTCTTTGACCCTGCTGGAGGTGGAGACCCTGTTTTATATCAACATCTATT

C--------------------------------------------

>983_Nais_communis_N9

---TACATTATATTTAATTCTAGGAGTATGAGCAGGAATAATCGGAACAGGGACAAGAAT

ACTAATTCGAATTGAATTAGCTCAACCCGGAGCCTTCTTAGGAAGAGACCAACTATACAA

CACATTAGTTACTGCACACGCGTTTTTAATAATTTTCTTTCTTGTTATACCTGTATTCAT

TGGTGGATTCGGAAACTGACTTCTACCTCTAATACTTGGTGCACCAGATATAGCATTCCC

ACGACTTAATAATCTTAGGTTCTGATTACTCCCCCCATCATTAATTCTTTTAGTTTCATC

CGCGGCCGTTGAAAAAGGAGCCGGAACAGGTTGAACTGTTTACCCACCACTATCAAGAAA

CCTGGCCCATGCAGGACCATCTGTAGACATGGCTATTTTCTCACTACATTTAGCCGGGGC

ATCATCTATTTTAGGAGCAGTAAATTTTATTACAACAGTAATAAATATACGATGAAACGG

TATACGATTAGAACGACTACCACTATTTGTATGAGCTGTAATACTTACTGTAATCCTTCT

ACTACTATCACTACCAGTTCTAGCAGGAGCAATTACTATACTATTAACAGACCGTAATTT

AAATACATCATTCTTCGACCCCGCTGGAGGGGGAGACCCAATCTTATATCAACATTTATT

C--------------------------------------------

>984_Nais_stolci_pardalis_N12

---TACACTATATCTAATTTTAGGAGTATGAGCAGGAATAGTAGGAACTGGTACAAGATT

ATTAATTCGAATTGAACTATCACAACCAGGATCATTTCTTGGAAGAGATCAACTATATAA

TACTCTCGTAACAGCCCACGCATTCTTAATAATTTTCTTCTTAGTAATACCTGTATTTAT

TGGGGGGTTTGGAAACTGACTTCTTCCATTAATACTAGGTGCTGCTGATATGGCATTCCC

ACGACTAAACAACCTTAGATTTTGACTACTACCACCATCACTAATTCTATTAGTTTCTTC

TGCTGCTGTAGAAAAAGGAGCCGGCACAGGATGAACAGTATATCCACCACTATCAAGAAA

TCTAGCCCATGCTGGACCTTCAGTAGATATGGCTATTTTTTCACTTCATTTAGCAGGTGC

TTCTTCTATTTTAGGAGCTGTAAACTTTATTACGACTGTAATAAACATGCGTTGAAATGG

AATACGATTAGAACGACTACCACTATTTGTATGAGCTGTATTCCTTACAGTAATTCTTCT

ACTATTATCTCTTCCAGTTCTTGCCGGAGCTATTACAATGCTTCTAACAGACCGAAACCT

TAATACTTCATTCTTCGACCCTGCTGGTGGTGGAGACCCGATCCTTTACCAACACTTATT

C--------------------------------------------

>985_Ophidonais_serpentina_N5

---TACATTATATTTAATCTTAGGAGTATGAGCAGGAATAGTTGGTACAGGAACAAGAAT

ACTGATTCGAATTGAACTAGCTCAACCAGGAGCTTTTCTAGGAAGAGATCAATTATATAA

CACTCTAGTAACAGCACATGCGTTTTTAATAATTTTCTTTTTAGTTATACCTGTATTTAT

TGGCGGATTCGGAAACTGACTTCTTCCATTAATATTAGGTGCTCCAGATATGGCATTCCC

ACGACTAAATAATCTTAGATTCTGACTTCTACCACCATCATTAATTCTATTAATTTCATC

TGCAGCCGTTGAAAAAGGTGCTGGAACAGGATGAACTGTATATCCTCCATTATCAAGAAA

TCTAGCTCACGCTGGACCTTCAGTTGACATGGCTATTTTTTCACTACATCTAGCAGGTGC

ATCTTCTATTTTAGGTGCAGTTAACTTCATTACTACAGTAATAAACATACGATGAAATGG

AATACGACTTGAACGAGTACCATTATTTGTATGAGCTGTAACACTTACTGTAATTCTTCT

TCTTTTATCATTACCTGTATTAGCTGGTGCAATTACCATACTATTAACAGATCGAAATCT

AAATACCTCATTCTTCGATCCTGCAGGAGGGGGAGACCCAATTTTATACCAACATTTATT

C--------------------------------------------

>986_Tubifex_tubifex_T12

---AACCCTATACATAATTTTCGGAATCTGAGCTGGTATGGTTGGAACTGGGACTAGGCT

ATTAATTCGCCTTGAACTCGCCCAACCTGGGTCCTTTTTAGGAAGAGACCAATTATATAA

TACCCTGGTAACGGCTCACGCATTTTTAATAATCTTCTTCATGGTGATACCAATCTATAT

TGGGGGCTTCGGCAACTGACTAGTACCATTAATACTGGGGGCTCCAGACATGGCCTTCCC

ACGATTAAACAACTTAAGATTCTGACTCCTACCCCCATCATTAATCCTTTTAGTGTCCTC

TGCCGCCGTTGAAAAAGGCGCCGGAACTGGCTGAACTGTTTACCCTCCTCTAGCTAGAAA

TCTAGCTCACTCTGGACCCTCAGTAGATTTAGCAATCTTTTCTCTACACTTAGCTGGTGT

TGCATCAATCCTGGGTGCAATCAACTTCATTACTACAATAATCAACATACGATGAAAAGG

TATGCGACTAGAACGTATTCCTTTATTCGTATGATCAGTAATTATCACTGTAATTCTACT

CCTGCTTACACTTCCAGTTTTAGCTGGGGCTATCACAATACTTCTAACAGACCGGAATCT

AAATACATCATTTTTCGATCCTGCTGGAGGGGGTGACCCTGTACTATATCAACACCTATT

C--------------------------------------------

>987_Lophochaeta_ignota_T6

---AACTCTCTATATAATCTTCGGCCTATGAGCGGGAATAGTCGGCACCGGTACTAGACT

ACTAATTCGACTAGAATTAGCTCAACCCGGATCATTCCTTGGTAGTGATCAACTATATAA

TACACTTGTTACTGCCCATGGATTCCTTATAATTTTCTTCATGGTAATACCAGTATTTAT

TGGGGGATTTGGTAACTGACTAGTCCCATTAATACTTGGTGCCCCAGATATAGCCTTCCC

ACGAATAAATAATTTAAGATTTTGATTAATACCCCCCTCTTTAATCCTTCTCGTATCTTC

AGCAGCCGTTGAAAAAGGTGCTGGAACAGGCTGAACAGTATATCCTCCATTAGCCGGGAA

TTTAGCTCACTCAGGACCATCCGTAGACCTAGCTATTTTCTCACTGCATTTAGCCGGAGC

TGCCTCAATTTTAGGTGCAATTAACTTTATTACGACTATAATTAACATGCGATGAAAAGG

AATACGCCTAGAACGCATTCCTCTATTTGTTTGATCCGTAATTATCACAGTAGTCCTACT

TTTATTAACACTACCAGTTCTAGCAGGGGCCATTACAATATTACTTACAGACCGAAACTT

AAATACCTCATTCTTTGACCCTGCTGGAGGTGGAGACCCTGTTTTATATCAACATCTATT

C--------------------------------------------

>988_Lophochaeta_ignota_T6

---AACTCTCTATATAATCTTCGGCCTATGAGCGGGAATAGTCGGCACCGGTACTAGACT

ACTAATTCGACTAGAATTAGCTCAACCCGGATCATTCCTTGGTAGTGATCAACTATATAA

TACACTTGTTACTGCCCATGGATTCCTTATAATTTTCTTCATGGTAATACCAGTATTTAT

TGGGGGATTTGGTAACTGACTAGTCCCATTAATACTTGGTGCCCCAGATATAGCCTTCCC

ACGAATAAATAATTTAAGATTTTGATTAATACCCCCCTCTTTAATCCTTCTCGTATCTTC

AGCAGCCGTTGAAAAAGGTGCTGGAACAGGCTGAACAGTATATCCTCCATTAGCCGGGAA

TTTAGCTCACTCAGGACCATCCGTAGACCTAGCTATTTTCTCACTGCATTTAGCCGGAGC

TGCCTCAATTTTAGGTGCAATTAACTTTATTACGACTATAATTAACATGCGATGAAAAGG

AATACGCCTAGAACGCATTCCTCTATTTGTTTGATCCGTAATTATCACAGTAGTCCTACT

TTTATTAACACTACCAGTTCTAGCAGGGGCCATTACAATATTACTTACAGACCGAAACTT

AAATACCTCATTCTTTGACCCTGCTGGAGGTGGAGACCCTGTTTTATATCAACATCTATT

C--------------------------------------------

>989_Lophochaeta_ignota_T6

---AACTCTCTATATAATCTTCGGCCTATGAGCGGGAATAGTCGGCACCGGTACTAGACT

ACTAATTCGACTAGAATTAGCTCAACCCGGATCATTCCTTGGTAGTGATCAACTATATAA

TACACTTGTTACTGCCCATGGATTCCTTATAATTTTCTTCATGGTAATACCAGTATTTAT

TGGGGGATTTGGTAACTGACTAGTCCCATTAATACTTGGTGCCCCAGATATAGCCTTCCC

ACGAATAAATAATTTAAGATTTTGATTAATACCCCCCTCTTTAATCCTTCTCGTATCTTC

AGCAGCCGTTGAAAAAGGTGCTGGAACAGGCTGAACAGTATATCCTCCATTAGCCGGGAA

TTTAGCTCACTCAGGACCATCCGTAGACCTAGCTATTTTCTCACTGCATTTAGCCGGAGC

TGCCTCAATTTTAGGTGCAATTAACTTTATTACGACTATAATTAACATGCGATGAAAAGG

AATACGCCTAGAACGCATTCCTCTATTTGTTTGATCCGTAATTATCACAGTAGTCCTACT

TTTATTAACACTACCAGTTCTAGCAGGGGCCATTACAATATTACTTACAGACCGAAACTT

AAATACCTCATTCTTTGACCCTGCTGGAGGTGGAGACCCTGTTTTATATCAACATCTATT

C--------------------------------------------

>990_Limnodrilus_hoffmeisteri_T18

---CACCTTATATATAATCTTCGGCCTATGAGCCGGAATAGTGGGCACAGGAACAAGCCT

GCTAATTCGATTCGAATTAGCACAACCTGGTTCATTCCTCGGTAGAGATCAACTCTATAA

TACCTTAGTGACAGCCCACGGTTTCCTTATAATCTTCTTCATGGTAATACCAATTTTTAT

TGGTGGCTTCGGAAATTGACTAGTCCCCCTAATGCTAGGAGCTCCTGACATGGCCTTTCC

ACGACTAAATAACCTAAGATTTTGACTAATACCTCCATCACTCATTCTATTAGTTTCATC

AGCCGCAGTCGAAAAGGGAGCGGGGACAGGGTGAACTGTATACCCCCCTCTAGCCAGAAA

CCTGGCGCACTCTGGGCCATCTGTGGATCTAGCAATCTTCTCTCTTCACTTAGCCGGGGC

TGCATCAATTCTAGGTGCCATTAACTTCATTACCACAATAATTAATATACGATGAAAAGG

AATGCGCCTAGAGCGCATTCCTCTATTTGTATGATCAGTAATCATTACAGTTGTCCTCCT

TCTTCTTACATTACCGGTTTTAGCGGGGGCAATCACCATACTTTTAACAGACCGAAACTT

AAACACATCATTCTTCGATCCCTGCGGAGGGGGGGACCCGGTACTATATCAACACTTATT

T--------------------------------------------

>991_Limnodrilus_claparedianus_T22

---CACTCTCTACATAATTTTCGGCCTTTGAGCCGGAATAGTCGGTACTGGAACAAGCCT

ACTAATTCGATTTGAGTTAGCTCAACCCGGGTCATTCTTAGGCAGAGACCAGTTATATAA

CACTTTAGTGACAGCCCACGGATTCTTAATAATTTTCTTTATGGTAATGCCAATCTTTAT

CGGTGGATTTGGAAATTGACTAATTCCCTTAATACTTGGGGCACCAGATATAGCATTCCC

ACGATTAAATAATCTCAGCTTTTGACTAATGCCACCATCGTTAATTCTATTAGTGTCATC

TGCTGCTGTAGAAAAAGGTGCTGGCACAGGTTGAACTGTATATCCGCCACTGGCAAGAAA

TTTAGCACATTCGGGGCCATCTGTAGATTTAGCAATTTTCTCCCTTCATCTTGCTGGTGC

AGCTTCAATTCTAGGGGCAATTAACTTTATTACAACAATAATTAACATGCGATGAAAAGG

AATACGCCTCGAACGAATTCCATTATTCGTATGATCTGTAATTATTACTGTTATTCTACT

ACTTCTGACCCTTCCGGTACTTGCGGGGGCTATCACGATACTATTAACAGACCGAAATCT

AAATACATCGTTCTTCGACCCTGCGGGGGGGGGAGACCCTGTACTTTATCAACACTTATT

C--------------------------------------------

>992_Nais_communis_N9

---TACATTATATTTAATTCTAGGAGTATGAGCAGGAATAATCGGAACAGGGACAAGAAT

ACTAATTCGAATCGAATTAGCTCAACCCGGAGCCTTCTTAGGAAGAGATCAATTATACAA

CACCTTAGTTACTGCACACGCGTTTTTAATAATTTTCTTTCTTGTTATACCTGTATTTAT

TGGTGGATTCGGAAATTGACTTCTACCTCTAATACTAGGTGCACCAGATATAGCATTCCC

ACGACTTAATAATCTTAGATTCTGATTACTTCCGCCATCATTAATTCTTTTAGTTTCATC

AGCGGCCGTTGAAAAAGGAGCCGGAACTGGTTGAACTGTTTACCCACCATTATCAAGAAA

TCTAGCCCATGCAGGACCATCAGTAGACATGGCTATTTTCTCATTACATTTAGCCGGGGC

ATCATCTATTTTAGGAGCAGTTAATTTTATTACAACAGTAATAAATATGCGATGAAATGG

TATACGATTAGAACGACTACCACTATTTGTATGAGCTGTAACACTTACTGTAATCCTTCT

ACTATTATCATTACCAGTTCTAGCAGGAGCAATTACTATGCTATTAACAGACCGTAATTT

AAATACATCATTCTTCGACCCAGCGGGAGGAGGAGACCCAATTCTATATCAACATTTATT

T--------------------------------------------

>993_Ophidonais_serpentina_N5

---TACATTATATTTAATCTTAGGAGTATGAGCAGGAATAGTTGGTACAGGAACAAGAAT

ACTGATTCGAATTGAACTAGCTCAACCAGGAGCTTTTCTAGGAAGAGATCAATTATATAA

CACTCTAGTAACAGCACATGCGTTTTTAATAATTTTCTTTTTAGTTATACCTGTATTTAT

TGGCGGATTCGGAAACTGACTTCTTCCATTAATATTAGGTGCTCCAGATATGGCATTCCC

ACGACTAAATAATCTTAGATTCTGACTTCTACCACCATCATTAATTCTATTAATTTCATC

TGCAGCCGTTGAAAAAGGTGCTGGAACAGGATGAACTGTATATCCTCCATTATCAAGAAA

TCTAGCTCACGCTGGACCTTCAGTTGACATGGCTATTTTTTCACTACATCTAGCAGGTGC

ATCTTCTATTTTAGGTGCAGTTAACTTCATTACTACAGTAATAAACATACGATGAAATGG

AATACGACTTGAACGAGTACCATTATTTGTATGAGCTGTAACACTTACTGTAATTCTTCT

TCTTTTATCATTACCTGTATTAGCTGGTGCAATTACCATACTATTAACAGATCGAAATCT

AAATACCTCATTCTTCGATCCTGCAGGAGGGGGAGACCCAATTTTATACCAACATTTATT

C--------------------------------------------

>994_Ophidonais_serpentina_N5

---TACATTATATTTAATCTTAGGAGTATGAGCAGGAATAGTTGGTACAGGAACAAGAAT

ACTGATTCGAATTGAACTAGCTCAACCAGGAGCTTTTCTAGGAAGAGATCAATTATATAA

CACTCTAGTAACAGCACATGCGTTTTTAATAATTTTCTTTTTAGTTATACCTGTATTTAT

TGGCGGATTCGGAAACTGACTTCTTCCATTAATATTAGGTGCTCCAGATATGGCATTCCC

ACGACTAAATAATCTTAGATTCTGACTTCTACCACCATCATTAATTCTATTAATTTCATC

TGCAGCCGTTGAAAAAGGTGCTGGAACAGGATGAACTGTATATCCTCCATTATCAAGAAA

TCTAGCTCACGCTGGACCTTCAGTTGACATGGCTATTTTTTCACTACATCTAGCAGGTGC

ATCTTCTATTTTAGGTGCAGTTAACTTCATTACTACAGTAATAAACATACGATGAAATGG

AATACGACTTGAACGAGTACCATTATTTGTATGAGCTGTAACACTTACTGTAATTCTTCT

TCTTTTATCATTACCTGTATTAGCTGGTGCAATTACCATACTATTAACAGATCGAAATCT

AAATACCTCATTCTTCGATCCTGCAGGAGGGGGAGACCCAATTTTATACCAACATTTATT

C--------------------------------------------

>995_Tasserkidrilus_kessleri_T33

---AACACTATATATAATCTTTGGATTCTGAGCCGGAATAGTTGGTACTGGAACAAGACT

TTTAATTCGATTTGAATTAGCCCAACCTGGGTCCTTCCTCGGCAGAGATCAACTCTACAA

TACACTAGTTACAGCCCATGCATTCTTAATAATTTTCTTCCTTGTTATGCCAGTCTTCAT

TGGTGGATTTGGCAATTGATTAGTTCCCTTAATACTAGGAGCTCCTGATATAGCATTCCC

ACGATTAAATAATTTAAGATTCTGACTACTACCCCCCTCCCTGATCCTACTAGTATCCTC

GGCCGCCGTAGAAAAGGGGGCAGGAACAGGATGAACAGTTTATCCACCATTAGCTGGCAA

TTTAGCCCATTCCGGCCCATCAGTAGACCTGGCTATTTTCTCTCTTCACCTGGCAGGAAT

CGCTTCTATTTTAGGGGCAATTAATTTTATTACTACAATAATTAATATACGATGAAAAGG

TATACGTCTAGAACGTATCCCTCTATTTGTATGAGCAGTAATCCTGACAGTAATTCTTCT

TCTCCTAACTCTCCCTGTCCTAGCAGGTGCTATTACTATACTCCTAACTGACCGAAATCT

AAATACATCATTCTTTGACCCTGCTGGGGGTGGTGATCCTGTGCTATATCAACATCTATT

T--------------------------------------------

>996_Tasserkidrilus_kessleri_T33

---AACACTATATATAATCTTTGGATTCTGAGCCGGAATAGTTGGTACTGGAACAAGACT

TTTAATTCGATTTGAATTAGCCCAACCTGGGTCCTTCCTCGGCAGAGATCAACTCTACAA

TACACTAGTTACAGCCCATGCATTCTTAATAATTTTCTTCCTTGTTATGCCAGTCTTCAT

TGGTGGATTTGGCAATTGATTAGTTCCCTTAATACTAGGAGCTCCTGATATAGCATTCCC

ACGATTAAATAATTTAAGATTCTGACTACTACCCCCCTCCCTGATCCTACTAGTATCCTC

GGCCGCCGTAGAAAAGGGGGCAGGAACAGGATGAACAGTTTATCCACCATTAGCTGGCAA

TTTAGCCCATTCCGGCCCATCAGTAGACCTGGCTATTTTCTCTCTTCACCTGGCAGGAAT

CGCTTCTATTTTAGGGGCAATTAATTTTATTACTACAATAATTAATATACGATGAAAAGG

TATACGTCTAGAACGTATCCCTCTATTTGTATGAGCAGTAATCCTGACAGTAATTCTTCT

TCTCCTAACTCTCCCTGTCCTAGCAGGTGCTATTACTATACTCCTAACTGACCGAAATCT

AAATACATCATTCTTTGACCCTGCTGGGGGTGGTGATCCTGTGCTATATCAACATCTATT

T--------------------------------------------

>997_Tubifex_tubifex_T12

---AACTCTATACATAATTTTCGGAATCTGAGCTGGTATGGTTGGAACTGGAACTAGGCT

ATTAATTCGCCTTGAACTTGCCCAACCCGGGTCCTTTTTAGGAAGAGATCAATTATATAA

TACTCTGGTAACGGCTCATGCATTTTTAATAATCTTCTTCATGGTGATACCAATCTATAT

TGGGGGCTTCGGCAACTGACTAGTACCATTAATACTGGGGGCTCCAGACATGGCCTTCCC

ACGATTAAACAACTTAAGATTCTGACTCCTACCCCCATCATTAATCCTTTTAGTATCCTC

TGCCGCCGTTGAAAAAGGCGCCGGAACTGGCTGAACTGTTTACCCCCCTCTAGCTAGAAA

TCTAGCTCACTCTGGGCCCTCAGTAGATTTAGCAATCTTTTCTCTACACTTAGCTGGTGT

TGCATCAATCCTGGGTGCAATCAACTTCATTACCACAATAATCAACATACGATGAAAAGG

GATGCGACTAGAACGTATTCCTTTATTTGTATGATCAGTAATTATCACTGTAATTCTACT

CCTACTCACACTTCCAGTTTTAGCTGGGGCTATCACAATACTTCTAACAGACCGGAATCT

AAATACATCATTTTTCGATCCTGCTGGAGGGGGTGACCCTGTACTATATCAACACCTATT

C--------------------------------------------

>998_Lophochaeta_ignota_T6

---AACTCTCTATATAATCTTCGGCCTATGAGCGGGAATAGTCGGCACCGGTACTAGACT

ACTAATTCGACTAGAATTAGCTCAACCCGGATCATTCCTTGGTAGTGATCAACTATATAA

TACACTTGTTACTGCCCATGGATTCCTTATAATTTTCTTCATGGTAATACCAGTATTTAT

TGGGGGATTTGGTAACTGACTAGTCCCATTAATACTTGGTGCCCCAGATATAGCCTTCCC

ACGAATAAATAATTTAAGATTTTGATTAATACCCCCCTCTTTAATCCTTCTCGTATCTTC

AGCAGCCGTTGAAAAAGGTGCTGGAACAGGCTGAACAGTATATCCTCCATTAGCCGGGAA

TTTAGCTCACTCAGGACCATCCGTAGACCTAGCTATTTTCTCACTGCATTTAGCCGGAGC

TGCCTCAATTTTAGGTGCAATTAACTTTATTACGACTATAATTAACATGCGATGAAAAGG

AATACGCCTAGAACGCATTCCTCTATTTGTTTGATCCGTAATTATCACAGTAGTCCTACT

TTTATTAACACTACCAGTTCTAGCAGGGGCCATTACAATATTACTTACAGACCGAAACTT

AAATACCTCATTCTTTGACCCTGCTGGAGGTGGAGACCCTGTTTTATATCAACATCTATT

C--------------------------------------------

>999_Limnodrilus_hoffmeisteri_T17

---CACCCTATACATAATCTTTGGCCTTTGAGCAGGAATAGTAGGCACAGGAACTAGACT

TTTAATTCGATTTGAGCTAGCACAACCCGGCTCATTTCTCGGTAGAGACCAATTATATAA

CACTCTAGTCACGGCTCACGGATTTTTAATAATTTTCTTTATAGTAATACCTATCTTTAT

TGGGGGATTTGGAAATTGATTAGTGCCTTTAATACTTGGAGCACCTGATATGGCATTCCC

ACGGCTTAATAACCTAAGATTCTGACTAATGCCCCCATCACTAATTCTACTAGTCTCATC

AGCTGCAGTTGAAAAAGGCGCAGGGACAGGGTGAACTGTATACCCCCCCTTAGCAAGAAA

TCTAGCTCATTCCGGGCCTTCTGTAGATCTGGCAATTTTTTCACTACACTTAGCAGGAGC

CGCATCAATCCTGGGAGCAATTAACTTCATCACAACAATAATCAATATACGATGAAAGGG

AATACGCTTAGAGCGTATTCCTTTATTTGTGTGATCCGTAATCATCACAGTTATTTTACT

TCTTCTTACCCTTCCAGTTCTTGCCGGAGCTATCACCATACTTTTAACAGATCGAAACCT

AAACACCTCATTCTTTGATCCGGCAGGTGGAGGCGATCCAGTTTTATACCAACATCTATT

---------------------------------------------

>1010_Uncinais_uncinata_N14

---TACACTATACTTAATTTTAGGAGTATGAGCGGGAATAGTAGGAACTGGAACTAGATT

ACTTATTCGAATTGAACTATCACAACCAGGATCATTTCTTGGAAGAGATCAATTATATAA

CACTCTTGTAACAGCACATGCATTCTTAATAATTTTCTTCTTAGTAATACCAGTATTTAT

TGGGGGATTTGGCAACTGACTTCTCCCATTAATACTAGGTGCTGCCGATATAGCATTTCC

TCGATTAAATAATCTTAGATTTTGATTACTTCCACCATCATTAATTCTATTAGTTTCTTC

TGCTGCAGTAGAAAAAGGTGCAGGAACAGGATGAACTGTATACCCACCACTATCAAGAAA

TCTAGCTCACGCTGGCCCTTCTGTAGATATGGCTATTTTTTCACTACACTTAGCTGGTGC

TTCTTCTATTCTAGGAGCAGTTAATTTTATCACCACTGTAATAAATATACGTTGAAACGG

AATACGACTAGAACGACTTCCACTATTTGTTTGATCAGTATTCCTTACAGTAATTCTTCT

CTTATTATCTCTTCCTGTACTTGCTGGTGCAATTACAATACTATTAACAGATCGAAACCT

AAATACCTCATTCTTCGATCCTGCTGGTGGTGGAGACCCTATTCTATATCAACATTTATT

C--------------------------------------------

>1013_Limnodrilus_claparedianus_T22

---CACTCTCTACATAATTTTCGGCCTTTGAGCCGGAATAGTCGGTACTGGAACAAGCCT

ACTAATTCGATTTGAGTTAGCTCAACCCGGGTCATTCTTAGGCAGAGACCAGTTATATAA

CACTTTAGTGACAGCCCACGGATTCTTAATAATTTTCTTTATGGTAATGCCAATCTTTAT

CGGTGGATTTGGAAATTGACTAATTCCCTTAATACTTGGGGCACCAGATATAGCATTCCC

ACGATTAAATAATCTCAGCTTTTGACTAATGCCACCATCGTTAATTCTATTAGTGTCATC

TGCTGCTGTAGAAAAAGGTGCTGGCACAGGTTGAACTGTATATCCGCCACTGGCAAGAAA

TTTAGCACATTCGGGGCCATCTGTAGATTTAGCAATTTTCTCCCTTCATCTTGCTGGTGC

AGCTTCAATTCTAGGGGCAATTAACTTTATTACAACAATAATTAACATGCGATGAAAAGG

AATACGCCTCGAACGAATTCCATTATTCGTATGATCTGTAATTATTACTGTTATTCTACT

ACTTCTGACCCTTCCGGTACTTGCGGGGGCTATCACGATACTATTAACAGACCGAAATCT

AAATACATCGTTCTTCGACCCTGCGGGGGGGGGAGACCCTGTACTTTATCAACACTTATT

C--------------------------------------------

>1016_Limnodrilus_udekemianus_T23

---CACCCTTTACTTTATCTTCGGCCTATGAGCCGGTATAGTTGGTACAGGTACCAGACT

ATTAATTCGATTTGAGCTAGCACAACCCGGATCATTCCTAGGCAGAGACCAACTCTATAA

CACGCTAGTAACAGCACATGGATTTCTAATAATCTTCTTCCTTGTAATACCAGTATTTAT

TGGGGGATTCGGTAATTGATTAGTACCATTAATACTTGGGGCTCCTGACATAGCATTCCC

ACGATTAAATAATCTAAGATTTTGATTAATACCTCCATCACTTATTCTACTTGTATCCTC

AGCAGCAGTGGAAAAAGGAGCTGGAACAGGATGAACTGTATACCCTCCATTAGCAGGAAA

TCTTGCCCATTCCGGACCTTCTGTAGATCTTGCTATTTTCTCTCTTCACCTGGCTGGAAT

TGCCTCAATTCTAGGAGCTATCAACTTTATCACCACTATAATTAATATACGATGAAAAGG

AATACGACTCGAACGAATTCCTTTATTTGTGTGATCTGTAATTATCACTGTAGTCTTACT

TCTACTAACTCTCCCTGTATTAGCAGGGGCAATTACAATGCTCCTAACAGATCGAAATCT

AAATACATCATTTTTTGATCCTGCCGGAGGAGGAGATCCTGTCCTATATCAACACTTATT

C--------------------------------------------

>1017_Limnodrilus_hoffmeisteri_T17

---CACCCTATACATAATCTTTGGCCTTTGAGCAGGAATAGTAGGCACAGGAACTAGACT

TTTAATTCGATTTGAGCTAGCACAACCCGGCTCATTTCTCGGTAGAGACCAATTATATAA

CACTCTAGTCACGGCTCACGGATTTTTAATAATTTTCTTTATAGTAATACCTATCTTTAT

TGGGGGATTTGGAAATTGATTAGTGCCTTTAATACTTGGAGCACCTGATATGGCATTCCC

ACGGCTTAATAACCTAAGATTCTGACTAATGCCCCCATCACTAATTCTACTAGTCTCATC

AGCTGCAGTTGAAAAAGGCGCAGGGACAGGGTGAACTGTATACCCCCCCTTAGCAAGAAA

TCTAGCTCATTCCGGGCCTTCTGTAGATCTGGCAATTTTTTCACTACACTTAGCAGGAGC

CGCATCAATCCTGGGAGCAATTAACTTCATCACAACAATAATCAATATACGATGAAAGGG

AATACGCTTAGAGCGTATTCCTTTATTTGTGTGATCCGTAATCATCACAGTTATTTTACT

TCTTCTTACCCTTCCAGTTCTTGCCGGAGCTATCACCATACTTTTAACAGATCGAAACCT

AAACACCTCATTCTTTGATCCGGCAGGTGGAGGCGATCCAGTTTTATACCAACATCTATT

T--------------------------------------------

>1019_Lumbriculus_variegatus_LL2

---CACTCTATATTTTATTCTTGGCGTCTGAGCCGGCATAGTAGGAGCAGGAATAAGACT

ACTAATCCGAATTGAGCTCACACAACCAGGATCATTTCTAGGCAGAGATCAACTATACAA

TACCATAGTTACCGCACATGCCTTTATTATAATTTTCTTTATAGTAATACCTATATTTAT

TGGCGGATTCGGAAATTGATTACTACCATTAATACTAGGTGCTCCAGACATAGCATTTCC

ACGACTAAATAATCTTAGTTTTTGACTACTACCCCCTTCCCTAATTTTATTAGTAAGATC

AGCTGCAGTAGAAAAAGGAGCAGGAACTGGATGAACTGTATACCCACCTCTAGCAAGTAA

TCTAGCACACGCTGGACCTTCTGTAGATCTAGCTATCTTCTCCCTTCATTTAGCCGGAGC

ATCCTCAATTCTAGGAGCCCTAAACTTTATTACAACTGTTATCAATATACGATGAAATGG

TATACGTCTAGAACGAATTCCTTTATTTGTATGAGCCGTAATAATTACAGTAATTTTACT

CCTATTATCCTTACCGGTATTAGCAGGAGCCATTACTATATTACTAACAGATCGAAACCT

AAACACCTCCTTCTTTGATCCAGCTGGGGGTGGAGACCCTGTCCTATATCAACATCTATT

T--------------------------------------------

>1020_Henlea_perpusilla_E16

---AACACTATATTTCATTCTAGGCGTATGAGCCGGAATGATAGGAGCAGCCATAAGCCT

TCTAATTCGAATTGAACTAAGTCAACCAGGTTCATTCCTCGGAAGAGATCAACTCTACAA

CACTATTGTTACTGCACATGCATTTCTTATAATTTTTTTCCTAGTTATACCTGTATTTAT

TGGGGGGTTCGGAAACTGACTTCTCCCATTAATACTGGGAGCCCCAGATATAGCTTTTCC

CCGACTAAATAACATAAGATTTTGACTTCTTCCCCCATCACTTCTTCTTCTACTTTCCTC

TGCAGCTGTGGAAAAAGGAGCCGGAACCGGCTGAACTGTATACCCCCCCCTATCAAGAAA

TATCGCTCATGCGGGCCCATCTGTAGACCTAGCCATTTTCTCCCTCCACTTAGCCGGAGC

ATCCTCCATCTTAGGTGCAGTAAACTTTATCACTACTGTTATTAACATACGTTGACAAGG

CCTTCACCTAGAACGAATTCCCCTATTTGTATGAGCTGTAACAATTACAGTAGTCCTTCT

CCTCTTATCTTTACCAGTGTTAGCCGGAGCAATTACTATACTACTAACTGACCGAAACCT

AAACACATCCTTTTTCGACCCAGCTGGAGGAGGTGACCCAATTCTATATCAACACCTATT

T--------------------------------------------

>1021_Achaeta_sp_E7

---AACTCTATACTTTATCCTCGGAATATGAGCTGGCATAATAGGTGCAGCTATAAGACT

CCTCATTCGATTTGAACTAAGTCAACCCGGATCATTTTTAGGGAGAGACCAATTATATAA

TACCATTGTAACAGCACATGCATTCTTAATAATTTTCTTTCTTGTAATACCAGTATTTAT

TGGAGGATTTGGAAATTGACTCATTCCATTAATACTTGGAGCTCCAGATATAGCTTTTCC

ACGATTAAATAATATAAGATTCTGATTACTTCCACCATCCCTCATATTACTTCTTTCTTC

TACAGCTGTAGAAAAAGGTATAGGAACAGGATGAACAGTATATCCTCCTTTAGCTAGAAA

CATTGCTCATGCAGGACCATCTGTAGATTTAGCCATTTTCTCTCTTCACTTAGCAGGAGC

TTCATCAATTCTAGGAGCAGTAAATTTCATTACTACTGTAATTAATATACGATGACAAGG

TATACGACTAGAACGAATTCCACTATTTGTATGAGCTATAATAATCACAGTAGCCCTCCT

ACTTTTAGCACTACCAGTCCTAGCTGGCGCAATTACTATGCTTCTTACTGATCGAAACCT

TAACACATCATTCTTTGATCCAGCTGGTGGAGGTGACCCTATTCTATATCAACATTTATT

T--------------------------------------------

>1022_Nais_elinguis_N4

---TACATTATACTTAATTCTAGGAGTATGAGCGGGAATAGTTGGAACTGGAACAAGAAT

ACTAATTCGAATTGAACTTGCTCAACCAGGAGCCTTCCTCGGAAGAGACCAACTCTATAA

CACCCTAGTTACAGCTCATGCATTTTTAATAATTTTCTTTCTTGTAATGCCAGTATTTAT

TGGTGGATTCGGAAATTGACTTCTACCACTAATACTTGGAGCCCCAGATATAGCATTTCC

ACGATTAAATAATCTAAGATTTTGATTACTACCACCATCATTAATTCTACTAATTTCATC

AGCCGCAGTTGAAAAAGGTGCAGGAACAGGATGAACTGTTTACCCTCCATTATCAAGAAA

TTTAGCTCATGCTGGACCATCAGTTGACATGGCTATTTTTTCTCTTCATTTAGCAGGTGC

ATCATCTATTTTAGGTGCAGTAAATTTTATTACTACAGTAATAAATATACGATGAAACGG

TATACGATTAGAACGTTTACCTTTATTTGTATGAGCTGTATTCCTTACTGTAATTCTCCT

TCTACTATCGCTACCAGTATTAGCTGGCGCTATTACAATACTTCTTACAGACCGTAATTT

AAATACCTCATTCTTTGATCCAGCAGGAGGAGGAGATCCGATTCTTTATCAACACTTATT

T--------------------------------------------

>1026_Nais_stolci_pardalis_N12

---TACACTATATCTAATTTTAGGAGTATGAGCAGGAATAGTAGGAACTGGTACAAGATT

ATTAATTCGAATTGAACTATCACAACCAGGATCATTTCTTGGAAGAGATCAACTATATAA

TACTCTCGTAACAGCCCACGCATTCTTAATAATTTTCTTCTTAGTAATACCTGTATTTAT

TGGGGGGTTTGGAAACTGACTTCTTCCATTAATACTAGGTGCTGCTGATATGGCATTCCC

ACGACTAAACAACCTTAGATTTTGACTACTACCACCATCACTAATTCTATTAGTTTCTTC

TGCTGCTGTAGAAAAAGGAGCCGGCACAGGATGAACAGTATATCCACCACTATCAAGAAA

TCTAGCCCATGCTGGACCTTCAGTAGATATGGCTATTTTTTCACTTCATTTAGCAGGTGC

TTCTTCTATTTTAGGAGCTGTAAACTTTATTACGACTGTAATAAACATGCGTTGAAATGG

AATACGATTAGAACGACTACCACTATTTGTATGAGCTGTATTCCTTACAGTAATTCTTCT

ACTATTATCTCTTCCAGTTCTTGCCGGAGCTATTACAATGCTTCTAACAGACCGAAACCT

TAATACTTCATTCTTCGACCCTGCTGGTGGTGGAGACCCGATCCTTTACCAACACTTATT

C--------------------------------------------

>1027_Ophidonais_serpentina_N5

---TACATTATATTTAATCTTAGGAGTATGAGCAGGAATAGTTGGTACAGGAACAAGAAT

ACTGATTCGAATTGAACTAGCTCAACCAGGAGCTTTTCTAGGAAGAGATCAATTATATAA

CACTCTAGTAACAGCACATGCGTTTTTAATAATTTTCTTTTTAGTTATACCTGTATTTAT

TGGCGGATTCGGAAACTGACTTCTTCCATTAATATTAGGTGCTCCAGATATGGCATTCCC

ACGACTAAATAATCTTAGATTCTGACTTCTACCACCATCATTAATTCTATTAATTTCATC

TGCAGCCGTTGAAAAAGGTGCTGGAACAGGATGAACTGTATATCCTCCATTATCAAGAAA

TCTAGCTCACGCTGGACCTTCAGTTGACATGGCTATTTTTTCACTACATCTAGCAGGTGC

ATCTTCTATTTTAGGTGCAGTTAACTTCATTACTACAGTAATAAACATACGATGAAATGG

AATACGACTTGAACGAGTACCATTATTTGTATGAGCTGTAACACTTACTGTAATTCTTCT

TCTTTTATCATTACCTGTATTAGCTGGTGCAATTACCATACTATTAACAGATCGAAATCT

AAATACCTCATTCTTCGATCCTGCAGGAGGGGGAGACCCAATTTTATACCAACATTTATT

C--------------------------------------------

>1028_Enchytraeus_buchholzi_E17

---CACATTATATTTTATTCTAGGAGTGTGGGCCGGAATAATAGGAGCAGCTATAAGCCT

ATTAATTCGAATTGAACTTAGACAACCTGGATCATTCTTAGGAAGAGATCAACTATACAA

CACTATTGTAACAGCACATGCATTTCTAATAATTTTCTTCTTAGTTATACCAGTATTTAT

TGGGGGCTTTGGTAACTGATTACTCCCATTAATATTGGGGGCCCCTGATATGGCCTTCCC

ACGACTAAACAATATAAGATTTTGATTACTTCCCCCAGCACTTATACTGCTTTTATCATC

GGCAGCAGTAGAAAAAGGGGCAGGTACAGGATGAACTGTGTACCCTCCTCTAGCCAGAAA

CATCGCACATGCAGGCCCATCAGTTGACCTAGCAATTTTTTCTCTTCATTTAGCAGGAGC

CTCATCAATTTTAGGAGCTGTAAATTTCATCACTACAGTTATTAATATGCGATGACAAGG

ACTTACACTAGAACGAATTCCCCTTTTCGTTTGAGCAGTTACAATTACAGTAGTGTTACT

ACTCCTATCTTTACCAGTACTAGCTGGAGCAATTACTATGCTACTAACCGATCGAAACCT

AAATACATCATTTTTCGACCCGGCTGGTGGTGGAGACCCAATTCTCTACCAACATTTATT

C--------------------------------------------

>1029_Nais_christinae_N11

---TACACTTTATCTAATTTTAGGAGTATGAGCAGGAATAGTAGGAACCGGAACAAGATT

ACTTATTCGAATTGAATTATCACAACCAGGATCATTTCTTGGAAGAGATCAATTATATAA

TACTCTTGTAACAGCACATGCATTCTTAATAATTTTCTTCTTAGTAATACCTGTATTTAT

TGGGGGGTTCGGAAACTGACTTCTTCCACTAATACTAGGAGCTGCTGATATGGCATTTCC

TCGATTAAACAATCTTAGATTTTGATTACTACCACCTTCATTAATTCTATTAATTTCGTC

TGCAGCAGTAGAAAAAGGTGCAGGAACTGGATGAACTGTATACCCTCCTCTATCTAGAAA

TCTAGCTCATGCTGGGCCTTCAGTAGATATGGCTATTTTCTCACTTCATTTAGCAGGTGC

TTCTTCTATTCTTGGAGCAGTAAATTTTATTACAACTGTAATAAACATACGTTGAAACGG

AATGCGATTAGAACGACTTCCATTATTTGTATGAGCAGTATTTCTTACAGTAATTCTCCT

TCTTCTATCTCTCCCAGTTCTTGCTGGGGCAATCACCATATTACTAACAGATCGAAATCT

AAACACTTCATTCTTTGATCCTGCTGGTGGTGGAGATCCAATTTTATATCAACATTTATT

C--------------------------------------------

>1030_Nais_communis_N9

---TACATTATATTTAATTCTAGGAGTATGAGCAGGAATAATCGGAACAGGGACAAGAAT

ACTAATTCGAATTGAATTAGCTCAACCCGGAGCCTTCTTAGGAAGAGACCAACTATACAA

CACATTAGTTACTGCACACGCGTTTTTAATAATTTTCTTTCTTGTTATACCTGTATTCAT

TGGTGGATTCGGAAACTGACTTCTACCTCTAATACTTGGTGCACCAGATATAGCATTCCC

ACGACTTAATAATCTTAGGTTCTGATTACTCCCCCCATCATTAATTCTTTTAGTTTCATC

CGCGGCCGTTGAAAAAGGAGCCGGAACAGGTTGAACTGTTTACCCACCACTATCAAGAAA

CCTGGCCCATGCAGGACCATCTGTAGACATGGCTATTTTCTCACTACATTTAGCCGGGGC

ATCATCTATTTTAGGAGCAGTAAATTTTATTACAACAGTAATAAATATACGATGAAACGG

TATACGATTAGAACGACTACCACTATTTGTATGAGCTGTAATACTTACTGTAATCCTTCT

ACTACTATCACTACCAGTTCTAGCAGGAGCAATTACTATACTATTAACAGACCGTAATTT

AAATACATCATTCTTCGACCCCGCTGGAGGGGGAGACCCAATCTTATATCAACATTTATT

C--------------------------------------------

>1031_Lumbriculidae_sp_LL3

---AACCTTATATTTCATCTTAGGAGTTTGAGCTGGAATAGTAGGAGCAGGAATAAGACT

ATTAATTCGAGTTGAATTAACACAACCAGGATCATTTTTAGGAAGTGATCAATTATATAA

TACTATTGTTACGGCTCACGCCTTTATTATAATTTTCTTTATAGTTATACCTATATTTAT

TGGTGGCTTTGGTAACTGAATATTACCATTAATATTAGGAGCACCAGATATGGCATTCCC

ACGACTTAACAACCTAAGATTTTGGTTACTCCCGCCTTCTTTAACTTTATTAGTAGCATC

GGCTGCAGTTGAAAAGGGAGCCGGCACAGGATGAACAGTTTATCCACCTCTATCAAGAAA

TTTAGCTCATGCAGGTCCATCAGTAGACCTAGCAATCTTTTCACTACATCTAGCTGGGGC

ATCCTCCATTCTAGGAGCTATTAACTTTATTACTACGGTAATTAATATACGATGAAATGG

ACTCCGACTTGAACGAGTTCCATTATTTGTATGAGCAGTAACAATTACAGTTGTTCTATT

ACTATTATCATTACCAGTACTAGCTGGGGCAATCACTATACTTCTTACAGATCGAAACTT

AAATACAACATTTTTCGATCCGGCTGGTGGTGGAGACCCAGTATTATATCAACATTTATT

---------------------------------------------

>1032_Psammoryctides_barbatus_T8

---TACTTTATATTTAATTTTTGGCTTATGAGCTGGGATGGTAGGAACTGGGACTAGTTT

ATTAATTCGTATAGAACTCGCTCAACCAGGATCATTCCTTGGAAGAGACCAGTTATATAA

CACACTAGTAACAGCACATGCTTTTCTAATAATCTTCTTCCTAGTAATACCCGTATTTAT

TGGTGGGTTTGGTAATTGACTTCTACCTTTAATACTTGGGGCACCAGATATAGCTTTCCC

ACGCCTAAACAATTTAAGATTTTGATTATTACCTCCATCCCTTATTCTTCTAGTATCATC

CGCTGCTGTTGAAAAAGGTGCTGGAACAGGATGAACAGTATATCCACCACTAGCTGGTAA

CCTGGCACACTCTGGCCCTTCTGTAGACCTGGCTATTTTCTCTCTTCATTTAGCTGGTGC

CGCATCTATTTTAGGAGCTATTAATTTTATTACTACTATAATTAATATACGGTGAAAAGG

TATACGATTAGAGCGAATTCCTTTATTTGTGTGAGCTGTAATCATTACAGTAGTTCTTCT

TTTATTAACCCTTCCAGTATTAGCCGGTGCAATTACTATACTACTAACGGATCGAAATCT

AAATACATCATTCTTTGATCCTGCTGGTGGTGGGGATCCTGTTCTTTATCAACACTTATT

C--------------------------------------------

>1033_Stylodrilus_heringianus_LL3

---AACCTTATATTTCATCTTAGGAGTTTGAGCTGGAATAGTAGGAGCAGGAATAAGACT

ATTAATTCGAGTTGAATTAACACAACCAGGATCATTTTTAGGAAGTGATCAATTATATAA

TACTATTGTTACGGCCCACGCCTTTATTATAATTTTCTTTATAGTTATACCTATATTTAT

TGGTGGCTTTGGTAACTGAATATTACCATTAATATTAGGAGCACCAGATATAGCATTCCC

ACGACTTAACAACCTAAGATTTTGGTTACTCCCGCCTTCTTTAACTTTATTAGTAGCATC

GGCTGCAGTTGAAAAGGGAGCCGGCACAGGATGAACAGTTTATCCACCTCTATCAAGAAA

TTTAGCTCATGCAGGTCCATCAGTAGACCTAGCAATCTTTTCACTACATCTAGCTGGGGC

ATCCTCCATTCTAGGAGCTATTAACTTTATTACTACGGTAATTAATATACGATGAAATGG

ACTCCGACTTGAACGAGTTCCATTATTTGTATGAGCAGTAACAATTACAGTTGTTCTATT

ACTATTATCGTTACCAGTACTAGCTGGGGCAATCACTATACTTCTTACAGATCGAAACTT

AAATACAACATTTTTCGATCCGGCTGGTGGTGGAGACCCAGTATTATATCAACATTTATT

T--------------------------------------------

>1037_Pristina_jenkinae_P1

---AACTCTATATTTATCTTCGGGTGTATGAGCAGGAATAGTTGGAACCGGAACAAGACT

ACTCATTCGAGTTGAATTAGCTCAACCAGGCTCATTTCTCGGAAGGGACCAACTTTACAA

TACACTTGTTACTGCACATGCATTCCTAATAATTTTCTTTCTAGTAATGCCAGTATTTAT

TGGAGGATTCGGTAATTGACTTCTTCCATTAATACTAGGAGCACCAGACATGGCATTTCC

ACGACTAAACAACATAAGATTTTGACTACTTCCCCCTGCACTAATTATACTAGTAGCTTC

AGCAGCAGTTGAAAAGGGGGCAGGAACAGGGTGAACAGTATATCCCCCACTTGCAAGAAA

TATTGCTCATGCAGGACCATCTGTAGACATAGCAATTTTTTCTCTTCATCTAGCAGGTGC

ATCATCAATCCTAGGGGCAGTAAACTTTATCTCAACTGTCCTAAATATACGAACTAAAGG

AATACGACTAGAACGAATTCCTCTATTTGTATGAGCTGTATTCTTAACAGTAATCCTACT

ACTTCTGTCACTTCCAGTACTAGCAGGAGCAATTACCATACTTCTTACTGATCGTAACCT

AAATACTTCATTCTTTGACCCAGCTGGGGGTGGTGACCCAATCCTATATCAACATCTATA

T--------------------------------------------

>1038_Nais_alpina_N8

---TACACTATATTTAATTTTAGGAGTATGAGCAGGAATAGTGGGAACTGGAACAAGATT

ACTTATTCGAATTGAACTATCACAACCAGGATCATTTCTTGGAAGAGATCAACTATACAA

CACTCTTGTAACAGCACATGCATTCTTAATAATTTTCTTCTTGGTAATACCAGTATTCAT

TGGGGGGTTTGGAAACTGACTTCTTCCATTAATACTAGGTGCTGCCGATATAGCATTTCC

ACGACTTAATAATCTTAGATTTTGATTGCTACCACCATCATTAATTTTATTAATTTCTTC

TGCAGCTGTAGAAAAAGGTGCTGGAACAGGATGAACTGTTTATCCGCCTCTATCAAGAAA

TCTAGCACATGCCGGACCTTCTGTAGATATGGCTATTTTTTCACTTCATTTAGCAGGTGC

TTCATCTATTTTAGGAGCAGTAAATTTTATTACTACAGTAATAAATATACGATGAAATGG

AATACGACTAGAACGGCTACCATTATTTGTTTGAGCAGTATTTCTTACAGTAATTCTTCT

TTTATTATCTCTTCCAGTACTTGCTGGAGCAATTACAATACTATTAACAGATCGAAATCT

TAATACTTCATTTTTTGATCCTGCTGGAGGTGGGGATCCAATCTTATATCAACATCTATT

T--------------------------------------------

>1039_Nais_pseudobtusa_N13

---TACACTATATTTAATTTTAGGAGTATGAGCAGGAATAGTAGGAACTGGAACTAGATT

ACTTATTCGAATTGAACTATCACAACCAGGATCATTCCTTGGAAGAGATCAATTATATAA

TACTCTTGTAACAGCACATGCATTCTTAATAATTTTCTTCTTAGTAATACCAGTATTTAT

TGGAGGATTCGGTAACTGACTGCTTCCACTAATACTAGGTGCTGCCGATATAGCATTCCC

ACGATTAAACAATCTTAGATTTTGACTTCTTCCACCATCATTAATTCTATTAGTTTCTTC

TGCCGCTGTAGAAAAAGGTGCGGGAACAGGATGAACTGTATATCCACCTCTATCAAGAAA

TCTAGCGCACGCTGGACCTTCTGTTGATATGGCTATTTTTTCACTTCATTTAGCTGGTGC

TTCTTCTATTTTAGGAGCAGTAAATTTTATCACTACTGTAATAAATATACGATGAAATGG

AATACGATTAGAACGACTTCCACTGTTTGTATGAGCTGTATTTCTTACAGTAATTCTTCT

TTTACTTTCTCTTCCAGTTCTTGCTGGTGCAATTACTATACTATTAACTGATCGAAATCT

TAATACTTCATTCTTCGATCCTGCTGGAGGTGGAGATCCAATTCTATATCAACATCTATT

C--------------------------------------------

>1041_Chaetogaster_diastrophus_N15

---CACTCTATACTTAATTTTAGGAGTTTGAGCAGGAATAATTGGTACAGGAACTAGAAT

ACTAATTCGAATTGAACTATCACAACCAGGATCATTCCTTGGGAGAGATCAATTATATAA

CACTCTAGTTACAGCCCATGCATTCCTAATAATTTTCTTCTTAGTGATACCAGTATTCAT

TGGTGGATTCGGAAACTGACTTCTTCCTCTAATACTAGGTGCTCCAGATATGGCATTCCC

ACGACTTAATAATTTAAGATTTTGACTTTTACCTCCATCACTAATTTTACTTATTTCATC

AGCAGCGGTGGAAAAAGGAGCAGGAACAGGATGAACTGTATACCCTCCTCTATCTAGAAA

TCTTGCCCATGCAGGACCGTCCGTAGACATGGCTATTTTTTCTCTTCACTTAGCAGGTGC

TTCATCTATTTTAGGAGCAGTGAATTTTATTACAACTGTAATTAACATACGATGAAACGG

AATACGACTAGAACGACTTCCTCTATTTGTATGAGCAGTATTCTTAACAGTCATTCTTCT

TCTACTTTCTCTTCCAGTACTTGCCGGAGCTATTACTATACTATTAACAGATCGAAACCT

AAATACTTCTTTCTTTGATCCAGCTGGTGGTGGTGACCCTATTCTATACCAACATCTATT

T--------------------------------------------

>1043_Cernosvitoviella_minor_E8

---AACACTTTACTTTATTTTAGGGGTATGAGCAGGAATACTAGGAGCTGCTATAAGACT

TCTAATTCGAATTGAACTAAGACAACCTGGCTCATTCCTAGGGAGAGATCAATTATATAA

TACTATCGTTACAGCACATGCATTCTTAATAATTTTTTTCTTAGTAATACCAGTATTTAT

TGGAGGGTTTGGGAACTGACTTTTACCATTAATATTAGGGGCCCCAGACATGGCCTTTCC

TCGATTAAACAACATAAGATTTTGACTCCTACCTCCATCATTAATACTATTAGTATCTTC

AGCAGCTGTAGAAAAAGGGGCCGGCACTGGATGAACAGTGTACCCCCCTCTTTCTAGAAA

CTTAGCACATGCTGGGCCATCAGTAGATTTAGCAATTTTTTCTCTTCATCTTGCAGGAGC

TTCTTCAATTCTTGGAGCTGTAAACTTTATTACTACAGTAGTGAATATACGTTGACAAGG

CCTTCGGCTAGAACGAATTCCCCTATTTGTATGAGCAGTAGTTATTACAGTAGTTCTACT

ACTTTTATCTCTACCAGTACTAGCAGGAGCAATTACTATACTATTAACAGACCGAAATTT

AAATACATCATTCTTTGATCCGGCAGGAGGAGGAGATCCTATTTTATACCAACACTTATT

C--------------------------------------------

>1047_Limnodrilus_claparedianus_T22

---CACTCTCTACATAGTTTTCGGCCTTTGAGCCGGAATAGTTGGTACTGGGACAAGCCT

ACTAATTCGATTTGAACTAGCTCAACCCGGATCATTTTTAGGCAGGGACCAGTTATATAA

CACTTTAGTGACAGCCCACGGATTCTTAATAATTTTCTTTATAGTGATGCCCATCTTTAT

TGGTGGGTTCGGAAATTGATTAATTCCCTTAATACTTGGGGCACCAGATATAGCATTCCC

ACGATTAAATAATCTCAGCTTTTGACTAATGCCACCATCATTAATTCTATTAGTATCATC

TGCTGCTGTAGAAAAAGGTGCCGGCACAGGTTGAACTGTATATCCACCCCTGGCAAGAAA

TTTAGCACATTCAGGGCCATCTGTAGATTTAGCAATTTTCTCCCTTCATCTTGCTGGTGC

AGCTTCAATTCTAGGGGCAATTAACTTTATTACAACAATAATTAACATACGATGAAAAGG

AATGCGCCTCGAACGAATTCCATTATTTGTATGATCTGTAATTATTACTGTTATTCTACT

ACTTCTAACCCTTCCAGTACTTGCGGGAGCTATCACGATGCTATTAACAGACCGAAATCT

AAATACATCATTCTTCGACCCTGCGGGGGGAGGAGACCCTGTACTTTATCAGCACTTATT

C--------------------------------------------

>1048_Helodrilus_oculatus_LC4

---AACCTTATATTTTATCCTTGGTGTTTGAGCCGGCATAGTAGGAGCTGGCATAAGCCT

TCTCATTCGAATTGAACTAAGACAACCGGGAGCCTTCCTGGGAAGAGATCAACTTTACAA

TACAATTGTTACAGCTCATGCATTCGTAATAATCTTCTTCCTTGTTATGCCCGTATTTAT

TGGAGGATTTGGAAATTGGCTTCTTCCATTAATACTGGGTGCCCCTGATATGGCGTTCCC

CCGACTAAATAACATAAGATTTTGACTACTTCCCCCCTCATTAATCCTCTTAGTTTCCTC

TGCAGCAGTAGAAAAAGGAGCAGGAACCGGCTGAACAATGTATCCACCTTTAGCCAGAAA

TCTTGCCCACGCAGGTCCCTCAGTAGATTTAGCCATTTTTTCCCTACACTTAGCAGGGGC

ATCTTCAATTCTCGGGGCCATCAATTTTATTACAACAGTTATCAACATACGATGAAGAGG

TCTACGTCTAGAACGTATTCCTTTATTCGTCTGAGCCGTTGTAATTACGGTAGTACTCCT

ACTCCTATCTCTTCCAGTGCTGGCAGGGGCAATTACTATGCTCCTCACAGACCGAAACCT

TAATACCTCTTTCTTTGACCCTGCTGGAGGGGGGGATCCTATCTTATACCAACATTTATT

T--------------------------------------------

**ITS2 sequences**

>348 _Limnodrilus_udekemianus_T23

---------AAGAACGCAGCCAGCTGCGTGAATTAATGTGAATTGCAGGACACATTGAAC

ATCGATATCTTGAACGCACATTGCGGGCCTCGGGCAATCCCGAGGCCACGCCTGTCTCAG

GGTCGGTTTAACATATCAATCGTCGGTCGTCCTCAG---AATGCCGACG--CATTGGATC

GTCGTGGCGAGCGGGTGAATGGTACCTTGACCGGGGTTCGAGCGGTGATGTTTTGCGAGC

AGAGCGATTCAGCGTGCA----TTCGCAAACGTCACCGTCTCGAGCTGCCTGCCGCCGCG

TCGTCCGAAGAACAGACGGGACCGCCGCGT-GA--CCACGCCGGCCGCACGTTCTAAGTC

CGCCAGCCGGTGGA-------CGGGGACGGCGACT---------------------CGGA

AGTCGAAATAC-----GATGTCTGCCGTCGCCC-GTCG-TGGCG-----GT-ACGG-TCG

GCGTCGGTCGCGCAATGGACCCACGAAGCTC--------TGCGAACGCGTTAGTACGT-C

GGACTCTCGCGAGTCCGTTCGTGCGC----------------------------------

------------------------------------------------------------

------------------------------------------------------------

-----------------------------------------------------------G

CGTTCATAGACTTCGACCTGAGATCAGACGAGATTACCCGCTGAATTTAAGCATA--

>929_Limnodrilus_udekemianus_T23

-------------------------------ATTAATGTGAAATGCAG-ACACATTGAAC

ATCGA-ATCT-GAACGCACAT-GCGG-CCTCGGGCAATCCCGAGGCCACGCCTGTCTCAG

GGTCGGTTTAACATATCAATCGTCGGTCGTCCTCAG---AATGCCGACG--CATTGGATC

GTCGTGGCGAGCGGGTGAATGGTACCTTGACCGGGGTTCGAGCGGTGATGTTTTGCGAGC

AGAGCGATTCAGCGTGCA----TTCGCAAACGTCACCGTCTCGAGCTGCCTGCCGCCGCG

TCGTCCGAAGAACAGACGGGACCGCCGCGT-GA--CCACGCCGGCCGCACGTTCTAAGTC

CGCCAGCCGGTGGA-------CGGGGACGGCGACT---------------------CGGA

AGTCGAAATAC-----GATGTCTGCCGTCGCCC-GTCG-TGGCG-----GT-ACGG-TCG

GCGTCGGTCGCGCAATGGACCCACGAAGCTC--------TGCGAACGCGTTAGTACGT-C

GGACTCTCGCGAGTCCGTTCGTGCGC----------------------------------

------------------------------------------------------------

------------------------------------------------------------

-----------------------------------------------------------G

CGTTCATAGACTTCGACCTGAGATCAGACGAGATTACCCGCTGAATTTAAGCATAT-

>688_Marionina_sp_E11

-------------------CCAGCTGCGTGAATTAATGTGAATTGCAGGACACATTGAAC

ATCGATATCTTGAACGCATATTGCGG-CCTCGGGCATTCCCGAGGCCACGCCTGTCTCAG

GGTCGGTTACACGTCAATCGCGAAGCCGCTCTCCGGTATGGCTCGCGCAGTGGCGGTCGC

AGGGTTCGCCCT----------------TCGTCCGCTCAAGCA-----------------

------GAG---------------------------------ACGGGTTCAAC---TCGA

TCGTCGG--------------------------------CGAT-----------------

----------------------CGGGACGGTGACTCGACGACTTGTGCGCCGCACCGCAT

CTCCCGGTCGC-----GATCGAGGC-----------------------------------

---------------------GAACCGAACACATCTAACACTGACTTCGACCTGAGA---

TCAGACGAGATTA-CCC-------------------------------------------

------------------------------------------------------------

------------------------------------------------------------

------------------------------------------------------------

---------------------------------------------------------

>689_Marionina_sp_E11

-------------------CCAGCTGCGTGAATTAATGTGAATTGCAGGACACATTGAAC

ATCGATATCTTGAACGCATATTGCGG-CCTCGGGCATTCCCGAGGCCACGCCTGTCTCAG

GGTCGGTTACACGTCAATCGCGAAGCCGCTCTCCGGTATGGCTCGCGCAGTGGCGGTCGC

AGGGTTCGCCCT----------------TCGTCCGCTCAAGCA-----------------

------GAG---------------------------------ACGGGTTCAAC---TCGA

TCGTCGG--------------------------------CGAT-----------------

----------------------CGGGACGGTGACTCGACGACTTGTGCGCCGCACCGCAT

CTCCCGGTCGC-----GATCGAGGC-----------------------------------

---------------------GAACCGAACACATCTAACACTGACTTCGACCTGAGA---

TCAGACGAGATTA-CCCGCTGA----ATT-------------------------------

------------------------------------------------------------

------------------------------------------------------------

------------------------------------------------------------

---------------------------------------------------------

>690_Marionina_sp_E11

------------------------------------------------------TTGAAC

ATCGATATCTTGAACGCATATTGCGGGCCTCGGGCATTCCCGAGGCCACGCCTGTCTCAG

GGTCGGTTACACGTCAATCGCGAAGCCGCTCTCCGGTATGGCTCGCGCAGTGGCGGTCGC

AGGGTTCGCCCT----------------TCGTCCGCTCAAGCA-----------------

------GAG---------------------------------ACGGGTTCAAC---TCGA

TCGTCGG--------------------------------CGAT-----------------

----------------------CGGGACGGTGACTCGACGACTTGTGCGCCGCACCGCAT

CTCCCGGTCGC-----GATCGAGGC-----------------------------------

---------------------GAACCGAACACATCTAACACTGACTTCGACCTGAGA---

TCAGACGAGATTA-CCCGCTGA----ATT-------------------------------

------------------------------------------------------------

------------------------------------------------------------

------------------------------------------------------------

---------------------------------------------------------

>695_Marionina_sp_E11

------------------GCCAGCTGCGTGAATTAATGTGAATTGCAGGACACATTGAAC

ATCGATATCTTGAACGCATATTGCGG-CCTCGGGCATTCCCGAGGCCACGCCTGTCTCAG

GGTCGGTTACACGTCAATCGCGAAGCCGCTCTCCGGTATGGCTCGCGCAGTGGCGGTCGC

AGGGTTCGCCCT----------------TCGTCCGCTCAAGCA-----------------

------GAG---------------------------------ACGGGTTCAAC---TCGA

TCGTCGG--------------------------------CGAT-----------------

----------------------CGGGACGGTGACTCGACGACTTGTGCGCCGCACCGCAT

CTCCCGGTCGC-----GATCGAGGC-----------------------------------

---------------------GAACCGAACACATCTAACACTGACTTCGACCTGAGA---

TCAGACGAGATTA-CCCGCTGA----ATT-------------------------------

------------------------------------------------------------

------------------------------------------------------------

------------------------------------------------------------

---------------------------------------------------------

>697_Marionina_sp_E11

------------------GCCAGCTGCGTGAATTAATGTGAATTGCAGGACACATTGAAC

ATCGATATCTTGAACGCATATTGCGGGCCTCGGGCATTCCCGAGGCCACGCCTGTCTCAG

GGTCGGTTACACGTCAATCGCGAAGCCGCTCTCCGGTATGGCTCGCGCAGTGGCGGTCGC

AGGGTTCGCCCT----------------TCGTCCGCTCAAGCA-----------------

------GAG---------------------------------ACGGGTTCAAC---TCGA

TCGTCGG--------------------------------CGAT-----------------

----------------------CGGGACGGTGACTCGACGACTTGTGCGCCGCACCGCAT

CTCCCGGTCGC-----GATCGAGGC-----------------------------------

---------------------GAACCAAACACATCTAACACTGACTTCGACCTGAGA---

TCNGACGAGATTA-CCC-------------------------------------------

------------------------------------------------------------

------------------------------------------------------------

------------------------------------------------------------

---------------------------------------------------------

>699_Marionina_sp_E11

------------------GCCAGCTGCGTGAATTAATGTGAATTGCAGGACACATTGAAC

ATCGATATCTTGAACGCATATTGCGGGCCTCGGGCATTCCCGAGGCCACGCCTGTCTCAG

GGTCGGTTACACGTCAATCGCGAAGCCGCTCTCCGGTATGGCTCGCGCAGTGGCGGTCGC

AGGGTTCGCCCT----------------TCGTCCGCTCAAGCA-----------------

------GAG---------------------------------ACGGGTTCAAC---TCGA

TCGTCGG--------------------------------CGAT-----------------

----------------------CGGGACGGTGACTCGACGACTTGTGCGCCGCACCGCAT

CTCCCGGTCGC-----GATCGAGGC-----------------------------------

---------------------GAACCGAACACATCTAACACTGACTTCGACCTGAGA---

TCAGACGAGATTA-CCCGGTGA----ATT-------------------------------

------------------------------------------------------------

------------------------------------------------------------

------------------------------------------------------------

---------------------------------------------------------

>701_Marionina_sp_E11

-------------ACGCNGCCAGCTGCGTGAATTAATGTGAATTGCAGGACACATTGAAC

ATCGATATCTTGAACGCATATTGCGG-CCTCGGGCATTCCCGAGGCCACGCCTGTCTCAG

GGTCGGTTACACGTCAATCGCGAAGCCGCTCTCCGGTATGGCTCGCGCAGTGGCGGTCGC

AGGGTTCGCCCT----------------TCGTCCGCTCAAGCA-----------------

------GAG---------------------------------ACGGGTTCAAC---TCGA

TCGTCGG--------------------------------CGAT-----------------

----------------------CGGGACGGTGACTCGACGACTTGTGCGCCGCACCGCAT

CTCCCGGTCGC-----GATCGAGGC-----------------------------------

---------------------GAACCGAACACATCTAACACTGACTTCGACCTGAGA---

TCAGACGAGATTA-CCCGCTGA----A---------------------------------

------------------------------------------------------------

------------------------------------------------------------

------------------------------------------------------------

---------------------------------------------------------

>702_Marionina_sp_E11

-------------------CCAGCTGCGTGAATTAATGTGAATTGCAGGACACATTGAAC

ATCGATATCTTGAACGCATATTGCGG-CCTCGGGCATTCCCGAGGCCACGCCTGTCTCAG

GGTCGGTTACACGTCAATCGCGAAGCCGCTCTCCGGTATGGCTCGCGCAGTGGCGGTCGC

AGGGTTCGCCCT----------------TCGTCCGCTCAAGCA-----------------

------GAG---------------------------------ACGGGTTCAAC---TCGA

TCGTCGG--------------------------------CGAT-----------------

----------------------CGGGACGGTGACTCGACGACTTGTGCGCCGCACCGCAT

CTCCCGGTCGC-----GATCGAGGC-----------------------------------

---------------------GAACCGAACACATCTAACACTGACTTCGACCTGAGA---

TCAGACGAGATTA-CCCGCTGA----ATTTAA---GC-----------------------

------------------------------------------------------------

------------------------------------------------------------

------------------------------------------------------------

---------------------------------------------------------

>707_Marionina_sp_E11

------------AACGCAGCCAGCTGCGTGAATTAATGTGAATTGCAGGACACATTGAAC

ATCGATATCTTGAACGCATATTGCGG-CCTCGGGCATTCCCGAGGCCACGCCTGTCTCAG

GGTCGGTTACACGTCAATCGCGAAGCCGCTCTCCGGTATGGCTCGCGCAGTGGCGGTCGC

AGGGTTCGCCCT----------------TCGTCCGCTCAAGCA-----------------

------GAG---------------------------------ACGGGTTCAAC---TCGA

TCGTCGG--------------------------------CGAT-----------------

----------------------CGGGACGGTGACTCGACGACTTGTGCGCCGCACCGCAT

CTCCCGGTCGC-----GATCGAGGC-----------------------------------

---------------------GAACCGAACACATCTAACACTGACTTCGACCTGAGA---

TCAGACGAGATTA-CCCGCTGA----ATT-------------------------------

------------------------------------------------------------

------------------------------------------------------------

------------------------------------------------------------

---------------------------------------------------------

>708_Marionina_sp_E11

------------AACGCAGCCAGCTGCGTGAATTAATGTGAATTGCAGGACACATTGAAC

ATCGATATCTTGAACGCATATTGCGG-CCTCGGGCATTCCCGAGGCCACGCCTGTCTCAG

GGTCGGTTACACGTCAATCGCGAAGCCGCTCTCCGGTATGGCTCGCGCAGTGGCGGTCGC

AGGGTTCGCCCT----------------TCGTCCGCTCAAGCA-----------------

------GAG---------------------------------ACGGGTTCAAC---TCGA

TCGTCGG--------------------------------CGAT-----------------

----------------------CGGGACGGTGACTCGACGACTTGTGCGCCGCACCGCAT

CTCCCGGTCGC-----GATCGAGGC-----------------------------------

---------------------GAACCGAACACATCTAACACTGACTTCGACCTGAGA---

TCAGACGAGATTA-CCCGCTGA----ATT-------------------------------

------------------------------------------------------------

------------------------------------------------------------

------------------------------------------------------------

---------------------------------------------------------

>712_Marionina_sp_E11

----------AGAACGCAGCCAGCTGCGTGAATTAATGTGAATTGCAGGACACATTGAAC

ATCGATATCTTGAACGCATATTGCGG-CCTCGGGCATTCCCGAGGCCACGCCTGTCTCAG

GGTCGGTTACACGTCAATCGCGAAGCCGCTCTCCGGTATGGCTCGCGCAGTGGCGGTCGC

AGGGTTCGCCCT----------------TCGTCCGCTCAAGCA-----------------

------GAG---------------------------------ACGGGTTCAAC---TCGA

TCGTCGG--------------------------------CGAT-----------------

----------------------CGGGACGGTGACTCGACGACTTGTGCGCCGCACCGCAT

CTCCCGGTCGC-----GATCGAGGC-----------------------------------

---------------------GAACCGAACACATCTAACACTGACTTCGACCTGAGA---

TCAGACGAGATTA-CCCGCTGA----ATT-------------------------------

------------------------------------------------------------

------------------------------------------------------------

------------------------------------------------------------

---------------------------------------------------------
